# Integrating Transcriptomics, Proteomics, and Metabolomics Profiling with System Pharmacology for the Delineation of Long-Term Therapeutic Mechanisms of Bufei Jianpi Formula in Treating COPD

**DOI:** 10.1155/2017/7091087

**Published:** 2017-03-23

**Authors:** Peng Zhao, Jiansheng Li, Ya Li, Yange Tian, Liping Yang, Suyun Li

**Affiliations:** ^1^Henan Key Laboratory of Chinese Medicine for Respiratory Disease, Henan University of Chinese Medicine, Zhengzhou, Henan 450046, China; ^2^Collaborative Innovation Center for Respiratory Disease Diagnosis and Treatment & Chinese Medicine Development of Henan Province, Henan University of Chinese Medicine, Zhengzhou, Henan 450046, China; ^3^Department of Respiratory Diseases, The First Affiliated Hospital of Henan University of Chinese Medicine, Renming Road 19, Zhengzhou 450000, China

## Abstract

In previous work, we identified 145 active compounds from Bufei Jianpi formula (BJF) by system pharmacology and found that BJF showed short-term effect on chronic obstructive pulmonary disease (COPD) rats. Here, we applied the transcriptomic, proteomic, and metabolomics approaches to illustrate the long-term anti-COPD action and its system mechanism of BJF. BJF has obvious anti-COPD effect through decreasing inflammatory cytokines level, preventing protease-antiprotease imbalance and collagen deposition on week 32 by continuous oral administration to rats from weeks 9 to 20. Subsequently, applying the transcriptomic, proteomic, and metabolomics techniques, we detected a number of regulated genes, proteins, and metabolites, mainly related to antioxidant activity, focal adhesion, or lipid metabolism, in lung tissues of COPD and BJF-treated rats. Afterwards, we integrated system pharmacology target, transcript, protein, and metabolite data sets and found that many genes, proteins, and metabolites in rats BJF-treated group and the target proteins of BJF were mainly attributed to lipid metabolism, inflammatory response, oxidative stress, and focal adhesion. Taken together, BJF displays long-term anti-COPD effect probably by system regulation of the lipid metabolism, inflammatory response pathways oxidative stress, and focal adhesion.

## 1. Introduction

Chronic obstructive pulmonary disease (COPD) is a major cause of death with increasing prevalence worldwide, and currently available treatments are largely ineffective [1, 2]. Traditional Chinese medicinal (TCM) formula has provided an effect treating approach to COPD. It is now well recognized that numerous compounds in herbs could act on different targets and show synergistic and long-term effect on complex disease. Bufei Jianpi formula (BJF), containing twelve Chinese herbs, has been used as a therapeutic agent of COPD for a long time. In clinical trial, BJF had beneficial effects on measured outcomes in stable COPD patients [3]. We applied systems pharmacology to identify 145 active compounds from BJF and 175 potential targets. Furthermore, we administrated COPD rats with BJF during weeks 9 to 20 and found that BJF had short-term therapeutic effect on COPD rats on week 20 [4]. Evidence from clinical studies suggested that BJF may have long-term effect on COPD. Herein, we aimed to explore the long-term anti-COPD effect and its mechanism of BJF.

Presently, new approaches allow a complete differential profile including transcriptomic, proteomic, and metabolomic analysis to provide a system level method to investigate the therapeutic mechanisms of TCM formula [5–7]. Transcriptomics is an efficient approach to detect the gene expression profiling, which may yield further insight into biological process of COPD development and medical intervention [8]. Proteomic profiles help dissect the complexity of therapeutic effect of TCM formula by analyzing protein expression, function, modification, and interactions over temporal scales. Metabolomics refers to the analytical approach used to study different cell products that help to understand the physiological and pathological state, which provide data-rich information of metabolic alterations that reflect genetic, epigenetic, and environmental factors influencing cellular physiology [9, 10]. Therefore, integrating transcriptomics, proteomics, and metabolomics studies may help us to get a more in-depth understanding of the complex therapeutic processes of TCM formula.

Here, we treated COPD rats with BJF during weeks 9 to 20 and evaluated the long-term anti-COPD effect of BJF on week 32. We then performed transcriptome, proteome, and metabolome analyses to identify differentially expressed genes, proteins, and metabolites. Finally, we integrated our transcriptomic, proteomic, metabolic, and system pharmacology data to uncover significantly perturbed pathways at both the transcript, protein, and metabolite levels and to dissect the long-term anti-COPD mechanism of BJF at system level.

## 2. Materials and Methods

### 2.1. Chemicals and Animals

Chemicals, such as* Klebsiella pneumoniae*, tobacco, aminophylline, antibodies, and rats, were prepared as described previously [4]. The animal facilities and protocols were used with the permission of the Experimental Animal Care and Ethics Committee (Henan University of Traditional Chinese Medicine).

### 2.2. COPD Rat and BJF Administration

COPD rats and BJF formula were prepared as described previously [4]. Normal saline, BJF (4.44 g/kg), and aminophylline (2.3 mg/kg) were orally administrated to COPD every day during weeks 9 to 20. Normal saline was also orally administrated to control rats. The control and COPD rats were anaesthetized and killed to obtain lung tissues and blood on week 32.

### 2.3. Pulmonary Function and Histological Analyses [4]

We detected the pulmonary function every four weeks during weeks 0 to 32. Lung tissues were harvested for histological and immunohistochemical examination. The specimens were fixed, embedded, cut into sections, and then stained with hematoxylin and eosin. Immunostaining for interleukin- (IL-) 6, IL-1*β*, tumor necrosis factor- (TNF-) *α*, soluble TNF-*α* receptor 2 (sTNFR2), collagen I, collagen III, collagen IV, matrix metalloproteinase- (MMP-) 2, MMP-9, and tissue inhibitor of MMP (TIMP-) 1 followed the methods described previously. The levels of IL-1*β*, IL-6, TNF-*α*, and sTNFR2 in sera were analyzed using ELISA kits.

### 2.4. Gene Expression Analyses

Total RNA was isolated, purified, amplified, and labeled. Finally, the slides were analyzed. Data were collected with Agilent GeneSpring GX software version 11.0 and filtered for significant detection (Student's *t*-test screening, *p* < 0.05) and differential expression versus COPD model rats (fold change, |log ratio| > 1).

### 2.5. Protein Expression Analysis

The lung tissues were lysed and homogenized. The lysates were digested by trypsin solution. Tryptic peptides were labeled. Peptides were fractionated using strong cation exchange fractionation and then used for LC-MS analysis. Fold changes are higher than 1.0 for upregulation or lower than 1.0 for downregulation. Statistical significance was evaluated using one sample *t*-test (*p* values < 0.05, statistically significant).

### 2.6. Metabolites Analysis

Lung tissue was prepared as previously [11]. The tissue metabolic profiling analysis was conducted. PLS-DA in software SIMCA-P (Ver 11.0, Umetric, Umea, Sweden) was applied to analyze the metabolite profiling [12]. Statistical significance was tested by Student's *t*-test (*p* values < 0.05, statistically significant).

### 2.7. Gene, Protein, and Metabolite Network Analyses

Cytoscape v3.1.1 plugins, such as BiNGO and CluGO, were used to analyze the molecular function of transcripts and proteins [13, 14]. Pathway enrichment analysis was performed using the DAVID (regulated pathways, *p* was < 0.05). Integrated pathway analysis of gene, protein, and metabolomics data was analyzed by Metscape [15]. The most relevant pathways of the metabolites were analyzed using MetaboAnalyst 3.0 [16].

### 2.8. Statistical Analysis

One-way analysis of variance with the SPSS 19.0 software package (IBM Corporation, Armonk, NY, USA) was applied to evaluate the statistical differences. Values are expressed as mean ± standard error of mean.

## 3. Results

### 3.1. Long-Term Effect of BJF on COPD Rats

**Figure 1:**
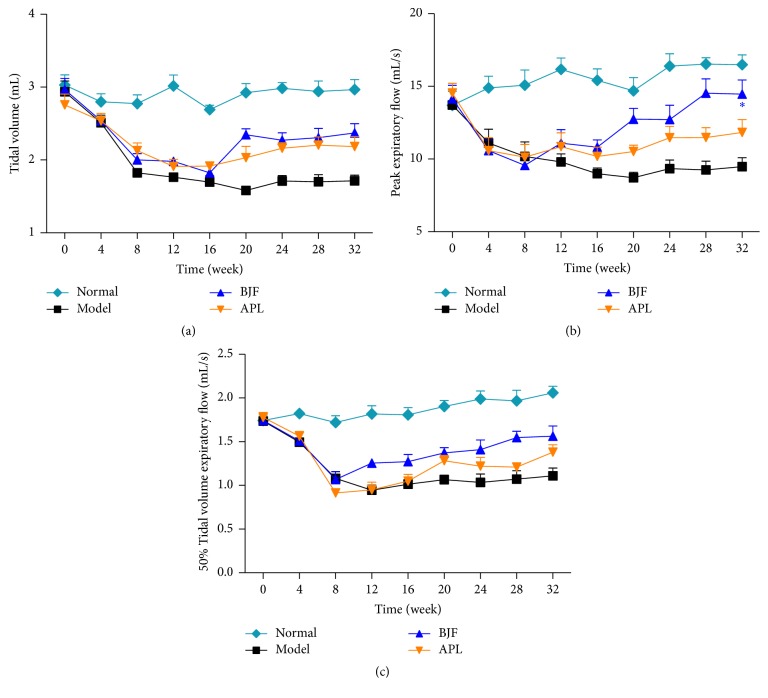
Effect of BJF on the pulmonary function. TV (a), PEF (b), and EF50 (c) were examined every four weeks from weeks 0 to 32. Results were given as means ± SEM and ^*∗*^*p* < 0.05 versus model.

**Figure 2:**
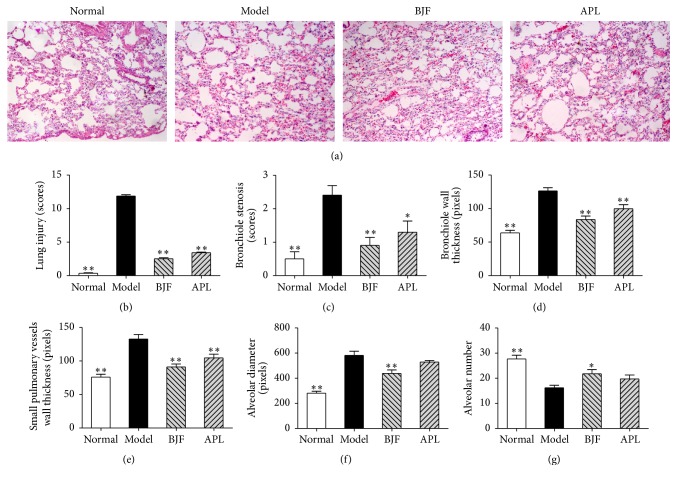
Effects of BJF on pathology changes in each experimental rat group. Histopathologic changes of the lung tissues were tested by hematoxylin and eosin staining on week 32 (magnification, ×100) (a). The lung injury scores (b), bronchiole stenosis (c), bronchial wall thickness (d), small pulmonary vessels wall thickness (e), alveolar diameter (f), and alveolar number (g) were analyzed. Results were given as means ± SEM, ^*∗*^*p* < 0.05, and ^*∗∗*^*p* < 0.01 versus model.

To investigate the long-term anti-COPD action of BJF, we administrated BJF to COPD rats during weeks 9 to 20 and tested the effect of BJF on the functional and morphological changes of lung tissues. In Figure 1, compared with the model group, BJF and aminophylline markedly elevated the TV, PEF, and EF50 in COPD rat at week 32. Meanwhile, lung injury scores, bronchiole wall thickness, small pulmonary vessels wall thickness, bronchiole stenosis, and alveolar diameter markedly elevated in the COPD rats and this elevation was significantly inhibited by BJF (Figures 2(a)–2(f)). Reduction of alveolar number in COPD rats was significantly suppressed by BJF (Figure 2(g)). These findings indicated that BJF showed long-term anti-COPD on COPD rats.

### 3.2. Long-Term Effect of BJF on the Inflammatory Responses in COPD Rats

**Figure 3:**
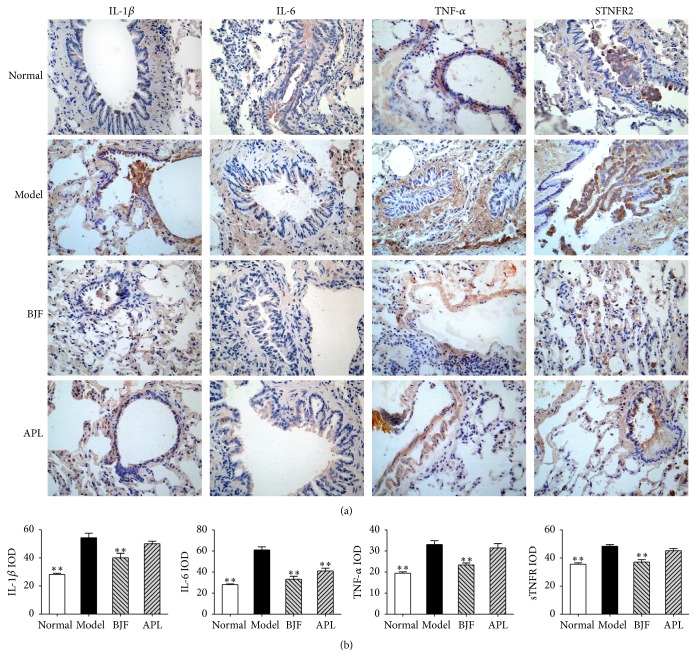
Effect of BJF on IL-1*β*, IL-6 TNF-*α*, and sTNFR2 expression in COPD rat lung tissue. IL-1*β*, IL-6, TNF-*α*, and sTNFR2 in lung tissues were detected with immunohistochemistry (magnification, ×100) on week 32 (a). Quantitative analysis for IL-6, IL-1*β*, TNF-*α*, and sTNFR2 expression was performed (b). Results were given as means ± SEM and ^*∗∗*^*p* < 0.01 versus model.

**Figure 4:**
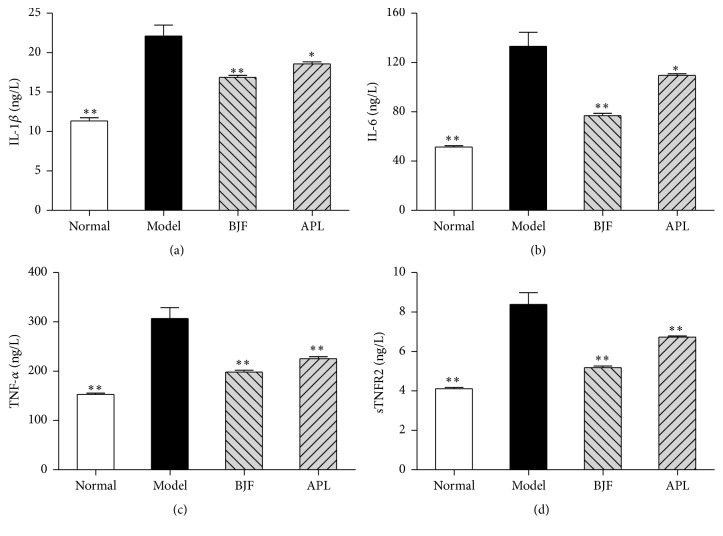
Effect of BJF on IL-1*β*, IL-6 TNF-*α*, and sTNFR2 expression in serum of COPD rats. IL-1*β* (a), IL-6 (b), TNF-*α* (c), and sTNFR2 (d) in serum were detected on week 32. Results were given as means ± SEM, ^*∗*^*p* < 0.05, and ^*∗∗*^*p* < 0.01 versus model.

The inflammatory cytokines are the central mediators in many immune-mediated pulmonary diseases, such as COPD [17–19]. Thus, we evaluated the long-term effect of BJF on the expression levels of inflammatory cytokines in the lung and serum. Compared with model rat, BJF and aminophylline significantly suppressed the levels of IL-1*β*, IL-6, TNF-*α*, and sTNFR2 in lung tissues on week 32 (Figure 3). There are many evidences suggesting the excessive pulmonary inflammation is believed to result in “spill-over” into the systemic inflammation [18]. We then analyzed the levels of IL-1*β*, IL-6, TNF-*α*, and sTNFR2 in serum on week 32. We observed BJF treatment significantly decreased these inflammatory cytokines levels in serum (Figure 4).

### 3.3. Long-Term Effect of BJF on Protease-Antiprotease Imbalance and Collagen Degradation

**Figure 5:**
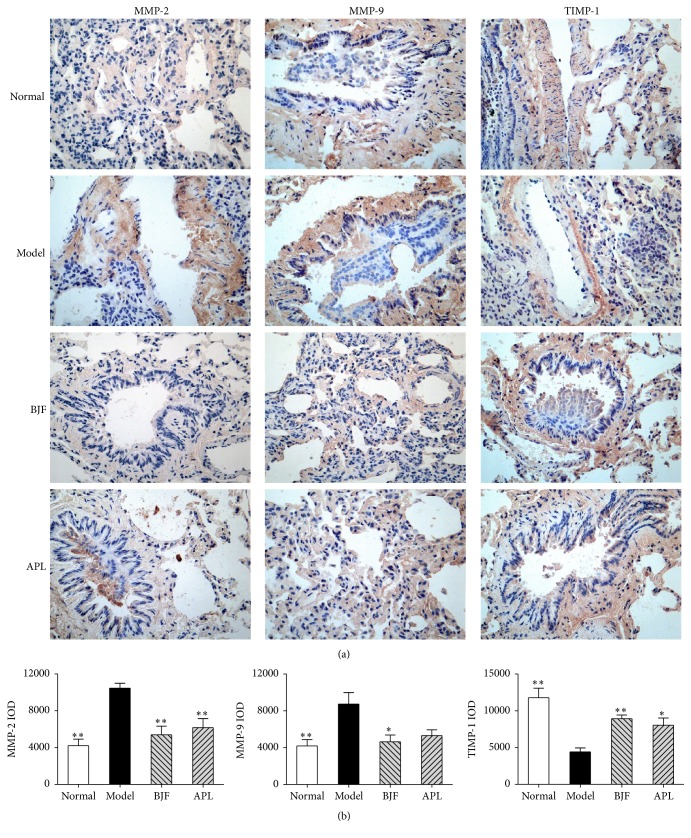
Effect of BJF on MMP-2, MMP-9, and TIMP-1 expression in the COPD rat lung tissue. Immunohistochemical analysis for MMP-2, MMP-9, and TIMP-1 expression in the lung tissues was performed on week 32 (magnification, ×100) (a). Quantitative analysis expression level of MMP-2, MMP-9, and TIMP-1 was quantified based on the immunohistochemical testing (b). Values represent means ± SEM and *n* = 10. ^*∗*^*p* < 0.05 and ^*∗∗*^*p* < 0.01 versus model.

**Figure 6:**
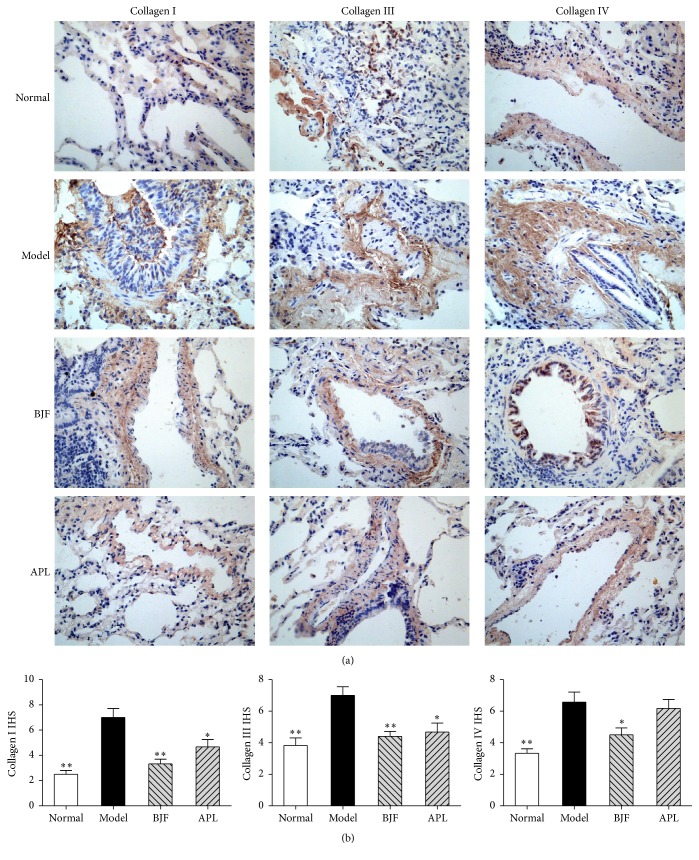
Effect of BJF on expression of collagens I, III, and IV in COPD rat lung tissues. The expression of collagens I, III, and IV was analyzed by immunohistochemistry (magnification, ×100) on week 32 (a). The expression levels of collagens I, III, and IV were quantitatively analyzed (b). Values represent means ± SEM. ^*∗*^*p* < 0.05 and ^*∗∗*^*p* < 0.01 versus model.

The protease-antiprotease imbalance and collagen degradation play significant role in the extensive remodeling of lung tissue structure, which is important pathogenesis of COPD [20–22]. Thus, we tested the long-term effect of BJF on MMP-2, MMP-9, TIMP-1, and collagens I, III, and IV expression. In Figure 5, on week 32, the protein levels of MMP-2/-9 were markedly suppressed by BJF treatment, and the expression of TIMP-1, endogenous inhibitor of MMP, was significantly increased by BJF. The expression of collagens I, III, IV was significantly decreased by BJF treatment (Figure 6).

### 3.4. Molecular Alterations at the Transcript, Protein, and Metabolite Levels

To illustrate the system-wide mechanism of long-term effect of BJF, we detected the transcriptomics, proteomics, and metabolomics profiling of lung tissues.

**Figure 7:**
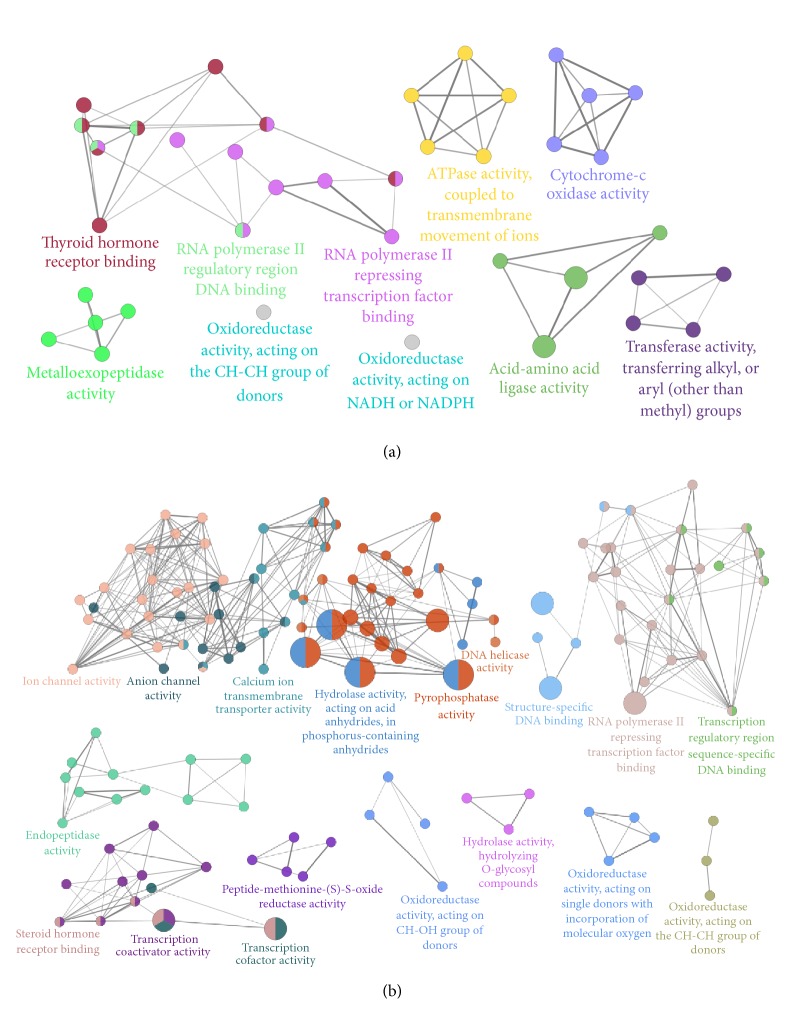
ClueGO analysis of molecular functions of regulated genes in COPD rat and BJF-treated rat lung tissues. (a) The molecular functions of regulated genes in COPD rats. (b) The molecular functions of regulated genes in BJF-treated rats. Functionally grouped network of enriched categories was generated for the regulated genes.

The microarray-based RNA expression analysis was performed. Of the 41000 genes profiled, 976 and 2857 exhibited significant alterations in COPD model rats (versus control) and BJF treatment rats (versus COPD model), respectively (see Supplementary Tables  A1 and A2 available online at https://doi.org/10.1155/2017/7091087). We found these genes were related to many biological functions, such as oxidoreductase, ion channel, or metalloendopeptidase activity (Figure 7). Then, we performed the gene set enrichment analysis and found many significantly altered pathways at mRNA expression level (Tables 1 and 2).

**Figure 8:**
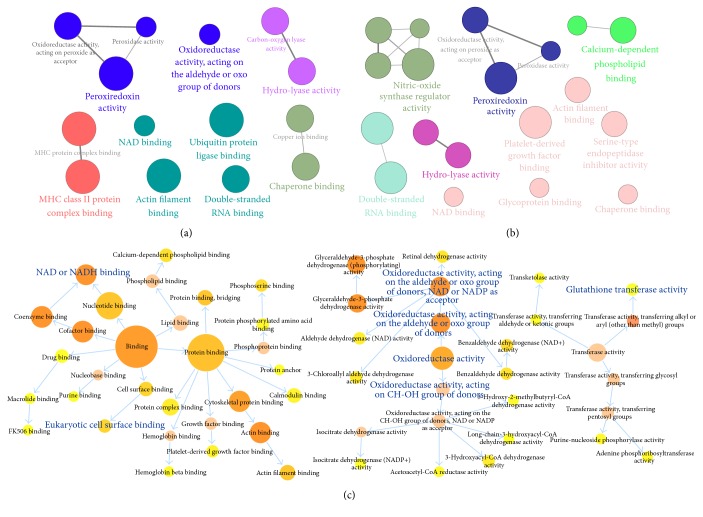
Molecular functions of regulated proteins in COPD rat and BJF-treated rat lung tissues. (a) The molecular functions of regulated proteins in COPD rats, (b) the molecular functions of regulated proteins in BJF-treated rats, and (c) the molecular function of the 127 overlapping proteins.

We then detected the protein expression profile of lung tissues. A total of 191 and 168 differentially expressed proteins were detected in model and BJF-treated rats (Supplementary Tables  A3 and A4). These proteins were mainly involved in these biological functions, such as oxidoreductase activity, peroxiredoxin activity, and nitric-oxide synthase regulator activity (Figures 8(a) and 8(b)), and attributed to many different pathways, such as focal adhesion (Tables 3 and 4).

Furthermore, we observed the COPD model group (191 proteins) shared 127 proteins of the BJF-treated-group (168 proteins). Expression changes of 82 proteins (among the 127 proteins) in COPD model rats were suppressed by BJF (Supplementary Table  A5). The 82 proteins were mainly related to these biological functions, including oxidoreductase, glutathione transferase, NAD or NADH binding, and eukaryotic cell surface binding activity (Figure 8(c)), which was probably relevant for the therapeutic action of BJF.

**Figure 9:**
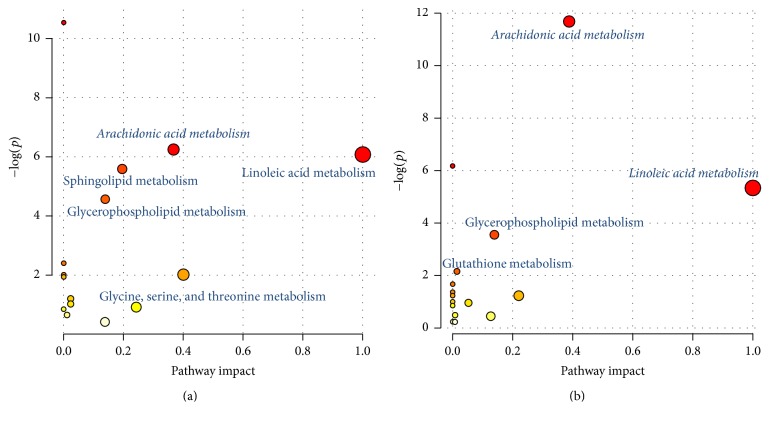
Pathway enrichment of the metabolites regulated in COPD rat and BJF-treated rat lung tissues. (a) Related pathway of the metabolites in COPD rat lung tissues. (b) Related pathway of the metabolites in BJF-treated rat lung tissues.

After metabolomics analysis, 41 and 68 differentially regulated metabolites were identified in model (versus control) and BJF-treated (versus model) rats, respectively (Supplementary Tables  A6 and A7). MetaboAnalyst was applied to analyze metabolic pathway (Tables 5 and 6). We found many significantly altered pathways at the metabolomic level in COPD model and BJF-treated rats, involved in metabolism of arachidonic acid, linoleic acid, glutathione, or glycerophospholipid among others (Figure 9).

### 3.5. System Views on Genes, Proteins, and Metabolites Data

Here, many significantly regulated genes, proteins, and metabolites, related to the therapeutic effect of BJF, were identified. For further holistic analysis, the genes, proteins, and metabolites were integrated to provide a systems biological interpretation.

**Figure 10:**
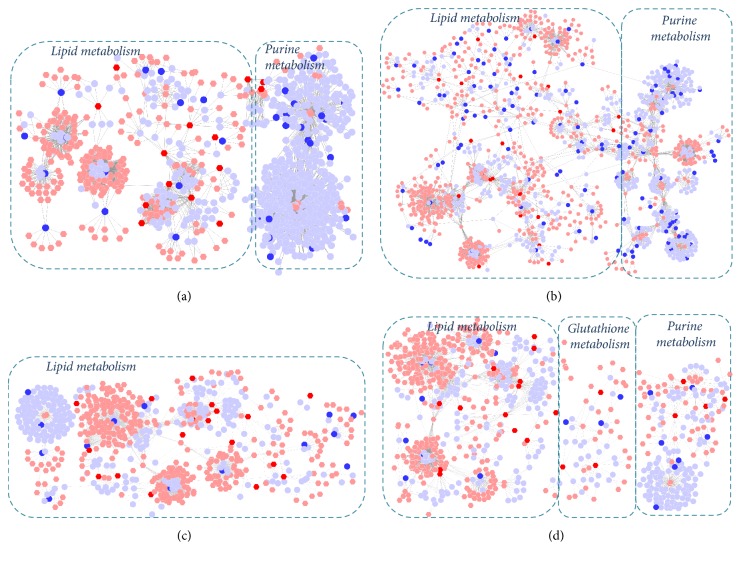
Correlation analysis of genes, proteins, and metabolites regulated in COPD rat and BJF-treated rat lung tissues. Metabolites-gene network analysis of COPD model (a) and BJF-treated group (b). Metabolites-protein network analysis of COPD model (c) and BJF-treated group (d). Networks of the metabolites, genes, and proteins were visualized by Metscape (hexagon node: compounds, round node: metabolic enzymes, and edge: reactions. Red: inputted genes and proteins; blue: inputted compounds).

We firstly built the gene-metabolite networks based on the transcriptomics and metabolomics data of model and BJF-treated rats. We observed these genes and metabolites were primarily involved in lipid, purine, or energy metabolism (Figures 10(a) and 10(b)). Similarly, we constructed metabolite-protein networks, which contained three main parts: lipid metabolism, purine metabolism, or glutathione metabolism (Figures 10(c) and 10(d)). These results suggested that lipid metabolism played an important role in COPD progression and long-term anti-COPD effect of BJF.

### 3.6. Integration of System Pharmacology, Transcriptomics, Proteomics, and Metabolomics Data

We previously identified 175 targets of the BJF using system pharmacology methods (Supplementary Table  A8) and examined the short-term anti-COPD of BJF. Now, the above data indicated that BJF has long-term efficiency for the treatment of COPD rats. Next, we sought to illustrate the system mechanism of the long-term anti-COPD effect of BJF by combining the transcriptomics, proteomics, and metabolomics and system pharmacology data.

**Figure 11:**
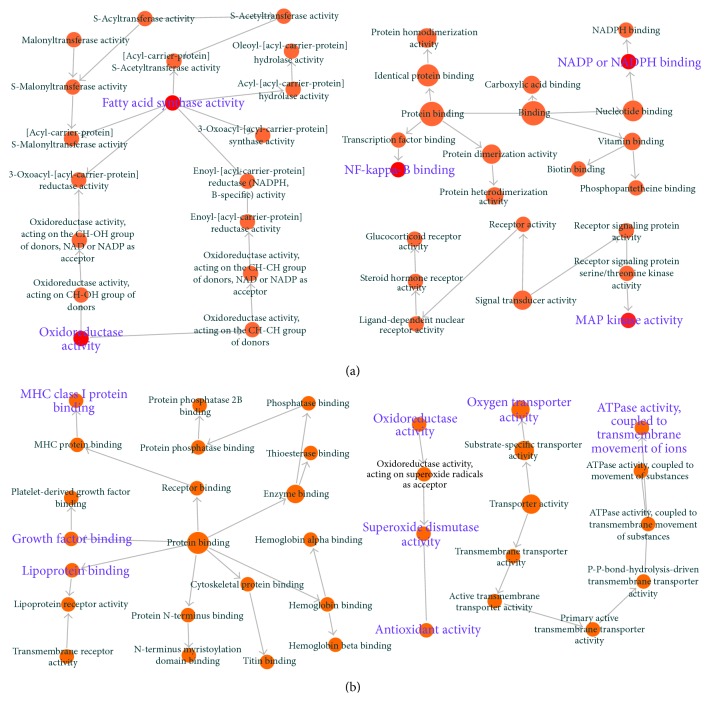
Overlapping proteins' molecular functions. BiNGO was applied to analyze the molecular functions of the overlapping proteins. (a) Molecular function analysis of overlapping proteins between the system pharmacology targets and transcript measurements in lung tissues BJF-treated rats. (b) Molecular function analysis of overlapping proteins between the potential targets and proteins regulated in BJF-treated rat lung tissues.

**Figure 12:**
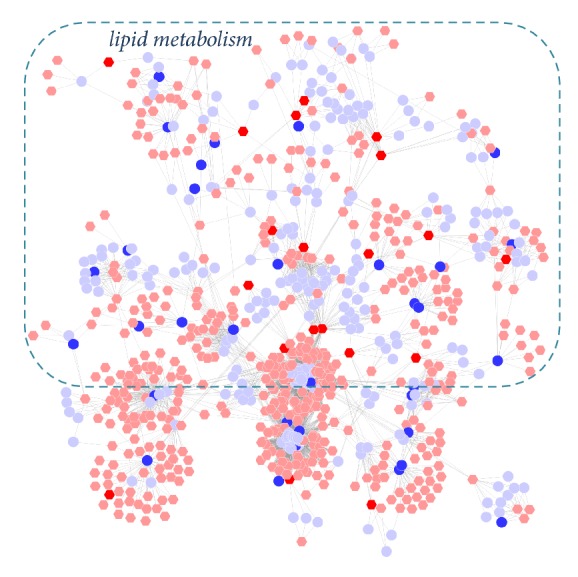
Correlation analysis of metabolites regulated in BJF-treated rats and system pharmacology target. Metscape was used to analyze the metabolite-target network (hexagon node: compounds, round node: metabolic enzymes, and edge: reactions. Red: inputted genes and proteins; blue: inputted compounds).

Initially we explored the biological functions of the overlapping proteins between potential targets of BJF and transcripts in BJF-treated rats. The result showed that 11 overlapping proteins (ACACA, ampC, APP, BCL2, CDK2, FASN, gyrB, katA, KCNH2, MAPK3, and NR3C1) were related to many different biological functions including oxidoreductase activity, MAP kinase activity, and NF-kappa-B binding (Figure 11(a)). Next, 9 overlapping proteins (ATP5B, CALM1, COL1A1, HBB, HSPA5, MGC72973, SOD1, LOC100134871, and LOC689064) between the system pharmacology targets and proteins regulated in BJF-treated rats were identified. The molecular functions of the 9 proteins were mainly related to oxidoreductase, antioxidant, superoxide dismutase activity, and ATPase activity (Figure 11(b)). Furthermore, the potential relationships of target proteins and metabolites regulated in BJF-treated rats were analyzed. In Figure 12, the results showed that metabolite-target protein network mainly contained lipid metabolism.

**Figure 13:**
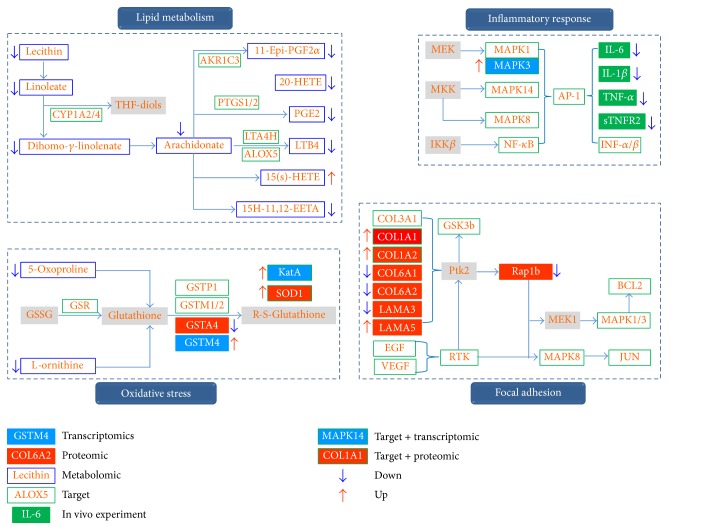
Comprehensive analysis of system pharmacology targets of BJF, transcripts, proteins, and metabolites regulated in BJF-treated rat lung tissues. The targets, transcripts, proteins, and metabolites were presented as rectangles (different colors).

Finally, we constructed a system picture of the molecular mechanisms of long-term anti-COPD effect of BJF based on above comprehensive data. As shown in Figure 13, the comprehensive picture consisted of four parts: lipid metabolism, inflammatory response, oxidative stress, and focal adhesion pathway.

In lipid metabolism analysis, BJF decreased the levels of metabolites, such as lecithin, arachidonate, PGE2, LTB4, and 11-epi-PGF2*α*. We also found these metabolites, including arachidonate, PGE2, and LTB4, elevated in the airways of COPD patients [23–25]. In addition, the critical metabolic enzymes including PTGS1/2, LTA4H, ALOX5, AKR1C3, and CYP1A2/4 were the system pharmacology targets of BJF. Furthermore, the system pharmacology study demonstrated that MAPK1/3 (ERK1/2), MAPK14 (p38), MAPK8 (JNK), and NF-*κ*B were the predicted targets of BJF. We also found that BJF treatment could sustainably decrease the level of IL-1*β*, IL-6 TNF-*α*, and sTNFR2 in lung tissues and serum of COPD rats.

The oxidative stress continuously generates reactive oxygen species, causes lipid peroxidation, and further leads to sustained inflammatory responses, which is a major contributing factor to obstructive disorders in lungs [26–28]. Glutathione (GSH) is one of the main antioxidants, which can provide antioxidant defenses by their ability to exist in reversible oxidized and reduced forms [29, 30]. In addition, the lung has also reliance on superoxide dismutase, catalase, glutathione peroxidase, and glutathione-S-transferase as they are important enzymatic antioxidants of the lungs. Here, we identified the metabolites (5-oxoproline, L-ornithine), transcripts (GSTM4), proteins (GSTA4), and potential targets (GSR, GSTP1, GSTM1/2), which were mainly related to glutathione metabolism. We also found that the antioxidant proteins including SOD1 (superoxide dismutase) and KatA (catalase) were increased by BJF treatment (Supplementary Tables  A2, A4). For correlation analyses on pathway levels, significant pathways from proteomic and potential targets data were integrated. We found focal adhesion was one of the significant deregulated pathways (Tables 3 and 4), and the activation of MAPK1/3, 8, and JUN could increase preinflammatory cytokines expression. Above results implied that BJF achieved its anti-inflammatory activity probably by modulating lipid metabolism, the preinflammatory cytokines production, and their corresponding pathways activation, the metabolism of glutathione, and the expression level of antioxidants.

In summary, these data suggested that BJF showed the long-term anti-COPD therapeutic effect on rats through modulating system biological functions, including lipid metabolism, inflammatory response, oxidative stress, and focal adhesion pathway.

## 4. Discussion

Combining transcriptomics, proteomics, and metabolomics approaches is becoming more important in investigating the mechanisms of the therapeutic effect of Chinese herbal medicine and will most likely change the way we approach investigations of complex disease development. However, attempts thus far to integrate 3-omics data have been met with limited success. In this study, a system level approach based on integrating transcriptomics, proteomics, metabolomics, and system pharmacology data analysis was applied to clarify the long-term anti-COPD effect and its system mechanism of BJF. We administrated COPD rats with BJF during weeks 9 to 20 and then found BJF had long-term anti-COPD effect and could effectively inhibit the inflammatory cytokine expression, protease-antiprotease imbalance, and the collagen deposition on week 32. The transcriptomics, proteomics, and metabolomics profiles model and BJF-treated rats were further characterized, which could help us to dissect the long-term therapeutic mechanism of BJF at system level. The results showed that the regulated transcripts, proteins, and metabolites were related to many different biological functions, including oxidoreductase activity, antioxidant activity, and lipid metabolism. Furthermore, the comprehensive analysis results suggested that BJF showed its long-term anti-COPD effect probably through modulating lipid metabolism, inflammatory response, oxidative stress, and focal adhesion pathways. Taken together, an integrated transcriptomics, proteomics, and metabolomics approach combined with system pharmacology has the potential to considerably advance our understanding of the system mechanism of long-term therapeutic effect of traditional Chinese medicine. However, the main limitation of this work is its direct demand for further experimental validation such as the significant regulated genes, proteins, and metabolites in COPD and BJF-treated rats, which will be resolved in the follow-up works.

## Supplementary Material

Related transcriptomics parameters of lung tissue of COPD and BJF-treated rats were showed in supplementary material.

## Figures and Tables

**Table 1:** Related pathways of transcriptomics detected in COPD rat lung tissue.

Term	Count	%	*p* value
Ribosome	16	0.2530	0.0000
Ubiquitin mediated proteolysis	12	0.1898	0.0008
Neurotrophin signaling pathway	11	0.1740	0.0027
Spliceosome	10	0.1582	0.0067
Long-term potentiation	7	0.1107	0.0108
Oxidative phosphorylation	10	0.1582	0.0145
Non-small cell lung cancer	6	0.0949	0.0147
Renal cell carcinoma	6	0.0949	0.0437
Parkinson's disease	9	0.1423	0.0457
Focal adhesion	11	0.1740	0.0469
GnRH signaling pathway	7	0.1107	0.0480
Alzheimer's disease	11	0.1740	0.0497

**Table 2:** Related pathways of transcriptomics detected in BJF-treated rats lung tissue.

Term	Count	%	*p* value
Ribosome	29	0.174751	6.30*E* − 12
Aminoacyl-tRNA biosynthesis	11	0.066285	7.15*E* − 04
Endocytosis	30	0.180777	7.19*E* − 04
Prostate cancer	17	0.10244	0.001294
Renal cell carcinoma	14	0.084363	0.002109
Ubiquitin mediated proteolysis	20	0.120518	0.00352
Neurotrophin signaling pathway	20	0.120518	0.00352
Adherens junction	14	0.084363	0.004004
Tight junction	20	0.120518	0.005003
Citrate cycle (TCA cycle)	8	0.048207	0.006777
MAPK signaling pathway	32	0.192829	0.013297
Proteasome	10	0.060259	0.013316
Nucleotide excision repair	9	0.054233	0.015824
N-Glycan biosynthesis	9	0.054233	0.018075
Spliceosome	17	0.10244	0.023539
Oxidative phosphorylation	18	0.108466	0.03465
Antigen processing and presentation	13	0.078337	0.037581
Propanoate metabolism	7	0.042181	0.038533
Endometrial cancer	9	0.054233	0.040556
Amino sugar and nucleotide sugar metabolism	8	0.048207	0.045144

**Table 3:** Related pathways of proteins identified in COPD rats lung tissue.

Term	Count	%	*p* value
ECM-receptor interaction	8	0.3666	0.0002
Focal adhesion	11	0.5041	0.0006
Leukocyte transendothelial migration	7	0.3208	0.0069
Glycolysis/gluconeogenesis	6	0.2750	0.0079
Propanoate metabolism	4	0.1833	0.0125
Pyruvate metabolism	4	0.1833	0.0196
Tryptophan metabolism	4	0.1833	0.0254
Valine, leucine, and isoleucine degradation	4	0.1833	0.0303
Small cell lung cancer	5	0.2291	0.0343
Regulation of actin cytoskeleton	8	0.3666	0.0348
Tight junction	6	0.2750	0.0433
Nitrogen metabolism	3	0.1375	0.0450
Pentose phosphate pathway	3	0.1375	0.0487

**Table 4:** Related pathways of proteins identified in BJF-treated rats lung tissue.

Term	Count	%	*p* value
ECM-receptor interaction	9	5.882353	1.71*E* − 05
Focal adhesion	13	8.496732	1.74*E* − 05
Leukocyte transendothelial migration	7	4.575163	0.005802
Regulation of actin cytoskeleton	9	5.882353	0.00959
Huntington's disease	8	5.228758	0.018396
Valine, leucine, and isoleucine degradation	4	2.614379	0.02757
Neurotrophin signaling pathway	6	3.921569	0.03371
Tight junction	6	3.921569	0.03782

**Table 5:** Related pathways of metabolites identified in COPD rat lung tissue.

Term	Total	Expected	Hits	Raw *p*
Biosynthesis of unsaturated fatty acids	42	0.6591	6	2.62*E* − 05
Arachidonic acid metabolism	36	0.5649	4	0.001929
Linoleic acid metabolism	5	0.0785	2	0.002286
Sphingolipid metabolism	21	0.3295	3	0.003718
Glycerophospholipid metabolism	30	0.4708	3	0.010353
Cyanoamino acid metabolism	6	0.0942	1	0.09069
alpha-Linolenic acid metabolism	9	0.1412	1	0.13304
Methane metabolism	9	0.1412	1	0.13304
Fatty acid biosynthesis	43	0.6748	2	0.1443
Steroid hormone biosynthesis	70	1.0984	2	0.3014
Cysteine and methionine metabolism	28	0.4394	1	0.36059
Glycine, serine, and threonine metabolism	32	0.5021	1	0.40061
Steroid biosynthesis	35	0.5492	1	0.42905
Primary bile acid biosynthesis	46	0.7218	1	0.52269
Aminoacyl-tRNA biosynthesis	67	1.0514	1	0.66232

**Table 6:** Related pathways of metabolites identified in BJF-treated rat lung tissue.

Term	Total	Expected	Hits	Raw *p*
Arachidonic acid metabolism	36	0.8217	7	8.52*E* − 06
Biosynthesis of unsaturated fatty acids	42	0.9586	5	0.002093
Linoleic acid metabolism	5	0.1141	2	0.004837
Glycerophospholipid metabolism	30	0.6847	3	0.028867
Glutathione metabolism	26	0.5934	2	0.11684
alpha-Linolenic acid metabolism	9	0.2054	1	0.18812
Fatty acid biosynthesis	43	0.9815	2	0.25705
Selenoamino acid metabolism	15	0.3424	1	0.29397
Histidine metabolism	15	0.3424	1	0.29397
Butanoate metabolism	20	0.4565	1	0.37185
Sphingolipid metabolism	21	0.4793	1	0.38639
Alanine, aspartate, and glutamate metabolism	24	0.5478	1	0.4281
Pyrimidine metabolism	41	0.9358	1	0.61733
Arginine and proline metabolism	44	1.0043	1	0.64371
Aminoacyl-tRNA biosynthesis	67	1.5292	1	0.79506
Purine metabolism	68	1.5521	1	0.79997
