# Supplementary material for: Integrating Transcriptomics, Proteomics, and Metabolomics Profiling with System Pharmacology for the Delineation of Long-Term Therapeutic Mechanisms of Bufei Jianpi Formula in Treating COPD
Source: Biomed Res Int. 2017 Mar 23;2017:7091087. doi: 10.1155/2017/7091087 (PMC5382313; doi:10.1155/2017/7091087)
Supplement: Supplementary file 1 — Related transcriptomics parameters of lung tissue of COPD and BJF-treated rats were showed in supplementary material. [file 7091087.f1.docx]

| Table A1 Analyzed transcriptomics parameters of lung tissue of chronic obstructive pulmonary | | | | |
| --- | --- | --- | --- | --- |
| disease rats | pvalues | foldchange | GeneSymbol | UniGeneID |
| A_42_P456701 | 0.03515 | 1.127492 | Mtfmt | Rn.34645 |
| A_42_P482349 | 0.027215 | 1.064921 | Rab3gap2 | Rn.101730 |
| A_42_P486517 | 0.039335 | 1.132763 | Cox7a2l | Rn.3907 |
| A_42_P489761 | 0.007405 | 1.261399 | Acaca | Rn.44372 |
| A_42_P493785 | 0.018442 | 0.780255 | Cdk2ap1 | Rn.40447 |
| A_42_P503768 | 0.041576 | 1.087292 | Gemin6 | Rn.108103 |
| A_42_P505280 | 0.043282 | 1.243959 | Sae1 | Rn.9014 |
| A_42_P505972 | 0.025619 | 0.803634 | Ube2s | Rn.120398 |
| A_42_P514254 | 0.029306 | 1.516627 |  | Rn.163307 |
| A_42_P516941 | 0.02577 | 0.810462 | Ube4b | Rn.211830 |
| A_42_P519910 | 0.039541 | 1.117108 | Eif3f | Rn.41724 |
| A_42_P529193 | 0.02934 | 0.882425 | Sdhc | Rn.1698 |
| A_42_P530171 | 0.013542 | 0.887525 | Lsm4 | Rn.27306 |
| A_42_P537443 | 0.002938 | 0.726694 | Jund | Rn.46225 |
| A_42_P544321 | 0.001992 | 1.128894 |  | Rn.3287 |
| A_42_P545943 | 0.042025 | 1.295797 | Car2 | Rn.26083 |
| A_42_P549271 | 0.043797 | 1.446957 | Mrpl43 | Rn.225372 |
| A_42_P553885 | 0.008032 | 1.448738 | Synpo | Rn.42910 |
| A_42_P558503 | 0.009133 | 0.847449 | Brd2 | Rn.98146 |
| A_42_P559085 | 0.021889 | 1.159735 | Sos2 | Rn.4226 |
| A_42_P561930 | 0.046472 | 0.826906 |  | Rn.50150 |
| A_42_P574345 | 0.048698 | 1.162109 | LOC100360737 | Rn.224598 |
| A_42_P574960 | 0.035439 | 1.116079 |  | Rn.12130 |
| A_42_P585750 | 0.012566 | 0.680425 | Cd300le-ps1 | |
| A_42_P585995 | 0.032656 | 1.710944 | Nim1 | Rn.19745 |
| A_42_P586681 | 0.008026 | 1.210133 | Upf2 | Rn.106291 |
| A_42_P596050 | 0.023585 | 1.546809 | Cdhr1 | Rn.19780 |
| A_42_P601920 | 0.008114 | 1.165518 | Cpt2 | Rn.11389 |
| A_42_P614547 | 0.0202 | 1.198899 | Ep400 | Rn.76053 |
| A_42_P619403 | 0.008316 | 1.390314 | Fam89a | Rn.66325 |
| A_42_P621826 | 0.034678 | 1.309294 | Tg | Rn.10429 |
| A_42_P621882 | 0.0162 | 0.918947 | Copa | Rn.203109 |
| A_42_P624251 | 0.007683 | 0.634498 | Edc4 | Rn.145173 |
| A_42_P624773 | 0.020928 | 1.322869 | Cpz | Rn.11056 |
| A_42_P639337 | 0.028124 | 0.891253 | Ptges3l1 | Rn.211968 |
| A_42_P651681 | 0.037081 | 1.342788 | Npvf | Rn.62354 |
| A_42_P656468 | 0.029595 | 1.070778 | Rps8 | Rn.3198 |
| A_42_P661837 | 0.005499 | 1.159819 | Crnkl1 | Rn.162694 |
| A_42_P662770 | 0.0067 | 0.82517 | Heatr2 | Rn.16461 |
| A_42_P664238 | 0.013887 | 0.901971 | Vps72 | Rn.1986 |
| A_42_P666663 | 0.039997 | 1.236796 | Fam129b | Rn.4205 |
| A_42_P670631 | 0.02658 | 0.722959 | RGD1305645 | Rn.16593 |
| A_42_P677866 | 0.014401 | 1.549736 | Csn2 | Rn.10745 |
| A_42_P678974 | 0.004564 | 1.292103 | Cox11 | Rn.19469 |
| A_42_P689905 | 0.029023 | 1.646876 | Apoc2 | Rn.16843 |
| A_42_P703751 | 0.023465 | 1.079859 | Lsm12 | Rn.130804 |
| A_42_P707992 | 0.038739 | 0.26302 | Tspan18 | Rn.145602 |
| A_42_P710659 | 0.027085 | 1.235609 | Caly | Rn.27756 |
| A_42_P712718 | 0.048274 | 1.137294 | Mmadhc | Rn.3269 |
| A_42_P736967 | 0.026636 | 0.753564 | Zdhhc18 | Rn.203345 |
| A_42_P745958 | 0.01027 | 1.130487 | Atrn | Rn.53846 |
| A_42_P749968 | 0.000865 | 0.712087 | Smg8 | Rn.45630 |
| A_42_P755414 | 0.001127 | 0.833291 |  |  |
| A_42_P756393 | 0.041086 | 1.144522 |  | Rn.28951 |
| A_42_P763424 | 0.049489 | 1.1595 | Atp11a | Rn.98540 |
| A_42_P767403 | 0.009561 | 1.326326 | Zbtb4 | Rn.15836 |
| A_42_P769290 | 0.00234 | 0.778799 |  | Rn.3440 |
| A_42_P769969 | 0.026017 | 1.201122 | Hmgn5 | Rn.25395 |
| A_42_P770171 | 0.030529 | 1.250647 | Isoc2b | Rn.1804 |
| A_42_P771848 | 0.032065 | 1.155367 | Eif4b | Rn.95954 |
| A_42_P772136 | 0.030147 | 0.823729 | Fam60a | Rn.9154 |
| A_42_P778437 | 0.044548 | 0.934249 | Mrpl37 | Rn.3445 |
| A_42_P803137 | 0.016822 | 0.554815 | Spint2 | Rn.3857 |
| A_42_P803153 | 0.032984 | 1.073458 | Rpl27a | Rn.2722 |
| A_42_P804387 | 0.022901 | 0.789562 | Lypd3 | Rn.38434 |
| A_42_P822588 | 0.02103 | 1.530445 | Alox15 | Rn.11318 |
| A_42_P842842 | 0.046644 | 0.732178 |  | Rn.8938 |
| A_43_P10191 | 0.025919 | 0.808911 | Rnf7 | Rn.2768 |
| A_43_P10227 | 0.007315 | 0.86589 | Abi1 | Rn.43675 |
| A_43_P10371 | 0.006663 | 1.296173 | RGD1311422 | Rn.113761 |
| A_43_P10382 | 0.034293 | 1.418634 | Slit1 | Rn.30002 |
| A_43_P10481 | 0.028985 | 1.276412 |  |  |
| A_43_P10548 | 0.048393 | 1.890603 |  | Rn.12112 |
| A_43_P10641 | 0.006631 | 1.291301 | Mgst2 | Rn.7854 |
| A_43_P10642 | 0.033346 | 1.203993 | Poldip3 | Rn.3893 |
| A_43_P10807 | 0.007437 | 1.269784 |  |  |
| A_43_P10887 | 0.041868 | 0.732709 | Map1b | Rn.98152 |
| A_43_P10888 | 0.032899 | 1.182932 | Sort1 | Rn.11286 |
| A_43_P11005 | 0.025346 | 1.255205 | Ppp1r13b | Rn.30183 |
| A_43_P11369 | 0.038329 | 1.156737 | RGD1304792 | Rn.38790 |
| A_43_P11495 | 0.049468 | 1.16324 | Prps2 | Rn.11320 |
| A_43_P11616 | 0.018719 | 0.51041 | Atf3 | Rn.9664 |
| A_43_P11621 | 0.032875 | 0.818175 | Cd44 | Rn.1120 |
| A_43_P11897 | 0.013358 | 1.515225 | Gabrd | Rn.10927 |
| A_43_P11984 | 0.025101 | 1.12232 | Itsn1 | Rn.48657 |
| A_43_P12059 | 0.011367 | 1.335752 | Rnase4 | Rn.1742 |
| A_43_P12304 | 0.047076 | 1.24503 | Sgta | Rn.9655 |
| A_43_P12404 | 0.033298 | 0.738057 | Evl | Rn.28912 |
| A_43_P12607 | 0.012268 | 1.89474 | Ptprn2 | Rn.11044 |
| A_43_P12794 | 0.044072 | 1.095045 | Ngfrap1 | Rn.3126 |
| A_43_P13057 | 0.0425 | 1.135576 | Baiap2 | Rn.95155 |
| A_43_P13074 | 0.041292 | 1.255443 | Rab3d | Rn.9822 |
| A_43_P13220 | 0.0455 | 1.300774 | Blcap | Rn.127812 |
| A_43_P13244 | 0.023122 | 0.789626 | Rap1b | Rn.95071 |
| A_43_P13270 | 0.043557 | 1.165505 | Cdk105 | Rn.15195 |
| A_43_P13393 | 0.027644 | 1.483876 | Gpsm1 | Rn.53933 |
| A_43_P13410 | 0.021235 | 1.391153 | Serpina4 | Rn.11152 |
| A_43_P13711 | 0.01819 | 0.824387 | Tbc1d22b | Rn.16811 |
| A_43_P13744 | 0.006576 | 0.594197 |  |  |
| A_43_P13828 | 0.010705 | 1.220379 |  | Rn.23354 |
| A_43_P14080 | 0.03191 | 1.125385 |  |  |
| A_43_P14581 | 0.01094 | 0.828844 | Cbll1 | Rn.92651 |
| A_43_P14615 | 0.044354 | 1.233911 |  | Rn.15594 |
| A_43_P14782 | 0.023061 | 1.145076 | Tnk2 | Rn.98335 |
| A_43_P14884 | 0.037164 | 1.069066 | Akap8 | Rn.10248 |
| A_43_P15387 | 0.002393 | 1.616492 | Guca2b | Rn.97593 |
| A_43_P15436 | 0.036005 | 1.112511 | Ep300 | Rn.12447 |
| A_43_P15475 | 0.00389 | 1.7806 | Tpc1808 | Rn.127789 |
| A_43_P15517 | 0.004536 | 0.50121 | Jund | Rn.46225 |
| A_43_P15523 | 0.017902 | 2.296674 | Prp15 | Rn.9841 |
| A_43_P15524 | 0.026862 | 0.89463 | Ccnd3 | Rn.3483 |
| A_43_P15633 | 0.022444 | 1.262552 | Pkn2 | Rn.30325 |
| A_43_P15865 | 0.013492 | 1.35587 | Slc24a3 | Rn.206123 |
| A_43_P15910 | 0.004304 | 1.604323 | LOC100363412 | Rn.137893 |
| A_43_P16070 | 0.007209 | 1.264503 | Axl | Rn.161805 |
| A_43_P16279 | 0.030742 | 1.552324 | Col9a1 | Rn.90726 |
| A_43_P16353 | 0.009174 | 1.853726 |  | Rn.214232 |
| A_43_P16537 | 0.001292 | 1.351826 | Pcnp | Rn.202642 |
| A_43_P16688 | 0.008647 | 1.394582 | Katnb1 | Rn.22157 |
| A_43_P16725 | 0.045914 | 1.167589 | RGD1311805 | Rn.35239 |
| A_43_P16755 | 0.043024 | 1.172329 | Alg5 | Rn.155539 |
| A_43_P16887 | 0.037281 | 1.842832 | Ahsp | Rn.136569 |
| A_43_P16926 | 0.002929 | 1.182197 | Appl1 | Rn.224576 |
| A_43_P17007 | 0.029775 | 1.438312 | Fbxo31 | Rn.163412 |
| A_43_P17008 | 0.018756 | 1.422374 | Bpgm | Rn.204528 |
| A_43_P17065 | 0.045484 | 0.713835 | Helz | Rn.19942 |
| A_43_P17238 | 0.047255 | 1.244874 | Rnd2 | Rn.22162 |
| A_43_P17264 | 0.043822 | 1.188043 | Pbrm1 | Rn.19314 |
| A_43_P17299 | 0.004338 | 1.523909 |  | Rn.205433 |
| A_43_P17326 | 0.014067 | 0.669422 | Mov10 | Rn.3508 |
| A_43_P17460 | 0.026921 | 1.298503 | Limd1 | Rn.3966 |
| A_43_P17682 | 0.021571 | 1.192109 |  | Rn.104463 |
| A_43_P17808 | 0.000753 | 1.520103 | RGD1307603 | Rn.16668 |
| A_43_P17979 | 0.023543 | 1.185157 | LOC100363361 | Rn.139466 |
| A_43_P18039 | 0.022152 | 0.813685 | Tab2 | Rn.228335 |
| A_43_P18163 | 0.002366 | 1.53283 | Tmem132e | Rn.46621 |
| A_43_P18263 | 0.011203 | 1.19957 | Celf1 | Rn.22432 |
| A_43_P18422 | 0.011724 | 1.826668 | Zcchc24 | Rn.44060 |
| A_43_P18469 | 0.025522 | 1.33389 | Sytl3 | Rn.136598 |
| A_43_P18596 | 0.034341 | 1.088309 | Slu7 | Rn.137183 |
| A_43_P18684 | 0.025936 | 1.221524 | Ric8a | Rn.94796 |
| A_43_P18798 | 0.011301 | 1.129164 | Fbxw8 | Rn.812 |
| A_43_P18970 | 0.03148 | 1.327288 | Abcd1 | Rn.163453 |
| A_43_P19050 | 0.001792 | 1.361024 | RGD1307597 | Rn.93564 |
| A_43_P19065 | 0.019722 | 1.365354 | Pcnxl3 | Rn.19007 |
| A_43_P19169 | 0.025727 | 1.29144 | Dcbld1 | Rn.105687 |
| A_43_P19210 | 0.020156 | 1.320793 | Taf1c | Rn.146984 |
| A_43_P19268 | 0.017049 | 1.486706 | Gsx2 | Rn.102073 |
| A_43_P19484 | 0.005353 | 1.609567 | Doxl1 | Rn.79162 |
| A_43_P19494 | 0.035678 | 1.845918 | LOC688972 | Rn.6717 |
| A_43_P19501 | 0.040657 | 1.216309 | Dnajc18 | Rn.38207 |
| A_43_P19529 | 0.033717 | 1.11516 | Thrap3 | Rn.228247 |
| A_43_P19560 | 0.032024 | 1.185593 | Brf1 | Rn.205393 |
| A_43_P19585 | 0.021219 | 1.401599 | Neurl4 | Rn.100409 |
| A_43_P19603 | 0.048885 | 1.183751 | Zc3h7b | Rn.91605 |
| A_43_P19633 | 0.005873 | 1.288075 | Nkx2-2 | Rn.32651 |
| A_43_P19663 | 0.038018 | 0.852412 | Pax2 | Rn.137112 |
| A_43_P19762 | 0.020878 | 1.372786 |  | Rn.9000 |
| A_43_P19776 | 0.01955 | 0.717716 | LOC681325 | Rn.48312 |
| A_43_P19852 | 0.00979 | 1.565164 | Fam71a | Rn.59403 |
| A_43_P19892 | 0.03323 | 1.142177 | Aph1a | Rn.22786 |
| A_43_P20044 | 0.045954 | 1.158399 |  | Rn.20310 |
| A_43_P20207 | 0.012526 | 1.38367 | Klhl21 | Rn.87563 |
| A_43_P20397 | 0.015995 | 0.858283 |  | Rn.20718 |
| A_43_P20412 | 0.025763 | 1.314484 | Arsg | Rn.221856 |
| A_43_P20534 | 0.043666 | 1.145896 | Fbxo22 | Rn.4284 |
| A_43_P20631 | 0.034426 | 1.201715 | Rilpl1 | Rn.62627 |
| A_43_P20745 | 0.015147 | 1.654022 | Siae | Rn.21775 |
| A_43_P20800 | 0.040167 | 1.248434 | Amigo1 | Rn.116338 |
| A_43_P20922 | 0.010133 | 1.308586 | Tbc1d9 | Rn.19710 |
| A_43_P21504 | 0.029965 | 1.495164 | Kcnt2 | Rn.212417 |
| A_43_P21507 | 0.005916 | 1.182095 | Herc1 | Rn.77165 |
| A_43_P21626 | 0.003271 | 1.573742 | Plcd3 | Rn.95716 |
| A_43_P21628 | 0.047639 | 1.123884 | Ddx23 | Rn.2319 |
| A_43_P21753 | 0.010506 | 1.282728 | Adam19 | Rn.113277 |
| A_43_P21830 | 0.000382 | 1.830944 |  | Rn.202171 |
| A_43_P22037 | 0.013712 | 0.792384 | Sall3 | Rn.99341 |
| A_43_P22157 | 0.04552 | 1.350044 | Pcdhga2 | Rn.8628 |
| A_43_P22210 | 0.032359 | 1.158911 | Zfp644 | Rn.8773 |
| A_43_P22276 | 0.00966 | 1.291207 | LOC100362283 | Rn.158990 |
| A_43_P22349 | 0.007048 | 1.827538 |  | Rn.219733 |
| A_43_P22661 | 0.009104 | 1.357378 | LOC499602 | Rn.12999 |
| A_43_P22719 | 0.0129 | 1.436763 | Pla2g2e | Rn.86225 |
| A_43_P23049 | 0.044013 | 1.323929 | Cryz | Rn.6746 |
| A_43_P23126 | 0.043619 | 1.224759 | Klf11 | Rn.18589 |
| A_43_P23459 | 0.003253 | 0.8054 |  |  |
| A_43_P23467 | 0.016818 | 1.223848 | B3galnt2 | Rn.140763 |
| A_44_P1000383 | 0.018574 | 0.617367 | Eprs | Rn.21240 |
| A_44_P1003794 | 0.022207 | 0.66141 | Nr3c1 | Rn.90070 |
| A_44_P100451 | 0.048031 | 1.254974 | Kdsr | Rn.22924 |
| A_44_P1006988 | 0.045699 | 1.384993 | Zfp318 | Rn.28520 |
| A_44_P1008094 | 0.037089 | 0.698264 | Exosc7 | Rn.45886 |
| A_44_P100996 | 0.042962 | 1.126439 | Rpl29 | Rn.3973 |
| A_44_P1010500 | 0.03696 | 1.096909 | Ctsa | Rn.104631 |
| A_44_P1011253 | 0.028493 | 0.711608 | Nars | Rn.48209 |
| A_44_P1011538 | 0.03026 | 1.149169 | Kin | Rn.53701 |
| A_44_P1012591 | 0.003135 | 0.77895 | Osbpl1a | Rn.178664 |
| A_44_P1012870 | 0.015019 | 1.101963 | RGD1309095 | Rn.40282 |
| A_44_P1013851 | 0.048168 | 0.755838 | Bmp2 | Rn.90931 |
| A_44_P1014005 | 0.027826 | 0.830259 | Cdc42se1 | Rn.111901 |
| A_44_P1014955 | 0.017072 | 0.794123 | Psph | Rn.8734 |
| A_44_P1015355 | 0.032144 | 0.540898 | Rnmt | Rn.20871 |
| A_44_P1015606 | 0.02368 | 0.658652 | Sfswap | Rn.12338 |
| A_44_P101591 | 0.019048 | 0.762784 |  | Rn.106939 |
| A_44_P1017125 | 0.036633 | 0.720636 | Sav1 | Rn.14087 |
| A_44_P1017458 | 0.02666 | 1.453259 | Spata20 | Rn.50006 |
| A_44_P1017820 | 0.036425 | 0.789051 | Leo1 | Rn.43195 |
| A_44_P1018957 | 0.044016 | 1.192787 | Hsd11b1 | Rn.888 |
| A_44_P1020708 | 0.034502 | 1.159727 | Rpl26 | Rn.144702 |
| A_44_P1024567 | 0.031584 | 1.308796 | Mxd4 | Rn.3279 |
| A_44_P1025750 | 0.023251 | 0.417033 | Rchy1 | Rn.2007 |
| A_44_P1026252 | 0.036293 | 0.847716 | Gyltl1b | Rn.205174 |
| A_44_P1026560 | 0.030522 | 0.862479 |  |  |
| A_44_P1027005 | 0.013834 | 0.732264 |  | Rn.2892 |
| A_44_P102845 | 0.026271 | 1.136844 | Ube2v1 | Rn.220168 |
| A_44_P1029241 | 0.048542 | 1.447846 | Car1 | Rn.6854 |
| A_44_P1030594 | 0.014785 | 0.797921 | Cebpg | Rn.10332 |
| A_44_P1030727 | 0.021724 | 0.777368 |  | Rn.3390 |
| A_44_P1031514 | 0.02438 | 1.129634 | Tmem168 | Rn.7056 |
| A_44_P1031524 | 0.008908 | 0.710378 | Rpl34 | Rn.2028 |
| A_44_P1032051 | 0.01312 | 1.105374 | LOC687565 | Rn.101864 |
| A_44_P1032538 | 0.007497 | 1.336002 | Gng3 | Rn.198523 |
| A_44_P1034209 | 0.039742 | 1.21704 | Lamb3 | Rn.49634 |
| A_44_P103472 | 0.039847 | 1.160081 | LOC503192 | Rn.69744 |
| A_44_P1035071 | 0.049019 | 0.809027 | Nmt2 | Rn.162255 |
| A_44_P1037275 | 0.037879 | 0.679084 | Vkorc1l1 | Rn.163208 |
| A_44_P1037886 | 0.006939 | 1.134122 | Sap18 | Rn.74373 |
| A_44_P1039205 | 0.029882 | 1.378917 | Cpa2 | Rn.101694 |
| A_44_P1039213 | 0.002488 | 1.571147 | Cpa2 | Rn.101694 |
| A_44_P1042473 | 0.043958 | 0.828955 | Lgals3bp | Rn.3251 |
| A_44_P104250 | 0.012059 | 0.753574 | Mrps14 | Rn.67 |
| A_44_P1042901 | 0.028402 | 0.92973 | Pum2 | Rn.8622 |
| A_44_P1042940 | 0.004394 | 0.677078 |  |  |
| A_44_P104483 | 0.034474 | 0.582092 | Ccdc64 | Rn.166161 |
| A_44_P1044830 | 0.028432 | 0.553067 | Lactb | Rn.73451 |
| A_44_P1045469 | 0.04239 | 0.735352 |  | Rn.199571 |
| A_44_P1045759 | 0.043191 | 0.907867 | RGD1309104 | Rn.228438 |
| A_44_P1046471 | 0.036433 | 0.859348 | Rpap3 | Rn.49051 |
| A_44_P1047315 | 0.014714 | 1.495476 | Lynx1 | Rn.32045 |
| A_44_P1048611 | 0.03516 | 0.813089 | Llph | Rn.205328 |
| A_44_P1049827 | 0.032971 | 1.582889 | Nsg1 | Rn.2865 |
| A_44_P1052324 | 0.046068 | 0.479049 | Lamp3 | Rn.19784 |
| A_44_P1052504 | 0.010187 | 0.512079 | Rhot2 | Rn.8882 |
| A_44_P1052868 | 0.049049 | 0.63645 | Syne1 | Rn.226002 |
| A_44_P1053645 | 0.049122 | 1.112397 |  | Rn.16562 |
| A_44_P1056429 | 0.025798 | 0.836848 | Trak1 | Rn.3090 |
| A_44_P1057137 | 0.009654 | 0.756082 | Phf1 | Rn.24149 |
| A_44_P1057331 | 0.023635 | 0.761725 | Stk40 | Rn.4052 |
| A_44_P1057602 | 0.021414 | 0.833397 | Ercc6 | Rn.19370 |
| A_44_P1059322 | 0.027909 | 0.291907 | Whamm | Rn.198506 |
| A_44_P1060108 | 0.020122 | 0.70535 | Tmsb10 | Rn.5983 |
| A_44_P1060345 | 0.012641 | 0.712405 | Cpox | Rn.19581 |
| A_44_P106248 | 0.026383 | 0.648375 |  |  |
| A_44_P106541 | 0.011205 | 1.199383 | Alg5 | Rn.155539 |
| A_44_P1070896 | 0.010772 | 1.355041 | Dmd |  |
| A_44_P107097 | 0.010782 | 1.568516 | Dcps | Rn.162991 |
| A_44_P1070980 | 0.028835 | 0.663796 |  | Rn.25307 |
| A_44_P1071170 | 0.033121 | 1.448624 |  | Rn.44300 |
| A_44_P107495 | 0.039335 | 1.24553 | Epb4.1l2 | Rn.1362 |
| A_44_P107766 | 0.024445 | 1.188855 | RGD1309540 | Rn.154938 |
| A_44_P107801 | 0.048893 | 1.164294 | Ppp1r16b | Rn.155635 |
| A_44_P108086 | 0.024453 | 0.862872 | Rras2 | Rn.3271 |
| A_44_P108102 | 0.013846 | 0.778029 |  |  |
| A_44_P108296 | 0.026021 | 0.780772 |  | Rn.219993 |
| A_44_P108430 | 0.048726 | 0.711208 |  | Rn.7774 |
| A_44_P110110 | 0.024943 | 1.142816 | Cst3 | Rn.106351 |
| A_44_P111347 | 0.007535 | 0.683671 |  | Rn.72721 |
| A_44_P112311 | 0.037889 | 1.183686 | Tmem127 | Rn.19881 |
| A_44_P113084 | 0.007881 | 0.662019 |  | Rn.98343 |
| A_44_P113360 | 0.020593 | 0.513997 |  | Rn.112601 |
| A_44_P113879 | 0.043772 | 2.139475 | Slco1a5 | Rn.9912 |
| A_44_P114500 | 0.013598 | 0.391211 | Gipc2 | Rn.176667 |
| A_44_P114606 | 0.04569 | 0.557819 |  | Rn.124755 |
| A_44_P115842 | 0.04281 | 1.410268 |  | Rn.198407 |
| A_44_P116532 | 0.042031 | 1.398686 | Cdh22 | Rn.108785 |
| A_44_P116758 | 0.037401 | 1.228607 | Paip2 | Rn.1073 |
| A_44_P117107 | 0.013241 | 1.128553 | Rpsa | Rn.161973 |
| A_44_P117245 | 0.048271 | 1.079732 |  |  |
| A_44_P117850 | 0.045444 | 0.77433 |  |  |
| A_44_P118540 | 0.013624 | 0.735429 | Erbb2 | Rn.93966 |
| A_44_P118734 | 0.009741 | 1.773367 | Olr95 | Rn.141892 |
| A_44_P118914 | 0.048778 | 2.790006 |  | Rn.112186 |
| A_44_P119577 | 0.019923 | 0.924372 | RGD1565403 | |
| A_44_P121003 | 0.008559 | 1.368021 | Aspa | Rn.21677 |
| A_44_P121728 | 0.020951 | 1.179213 | Il17rc | Rn.139872 |
| A_44_P121887 | 0.037148 | 1.175971 | Sidt2 | Rn.162997 |
| A_44_P122333 | 0.01106 | 1.435724 |  | Rn.3043 |
| A_44_P124013 | 0.010478 | 1.326011 | Tcf15 | Rn.222438 |
| A_44_P124091 | 0.018662 | 1.135804 |  |  |
| A_44_P127676 | 0.016598 | 0.90945 |  | Rn.22432 |
| A_44_P128583 | 0.011264 | 0.824648 | Ranbp3 | Rn.101054 |
| A_44_P129029 | 0.019507 | 1.866674 | Hoxa9 | Rn.203714 |
| A_44_P130780 | 0.009925 | 0.907888 | Rpl13 | Rn.908 |
| A_44_P131084 | 0.009791 | 0.850137 | Rbmx2 | Rn.67383 |
| A_44_P131870 | 0.04519 | 0.747345 |  | Rn.219352 |
| A_44_P132333 | 0.036469 | 0.646012 |  | Rn.203111 |
| A_44_P134739 | 0.035186 | 1.148382 | Csde1 | Rn.3562 |
| A_44_P136141 | 0.02628 | 0.838783 |  | Rn.160589 |
| A_44_P137262 | 0.003634 | 1.225013 | Nmt1 | Rn.830 |
| A_44_P137273 | 0.019068 | 1.664684 | Fbxl7 | Rn.45065 |
| A_44_P138057 | 0.0496 | 1.09285 | Rpl13 | Rn.908 |
| A_44_P138081 | 0.037799 | 0.808028 | Med14 | Rn.222594 |
| A_44_P138689 | 0.008104 | 0.527429 |  | Rn.37338 |
| A_44_P139494 | 0.009499 | 1.626573 | Mef2bnb | Rn.27139 |
| A_44_P139500 | 0.021602 | 1.939389 |  | Rn.27139 |
| A_44_P139694 | 0.04427 | 0.825552 | Tnfaip1 | Rn.18199 |
| A_44_P139956 | 0.010923 | 1.342215 | Hbb-b1 | Rn.36966 |
| A_44_P140214 | 0.015688 | 0.713032 | RGD1560069 | |
| A_44_P140805 | 0.010275 | 1.192928 | Top2b | Rn.104585 |
| A_44_P141091 | 0.002552 | 1.22468 |  | Rn.203964 |
| A_44_P141288 | 0.033517 | 1.135956 | Rab34 | Rn.98493 |
| A_44_P141897 | 0.017266 | 1.67713 | Abcb1b | Rn.144554 |
| A_44_P144796 | 0.048734 | 0.404037 |  |  |
| A_44_P148280 | 0.003607 | 1.15563 | Crcp | Rn.9449 |
| A_44_P148695 | 0.037293 | 0.87229 | Ammecr1l | Rn.64258 |
| A_44_P149540 | 0.029684 | 1.141541 |  |  |
| A_44_P151675 | 0.014719 | 1.220659 | Gfra3 | Rn.15499 |
| A_44_P151920 | 0.027828 | 1.197866 | Fam53b | Rn.12282 |
| A_44_P153753 | 0.048628 | 1.276426 | Ttl | Rn.205120 |
| A_44_P156705 | 0.034497 | 0.463011 |  |  |
| A_44_P158777 | 0.018927 | 0.768518 | Prkacb | Rn.202491 |
| A_44_P158783 | 0.026837 | 1.337626 | Aoc3 | Rn.198327 |
| A_44_P159341 | 0.031395 | 1.323679 | Tbc1d16 |  |
| A_44_P159400 | 0.046409 | 0.711091 |  |  |
| A_44_P160545 | 0.02494 | 0.767524 |  | Rn.203252 |
| A_44_P160877 | 0.035561 | 1.336226 | Cfh | Rn.101777 |
| A_44_P161674 | 0.047979 | 0.338696 |  |  |
| A_44_P165612 | 0.017475 | 1.439995 | Olr1415 | Rn.142152 |
| A_44_P166186 | 0.007416 | 1.239838 | Tor1b | Rn.6487 |
| A_44_P166267 | 0.00262 | 1.334401 | Abcb10 | Rn.22937 |
| A_44_P168103 | 0.035001 | 0.926046 | Skp1 | Rn.3477 |
| A_44_P169662 | 0.032747 | 0.824218 | Faf1 | Rn.198288 |
| A_44_P170971 | 0.033923 | 0.705744 |  | Rn.124815 |
| A_44_P171109 | 0.039108 | 0.615029 |  |  |
| A_44_P171143 | 0.035687 | 0.616281 |  |  |
| A_44_P173511 | 0.002767 | 1.11541 |  | Rn.148155 |
| A_44_P174032 | 0.028445 | 1.23616 |  | Rn.174745 |
| A_44_P174682 | 0.020286 | 0.781761 | Nfyc | Rn.1457 |
| A_44_P174970 | 0.045072 | 1.225769 |  | Rn.22288 |
| A_44_P175234 | 0.044744 | 1.241229 | Rgs6 | Rn.207298 |
| A_44_P175376 | 0.029676 | 1.512188 | Aldh1b1 | Rn.12547 |
| A_44_P175734 | 0.015345 | 1.565529 | Fam65c | Rn.104575 |
| A_44_P175785 | 0.043702 | 1.211191 | Slc35a2 | Rn.2866 |
| A_44_P176063 | 0.031743 | 0.51851 |  |  |
| A_44_P177736 | 0.035119 | 1.185691 |  | Rn.89150 |
| A_44_P180080 | 0.041041 | 1.193381 | Map1lc3b | Rn.41412 |
| A_44_P180519 | 0.01767 | 1.162051 | Mon1b | Rn.225132 |
| A_44_P182568 | 0.014015 | 1.301564 | Fntb | Rn.8873 |
| A_44_P182721 | 0.00488 | 0.616118 |  |  |
| A_44_P183007 | 0.038633 | 0.464893 |  |  |
| A_44_P183087 | 0.034394 | 1.125483 | Moap1 | Rn.98234 |
| A_44_P183142 | 0.017648 | 0.82391 |  | Rn.119950 |
| A_44_P185138 | 0.028899 | 0.825792 |  |  |
| A_44_P185337 | 0.045637 | 1.066754 | RGD1560187 | |
| A_44_P185629 | 0.026871 | 0.679847 |  | Rn.128129 |
| A_44_P185673 | 0.001758 | 0.847113 |  | Rn.80611 |
| A_44_P187249 | 0.013054 | 1.226573 |  |  |
| A_44_P191862 | 0.046816 | 1.107147 | Hras | Rn.102180 |
| A_44_P193593 | 0.001622 | 0.425759 |  | Rn.219950 |
| A_44_P194439 | 0.041349 | 1.233633 | Dnajc9 | Rn.16186 |
| A_44_P195468 | 0.040299 | 0.762032 | Ythdc1 | Rn.48752 |
| A_44_P195499 | 0.01837 | 0.715059 |  | Rn.166161 |
| A_44_P196532 | 0.005409 | 0.496427 | Mk1 | Rn.201717 |
| A_44_P196805 | 0.003294 | 1.210002 | Golga7 | Rn.4105 |
| A_44_P197981 | 0.035005 | 0.9117 |  | Rn.11790 |
| A_44_P199077 | 0.049092 | 1.112934 | Ubac2 | Rn.2539 |
| A_44_P199161 | 0.005606 | 1.112154 | Rps16 | Rn.29791 |
| A_44_P199624 | 0.039109 | 1.14786 | Bad | Rn.36696 |
| A_44_P200146 | 0.016415 | 1.26735 | Oxr1 | Rn.4252 |
| A_44_P200617 | 0.029752 | 1.356729 | Ube2o | Rn.137727 |
| A_44_P201077 | 0.007161 | 1.335729 | Asrgl1 | Rn.22774 |
| A_44_P201389 | 0.016462 | 1.123066 | Psmc6 | Rn.103233 |
| A_44_P203493 | 0.02121 | 1.120862 | Tceb1 | Rn.5996 |
| A_44_P205928 | 0.019639 | 1.260497 | Zkscan5 | Rn.38399 |
| A_44_P206434 | 0.003222 | 0.748685 | Lmnb2 | Rn.12408 |
| A_44_P206466 | 0.000517 | 1.149037 | Senp6 | Rn.98553 |
| A_44_P208415 | 0.006478 | 1.284063 |  | Rn.3613 |
| A_44_P211028 | 0.019163 | 0.456886 |  |  |
| A_44_P212294 | 0.020568 | 1.188582 |  | Rn.148299 |
| A_44_P212552 | 0.012795 | 0.341258 | Tob1 | Rn.228429 |
| A_44_P212682 | 0.033175 | 0.904149 | Polr2l | Rn.30672 |
| A_44_P214797 | 0.049816 | 0.816043 |  | Rn.178356 |
| A_44_P215698 | 0.009604 | 1.117916 |  |  |
| A_44_P215731 | 0.020868 | 0.758115 |  | Rn.212628 |
| A_44_P217383 | 0.037726 | 1.490037 | Olr1335 | Rn.143466 |
| A_44_P218263 | 0.036998 | 0.790429 | Sned1 | Rn.23447 |
| A_44_P218695 | 0.020539 | 0.766215 |  | Rn.4165 |
| A_44_P219055 | 0.019782 | 1.660967 |  | Rn.11046 |
| A_44_P219454 | 0.023979 | 1.229998 |  | Rn.10990 |
| A_44_P220394 | 0.020673 | 1.101028 | RGD1560099 | |
| A_44_P220661 | 0.033438 | 1.16747 |  |  |
| A_44_P221213 | 0.019633 | 0.901813 |  | Rn.1441 |
| A_44_P222367 | 0.047743 | 1.21476 |  |  |
| A_44_P223607 | 0.018168 | 1.280866 |  | Rn.175195 |
| A_44_P223741 | 0.01495 | 0.798079 |  | Rn.116073 |
| A_44_P224466 | 0.030417 | 1.479568 | Olr567 | Rn.142899 |
| A_44_P224900 | 0.009439 | 1.239146 | Tspan14 | Rn.224880 |
| A_44_P226029 | 0.043099 | 0.837601 | Rbmxrtl | Rn.19966 |
| A_44_P227251 | 0.017237 | 1.089029 | Fam13b | Rn.25218 |
| A_44_P227421 | 0.044256 | 0.513132 |  |  |
| A_44_P229540 | 0.02232 | 1.177592 | Epc2 | Rn.23462 |
| A_44_P229948 | 0.012473 | 1.602193 |  | Rn.104800 |
| A_44_P230030 | 0.011063 | 1.116965 | Snap29 | Rn.17902 |
| A_44_P231066 | 0.047531 | 0.932509 | Uba1 | Rn.11800 |
| A_44_P231737 | 0.010275 | 0.564822 | RGD1562755 | |
| A_44_P232113 | 0.03452 | 0.444548 |  |  |
| A_44_P232255 | 0.036621 | 1.602181 | Alppl2 | Rn.129988 |
| A_44_P232285 | 0.028063 | 0.363327 |  |  |
| A_44_P233231 | 0.012408 | 1.112296 |  | Rn.216496 |
| A_44_P234315 | 0.013063 | 1.798366 |  | Rn.154683 |
| A_44_P234460 | 0.016971 | 0.783 |  |  |
| A_44_P235815 | 0.013937 | 0.816987 | Clns1a | Rn.4089 |
| A_44_P235848 | 0.037099 | 0.664358 | Bccip | Rn.154691 |
| A_44_P236271 | 0.01936 | 2.287216 | Hoxc8 | Rn.91646 |
| A_44_P236636 | 0.021477 | 0.657843 |  |  |
| A_44_P237621 | 0.018961 | 0.587964 | Txnip | Rn.2758 |
| A_44_P238733 | 0.03159 | 0.312228 | Tmed5 | Rn.204558 |
| A_44_P239650 | 0.042268 | 0.810132 |  |  |
| A_44_P241230 | 0.029753 | 0.55335 |  |  |
| A_44_P242588 | 0.035683 | 0.785181 |  | Rn.26720 |
| A_44_P243153 | 0.022562 | 1.181369 | Ncoa6 | Rn.9077 |
| A_44_P243433 | 0.003598 | 1.578695 |  |  |
| A_44_P243564 | 0.046745 | 0.879195 |  |  |
| A_44_P243718 | 0.008684 | 1.276206 |  |  |
| A_44_P244063 | 0.024879 | 1.498187 | LOC363458 | Rn.182586 |
| A_44_P246146 | 0.032938 | 1.193268 | Rbmx | Rn.40449 |
| A_44_P246381 | 0.032693 | 1.178833 | Dgcr6 | Rn.22467 |
| A_44_P248291 | 0.03119 | 0.84102 | Prmt6 | Rn.18530 |
| A_44_P249578 | 0.045164 | 1.255623 |  | Rn.112761 |
| A_44_P250460 | 0.036671 | 1.259935 | Ercc2 | Rn.41871 |
| A_44_P251875 | 0.049379 | 1.824501 |  | Rn.43529 |
| A_44_P251897 | 0.008288 | 0.807905 |  | Rn.224540 |
| A_44_P251908 | 0.021354 | 0.806355 | Xpnpep1 | Rn.25763 |
| A_44_P252861 | 0.022375 | 1.204122 |  | Rn.5977 |
| A_44_P254413 | 0.048987 | 1.069836 | Hnrnpu | Rn.4328 |
| A_44_P254896 | 0.006597 | 0.722594 | Ddb1 |  |
| A_44_P255359 | 0.041074 | 0.812902 | Ndufs2 | Rn.225926 |
| A_44_P256345 | 0.040398 | 0.803743 | Smarcad1 | Rn.7758 |
| A_44_P257563 | 0.023024 | 1.338835 | Cdk18 | Rn.219420 |
| A_44_P258203 | 0.027702 | 1.101171 |  |  |
| A_44_P260787 | 0.040807 | 0.723044 |  | Rn.23125 |
| A_44_P261259 | 0.047873 | 0.75074 |  |  |
| A_44_P261726 | 0.026414 | 1.297313 |  | Rn.35304 |
| A_44_P262625 | 0.047569 | 0.697436 |  |  |
| A_44_P264880 | 0.007821 | 0.480053 | LOC100363884 | |
| A_44_P264965 | 0.019239 | 1.601606 | RGD1564961 | |
| A_44_P265127 | 0.015467 | 1.597869 | Svs3b | Rn.100666 |
| A_44_P265416 | 0.00244 | 0.786208 | Aqp12a | Rn.20532 |
| A_44_P269499 | 0.043505 | 0.6074 |  | Rn.154560 |
| A_44_P269582 | 0.001343 | 0.783085 | Dusp8 | Rn.219421 |
| A_44_P269752 | 0.012753 | 1.189403 |  |  |
| A_44_P270096 | 0.035889 | 1.106642 | Smg6 | Rn.1945 |
| A_44_P270395 | 0.033225 | 0.841428 |  | Rn.56373 |
| A_44_P270669 | 0.007211 | 0.818615 |  | Rn.203335 |
| A_44_P271039 | 0.021142 | 0.204388 |  | Rn.215881 |
| A_44_P271229 | 0.003546 | 1.484542 |  | Rn.153937 |
| A_44_P274605 | 0.00853 | 1.234334 | RGD1562165 | |
| A_44_P275523 | 0.043019 | 1.107856 |  | Rn.7319 |
| A_44_P277423 | 0.007079 | 0.602459 |  | Rn.90166 |
| A_44_P278140 | 0.026106 | 1.122645 |  | Rn.8634 |
| A_44_P278938 | 0.016021 | 1.279137 | Elf2 | Rn.107459 |
| A_44_P279240 | 0.038916 | 1.149729 | Rpl29 | Rn.3973 |
| A_44_P280005 | 0.000255 | 0.680306 |  | Rn.8153 |
| A_44_P281393 | 0.024712 | 0.69863 |  |  |
| A_44_P282415 | 0.022612 | 0.690601 |  | Rn.190571 |
| A_44_P283561 | 0.034885 | 1.115477 | Mgst1 | Rn.2580 |
| A_44_P283646 | 0.046045 | 1.393968 | Plekhf1 | Rn.9380 |
| A_44_P284169 | 0.023354 | 0.726954 |  |  |
| A_44_P286104 | 0.016114 | 1.289503 | Pygo2 | Rn.24988 |
| A_44_P286158 | 0.038908 | 0.668978 |  |  |
| A_44_P286439 | 0.038632 | 1.302361 | Trim42 | Rn.109830 |
| A_44_P286467 | 0.029135 | 1.201881 | Tmem131 | Rn.63731 |
| A_44_P286816 | 0.026613 | 1.634366 |  | Rn.15665 |
| A_44_P288377 | 0.03316 | 1.870309 |  |  |
| A_44_P289637 | 0.03143 | 2.0744 | Slc4a1 | Rn.32202 |
| A_44_P290867 | 0.043818 | 0.850952 |  |  |
| A_44_P294637 | 0.022845 | 1.480306 | Nlgn3 | Rn.226139 |
| A_44_P295125 | 0.002807 | 0.658866 |  |  |
| A_44_P295385 | 0.028826 | 1.178322 |  |  |
| A_44_P299214 | 0.019096 | 0.793581 |  |  |
| A_44_P301799 | 0.046735 | 1.221355 | Ryk | Rn.11796 |
| A_44_P301805 | 0.040681 | 1.145757 | Ryk | Rn.11796 |
| A_44_P302519 | 0.007489 | 1.230099 |  |  |
| A_44_P302613 | 0.026057 | 1.175926 | Lkap | Rn.27366 |
| A_44_P302676 | 0.0331 | 0.601885 |  |  |
| A_44_P302972 | 0.009508 | 0.71065 |  |  |
| A_44_P304043 | 0.03237 | 1.189341 | Dyt1 | Rn.20041 |
| A_44_P304408 | 0.022232 | 1.124212 | Dctn3 | Rn.2038 |
| A_44_P305689 | 0.036416 | 0.703063 | Lap3 | Rn.99790 |
| A_44_P305961 | 0.025242 | 1.135438 |  | Rn.3271 |
| A_44_P306307 | 0.043787 | 1.351324 | Hbb | Rn.202945 |
| A_44_P307308 | 0.040182 | 0.614192 | LOC301725 | |
| A_44_P307971 | 0.038785 | 0.776197 |  |  |
| A_44_P308877 | 0.039857 | 1.168727 | Dmd | Rn.10307 |
| A_44_P309325 | 0.00885 | 1.283908 | Lig4 | Rn.219326 |
| A_44_P310851 | 0.036438 | 0.871405 | Psmf1 | Rn.103325 |
| A_44_P313022 | 0.008539 | 0.769868 |  | Rn.98819 |
| A_44_P313077 | 0.04919 | 1.175066 | Usp46 | Rn.228328 |
| A_44_P313431 | 0.018168 | 1.090733 | Thoc2 | Rn.17823 |
| A_44_P314152 | 0.034842 | 0.80635 |  |  |
| A_44_P314386 | 0.016244 | 1.202251 | Vps13b | Rn.7442 |
| A_44_P315895 | 0.016821 | 1.31517 | Slc14a2 | Rn.10157 |
| A_44_P316286 | 0.039811 | 1.323174 | Sap25 | Rn.59091 |
| A_44_P318103 | 0.008323 | 0.825965 | Atp9b | Rn.40336 |
| A_44_P319208 | 0.02992 | 1.18427 | Dpp8 | Rn.17532 |
| A_44_P320213 | 0.015882 | 0.813217 |  | Rn.198957 |
| A_44_P320754 | 0.030929 | 1.201994 | Olr37 | Rn.110414 |
| A_44_P320858 | 0.020395 | 1.185457 | Ghr | Rn.2178 |
| A_44_P321669 | 0.040702 | 1.248283 | Map3k3 | Rn.72680 |
| A_44_P321675 | 0.029597 | 1.223629 | Map3k3 | Rn.72680 |
| A_44_P322699 | 0.023561 | 0.684547 |  | Rn.224474 |
| A_44_P323496 | 0.008118 | 1.219004 | Inpp5d | Rn.10659 |
| A_44_P324093 | 0.040893 | 1.237168 | Spryd3 | Rn.9246 |
| A_44_P324116 | 0.013132 | 0.837671 | Tmem194b | Rn.168123 |
| A_44_P324508 | 0.013029 | 1.665583 |  | Rn.13452 |
| A_44_P324806 | 0.036056 | 0.787379 |  | Rn.8628 |
| A_44_P325426 | 0.039714 | 0.683538 |  | Rn.164817 |
| A_44_P325791 | 0.041631 | 0.853704 | Ywhag | Rn.29936 |
| A_44_P325971 | 0.007215 | 1.392417 |  |  |
| A_44_P326289 | 0.024443 | 0.805278 | Kdm4c | Rn.15348 |
| A_44_P327945 | 0.021786 | 1.229253 | G4 | Rn.205352 |
| A_44_P328806 | 0.009583 | 1.111771 |  | Rn.225442 |
| A_44_P330765 | 0.020114 | 1.347171 | Cdk20 | Rn.7013 |
| A_44_P333232 | 0.030964 | 1.428607 | Plekhf2 | Rn.11991 |
| A_44_P334494 | 0.002624 | 0.817688 |  | Rn.170015 |
| A_44_P334709 | 0.007191 | 1.15427 | Rps14 | Rn.36102 |
| A_44_P334940 | 0.021344 | 1.323349 | Ptpn13 | Rn.35546 |
| A_44_P336054 | 0.044666 | 0.641183 |  |  |
| A_44_P337488 | 0.027137 | 1.449493 | Pdxp | Rn.222285 |
| A_44_P339594 | 0.000736 | 1.93253 | Olr60 | Rn.141919 |
| A_44_P339708 | 0.018105 | 0.801869 | Camk2d | Rn.87208 |
| A_44_P340558 | 0.037205 | 0.449636 |  | Rn.145427 |
| A_44_P342208 | 0.015283 | 1.195734 |  |  |
| A_44_P343548 | 0.020582 | 0.673227 |  | Rn.198918 |
| A_44_P343888 | 0.006191 | 0.744329 |  | Rn.98833 |
| A_44_P344777 | 0.023138 | 0.302344 |  |  |
| A_44_P346832 | 0.007552 | 1.751748 | Tesc | Rn.198314 |
| A_44_P347938 | 0.03393 | 0.849398 | Grinl1a | Rn.144666 |
| A_44_P347981 | 0.01765 | 1.508999 |  | Rn.190831 |
| A_44_P349769 | 0.024279 | 0.723552 |  |  |
| A_44_P352016 | 0.028942 | 1.547613 |  |  |
| A_44_P355842 | 0.045593 | 1.380448 | Ly6g5c | Rn.213816 |
| A_44_P357172 | 0.000785 | 0.76594 |  | Rn.106017 |
| A_44_P358306 | 0.04386 | 0.740177 | Olr193 | Rn.141817 |
| A_44_P358358 | 0.048143 | 0.725418 | RT1-T18 | Rn.96971 |
| A_44_P359113 | 0.012156 | 1.935419 |  | Rn.82827 |
| A_44_P360126 | 0.027018 | 0.851384 |  |  |
| A_44_P360496 | 0.029991 | 1.274274 | Sec61a1 | Rn.47029 |
| A_44_P361041 | 0.020761 | 1.244329 |  |  |
| A_44_P362723 | 0.017092 | 0.83703 | Psmd7 | Rn.20659 |
| A_44_P363525 | 0.036681 | 0.779056 |  |  |
| A_44_P363736 | 0.033711 | 1.436303 | Fhl3 | Rn.100732 |
| A_44_P363824 | 0.044361 | 1.09634 | Brd1 | Rn.9133 |
| A_44_P363913 | 0.028031 | 0.364316 |  |  |
| A_44_P363937 | 0.0255 | 0.676725 |  | Rn.124542 |
| A_44_P367194 | 0.029335 | 0.823047 |  | Rn.101929 |
| A_44_P369617 | 0.010973 | 0.7474 |  | Rn.219234 |
| A_44_P372377 | 0.015339 | 0.815374 | Olr1439 | Rn.142135 |
| A_44_P372431 | 0.014378 | 1.595341 | LOC686180 | Rn.133963 |
| A_44_P373285 | 0.027092 | 0.72858 |  | Rn.2894 |
| A_44_P374247 | 0.041679 | 1.483085 |  | Rn.225886 |
| A_44_P375042 | 0.005514 | 0.835472 | Smg9 | Rn.32677 |
| A_44_P379647 | 0.01742 | 1.139313 | Uqcrc2 | Rn.2334 |
| A_44_P381008 | 0.000434 | 0.795359 |  | Rn.220063 |
| A_44_P381384 | 0.037547 | 0.841429 |  | Rn.223223 |
| A_44_P382438 | 0.024717 | 1.173045 | Supt7l | Rn.11526 |
| A_44_P384506 | 0.014078 | 1.386739 | Pragmin | Rn.51831 |
| A_44_P384698 | 0.044753 | 0.483408 |  |  |
| A_44_P384937 | 0.033195 | 1.168008 | Mterfd2 | Rn.41249 |
| A_44_P386734 | 0.047962 | 1.224523 | Trrap | Rn.92036 |
| A_44_P389216 | 0.000627 | 1.63543 | Nanos1 | Rn.218362 |
| A_44_P391345 | 0.00263 | 1.802345 | Slc6a11 | Rn.10545 |
| A_44_P391599 | 0.019624 | 1.2073 | Tbc1d7 | Rn.13671 |
| A_44_P391680 | 0.023327 | 1.193362 | Tnpo2 | Rn.228319 |
| A_44_P391688 | 0.018322 | 1.156194 | RGD1566073 | |
| A_44_P393731 | 0.04954 | 1.233852 | Cdc42bpg | Rn.99897 |
| A_44_P394636 | 0.035289 | 1.163145 | Inpp5d | Rn.10659 |
| A_44_P395173 | 0.003381 | 1.712068 |  | Rn.38932 |
| A_44_P395471 | 0.036285 | 1.092371 |  | Rn.165363 |
| A_44_P395975 | 0.002269 | 1.14101 | Ndnl2 | Rn.9406 |
| A_44_P395977 | 0.016708 | 1.148912 | Ndnl2 | Rn.9406 |
| A_44_P396817 | 0.018178 | 1.328656 | Prlh | Rn.91303 |
| A_44_P398300 | 0.027561 | 0.849141 | Fam160a2 | Rn.23025 |
| A_44_P398368 | 0.015871 | 0.737772 |  |  |
| A_44_P398522 | 0.015085 | 1.177087 | Aqr | Rn.37299 |
| A_44_P400884 | 0.027009 | 0.799747 |  |  |
| A_44_P401809 | 0.023287 | 1.108139 | Rps20 | Rn.103133 |
| A_44_P402884 | 0.003367 | 1.414792 | Pitpnm2 | Rn.75968 |
| A_44_P403065 | 0.03943 | 0.65838 |  |  |
| A_44_P403978 | 0.014417 | 0.823991 | Supt4h1 | Rn.7906 |
| A_44_P404898 | 0.018167 | 1.333998 | Espn | Rn.91373 |
| A_44_P405581 | 0.030844 | 1.114566 | Upf3b | Rn.29454 |
| A_44_P406775 | 0.005768 | 0.652949 | Dalrd3 | Rn.120770 |
| A_44_P408011 | 0.034552 | 0.889484 | Srsf10 | Rn.162118 |
| A_44_P408110 | 0.027076 | 1.260049 |  | Rn.7632 |
| A_44_P409696 | 0.001435 | 1.124517 | Raf1 | Rn.33262 |
| A_44_P410005 | 0.038489 | 0.907642 | Ugp2 | Rn.3415 |
| A_44_P410576 | 0.030362 | 1.421844 |  | Rn.23050 |
| A_44_P414087 | 0.016103 | 0.722154 |  | Rn.4294 |
| A_44_P414102 | 0.04022 | 1.055046 | Cox6c | Rn.846 |
| A_44_P414345 | 0.004913 | 0.687741 | Olr559 | Rn.142904 |
| A_44_P414700 | 0.041315 | 0.694903 |  |  |
| A_44_P414838 | 0.039435 | 0.547658 | RGD1561841 | |
| A_44_P415349 | 0.025892 | 0.753713 | Gata6 | Rn.8701 |
| A_44_P416477 | 0.01403 | 1.208508 | Uqcrc1 | Rn.3428 |
| A_44_P416761 | 0.017682 | 0.788521 | Lmna | Rn.44161 |
| A_44_P417083 | 0.005738 | 0.77353 | Atad1 | Rn.54918 |
| A_44_P417245 | 0.020351 | 1.45599 |  |  |
| A_44_P420005 | 0.021071 | 1.213126 | Ormdl3 | Rn.202345 |
| A_44_P421425 | 0.005999 | 0.781838 | Olr309 | Rn.141729 |
| A_44_P421539 | 0.034603 | 1.093087 | Cast | Rn.17481 |
| A_44_P423786 | 0.014363 | 1.102255 | Eef1g | Rn.43300 |
| A_44_P424164 | 0.025549 | 0.535895 |  |  |
| A_44_P424440 | 0.037884 | 0.628594 | LOC291480 | |
| A_44_P424665 | 0.048671 | 0.812371 |  |  |
| A_44_P425076 | 0.034464 | 1.110594 | Pi4kb | Rn.14991 |
| A_44_P425143 | 0.025935 | 0.804198 | Grin2d | Rn.91209 |
| A_44_P425481 | 0.009968 | 0.852781 |  | Rn.15451 |
| A_44_P426307 | 0.036284 | 0.755956 |  | Rn.158987 |
| A_44_P426387 | 0.005331 | 1.175992 | Snap29 | Rn.17902 |
| A_44_P426498 | 0.049304 | 0.78941 | RGD1564617 | |
| A_44_P428227 | 0.002413 | 0.8427 |  | Rn.202987 |
| A_44_P428228 | 0.033397 | 0.859822 |  | Rn.202987 |
| A_44_P428232 | 0.013592 | 0.830382 |  | Rn.202987 |
| A_44_P428233 | 0.00753 | 0.803859 |  | Rn.202987 |
| A_44_P429304 | 0.035434 | 0.844655 |  |  |
| A_44_P429354 | 0.026634 | 0.572408 |  |  |
| A_44_P429379 | 0.011067 | 0.601208 | RGD1564268 | |
| A_44_P431053 | 0.043377 | 1.357607 | Dpyd | Rn.158382 |
| A_44_P431092 | 0.044469 | 1.288066 | Cyp2d4 | Rn.26060 |
| A_44_P433383 | 0.009817 | 0.762773 |  |  |
| A_44_P433519 | 0.046138 | 0.835842 | Rhbdd2 | Rn.104587 |
| A_44_P433992 | 0.038823 | 0.836425 | Acot5 | Rn.145861 |
| A_44_P434030 | 0.045668 | 1.197819 | LOC100362836 | Rn.224852 |
| A_44_P434109 | 0.038492 | 0.767769 | Hes7 | Rn.218470 |
| A_44_P434118 | 0.014555 | 0.647235 |  |  |
| A_44_P435422 | 0.017922 | 0.551567 | Mef2a | Rn.162435 |
| A_44_P435596 | 0.038116 | 0.372149 | Zfp36 | Rn.82737 |
| A_44_P436352 | 0.044401 | 0.811628 | Pdzd11 | Rn.144513 |
| A_44_P438529 | 0.008838 | 0.533417 |  |  |
| A_44_P439597 | 0.035467 | 0.768768 |  | Rn.14919 |
| A_44_P441833 | 0.03709 | 0.743112 |  | Rn.162361 |
| A_44_P442802 | 0.044594 | 0.750114 | Rassf5 | Rn.36106 |
| A_44_P443572 | 0.016217 | 1.118066 | RGD1561360 | |
| A_44_P444116 | 0.004242 | 0.68481 | Aox1 |  |
| A_44_P444122 | 0.025282 | 0.840186 |  |  |
| A_44_P444343 | 0.03285 | 1.117777 |  | Rn.763 |
| A_44_P444897 | 0.017286 | 0.474464 |  | Rn.101115 |
| A_44_P445966 | 0.021163 | 1.519925 |  |  |
| A_44_P447066 | 0.021746 | 1.511984 |  | Rn.106944 |
| A_44_P448286 | 0.039643 | 1.303887 | Fam135a | Rn.43059 |
| A_44_P449297 | 0.040444 | 0.753072 |  | Rn.1078 |
| A_44_P449567 | 0.013769 | 0.72408 | Rtn4rl1 | Rn.22361 |
| A_44_P450258 | 0.007727 | 1.540637 | Fitm2 | Rn.107822 |
| A_44_P450307 | 0.046519 | 1.280389 | Eps8 | Rn.30310 |
| A_44_P451568 | 0.029099 | 0.791981 | Myo1e | Rn.8737 |
| A_44_P453870 | 0.033104 | 1.267754 |  | Rn.129121 |
| A_44_P453874 | 0.034931 | 1.209416 | Rpl7 | Rn.129121 |
| A_44_P455108 | 0.041235 | 0.429402 | Eif1 | Rn.137199 |
| A_44_P455715 | 0.006976 | 0.806309 |  | Rn.207938 |
| A_44_P456072 | 0.040547 | 0.789762 |  | Rn.225928 |
| A_44_P456836 | 0.022479 | 0.838455 | LOC100362849 | Rn.214247 |
| A_44_P459412 | 0.002157 | 1.603297 |  |  |
| A_44_P459679 | 0.015057 | 0.784063 | LOC100365604 | Rn.21749 |
| A_44_P460124 | 0.019747 | 1.322801 |  | Rn.25084 |
| A_44_P461201 | 0.011773 | 0.528332 | Pcsk7 | Rn.10653 |
| A_44_P461315 | 0.010749 | 1.068658 | Son | Rn.7967 |
| A_44_P461383 | 0.025745 | 1.814683 | Vom2r45 | Rn.89385 |
| A_44_P461545 | 0.01851 | 0.804022 |  | Rn.168951 |
| A_44_P461763 | 0.019755 | 1.184718 | Otud7b | Rn.199225 |
| A_44_P461932 | 0.040696 | 0.731461 |  |  |
| A_44_P461944 | 0.007584 | 0.810767 | Morf4l2 | Rn.6961 |
| A_44_P462018 | 0.0273 | 0.739675 | LOC100360990 | |
| A_44_P462823 | 0.004514 | 0.877441 | Nub1 | Rn.72440 |
| A_44_P464221 | 0.012707 | 0.598667 | RGD1566373 | |
| A_44_P465895 | 0.043847 | 1.094466 | Atp6v1f | Rn.6167 |
| A_44_P466832 | 0.040131 | 1.228805 | Adamts2 | Rn.86986 |
| A_44_P466845 | 0.019863 | 1.707319 |  | Rn.204593 |
| A_44_P466989 | 0.0138 | 1.204537 |  | Rn.3831 |
| A_44_P469188 | 0.01651 | 1.191403 | Appbp2 | Rn.104856 |
| A_44_P473437 | 0.019478 | 1.089328 | RGD1564425 | |
| A_44_P473449 | 0.025497 | 0.496167 |  |  |
| A_44_P473648 | 0.011332 | 0.508626 |  |  |
| A_44_P475444 | 0.0088 | 1.261519 | RGD1564614 | Rn.225202 |
| A_44_P477116 | 0.028183 | 0.863483 | Pcyt1a | Rn.81192 |
| A_44_P477371 | 0.015044 | 1.285999 |  | Rn.15594 |
| A_44_P478144 | 0.031766 | 1.479148 | LOC290595 | Rn.103148 |
| A_44_P483288 | 0.020089 | 1.219061 | Txndc17 | Rn.18447 |
| A_44_P484677 | 0.021913 | 0.777405 | Notch3 | Rn.53876 |
| A_44_P484877 | 0.006223 | 1.28843 |  |  |
| A_44_P484950 | 0.019897 | 1.122988 | Atp5g3 | Rn.2180 |
| A_44_P487300 | 0.032708 | 0.637933 |  |  |
| A_44_P487482 | 0.045238 | 0.691143 |  |  |
| A_44_P489793 | 0.049943 | 0.894294 |  |  |
| A_44_P490235 | 0.040622 | 0.384446 |  |  |
| A_44_P490265 | 0.022139 | 0.79435 | RGD1564730 | |
| A_44_P490296 | 0.021546 | 1.262402 | Gga3 | Rn.198912 |
| A_44_P491187 | 0.010173 | 0.864127 |  | Rn.3589 |
| A_44_P491929 | 0.030828 | 1.165704 | Prpf18 | Rn.203914 |
| A_44_P492409 | 0.033803 | 0.895126 | Kdm4c | Rn.15348 |
| A_44_P492629 | 0.043064 | 1.151549 | Znhit3 | Rn.224655 |
| A_44_P492651 | 0.035651 | 1.39207 | Tbcd | Rn.214589 |
| A_44_P493747 | 0.047327 | 0.805046 |  | Rn.86985 |
| A_44_P493980 | 0.032813 | 1.254849 | Nptxr | Rn.34907 |
| A_44_P494779 | 0.040214 | 1.112926 |  |  |
| A_44_P497107 | 0.039247 | 0.657151 |  | Rn.198729 |
| A_44_P497339 | 0.015121 | 1.222915 | Asf1a | Rn.163427 |
| A_44_P498620 | 0.043874 | 1.454125 | Cga | Rn.10589 |
| A_44_P499088 | 0.011894 | 1.227599 | Rpp30 | Rn.17421 |
| A_44_P500457 | 0.026332 | 0.68249 |  | Rn.5789 |
| A_44_P501602 | 0.037346 | 0.888994 |  |  |
| A_44_P506124 | 0.030477 | 0.761364 |  |  |
| A_44_P506447 | 0.034805 | 1.377955 |  |  |
| A_44_P506568 | 0.02548 | 0.376274 |  |  |
| A_44_P507469 | 0.045733 | 0.600653 | Myof | Rn.73651 |
| A_44_P508066 | 0.032062 | 0.81202 | Nnat | Rn.5785 |
| A_44_P508307 | 0.025843 | 0.848337 | Rgs11 | Rn.84083 |
| A_44_P508348 | 0.005261 | 0.833757 | Ahnak | Rn.226248 |
| A_44_P508573 | 0.037721 | 0.521762 | Cr2 | Rn.23490 |
| A_44_P508809 | 0.024343 | 0.795867 | Dpm1 | Rn.7840 |
| A_44_P514140 | 0.026042 | 0.843423 |  | Rn.220617 |
| A_44_P514431 | 0.016468 | 1.19494 |  | Rn.8982 |
| A_44_P514987 | 0.012234 | 1.204835 | Tshz1 | Rn.205514 |
| A_44_P515538 | 0.012595 | 0.734447 | LOC688812 | Rn.224991 |
| A_44_P515560 | 0.044638 | 1.15508 | RGD1310788 | Rn.196112 |
| A_44_P515888 | 0.049547 | 1.192705 | Col6a2 | Rn.128903 |
| A_44_P517614 | 0.046653 | 1.17529 | Trak2 | Rn.26957 |
| A_44_P517944 | 0.039501 | 0.574627 |  |  |
| A_44_P518176 | 0.019301 | 0.801095 | Ccdc90a | Rn.162214 |
| A_44_P518528 | 0.001023 | 1.171308 | Trim11 | Rn.9308 |
| A_44_P520992 | 0.017398 | 0.855654 |  | Rn.212709 |
| A_44_P522293 | 0.03954 | 1.106215 | Ufc1 | Rn.8050 |
| A_44_P523170 | 0.046162 | 1.17041 |  |  |
| A_44_P523261 | 0.007773 | 1.169334 | RGD1559743 | Rn.225264 |
| A_44_P524953 | 0.004831 | 1.137384 | Rpl6 | Rn.2660 |
| A_44_P525110 | 0.009266 | 0.433885 |  |  |
| A_44_P527123 | 0.035808 | 1.380208 | Hoxb6 | Rn.203800 |
| A_44_P527253 | 0.023065 | 1.222361 | Hmgcl | Rn.12297 |
| A_44_P527376 | 0.048998 | 1.193501 | Ate1 | Rn.40326 |
| A_44_P528235 | 0.0002 | 1.33199 |  |  |
| A_44_P529114 | 0.029394 | 0.724679 |  | Rn.1425 |
| A_44_P529418 | 0.030825 | 0.912059 | Akap9 | Rn.57458 |
| A_44_P529672 | 0.030984 | 0.6619 |  |  |
| A_44_P529691 | 0.019873 | 0.745502 | Rbbp8 | Rn.128724 |
| A_44_P530187 | 0.009574 | 0.814648 | Plekho2 |  |
| A_44_P532057 | 0.007933 | 0.905324 | LOC501979 | Rn.1742 |
| A_44_P532686 | 0.005811 | 0.515064 | Efnb1 | Rn.44398 |
| A_44_P533172 | 0.00993 | 1.146718 | Wdr26 | Rn.23078 |
| A_44_P534490 | 0.030032 | 1.970049 | Slc5a4a | Rn.133639 |
| A_44_P534518 | 0.035259 | 1.206608 | Abtb1 | Rn.40721 |
| A_44_P534781 | 0.036833 | 0.27687 | RGD1564550 | |
| A_44_P535480 | 0.028639 | 0.717198 | LOC100360679 | Rn.199092 |
| A_44_P535993 | 0.005505 | 1.51352 |  | Rn.7755 |
| A_44_P536553 | 0.010052 | 0.847169 |  |  |
| A_44_P536951 | 0.032054 | 1.308297 |  |  |
| A_44_P538882 | 0.035681 | 1.482997 | Dnmbp | Rn.11811 |
| A_44_P539111 | 0.030577 | 1.221383 | Leprel2 |  |
| A_44_P539215 | 0.021696 | 0.789503 |  |  |
| A_44_P539231 | 0.038286 | 0.429336 |  |  |
| A_44_P540570 | 0.024168 | 0.394198 | Cox7c | Rn.105968 |
| A_44_P541074 | 0.025958 | 0.761053 | RragB | Rn.203382 |
| A_44_P541623 | 0.035715 | 0.680508 | Trabd | Rn.3565 |
| A_44_P541937 | 0.031886 | 1.232207 | Mid2 | Rn.15520 |
| A_44_P542785 | 0.038305 | 0.861343 | Ube2k | Rn.203330 |
| A_44_P543576 | 0.001979 | 0.807795 |  |  |
| A_44_P547745 | 0.008359 | 0.797775 |  | Rn.190571 |
| A_44_P548524 | 0.033982 | 1.109989 | RGD1564093 | Rn.121465 |
| A_44_P549445 | 0.033157 | 1.156373 | Cs | Rn.66581 |
| A_44_P549998 | 0.049162 | 1.08968 | Dhps | Rn.105891 |
| A_44_P550918 | 0.002636 | 1.231552 |  |  |
| A_44_P550981 | 0.034178 | 1.178216 | Dmap1 | Rn.22845 |
| A_44_P551909 | 0.01685 | 0.852188 | Ep400 | Rn.76053 |
| A_44_P553158 | 0.017532 | 1.124319 | Nip30 | Rn.6506 |
| A_44_P554965 | 0.03506 | 1.822223 | Mamdc4 | Rn.10059 |
| A_44_P555567 | 0.031817 | 0.870254 | Zfp407 | Rn.28602 |
| A_44_P555664 | 0.044144 | 0.548989 |  |  |
| A_44_P555890 | 0.041864 | 1.385561 | Mnt | Rn.30696 |
| A_44_P557575 | 0.02775 | 1.144818 |  |  |
| A_44_P557752 | 0.030187 | 0.803164 |  |  |
| A_44_P557921 | 0.045017 | 0.864412 | Stk38 | Rn.13142 |
| A_44_P558846 | 0.037764 | 0.884834 |  | Rn.164412 |
| A_44_P559080 | 0.04737 | 1.207054 | Ankrd44 | Rn.136174 |
| A_44_P560036 | 0.007056 | 0.742595 |  |  |
| A_44_P560038 | 0.030559 | 0.770578 |  |  |
| A_44_P560195 | 0.035578 | 0.701644 |  |  |
| A_44_P561352 | 0.015275 | 0.883487 | Meaf6 | Rn.13608 |
| A_44_P562830 | 0.042154 | 1.265139 | Dusp15 | Rn.58260 |
| A_44_P571530 | 0.011099 | 0.811456 |  | Rn.3997 |
| A_44_P571978 | 0.019518 | 0.675049 |  | Rn.170050 |
| A_44_P575319 | 0.009709 | 1.293161 |  |  |
| A_44_P575533 | 0.046619 | 1.111747 | Rpl31 | Rn.1101 |
| A_44_P575763 | 0.016257 | 0.535178 |  |  |
| A_44_P577138 | 0.042107 | 1.197442 |  |  |
| A_44_P583151 | 0.035928 | 1.399171 |  |  |
| A_44_P586190 | 0.001436 | 1.682063 |  |  |
| A_44_P587335 | 7.02E-06 | 0.669754 |  |  |
| A_44_P591437 | 0.003412 | 0.896473 | Polr3d | Rn.16896 |
| A_44_P593525 | 0.041478 | 0.878789 |  | Rn.105357 |
| A_44_P599208 | 0.019382 | 1.164522 |  | Rn.181326 |
| A_44_P602663 | 0.014659 | 0.608009 |  | Rn.164226 |
| A_44_P603427 | 0.03845 | 1.318768 | LOC100359861 | Rn.21474 |
| A_44_P607547 | 0.030264 | 0.840005 |  |  |
| A_44_P608715 | 0.018873 | 0.753961 |  |  |
| A_44_P609428 | 0.02283 | 1.311842 |  | Rn.209618 |
| A_44_P610959 | 0.003224 | 1.282415 |  |  |
| A_44_P613318 | 0.003037 | 0.709453 |  | Rn.202748 |
| A_44_P614514 | 0.009955 | 0.644675 |  | Rn.155246 |
| A_44_P621338 | 0.012806 | 0.769932 | Zfp458 | Rn.122959 |
| A_44_P621366 | 0.023637 | 0.49782 |  |  |
| A_44_P621378 | 0.04763 | 0.750664 |  |  |
| A_44_P621592 | 0.007554 | 1.180279 |  |  |
| A_44_P623236 | 0.047317 | 1.307017 | Rbak | Rn.137256 |
| A_44_P623560 | 0.009868 | 0.632612 |  |  |
| A_44_P628867 | 0.042505 | 1.203446 |  | Rn.199674 |
| A_44_P633381 | 0.030531 | 1.515421 |  |  |
| A_44_P637322 | 0.028294 | 1.170757 |  |  |
| A_44_P638014 | 0.019537 | 0.78405 | Irf6 | Rn.12385 |
| A_44_P638087 | 0.041625 | 1.167926 |  | Rn.7993 |
| A_44_P638377 | 0.011008 | 0.797144 |  |  |
| A_44_P638565 | 0.028708 | 1.379504 |  |  |
| A_44_P640364 | 0.028048 | 1.206563 |  |  |
| A_44_P649412 | 0.022513 | 0.730999 |  | Rn.162695 |
| A_44_P650082 | 0.034012 | 0.796654 |  | Rn.169873 |
| A_44_P651992 | 0.010833 | 0.508609 |  |  |
| A_44_P653240 | 0.007672 | 0.888243 | Eif4ebp3 | Rn.11853 |
| A_44_P653949 | 0.007884 | 1.927092 |  | Rn.153833 |
| A_44_P654096 | 0.021735 | 1.273413 |  |  |
| A_44_P654250 | 0.034528 | 0.696843 |  |  |
| A_44_P659386 | 0.010341 | 1.700959 |  |  |
| A_44_P661802 | 0.031562 | 1.169893 | Eif2a | Rn.198593 |
| A_44_P667069 | 0.024156 | 0.687978 | Qsox2 | Rn.199433 |
| A_44_P667509 | 0.030383 | 0.343095 |  |  |
| A_44_P667648 | 0.019246 | 0.546698 |  |  |
| A_44_P672275 | 0.038631 | 1.627774 |  |  |
| A_44_P674922 | 0.006594 | 2.116773 |  | Rn.42011 |
| A_44_P681544 | 0.008797 | 1.171084 |  | Rn.77521 |
| A_44_P683392 | 0.046489 | 0.842815 |  |  |
| A_44_P683608 | 0.007043 | 0.655893 |  | Rn.167770 |
| A_44_P696677 | 0.029459 | 1.159434 |  | Rn.41920 |
| A_44_P698456 | 0.033716 | 1.43719 |  |  |
| A_44_P699410 | 0.015724 | 0.808647 |  |  |
| A_44_P699502 | 0.020821 | 0.806077 | LOC687105 | Rn.216416 |
| A_44_P700917 | 0.042655 | 1.197576 |  |  |
| A_44_P701112 | 0.031648 | 1.173553 |  | Rn.167930 |
| A_44_P704947 | 0.021531 | 1.193923 |  |  |
| A_44_P707159 | 0.019944 | 1.261456 | LOC100365370 | Rn.144602 |
| A_44_P713311 | 0.010421 | 0.883671 | Ncor1 | Rn.24948 |
| A_44_P713474 | 0.029823 | 1.119502 |  |  |
| A_44_P713802 | 0.004346 | 0.688956 | LOC100360380 | Rn.225673 |
| A_44_P714771 | 0.008448 | 1.230111 |  |  |
| A_44_P715164 | 0.016166 | 1.278228 |  | Rn.205471 |
| A_44_P720147 | 0.048731 | 1.598175 |  | Rn.16029 |
| A_44_P723176 | 0.023583 | 1.325302 |  | Rn.212883 |
| A_44_P723917 | 0.019522 | 0.765074 |  |  |
| A_44_P726568 | 0.033015 | 1.120598 | Ptpn3 | Rn.22271 |
| A_44_P729183 | 0.034403 | 0.697827 |  |  |
| A_44_P730099 | 0.035466 | 0.835241 |  |  |
| A_44_P731013 | 0.037225 | 1.214761 |  |  |
| A_44_P744765 | 0.014911 | 0.626496 |  |  |
| A_44_P745478 | 0.042695 | 0.848234 |  | Rn.8858 |
| A_44_P747084 | 0.019888 | 0.746008 |  |  |
| A_44_P747545 | 0.041517 | 1.280367 | Xpr1 | Rn.138575 |
| A_44_P748217 | 0.027053 | 0.656573 |  | Rn.42633 |
| A_44_P748361 | 0.037905 | 0.679163 |  |  |
| A_44_P753601 | 0.010711 | 0.742185 |  |  |
| A_44_P759911 | 0.031024 | 0.491338 |  |  |
| A_44_P760225 | 0.018755 | 0.478231 |  |  |
| A_44_P760260 | 0.025573 | 1.625826 |  |  |
| A_44_P760327 | 0.040446 | 1.062165 | MGC94282 | Rn.224923 |
| A_44_P761041 | 0.026165 | 1.222828 | Trim2 | Rn.16973 |
| A_44_P761179 | 0.034085 | 0.821016 |  |  |
| A_44_P761886 | 0.018394 | 0.856365 | Zswim1 | Rn.27453 |
| A_44_P767065 | 0.041232 | 1.352199 |  | Rn.168798 |
| A_44_P767445 | 0.040707 | 0.7188 |  |  |
| A_44_P776209 | 0.018868 | 0.75397 | Dpy19l1 | Rn.22725 |
| A_44_P777373 | 0.009359 | 0.749026 |  |  |
| A_44_P777617 | 0.016651 | 1.682014 |  |  |
| A_44_P777874 | 0.027703 | 0.817625 |  |  |
| A_44_P777990 | 0.041693 | 1.121501 |  |  |
| A_44_P778557 | 0.011432 | 1.182525 |  | Rn.206928 |
| A_44_P782073 | 0.032011 | 0.845443 |  | Rn.178219 |
| A_44_P787835 | 0.023802 | 0.857867 |  | Rn.216517 |
| A_44_P788355 | 0.039168 | 1.164158 | Pcdhb20 | Rn.198396 |
| A_44_P790417 | 0.004771 | 1.847283 | Spert | Rn.136809 |
| A_44_P790750 | 0.025201 | 0.652497 |  |  |
| A_44_P790825 | 0.034171 | 0.758486 |  |  |
| A_44_P791729 | 0.046376 | 0.550791 | Rtn4rl1 | Rn.22361 |
| A_44_P792192 | 0.010758 | 0.858146 | Srp54a | Rn.24880 |
| A_44_P797354 | 0.010251 | 1.480008 |  | Rn.17369 |
| A_44_P798851 | 0.016896 | 1.390765 |  | Rn.177278 |
| A_44_P799393 | 0.037444 | 0.784097 |  | Rn.41732 |
| A_44_P802495 | 0.044429 | 1.11501 | Apbb2 | Rn.24283 |
| A_44_P804447 | 0.015605 | 1.743762 |  | Rn.89832 |
| A_44_P804460 | 0.003888 | 1.266687 |  | Rn.122139 |
| A_44_P806572 | 0.022497 | 0.473711 |  |  |
| A_44_P806646 | 0.014584 | 0.787602 | Zfp609 | Rn.161077 |
| A_44_P806699 | 0.045052 | 1.277819 | St8sia4 | Rn.63696 |
| A_44_P807338 | 0.010372 | 0.803894 | Cog3 | Rn.163311 |
| A_44_P810190 | 0.005681 | 0.809345 | LOC686980 | |
| A_44_P831621 | 0.014969 | 0.855016 |  |  |
| A_44_P831631 | 0.04883 | 1.247997 | RGD1562161 | Rn.214618 |
| A_44_P834642 | 0.025971 | 1.064001 |  | Rn.979 |
| A_44_P835675 | 0.038884 | 0.863345 |  | Rn.2762 |
| A_44_P836900 | 0.039777 | 1.105922 |  |  |
| A_44_P837669 | 0.023048 | 0.740972 | Abce1 | Rn.2961 |
| A_44_P838603 | 0.025639 | 0.843524 | Pycr1 | Rn.99704 |
| A_44_P839176 | 0.044036 | 0.840693 | Usp25 | Rn.23509 |
| A_44_P842451 | 0.038502 | 1.156649 |  |  |
| A_44_P848759 | 0.024458 | 0.641183 |  | Rn.203336 |
| A_44_P852219 | 0.040574 | 1.155281 |  |  |
| A_44_P852241 | 0.04546 | 1.254401 |  |  |
| A_44_P852338 | 0.034346 | 1.075117 | RGD1563145 | |
| A_44_P852623 | 0.048047 | 0.61142 |  |  |
| A_44_P853733 | 0.009282 | 0.702676 |  |  |
| A_44_P854437 | 0.046145 | 1.323371 |  |  |
| A_44_P854619 | 0.006414 | 1.344839 |  |  |
| A_44_P855808 | 0.004982 | 0.831763 |  |  |
| A_44_P867279 | 0.000562 | 0.553943 |  | Rn.33804 |
| A_44_P867427 | 0.019059 | 1.380452 |  |  |
| A_44_P867638 | 0.046143 | 1.089525 |  |  |
| A_44_P879099 | 0.004435 | 0.488933 |  |  |
| A_44_P880354 | 0.009514 | 0.873696 |  | Rn.3502 |
| A_44_P880417 | 0.019998 | 0.537859 |  | Rn.101986 |
| A_44_P881890 | 0.036383 | 1.122296 | RGD1559442 | Rn.37595 |
| A_44_P882084 | 0.009965 | 0.86663 | Slc9a8 | Rn.153853 |
| A_44_P882921 | 0.024581 | 0.799506 |  |  |
| A_44_P883350 | 0.03308 | 0.575266 |  |  |
| A_44_P884616 | 0.015024 | 0.869764 | Zfat | Rn.33031 |
| A_44_P886161 | 0.002776 | 1.16843 |  |  |
| A_44_P886818 | 0.011095 | 0.589114 |  |  |
| A_44_P888285 | 0.034691 | 1.411082 |  |  |
| A_44_P898694 | 0.046735 | 0.706756 |  |  |
| A_44_P900655 | 0.024902 | 0.861907 |  | Rn.33257 |
| A_44_P900688 | 0.017239 | 0.842167 |  |  |
| A_44_P901064 | 0.04183 | 0.564317 |  | Rn.4896 |
| A_44_P902244 | 0.038307 | 1.159715 | Rnf14 | Rn.115357 |
| A_44_P913360 | 0.043173 | 0.679083 |  |  |
| A_44_P913689 | 0.018288 | 0.759335 |  |  |
| A_44_P914632 | 0.016051 | 0.678188 |  |  |
| A_44_P917870 | 0.044814 | 0.731252 |  |  |
| A_44_P920991 | 0.021409 | 0.815959 | Pter | Rn.44484 |
| A_44_P928592 | 0.043807 | 0.763162 |  |  |
| A_44_P928969 | 0.00538 | 0.778972 |  |  |
| A_44_P929119 | 0.023344 | 0.531805 |  |  |
| A_44_P931865 | 0.019488 | 1.111819 | Fam160b2 | Rn.124637 |
| A_44_P932635 | 0.046872 | 0.688362 |  |  |
| A_44_P933459 | 0.045217 | 1.164883 | RGD1308874 | Rn.2352 |
| A_44_P938073 | 0.042708 | 0.752647 |  |  |
| A_44_P944294 | 0.044836 | 1.159642 | Rfxap | Rn.198587 |
| A_44_P945096 | 0.031713 | 0.863976 | Glyr1 | Rn.2639 |
| A_44_P946803 | 0.048748 | 1.143018 |  |  |
| A_44_P947342 | 0.016359 | 0.505673 |  |  |
| A_44_P952373 | 0.029874 | 0.809633 |  | Rn.100240 |
| A_44_P955940 | 0.021785 | 0.831389 | Dhx36 | Rn.2744 |
| A_44_P958650 | 0.020552 | 0.77882 | Isy1 | Rn.25170 |
| A_44_P959572 | 0.039914 | 1.150177 | Eef1g | Rn.43300 |
| A_44_P961164 | 0.00271 | 0.577918 | Rhbdl3 | Rn.20693 |
| A_44_P961999 | 0.00523 | 1.571311 |  |  |
| A_44_P967625 | 0.048302 | 0.881427 | Rps25 | Rn.60855 |
| A_44_P969565 | 0.048935 | 1.306803 |  |  |
| A_44_P971443 | 0.037568 | 1.173302 |  | Rn.7260 |
| A_44_P973562 | 0.003338 | 0.650665 | Tspan5 | Rn.98240 |
| A_44_P974489 | 0.028698 | 1.328619 | Dusp22 | Rn.162221 |
| A_44_P974975 | 0.048332 | 0.745817 |  | Rn.196482 |
| A_44_P975235 | 0.04011 | 0.91426 | RGD1309220 | Rn.34623 |
| A_44_P975471 | 0.041546 | 1.287712 | RGD1560398 | Rn.204292 |
| A_44_P978216 | 0.031908 | 1.291639 |  |  |
| A_44_P986636 | 0.034513 | 1.158926 |  | Rn.199245 |
| A_44_P988400 | 0.017568 | 0.529285 |  | Rn.155647 |
| A_44_P991045 | 0.045219 | 0.832708 | RGD1359460 | Rn.13090 |
| A_44_P991459 | 0.024669 | 0.543115 | Nsmce2 | Rn.203454 |
| A_44_P991949 | 0.039989 | 0.63162 | Cox18 | Rn.24568 |
| A_44_P992306 | 0.008389 | 1.183568 | Gnpda2 | Rn.228412 |
| A_44_P992468 | 0.033839 | 1.078184 | Trappc5 | Rn.162083 |
| A_44_P992854 | 0.012256 | 0.839819 | Rhoa | Rn.107401 |
| A_44_P993027 | 0.023301 | 0.803251 | Pla2g16 | Rn.11377 |

| Table A2 Analyzed transcriptomics parameters of lung tissue of Bufei Jianpi formula-treated rats | | | | |
| --- | --- | --- | --- | --- |
| ProbeName | pvalues | foldchange | GeneSymbol | UniGeneID |
| A_42_P453935 | 0.026082 | 0.898358 | Naca | Rn.162909 |
| A_42_P455785 | 0.039834 | 0.914392 | Copb1 | Rn.4327 |
| A_42_P457773 | 0.043732 | 0.78193 | Txndc15 | Rn.4142 |
| A_42_P459149 | 0.006588 | 0.706239 | Phkg2 | Rn.11153 |
| A_42_P460122 | 0.001662 | 0.879363 | Higd2a | Rn.13525 |
| A_42_P466283 | 0.015812 | 0.692501 | Gzmk | Rn.11190 |
| A_42_P467994 | 0.030077 | 0.903586 | Slc12a4 | Rn.32091 |
| A_42_P468038 | 0.01831 | 0.940734 | Rps25 | Rn.60855 |
| A_42_P468632 | 0.009319 | 0.954582 | Rps28 | Rn.6005 |
| A_42_P470649 | 0.01552 | 0.425748 | Dio1 | Rn.87549 |
| A_42_P472304 | 0.028999 | 0.664827 | Phf15 | Rn.99384 |
| A_42_P481176 | 0.016484 | 0.902805 | Scamp4 | Rn.7309 |
| A_42_P483419 | 0.0079 | 0.83372 | Oaz1 | Rn.266 |
| A_42_P492135 | 0.041352 | 0.892591 | Pcbp4 | Rn.40405 |
| A_42_P493785 | 0.001135 | 1.323996 | Cdk2ap1 | Rn.40447 |
| A_42_P495397 | 0.019948 | 1.083331 | Rpl21 | Rn.83200 |
| A_42_P499546 | 0.003176 | 1.334203 | Hrasls5 | Rn.136627 |
| A_42_P501877 | 0.02629 | 0.776372 | Gls | Rn.5762 |
| A_42_P501972 | 0.013625 | 0.893072 | Ppp2r1b | Rn.163017 |
| A_42_P502671 | 0.002202 | 1.129895 | Znrf2 | Rn.32245 |
| A_42_P504077 | 0.022209 | 1.216825 | Pdxp | Rn.222285 |
| A_42_P504507 | 0.044806 | 0.932915 |  | Rn.1432 |
| A_42_P505280 | 0.03071 | 0.787801 | Sae1 | Rn.9014 |
| A_42_P505972 | 0.011878 | 1.292063 | Ube2s | Rn.120398 |
| A_42_P515931 | 0.043573 | 0.849575 |  | Rn.202598 |
| A_42_P516561 | 0.049216 | 0.861918 | Atn1 | Rn.11305 |
| A_42_P516941 | 0.031748 | 1.297029 | Ube4b | Rn.211830 |
| A_42_P519770 | 0.049712 | 0.917049 | Rpl29 | Rn.3973 |
| A_42_P521940 | 0.010731 | 0.858758 |  |  |
| A_42_P522899 | 0.013034 | 0.815079 | Tgfb1 | Rn.40136 |
| A_42_P527788 | 0.011929 | 0.565242 | Susd2 | Rn.166705 |
| A_42_P527887 | 0.040238 | 0.895246 | Atg4b | Rn.163086 |
| A_42_P529193 | 0.002146 | 1.233076 | Sdhc | Rn.1698 |
| A_42_P529445 | 0.010581 | 0.749309 | Prorsd1 | Rn.108544 |
| A_42_P532505 | 0.010047 | 1.799495 | Ccl11 | Rn.10632 |
| A_42_P536266 | 0.037513 | 0.783694 |  |  |
| A_42_P537881 | 0.003558 | 1.264653 | Itpa | Rn.203856 |
| A_42_P537971 | 0.039562 | 0.805104 | Pard6b | Rn.21123 |
| A_42_P538670 | 0.011198 | 0.783897 | Cybb | Rn.98491 |
| A_42_P539352 | 0.034694 | 1.397535 | Adrm1 | Rn.9320 |
| A_42_P540908 | 0.008084 | 0.842024 | Asna1 | Rn.7354 |
| A_42_P541857 | 0.019178 | 0.889104 | Cab39 | Rn.103055 |
| A_42_P544108 | 0.025406 | 0.897147 | Rplp0 | Rn.1079 |
| A_42_P545721 | 0.009522 | 1.199842 |  | Rn.3243 |
| A_42_P548796 | 0.013865 | 0.882835 | Tsn | Rn.156195 |
| A_42_P549271 | 0.04155 | 0.711197 | Mrpl43 | Rn.225372 |
| A_42_P552233 | 0.042552 | 1.10694 | Cct6a | Rn.33807 |
| A_42_P552807 | 0.001114 | 0.89523 | Hcfc1 | Rn.94043 |
| A_42_P553885 | 0.041651 | 0.783967 | Synpo | Rn.42910 |
| A_42_P559085 | 0.041102 | 0.871063 | Sos2 | Rn.4226 |
| A_42_P559874 | 0.017182 | 0.876001 |  | Rn.165195 |
| A_42_P561930 | 0.048229 | 1.259864 |  | Rn.50150 |
| A_42_P561946 | 0.013929 | 1.107668 | Pex12 | Rn.228342 |
| A_42_P571169 | 0.049297 | 1.091109 | Ttc17 | Rn.207415 |
| A_42_P572366 | 0.004534 | 0.882207 | Kat7 | Rn.12618 |
| A_42_P581001 | 0.048116 | 0.892746 | S100a6 | Rn.3233 |
| A_42_P585467 | 0.016155 | 0.875167 | Rab25 | Rn.7929 |
| A_42_P586681 | 0.012561 | 0.860865 | Upf2 | Rn.106291 |
| A_42_P592059 | 0.003442 | 1.1332 |  | Rn.2168 |
| A_42_P598733 | 0.021811 | 0.876669 | Dnajc21 | Rn.53644 |
| A_42_P600372 | 0.030288 | 1.540662 | Creb3l1 | Rn.24541 |
| A_42_P600947 | 0.03176 | 0.858568 | Dpp9 | Rn.41201 |
| A_42_P614547 | 0.04516 | 0.893035 | Ep400 | Rn.76053 |
| A_42_P618436 | 0.000309 | 0.860137 | Mfsd5 | Rn.17060 |
| A_42_P619114 | 0.029778 | 0.808777 | Dnajc22 | Rn.19564 |
| A_42_P619403 | 0.001683 | 0.639497 | Fam89a | Rn.66325 |
| A_42_P621628 | 0.02285 | 0.837139 | Fam63a | Rn.7768 |
| A_42_P621882 | 0.038131 | 1.069584 | Copa | Rn.203109 |
| A_42_P621952 | 0.00631 | 0.845814 |  | Rn.32366 |
| A_42_P622574 | 0.000207 | 0.824476 | Daxx | Rn.870 |
| A_42_P622746 | 0.025848 | 0.882252 | Zc3h13 | Rn.100827 |
| A_42_P624251 | 1.11E-05 | 1.88435 | Edc4 | Rn.145173 |
| A_42_P626104 | 0.044903 | 1.299914 | Fbxw11 | Rn.159760 |
| A_42_P627330 | 0.019219 | 1.172824 | Mrps35 | Rn.8870 |
| A_42_P633958 | 0.025688 | 0.663012 | Col4a2 | Rn.2237 |
| A_42_P634825 | 0.011007 | 0.879478 | Stambp | Rn.14662 |
| A_42_P638128 | 0.003767 | 1.762639 | Actg2 | Rn.958 |
| A_42_P638474 | 0.018811 | 1.236445 |  |  |
| A_42_P639337 | 0.031769 | 1.112719 | Ptges3l1 | Rn.211968 |
| A_42_P642557 | 0.000998 | 0.803998 | Arrdc1 | Rn.14816 |
| A_42_P647710 | 0.000774 | 0.766084 | Farsa | Rn.2972 |
| A_42_P648597 | 0.010374 | 0.864581 | Kat5 | Rn.6629 |
| A_42_P649762 | 0.001766 | 0.860285 | Sh3gl1 | Rn.8762 |
| A_42_P658562 | 0.035302 | 0.87757 | Ints10 | Rn.2385 |
| A_42_P660334 | 0.018639 | 1.213884 | Ssu72 | Rn.103950 |
| A_42_P668858 | 0.005189 | 1.386801 | Hnf1b | Rn.11342 |
| A_42_P670631 | 0.012762 | 1.579116 | RGD1305645 | Rn.16593 |
| A_42_P671125 | 0.003093 | 0.86689 | Trim28 | Rn.198494 |
| A_42_P671285 | 0.032813 | 0.725513 | Ctdspl | Rn.37030 |
| A_42_P672401 | 0.003641 | 0.889235 | Dhx30 | Rn.163034 |
| A_42_P685418 | 0.011664 | 1.156626 | Rbm8a | Rn.37716 |
| A_42_P687133 | 0.039915 | 0.811558 | Nrgn | Rn.11236 |
| A_42_P688442 | 0.031542 | 0.817683 | Usf1 | Rn.10845 |
| A_42_P688815 | 0.023618 | 1.197465 | Slc12a7 | Rn.64199 |
| A_42_P689905 | 0.038008 | 0.682525 | Apoc2 | Rn.16843 |
| A_42_P696578 | 0.004056 | 0.876221 | Scnm1 | Rn.40268 |
| A_42_P697274 | 0.023982 | 0.785968 |  | Rn.113703 |
| A_42_P698188 | 0.037047 | 0.802174 | Neo1 | Rn.10667 |
| A_42_P703403 | 0.011818 | 1.140045 | Clta | Rn.112599 |
| A_42_P707992 | 0.027915 | 2.782279 | Tspan18 | Rn.145602 |
| A_42_P708569 | 0.000815 | 1.193963 | Ube3a | Rn.162420 |
| A_42_P709819 | 0.029312 | 0.891626 | Tmed7 | Rn.67055 |
| A_42_P712718 | 0.000441 | 0.814347 | Mmadhc | Rn.3269 |
| A_42_P713975 | 0.0137 | 0.787454 | Swi5 | Rn.30992 |
| A_42_P718324 | 0.007139 | 0.892717 | Usp34 | Rn.16057 |
| A_42_P726132 | 0.021436 | 0.902616 | Tmem62 | Rn.138047 |
| A_42_P727530 | 0.020793 | 1.098699 | Mnat1 | Rn.15168 |
| A_42_P728472 | 0.034176 | 0.825808 | Rps4x | Rn.40171 |
| A_42_P733295 | 0.026637 | 0.869238 | Armc5 | Rn.15020 |
| A_42_P733814 | 0.043828 | 1.114269 | Tox | Rn.161967 |
| A_42_P734749 | 0.020864 | 0.751291 | Lrfn3 | Rn.50030 |
| A_42_P736967 | 0.006003 | 1.310309 | Zdhhc18 | Rn.203345 |
| A_42_P740386 | 0.030329 | 0.871113 | Zfp513 | Rn.15926 |
| A_42_P743825 | 0.031304 | 1.202542 | Psme3 | Rn.198325 |
| A_42_P749968 | 1.43E-06 | 1.52609 | Smg8 | Rn.45630 |
| A_42_P751368 | 0.004087 | 0.844414 | RGD1304704 | Rn.1434 |
| A_42_P751720 | 0.034474 | 0.631973 | LOC683720 | |
| A_42_P752901 | 0.03589 | 1.055781 | Rpl7a | Rn.144857 |
| A_42_P753270 | 0.022576 | 0.722553 | Zfp444 | Rn.43215 |
| A_42_P756393 | 0.02455 | 0.856413 |  | Rn.28951 |
| A_42_P759192 | 0.000195 | 0.911936 | Blzf1 | Rn.11987 |
| A_42_P759427 | 0.004598 | 0.715834 | Strn4 | Rn.137083 |
| A_42_P760101 | 0.046122 | 0.848176 | Pold2 | Rn.22989 |
| A_42_P765031 | 0.012946 | 0.734274 | Wash2 | Rn.15475 |
| A_42_P768799 | 0.027655 | 0.85367 | Rabac1 | Rn.25604 |
| A_42_P769290 | 0.00615 | 1.295166 |  | Rn.3440 |
| A_42_P769969 | 0.032863 | 0.769697 | Hmgn5 | Rn.25395 |
| A_42_P770171 | 0.019364 | 0.805662 | Isoc2b | Rn.1804 |
| A_42_P772136 | 0.024247 | 1.234929 | Fam60a | Rn.9154 |
| A_42_P773816 | 0.031167 | 0.814664 | Cd1d1 | Rn.11120 |
| A_42_P775256 | 0.013717 | 0.87482 | Ctf1 | Rn.10253 |
| A_42_P784931 | 0.00645 | 0.887115 | Dopey2 | Rn.52409 |
| A_42_P785075 | 0.04056 | 0.858568 | Actr6 | Rn.16356 |
| A_42_P785419 | 0.010814 | 0.852908 | Cdk2 | Rn.104460 |
| A_42_P785632 | 0.015728 | 1.079436 | Tbp | Rn.22712 |
| A_42_P792945 | 0.006647 | 1.660495 | Sds | Rn.9918 |
| A_42_P800008 | 0.018081 | 1.203958 | Slc4a3 | Rn.87739 |
| A_42_P803137 | 4.33E-05 | 1.927618 | Spint2 | Rn.3857 |
| A_42_P808294 | 0.027152 | 0.774914 | Ccndbp1 | Rn.162679 |
| A_42_P814081 | 0.00054 | 1.356707 | Med8 | Rn.102063 |
| A_42_P814410 | 0.00259 | 0.695067 | Gnl1 | Rn.35325 |
| A_42_P815235 | 0.028603 | 0.935483 | Prmt10 | Rn.199059 |
| A_42_P815372 | 0.000963 | 1.357714 | Snrpb2 | Rn.7712 |
| A_42_P815485 | 0.009694 | 1.091157 | RGD1306583 | Rn.15285 |
| A_42_P819194 | 0.015773 | 1.149546 | Dpep1 | Rn.6051 |
| A_42_P820847 | 0.020852 | 0.86435 | Clec14a | Rn.3962 |
| A_42_P828934 | 0.008782 | 0.826157 | Sf3b4 | Rn.116275 |
| A_42_P829863 | 0.006652 | 0.842022 | Galk1 | Rn.198917 |
| A_42_P831504 | 0.003497 | 1.158766 | Prpf38b | Rn.7210 |
| A_42_P835247 | 0.033565 | 0.811584 | Egln2 | Rn.43384 |
| A_42_P837568 | 0.035996 | 0.779658 | L1cam | Rn.10378 |
| A_42_P837865 | 0.035895 | 1.187581 | Nedd1 | Rn.23035 |
| A_42_P842367 | 0.021339 | 0.93063 | Lrpprc | Rn.92051 |
| A_42_P842842 | 0.005009 | 1.522257 |  | Rn.8938 |
| A_43_P10191 | 0.002895 | 1.346797 | Rnf7 | Rn.2768 |
| A_43_P10207 | 0.009399 | 0.799042 |  | Rn.1619 |
| A_43_P10227 | 3.94E-05 | 1.222832 | Abi1 | Rn.43675 |
| A_43_P10237 | 0.008591 | 0.875875 |  | Rn.8879 |
| A_43_P10254 | 0.035662 | 0.893202 | Gbf1 | Rn.61235 |
| A_43_P10264 | 0.041718 | 0.843143 | Sf3b2 | Rn.103341 |
| A_43_P10314 | 0.012458 | 0.796795 | Ppp1r12c | Rn.41379 |
| A_43_P10327 | 0.00346 | 0.800244 | RGD1359310 | Rn.4038 |
| A_43_P10371 | 0.009012 | 0.766492 | RGD1311422 | Rn.113761 |
| A_43_P10399 | 0.035751 | 0.779531 | Kctd9 | Rn.9317 |
| A_43_P10403 | 0.040222 | 0.831189 | Abhd12 | Rn.128955 |
| A_43_P10414 | 0.002747 | 0.798809 | Faf2 | Rn.162188 |
| A_43_P10428 | 0.000862 | 1.090171 |  | Rn.108232 |
| A_43_P10445 | 0.035169 | 0.750784 | Rad23a | Rn.105419 |
| A_43_P10468 | 0.029916 | 1.314593 | Myl9 | Rn.6870 |
| A_43_P10488 | 0.025682 | 0.875183 | R3hcc1 | Rn.30496 |
| A_43_P10534 | 0.03981 | 0.815055 | 2-Mar | Rn.145219 |
| A_43_P10587 | 0.038471 | 0.886456 | Ube2m | Rn.15135 |
| A_43_P10641 | 0.025235 | 0.808034 | Mgst2 | Rn.7854 |
| A_43_P10642 | 0.002135 | 0.821015 | Poldip3 | Rn.3893 |
| A_43_P10648 | 0.043185 | 1.175518 |  |  |
| A_43_P10751 | 0.036211 | 0.847287 | Ngrn | Rn.1278 |
| A_43_P10799 | 0.002755 | 1.361625 |  | Rn.115416 |
| A_43_P10807 | 0.031111 | 0.79575 |  |  |
| A_43_P10851 | 0.018539 | 1.211618 | Sh3glb1 | Rn.203013 |
| A_43_P10855 | 0.015287 | 1.652497 |  | Rn.16652 |
| A_43_P10888 | 0.018947 | 0.897871 | Sort1 | Rn.11286 |
| A_43_P10935 | 0.003652 | 1.262241 |  | Rn.95097 |
| A_43_P11075 | 0.031016 | 0.824933 | Mospd2 | Rn.15602 |
| A_43_P11098 | 0.013 | 1.144753 | Cdc42bpb | Rn.105815 |
| A_43_P11221 | 0.007134 | 0.865184 | Afg3l2 | Rn.8386 |
| A_43_P11329 | 0.026788 | 0.763497 | Zfp574 | Rn.15466 |
| A_43_P11448 | 0.001695 | 2.786139 | Cp | Rn.32777 |
| A_43_P11457 | 0.017039 | 0.835839 | Ace | Rn.10149 |
| A_43_P11495 | 0.009132 | 0.791629 | Prps2 | Rn.11320 |
| A_43_P11514 | 0.045864 | 0.90442 | Tpm4 | Rn.108199 |
| A_43_P11556 | 0.035937 | 0.829481 | Tgfbr1 | Rn.44402 |
| A_43_P11561 | 0.005416 | 0.812413 | Arnt | Rn.10520 |
| A_43_P11570 | 0.014065 | 0.819534 | Abcd3 | Rn.7024 |
| A_43_P11577 | 0.037429 | 0.734877 | Anxa3 | Rn.6589 |
| A_43_P11604 | 0.044918 | 0.781827 | Timp3 | Rn.119634 |
| A_43_P11606 | 0.009653 | 0.814376 | Adk | Rn.64165 |
| A_43_P11684 | 0.014553 | 1.286704 | Alpl | Rn.82764 |
| A_43_P11759 | 0.000113 | 4.313115 | Rps26 | Rn.135639 |
| A_43_P11924 | 0.00383 | 1.253984 | Calca | Rn.90085 |
| A_43_P12022 | 0.010785 | 1.137724 | Tpsab1 | Rn.10699 |
| A_43_P12127 | 0.016775 | 0.828334 | Ensa | Rn.27154 |
| A_43_P12133 | 0.002734 | 1.472305 | Epn2 | Rn.44273 |
| A_43_P12172 | 0.049548 | 0.86923 | Lgmn | Rn.206021 |
| A_43_P12189 | 0.017196 | 0.793021 | Tsnax | Rn.2315 |
| A_43_P12193 | 0.033078 | 0.775198 | Fbxl20 | Rn.18281 |
| A_43_P12194 | 0.033218 | 0.809782 | Aldh9a1 | Rn.98155 |
| A_43_P12212 | 0.000223 | 0.805262 | Cap1 | Rn.112585 |
| A_43_P12304 | 0.000223 | 0.710209 | Sgta | Rn.9655 |
| A_43_P12343 | 0.044764 | 0.905633 | Stx12 | Rn.54570 |
| A_43_P12404 | 0.024007 | 1.367048 | Evl | Rn.28912 |
| A_43_P12438 | 0.023811 | 0.88071 | Hmox2 | Rn.10241 |
| A_43_P12524 | 0.034942 | 0.865748 | Ssr3 | Rn.3264 |
| A_43_P12572 | 0.014789 | 1.190292 | Cd200 | Rn.7085 |
| A_43_P12757 | 0.027258 | 0.730045 | Gpr182 | Rn.87119 |
| A_43_P12758 | 0.007642 | 0.795636 | Msra | Rn.163306 |
| A_43_P12761 | 0.049174 | 0.8346 | Atp2b1 | Rn.7208 |
| A_43_P12800 | 0.030095 | 0.838063 | Gnpat | Rn.1739 |
| A_43_P12844 | 0.022159 | 0.832751 | Cox17 | Rn.19207 |
| A_43_P12860 | 0.011514 | 1.20691 | Cox5b | Rn.6686 |
| A_43_P12865 | 0.036032 | 1.215996 | Nudt4 | Rn.8426 |
| A_43_P12896 | 0.001779 | 1.152792 | Yme1l1 | Rn.8153 |
| A_43_P12992 | 0.03812 | 1.816358 | Kcnh2 | Rn.10970 |
| A_43_P13035 | 0.0038 | 1.168214 | Tra2b | Rn.8538 |
| A_43_P13040 | 0.007431 | 0.916575 | Rhoa | Rn.107401 |
| A_43_P13042 | 0.000741 | 0.862944 | Epn1 | Rn.30007 |
| A_43_P13047 | 0.009723 | 0.842568 | 2-Sep | Rn.98570 |
| A_43_P13057 | 0.009356 | 0.84263 | Baiap2 | Rn.95155 |
| A_43_P13059 | 0.036292 | 1.187071 | Uba3 | Rn.162761 |
| A_43_P13074 | 0.021517 | 0.782752 | Rab3d | Rn.9822 |
| A_43_P13091 | 0.049647 | 0.82879 | As3mt | Rn.95453 |
| A_43_P13244 | 0.001964 | 1.402798 | Rap1b | Rn.95071 |
| A_43_P13268 | 0.037231 | 1.095799 | Pmf31 | Rn.165271 |
| A_43_P13277 | 0.020818 | 1.099377 | Psmc3ip | Rn.144650 |
| A_43_P13294 | 0.039577 | 0.828227 | Tecr | Rn.107358 |
| A_43_P13335 | 0.001447 | 1.321594 | Foxe1 | Rn.26546 |
| A_43_P13447 | 0.044154 | 0.731322 |  | Rn.59193 |
| A_43_P13569 | 0.016979 | 0.635764 |  |  |
| A_43_P13622 | 0.005831 | 0.700965 | Pddc1 | Rn.99147 |
| A_43_P13711 | 0.049167 | 1.18408 | Tbc1d22b | Rn.16811 |
| A_43_P13744 | 0.000187 | 1.60537 |  |  |
| A_43_P13828 | 0.005275 | 0.802234 |  | Rn.23354 |
| A_43_P13862 | 0.049387 | 0.832743 |  | Rn.162348 |
| A_43_P14080 | 0.036158 | 0.872984 |  |  |
| A_43_P14247 | 0.005155 | 0.903985 | Rps3a | Rn.94935 |
| A_43_P14252 | 0.01143 | 1.587511 | Rab5c | Rn.228301 |
| A_43_P14285 | 0.007218 | 0.801566 | Elp2 | Rn.25007 |
| A_43_P14304 | 0.035118 | 0.788209 |  |  |
| A_43_P14367 | 0.027818 | 0.859736 |  |  |
| A_43_P14410 | 0.034761 | 0.896752 |  |  |
| A_43_P14483 | 0.042779 | 1.051161 | Bud31 | Rn.8172 |
| A_43_P14566 | 0.035374 | 1.285598 | Aatf | Rn.15500 |
| A_43_P14581 | 0.005508 | 1.192752 | Cbll1 | Rn.92651 |
| A_43_P14686 | 0.048374 | 0.887102 | Ubap2 | Rn.7333 |
| A_43_P14802 | 0.003913 | 0.893046 | Ncoa6 | Rn.9077 |
| A_43_P14884 | 0.042145 | 0.904138 | Akap8 | Rn.10248 |
| A_43_P14969 | 0.024406 | 0.6399 | Fbp1 | Rn.33703 |
| A_43_P15033 | 0.04702 | 0.910355 |  |  |
| A_43_P15116 | 0.031774 | 0.751108 |  | Rn.23080 |
| A_43_P15198 | 0.042128 | 1.267072 | Rap1gap2 | Rn.20246 |
| A_43_P15289 | 0.021161 | 0.790031 |  | Rn.93138 |
| A_43_P15312 | 0.037623 | 0.812671 | Gna12 | Rn.10497 |
| A_43_P15341 | 0.024078 | 0.832365 | Tdg | Rn.98685 |
| A_43_P15344 | 0.000301 | 1.357603 | Atp5g3 | Rn.2180 |
| A_43_P15367 | 0.029223 | 0.849533 | Lmbrd1 | Rn.201860 |
| A_43_P15494 | 0.00855 | 0.852642 | Cebpa | Rn.204833 |
| A_43_P15524 | 0.046536 | 1.203514 | Ccnd3 | Rn.3483 |
| A_43_P15527 | 0.033815 | 0.802923 | Hmgb1 | Rn.144565 |
| A_43_P15633 | 0.032193 | 0.848547 | Pkn2 | Rn.30325 |
| A_43_P15648 | 0.00074 | 0.78152 | Foxo1 | Rn.116108 |
| A_43_P15665 | 0.005934 | 0.796398 | Bckdha | Rn.49145 |
| A_43_P15751 | 0.01303 | 1.313437 | Grm2 | Rn.9681 |
| A_43_P15788 | 0.026348 | 0.719399 | Kifc3 | Rn.14753 |
| A_43_P15898 | 0.007166 | 0.604232 | Bmp1 | Rn.9305 |
| A_43_P15998 | 0.025207 | 1.255307 | Ntn3 | Rn.95949 |
| A_43_P16029 | 0.044247 | 0.623766 | LOC100361261 | Rn.132115 |
| A_43_P16070 | 0.011772 | 0.721861 | Axl | Rn.161805 |
| A_43_P16362 | 0.015054 | 0.617272 |  |  |
| A_43_P16415 | 0.002654 | 0.833389 | Ndufv1 | Rn.3159 |
| A_43_P16445 | 0.004914 | 0.727104 | F8a1 | Rn.41311 |
| A_43_P16469 | 0.034642 | 0.779003 | Chmp1a | Rn.6239 |
| A_43_P16488 | 0.000578 | 1.267965 | Chrac1 | Rn.17653 |
| A_43_P16496 | 0.004733 | 0.884942 |  | Rn.2459 |
| A_43_P16537 | 0.000911 | 0.778009 | Pcnp | Rn.202642 |
| A_43_P16552 | 0.028666 | 0.860017 | Git2 | Rn.77521 |
| A_43_P16583 | 0.031647 | 0.819357 | Snx2 | Rn.106977 |
| A_43_P16643 | 0.00897 | 0.823924 | Abhd4 | Rn.1831 |
| A_43_P16685 | 0.033744 | 0.722039 | Abca3 | Rn.38406 |
| A_43_P16741 | 0.041264 | 1.107984 | Vta1 | Rn.1896 |
| A_43_P16755 | 0.0054 | 0.836501 | Alg5 | Rn.155539 |
| A_43_P16767 | 0.043441 | 1.256156 | Igfbp4 | Rn.160666 |
| A_43_P16884 | 0.034429 | 0.831821 | Acin1 | Rn.97147 |
| A_43_P16919 | 0.015401 | 0.873001 | Rnf2 | Rn.19719 |
| A_43_P16961 | 0.04814 | 0.852104 | Msl3 | Rn.7258 |
| A_43_P16964 | 0.036118 | 1.158314 | Ppfibp1 | Rn.22871 |
| A_43_P16992 | 0.007915 | 0.790101 | Auh | Rn.50 |
| A_43_P17007 | 0.038711 | 0.703952 | Fbxo31 | Rn.163412 |
| A_43_P17027 | 0.015741 | 0.770918 | Lonp2 | Rn.103067 |
| A_43_P17033 | 5.27E-05 | 0.857025 | Pum2 | Rn.8622 |
| A_43_P17065 | 1.89E-05 | 1.608376 | Helz | Rn.19942 |
| A_43_P17068 | 0.003492 | 0.880309 | Fam48a | Rn.45268 |
| A_43_P17140 | 0.002265 | 0.828273 | Flii | Rn.144698 |
| A_43_P17141 | 0.019837 | 0.860023 | Smyd2 | Rn.7052 |
| A_43_P17180 | 0.013836 | 0.860048 | mrpl9 | Rn.763 |
| A_43_P17186 | 0.041867 | 0.723898 | Gatsl3 | Rn.155378 |
| A_43_P17230 | 0.038691 | 1.172448 | Iws1 | Rn.206133 |
| A_43_P17233 | 0.006828 | 0.855858 | Sars2 | Rn.154470 |
| A_43_P17238 | 0.046648 | 0.779517 | Rnd2 | Rn.22162 |
| A_43_P17264 | 0.030395 | 0.797271 | Pbrm1 | Rn.19314 |
| A_43_P17266 | 0.024357 | 0.804328 | Naa15 | Rn.12587 |
| A_43_P17326 | 0.019845 | 1.518547 | Mov10 | Rn.3508 |
| A_43_P17341 | 0.002859 | 0.848437 | Slu7 | Rn.137183 |
| A_43_P17382 | 0.003185 | 0.763159 | Lrrc16a | Rn.40387 |
| A_43_P17387 | 0.039361 | 0.835812 |  | Rn.34400 |
| A_43_P17392 | 0.037951 | 0.845575 | Arhgap21 | Rn.162259 |
| A_43_P17401 | 0.018951 | 1.213066 | Ppme1 | Rn.137659 |
| A_43_P17406 | 0.018215 | 0.808353 | Vps16 | Rn.14968 |
| A_43_P17439 | 0.0134 | 0.854859 | Gtf3c2 | Rn.22450 |
| A_43_P17443 | 0.020649 | 0.790623 | Rin2 | Rn.34193 |
| A_43_P17468 | 0.032616 | 1.189895 | Med21 | Rn.6795 |
| A_43_P17491 | 0.032106 | 0.721818 | Mmp15 | Rn.165433 |
| A_43_P17527 | 0.013083 | 0.898917 | Trnt1 | Rn.159746 |
| A_43_P17577 | 0.000229 | 0.714684 | Vps11 | Rn.2637 |
| A_43_P17580 | 0.041809 | 0.781377 | Pck2 | Rn.35508 |
| A_43_P17608 | 0.030276 | 1.199918 | Smek2 | Rn.35621 |
| A_43_P17632 | 0.03613 | 0.862733 | Camta2 | Rn.13745 |
| A_43_P17667 | 0.02514 | 0.802455 | Txndc11 | Rn.54595 |
| A_43_P17703 | 0.045364 | 1.125596 | Dph5 | Rn.12336 |
| A_43_P17761 | 0.030911 | 0.904092 | Etaa1 | Rn.121635 |
| A_43_P17812 | 0.040168 | 1.156007 | Wdr47 | Rn.18637 |
| A_43_P17824 | 0.047184 | 0.769245 | Nphp1 | Rn.32745 |
| A_43_P17977 | 0.000934 | 0.774467 | Sec24c | Rn.122667 |
| A_43_P17979 | 0.005337 | 0.851001 | LOC100363361 | Rn.139466 |
| A_43_P18029 | 0.003135 | 0.846847 | Trip4 | Rn.102294 |
| A_43_P18031 | 0.005971 | 0.850736 | Tm9sf1 | Rn.12071 |
| A_43_P18039 | 0.000672 | 1.401101 | Tab2 | Rn.228335 |
| A_43_P18054 | 0.006051 | 0.81147 | Pigs | Rn.59136 |
| A_43_P18086 | 0.015469 | 1.150291 | Dnaaf2 | Rn.85355 |
| A_43_P18111 | 0.024071 | 0.837467 | Exd2 | Rn.102 |
| A_43_P18178 | 0.023297 | 0.877183 | Jagn1 | Rn.203687 |
| A_43_P18203 | 0.047015 | 1.119394 | Sf3a3 | Rn.16732 |
| A_43_P18210 | 0.01321 | 0.822576 |  | Rn.154631 |
| A_43_P18238 | 0.000726 | 0.756219 | Arfip2 | Rn.73878 |
| A_43_P18263 | 0.008787 | 0.815973 | Celf1 | Rn.22432 |
| A_43_P18268 | 0.037195 | 0.789672 |  | Rn.16368 |
| A_43_P18282 | 0.018644 | 0.792632 | RGD1311345 | Rn.53930 |
| A_43_P18294 | 0.006236 | 0.791966 | Jarid1a | Rn.224608 |
| A_43_P18380 | 0.029744 | 0.807749 | LOC100360606 | Rn.202182 |
| A_43_P18385 | 0.031862 | 0.87793 | Sqrdl | Rn.1416 |
| A_43_P18469 | 0.010969 | 0.727514 | Sytl3 | Rn.136598 |
| A_43_P18485 | 0.03604 | 0.83221 | B4galt7 | Rn.41210 |
| A_43_P18497 | 0.00441 | 0.805806 | Mfap3 | Rn.163119 |
| A_43_P18579 | 0.000497 | 0.817718 | Fbxw2 | Rn.103250 |
| A_43_P18610 | 0.029413 | 0.714661 | RGD1563235 | Rn.46749 |
| A_43_P18684 | 0.028205 | 0.839394 | Ric8a | Rn.94796 |
| A_43_P18687 | 0.0042 | 0.724165 | Snx25 | Rn.16962 |
| A_43_P18690 | 0.000643 | 0.83096 | Pds5a | Rn.24482 |
| A_43_P18721 | 0.014088 | 0.855073 | Myef2 | Rn.1269 |
| A_43_P18736 | 0.015031 | 0.750808 | Mynn | Rn.162582 |
| A_43_P18781 | 0.01859 | 0.832956 | Lpin3 | Rn.145057 |
| A_43_P18787 | 0.007319 | 0.847348 | Ncoa6 | Rn.9077 |
| A_43_P18805 | 0.000927 | 0.789546 | R3hdm2 | Rn.203556 |
| A_43_P18815 | 0.006564 | 0.765403 | Stag1 | Rn.11715 |
| A_43_P18817 | 0.009217 | 0.772473 | Ift52 | Rn.36626 |
| A_43_P18861 | 0.027868 | 0.816419 | RGD1307100 | Rn.162572 |
| A_43_P18907 | 0.021076 | 0.759631 | Lrig1 | Rn.54266 |
| A_43_P18993 | 0.002472 | 0.811375 |  | Rn.48387 |
| A_43_P19094 | 0.046265 | 0.764271 | Elovl7 | Rn.105650 |
| A_43_P19145 | 0.018905 | 0.757738 | RGD1309676 | Rn.13520 |
| A_43_P19148 | 0.011189 | 0.796165 | Sos1 | Rn.91844 |
| A_43_P19169 | 0.031208 | 0.808041 | Dcbld1 | Rn.105687 |
| A_43_P19181 | 0.000808 | 0.837791 |  | Rn.32535 |
| A_43_P19227 | 0.010399 | 0.805554 | RGD1561537 | Rn.12539 |
| A_43_P19229 | 0.005184 | 0.799489 | Mocs1 | Rn.33171 |
| A_43_P19276 | 0.037797 | 0.849159 | Cep68 | Rn.105766 |
| A_43_P19318 | 0.004575 | 0.869082 | Cdk9 | Rn.98228 |
| A_43_P19328 | 0.039016 | 0.889084 | Ttc19 | Rn.216288 |
| A_43_P19361 | 0.020989 | 0.823604 | Abhd5 | Rn.12459 |
| A_43_P19499 | 0.017598 | 0.742364 | Gzf1 | Rn.40179 |
| A_43_P19519 | 0.040336 | 1.117069 | Vcl | Rn.164613 |
| A_43_P19526 | 0.003695 | 0.7589 | Rbm12 | Rn.7119 |
| A_43_P19560 | 0.011398 | 0.824476 | Brf1 | Rn.205393 |
| A_43_P19606 | 0.023427 | 0.80627 | Slc12a7 | Rn.64199 |
| A_43_P19626 | 0.024832 | 1.701664 | Sncaip | Rn.199043 |
| A_43_P19656 | 0.004759 | 0.751609 | Mgat4b | Rn.24701 |
| A_43_P19673 | 0.023712 | 0.836122 | Lrba | Rn.2120 |
| A_43_P19709 | 0.011548 | 0.8017 | Scrib | Rn.138068 |
| A_43_P19762 | 0.042165 | 0.77579 |  | Rn.9000 |
| A_43_P19830 | 0.025433 | 1.324691 | Ythdf2 | Rn.21737 |
| A_43_P19850 | 0.011991 | 0.724472 |  |  |
| A_43_P19892 | 0.008849 | 0.854404 | Aph1a | Rn.22786 |
| A_43_P20053 | 0.027953 | 0.74339 | Fut8 | Rn.1717 |
| A_43_P20057 | 0.000126 | 1.353997 |  | Rn.55605 |
| A_43_P20118 | 0.013248 | 0.929556 | Suclg2 | Rn.4098 |
| A_43_P20142 | 0.035988 | 0.77214 | Atp6v0a2 | Rn.204067 |
| A_43_P20191 | 0.0116 | 0.768624 | Pgm3 | Rn.24236 |
| A_43_P20205 | 0.047087 | 0.654985 | Ldb2 | Rn.94720 |
| A_43_P20319 | 0.018926 | 0.809048 | Dbf4 | Rn.48553 |
| A_43_P20329 | 0.001516 | 0.846869 | Pum1 | Rn.44196 |
| A_43_P20363 | 0.000582 | 0.83089 | Sf4 | Rn.67035 |
| A_43_P20397 | 0.041435 | 1.147043 |  | Rn.20718 |
| A_43_P20408 | 0.024585 | 0.86144 | Pepd | Rn.101639 |
| A_43_P20412 | 0.031549 | 0.842603 | Arsg | Rn.221856 |
| A_43_P20422 | 0.036013 | 0.718846 | Cdc14a | Rn.147123 |
| A_43_P20474 | 0.047711 | 0.73613 |  | Rn.226310 |
| A_43_P20478 | 0.036305 | 0.809229 | Slc37a1 | Rn.138019 |
| A_43_P20534 | 0.002564 | 0.883582 | Fbxo22 | Rn.4284 |
| A_43_P20568 | 0.025454 | 0.882718 | Mettl13 | Rn.18927 |
| A_43_P20602 | 0.006793 | 0.82806 |  | Rn.50103 |
| A_43_P20631 | 0.001299 | 0.759414 | Rilpl1 | Rn.62627 |
| A_43_P20634 | 0.011438 | 0.886569 |  | Rn.137956 |
| A_43_P20646 | 0.028795 | 0.74791 | Mpzl2 | Rn.8193 |
| A_43_P20647 | 0.0171 | 0.81182 | Gatad2b | Rn.133230 |
| A_43_P20729 | 0.03441 | 0.838139 | Prrc2a | Rn.34262 |
| A_43_P20922 | 0.044756 | 0.835202 | Tbc1d9 | Rn.19710 |
| A_43_P20943 | 0.005147 | 0.785225 | LOC100362678 | Rn.15650 |
| A_43_P20951 | 0.039405 | 0.810383 | Anapc1 | Rn.8 |
| A_43_P20972 | 0.019594 | 0.858778 | Lrrc8b | Rn.170566 |
| A_43_P21173 | 0.006077 | 0.827473 | Fbxl14 | Rn.225319 |
| A_43_P21247 | 0.004444 | 0.729956 | Traf3ip2 | Rn.9031 |
| A_43_P21280 | 0.039713 | 0.861253 | Agap1 | Rn.25493 |
| A_43_P21347 | 0.021969 | 0.826931 | Capn7 | Rn.104104 |
| A_43_P21383 | 0.017706 | 1.558413 |  |  |
| A_43_P21538 | 0.004342 | 0.777715 | Taok2 | Rn.9575 |
| A_43_P21557 | 0.025727 | 0.746502 | Ctnnbl1 | Rn.98091 |
| A_43_P21650 | 0.016638 | 0.888393 | Adpgk | Rn.66171 |
| A_43_P21658 | 0.026481 | 0.879769 | Gpc4 | Rn.19945 |
| A_43_P21715 | 0.04549 | 0.822225 | Rwdd2b | Rn.206833 |
| A_43_P21719 | 0.048633 | 0.778525 | Mpzl2 | Rn.8193 |
| A_43_P21741 | 0.02144 | 0.852529 | RGD1307830 | Rn.211806 |
| A_43_P21762 | 0.037046 | 0.761634 | Slc25a30 | Rn.19440 |
| A_43_P21776 | 0.015204 | 0.748367 | Rfc5 | Rn.105670 |
| A_43_P21855 | 0.020507 | 0.822303 | Wipi2 | Rn.163194 |
| A_43_P21945 | 0.01428 | 0.659068 | Ppl | Rn.25259 |
| A_43_P22037 | 0.009024 | 1.406661 | Sall3 | Rn.99341 |
| A_43_P22124 | 0.032185 | 0.859889 | Dnajc7 | Rn.92155 |
| A_43_P22126 | 4.83E-05 | 0.826876 | Ptcd1 | Rn.105071 |
| A_43_P22189 | 0.002789 | 0.86713 |  | Rn.201492 |
| A_43_P22210 | 0.015907 | 0.780678 | Zfp644 | Rn.8773 |
| A_43_P22276 | 0.030274 | 0.839907 | LOC100362283 | Rn.158990 |
| A_43_P22351 | 0.001216 | 0.786503 | Slc46a3 | Rn.16498 |
| A_43_P22513 | 0.027641 | 0.860827 | Cyb561d1 | Rn.17547 |
| A_43_P22555 | 0.045664 | 0.741022 | Fam189a2 | Rn.8399 |
| A_43_P22661 | 0.016358 | 0.768961 | LOC499602 | Rn.12999 |
| A_43_P22712 | 0.024708 | 0.775211 | Dmwd | Rn.138418 |
| A_43_P22777 | 0.003083 | 0.884503 | Cyfip1 | Rn.143776 |
| A_43_P22864 | 0.016986 | 0.874136 | Rad54l | Rn.202025 |
| A_43_P22983 | 0.04097 | 0.744196 | Pnldc1 | Rn.136599 |
| A_43_P23023 | 0.009884 | 0.883888 | RGD1309543 | Rn.16324 |
| A_43_P23025 | 0.038996 | 0.856708 | Zfp259 | Rn.102011 |
| A_43_P23029 | 0.009425 | 0.824908 | Fars2 | Rn.8968 |
| A_43_P23093 | 0.007825 | 0.747677 |  |  |
| A_43_P23108 | 0.029739 | 0.887464 | Mcm7 | Rn.113 |
| A_43_P23116 | 0.020969 | 0.804892 | Pdss2 | Rn.20063 |
| A_43_P23126 | 0.03606 | 0.821553 | Klf11 | Rn.18589 |
| A_43_P23165 | 0.024406 | 0.783293 | Ophn1 | Rn.51010 |
| A_43_P23197 | 0.004242 | 1.540334 | MGC95208 | Rn.19101 |
| A_43_P23459 | 0.012005 | 1.237779 |  |  |
| A_44_P1000203 | 0.022989 | 0.895714 | Mrpl44 | Rn.3298 |
| A_44_P1000383 | 0.000251 | 2.01656 | Eprs | Rn.21240 |
| A_44_P1001301 | 0.01517 | 0.890419 | Eef1g | Rn.43300 |
| A_44_P100139 | 0.036536 | 1.46329 | Olr4 | Rn.142005 |
| A_44_P1001820 | 0.003574 | 1.288312 | Slc22a5 | Rn.8844 |
| A_44_P1002141 | 0.034199 | 0.814003 | Tsen34 | Rn.1591 |
| A_44_P1003072 | 0.013558 | 1.229621 | Dusp11 | Rn.24649 |
| A_44_P1003378 | 0.031351 | 1.198195 |  | Rn.106964 |
| A_44_P1003517 | 0.011065 | 1.216446 | Psmd6 | Rn.103875 |
| A_44_P1003794 | 0.001627 | 1.750213 | Nr3c1 | Rn.90070 |
| A_44_P1004737 | 0.021217 | 1.503635 |  | Rn.178435 |
| A_44_P1005450 | 0.027354 | 1.423157 | Ktn1 | Rn.66508 |
| A_44_P100557 | 0.026979 | 0.846945 | Zfp219 | Rn.206680 |
| A_44_P1006058 | 0.025988 | 1.688103 | Ctsk | Rn.5598 |
| A_44_P100618 | 0.002555 | 1.479782 | LOC100365875 | |
| A_44_P100636 | 0.023268 | 1.716895 |  |  |
| A_44_P100659 | 0.025389 | 1.830558 | Rnf224 |  |
| A_44_P100660 | 0.010518 | 1.521464 | LOC100362391 | |
| A_44_P1006655 | 0.002484 | 0.894734 | Rps27 | Rn.153914 |
| A_44_P1006988 | 0.037212 | 0.78598 | Zfp318 | Rn.28520 |
| A_44_P1007008 | 0.020495 | 1.492643 | Ube2l6 | Rn.8609 |
| A_44_P1008094 | 0.000354 | 1.634266 | Exosc7 | Rn.45886 |
| A_44_P1008120 | 0.019518 | 0.819947 | Nans | Rn.17006 |
| A_44_P100847 | 0.03036 | 0.749466 | Ttc7 | Rn.16768 |
| A_44_P1009892 | 0.000107 | 1.86816 | Pja2 | Rn.18446 |
| A_44_P1010653 | 0.002037 | 1.106538 | Bcl7b | Rn.37635 |
| A_44_P1011253 | 0.010706 | 1.446572 | Nars | Rn.48209 |
| A_44_P1011373 | 0.001109 | 1.29851 | Camsap1 | Rn.203213 |
| A_44_P1012591 | 2.73E-05 | 1.334921 | Osbpl1a | Rn.178664 |
| A_44_P1012690 | 0.026937 | 0.875742 | Snrnp70 | Rn.203159 |
| A_44_P101375 | 0.000309 | 0.758895 | Vps16 | Rn.14968 |
| A_44_P1014005 | 0.002114 | 1.298963 | Cdc42se1 | Rn.111901 |
| A_44_P1014018 | 0.023168 | 1.361604 | Mex3d | Rn.162915 |
| A_44_P1014263 | 0.011099 | 1.163139 | Hypk | Rn.17258 |
| A_44_P1014955 | 0.004599 | 1.323977 | Psph | Rn.8734 |
| A_44_P1015355 | 0.003749 | 2.152432 | Rnmt | Rn.20871 |
| A_44_P1015606 | 0.001159 | 1.648977 | Sfswap | Rn.12338 |
| A_44_P101684 | 0.001638 | 1.445965 |  | Rn.162277 |
| A_44_P1017125 | 0.030237 | 1.501855 | Sav1 | Rn.14087 |
| A_44_P1017763 | 4.67E-05 | 0.877209 | Rpp21 | Rn.3720 |
| A_44_P1017820 | 0.007716 | 1.364519 | Leo1 | Rn.43195 |
| A_44_P1018067 | 0.038077 | 0.832025 | Setd3 | Rn.7951 |
| A_44_P1018662 | 0.024856 | 1.125215 | Coa5 | Rn.3412 |
| A_44_P1019639 | 0.049503 | 1.374609 | Gnai3 | Rn.4368 |
| A_44_P1020428 | 0.035229 | 0.789097 | Nes | Rn.9701 |
| A_44_P1020448 | 0.041714 | 0.825482 | Prr12 | Rn.222647 |
| A_44_P1021165 | 0.024482 | 0.85317 | Trim41 | Rn.79871 |
| A_44_P1023611 | 0.022931 | 0.847267 | Ino80b | Rn.106816 |
| A_44_P1024165 | 0.043259 | 1.162119 |  | Rn.101 |
| A_44_P1024567 | 0.001409 | 0.718208 | Mxd4 | Rn.3279 |
| A_44_P1024836 | 0.000163 | 2.581417 | Utx | Rn.29021 |
| A_44_P1025310 | 0.012418 | 0.917301 | Psmb3 | Rn.94551 |
| A_44_P1025750 | 6.13E-05 | 3.031491 | Rchy1 | Rn.2007 |
| A_44_P1025802 | 0.026829 | 0.876598 | Metap1 | Rn.6799 |
| A_44_P1025812 | 0.041647 | 0.80196 | LOC689074 | Rn.24684 |
| A_44_P102607 | 0.001011 | 0.853248 | Sec31a | Rn.6322 |
| A_44_P1026233 | 0.006284 | 0.871454 | Tmem203 | Rn.23564 |
| A_44_P1026598 | 0.003538 | 0.865084 |  | Rn.12762 |
| A_44_P1027005 | 0.037121 | 1.414627 |  | Rn.2892 |
| A_44_P102755 | 0.016775 | 1.419636 | Vangl2 | Rn.198958 |
| A_44_P1027574 | 0.000502 | 1.217759 |  | Rn.199181 |
| A_44_P1028282 | 0.012682 | 1.138348 | Mrpl51 | Rn.154504 |
| A_44_P102845 | 0.004039 | 0.850453 | Ube2v1 | Rn.220168 |
| A_44_P1028765 | 0.01151 | 0.872575 | R3hdm2 | Rn.203556 |
| A_44_P1029492 | 0.01234 | 1.685024 | Snapc1 | Rn.18361 |
| A_44_P102962 | 0.002278 | 1.474525 |  |  |
| A_44_P1030594 | 0.005388 | 1.388944 | Cebpg | Rn.10332 |
| A_44_P1030727 | 0.014988 | 1.223718 |  | Rn.3390 |
| A_44_P1030766 | 0.015902 | 1.212544 | Pcf11 | Rn.27595 |
| A_44_P1031524 | 6.54E-05 | 1.640029 | Rpl34 | Rn.2028 |
| A_44_P1031638 | 0.014763 | 0.834033 | Nsrp1 | Rn.145937 |
| A_44_P1031737 | 0.004613 | 1.8252 | Pecr | Rn.163081 |
| A_44_P1032051 | 0.020365 | 0.91035 | LOC687565 | Rn.101864 |
| A_44_P1032305 | 0.005167 | 1.370674 | Fert2 | Rn.102629 |
| A_44_P1033595 | 0.01621 | 0.794849 | Atl3 | Rn.202359 |
| A_44_P1033819 | 0.047923 | 1.127286 | Gtf2h2 | Rn.144260 |
| A_44_P1034201 | 0.044743 | 0.872007 | Lamb3 | Rn.49634 |
| A_44_P1034209 | 0.002194 | 0.77543 | Lamb3 | Rn.49634 |
| A_44_P1034417 | 0.04722 | 0.897996 | Sec16a | Rn.199218 |
| A_44_P1034522 | 0.043822 | 0.892887 | Impad1 | Rn.228227 |
| A_44_P1034613 | 0.011987 | 1.206038 | LOC619574 | Rn.162222 |
| A_44_P103472 | 0.002902 | 0.85544 | LOC503192 | Rn.69744 |
| A_44_P1035071 | 0.034955 | 1.248341 | Nmt2 | Rn.162255 |
| A_44_P1035926 | 0.020826 | 1.258609 | Ercc4 | Rn.195555 |
| A_44_P1036480 | 0.043204 | 1.335371 | Prrc2c | Rn.206334 |
| A_44_P1036481 | 0.038264 | 1.346211 | Prrc2c | Rn.206334 |
| A_44_P1036875 | 0.011744 | 0.902294 | Fam193a | Rn.7798 |
| A_44_P1037131 | 0.009276 | 0.853305 | Actr1a | Rn.32312 |
| A_44_P1037275 | 0.000947 | 1.487423 | Vkorc1l1 | Rn.163208 |
| A_44_P1037493 | 0.010226 | 0.905494 | Tssc4 | Rn.18732 |
| A_44_P1037796 | 0.012216 | 0.897662 | Arfgap1 | Rn.11219 |
| A_44_P1037817 | 0.003809 | 1.130897 | Lman2 | Rn.47037 |
| A_44_P1037886 | 0.033468 | 0.907096 | Sap18 | Rn.74373 |
| A_44_P1038266 | 0.002077 | 0.845678 | Fkbp15 | Rn.162820 |
| A_44_P1038392 | 0.003863 | 1.204097 | Cox7a2 | Rn.1745 |
| A_44_P1038946 | 0.006068 | 1.15642 | Csde1 | Rn.3562 |
| A_44_P1039184 | 0.047648 | 1.214181 | Zfand5 | Rn.4050 |
| A_44_P1039435 | 0.034076 | 1.166357 | Lasp1 | Rn.94195 |
| A_44_P1039760 | 0.017058 | 0.846656 | Dynlt3 | Rn.106023 |
| A_44_P1039927 | 0.012379 | 1.440937 | Eepd1 | Rn.110716 |
| A_44_P1040434 | 0.049091 | 1.344838 |  | Rn.205491 |
| A_44_P1041179 | 0.002326 | 1.36981 | Ep400 | Rn.76053 |
| A_44_P1042125 | 0.04488 | 1.135224 | Psmg4 | Rn.98733 |
| A_44_P1042294 | 0.002034 | 1.144406 | Pbrm1 | Rn.19314 |
| A_44_P1042535 | 0.005571 | 1.333691 |  | Rn.98667 |
| A_44_P1042901 | 0.000686 | 1.102243 | Pum2 | Rn.8622 |
| A_44_P1042940 | 0.000729 | 1.492427 |  |  |
| A_44_P104295 | 0.037142 | 1.190322 |  | Rn.17097 |
| A_44_P1043145 | 0.036233 | 0.849987 | Ctnna1 | Rn.103790 |
| A_44_P1043457 | 0.023468 | 1.130176 | Lgals8 | Rn.10069 |
| A_44_P1044270 | 0.037196 | 0.77537 | Rpain | Rn.43464 |
| A_44_P1044517 | 0.037671 | 1.852975 | N4bp1 | Rn.137980 |
| A_44_P104483 | 0.001302 | 1.927907 | Ccdc64 | Rn.166161 |
| A_44_P1044830 | 3.41E-05 | 2.279202 | Lactb | Rn.73451 |
| A_44_P1045469 | 0.048229 | 1.348939 |  | Rn.199571 |
| A_44_P1045759 | 0.013022 | 1.16582 | RGD1309104 | Rn.228438 |
| A_44_P104590 | 0.008796 | 0.810808 | Cdc42bpb | Rn.105815 |
| A_44_P1045950 | 0.027727 | 0.788544 | Pcsk1n | Rn.25745 |
| A_44_P1046471 | 0.002121 | 1.265137 | Rpap3 | Rn.49051 |
| A_44_P1046697 | 0.017135 | 0.829357 | Ctdsp1 | Rn.163070 |
| A_44_P1047375 | 0.005559 | 1.17105 | Rnf10 | Rn.11656 |
| A_44_P1047628 | 0.045916 | 0.865495 | Pars2 | Rn.205937 |
| A_44_P1048611 | 0.001274 | 1.275701 | Llph | Rn.205328 |
| A_44_P1049638 | 0.012622 | 1.229507 | Uqcrfs1 | Rn.2603 |
| A_44_P1050333 | 0.00329 | 1.755432 | Nlk | Rn.113514 |
| A_44_P1051685 | 0.038217 | 0.908075 | Cxxc1 | Rn.86349 |
| A_44_P1052324 | 0.00669 | 2.43335 | Lamp3 | Rn.19784 |
| A_44_P105249 | 0.036985 | 1.184797 |  |  |
| A_44_P1052504 | 0.00016 | 2.214778 | Rhot2 | Rn.8882 |
| A_44_P1052772 | 0.039039 | 0.843338 |  | Rn.49121 |
| A_44_P1052868 | 0.000443 | 1.823129 | Syne1 | Rn.226002 |
| A_44_P1052976 | 0.015111 | 0.902424 | Ppp6r2 | Rn.135331 |
| A_44_P1053365 | 0.041831 | 0.899391 | Rsrc2 | Rn.79033 |
| A_44_P1053383 | 0.014281 | 1.330903 | Rab7a | Rn.1425 |
| A_44_P1053645 | 0.004179 | 0.875858 |  | Rn.16562 |
| A_44_P105377 | 0.023141 | 0.785228 | Cenpb | Rn.33056 |
| A_44_P105497 | 0.001686 | 1.185586 |  |  |
| A_44_P105560 | 0.0143 | 1.620948 |  |  |
| A_44_P105566 | 0.000875 | 1.438631 |  |  |
| A_44_P1055681 | 0.000388 | 0.832312 | Lonp1 | Rn.146805 |
| A_44_P105680 | 0.015534 | 0.831315 | Zfp496 | Rn.13860 |
| A_44_P1057137 | 0.000201 | 1.26424 | Phf1 | Rn.24149 |
| A_44_P1057211 | 0.013548 | 0.83549 | Fzr1 | Rn.12924 |
| A_44_P1057331 | 0.02957 | 1.226325 | Stk40 | Rn.4052 |
| A_44_P1057602 | 0.001399 | 1.285618 | Ercc6 | Rn.19370 |
| A_44_P1058112 | 0.039025 | 1.158889 | Yars2 | Rn.163187 |
| A_44_P1059322 | 0.000145 | 4.297409 | Whamm | Rn.198506 |
| A_44_P1059422 | 0.043457 | 0.906443 | Actr2 | Rn.102249 |
| A_44_P1059623 | 0.018673 | 1.147514 | Scaf11 | Rn.199124 |
| A_44_P1059827 | 0.000293 | 0.862467 |  | Rn.8959 |
| A_44_P1060108 | 0.009425 | 1.431595 | Tmsb10 | Rn.5983 |
| A_44_P1060345 | 0.001201 | 1.510829 | Cpox | Rn.19581 |
| A_44_P106248 | 0.000404 | 1.884059 |  |  |
| A_44_P106541 | 0.006147 | 0.859484 | Alg5 | Rn.155539 |
| A_44_P106954 | 0.002463 | 0.79735 | Arf1 | Rn.93735 |
| A_44_P1070020 | 0.032902 | 1.365151 |  |  |
| A_44_P1070980 | 0.001161 | 1.561293 |  | Rn.25307 |
| A_44_P1071344 | 0.011181 | 0.873677 | Atxn2l | Rn.220440 |
| A_44_P107160 | 0.037969 | 0.931356 | Gps1 | Rn.16873 |
| A_44_P107219 | 0.000257 | 0.824914 | Gltscr2 | Rn.8075 |
| A_44_P107285 | 0.020929 | 0.928036 | Psmb6 | Rn.8118 |
| A_44_P107495 | 0.016372 | 0.782344 | Epb4.1l2 | Rn.1362 |
| A_44_P107821 | 0.02531 | 1.337442 | Clic2 | Rn.103254 |
| A_44_P107996 | 0.004604 | 1.615044 | LOC367050 | |
| A_44_P108028 | 0.019054 | 1.185728 |  |  |
| A_44_P108086 | 0.007604 | 1.1667 | Rras2 | Rn.3271 |
| A_44_P108102 | 0.004812 | 1.671848 |  |  |
| A_44_P108296 | 0.002344 | 1.417372 |  | Rn.219993 |
| A_44_P108982 | 0.031787 | 0.891194 | Sucla2 | Rn.62159 |
| A_44_P109271 | 0.021791 | 1.164948 |  | Rn.1574 |
| A_44_P109680 | 0.021524 | 0.853471 | Zfp105 | Rn.12040 |
| A_44_P109791 | 0.011942 | 0.827209 | Pja2 | Rn.18446 |
| A_44_P109887 | 0.034675 | 0.850711 | Ccni | Rn.6670 |
| A_44_P110044 | 0.046542 | 0.715831 | Mboat1 | Rn.14994 |
| A_44_P110098 | 0.021529 | 0.712227 | Ppip5k1 | Rn.46305 |
| A_44_P110109 | 0.042597 | 0.859037 | Cst3 | Rn.106351 |
| A_44_P110110 | 0.003729 | 0.822474 | Cst3 | Rn.106351 |
| A_44_P110660 | 0.010194 | 1.336282 |  | Rn.17706 |
| A_44_P110918 | 0.007327 | 1.090001 | Copb2 | Rn.37178 |
| A_44_P111347 | 2.13E-05 | 1.597171 |  | Rn.72721 |
| A_44_P111430 | 0.027049 | 0.906349 |  | Rn.22450 |
| A_44_P111881 | 0.014182 | 0.746384 | Ptpn13 | Rn.35546 |
| A_44_P112061 | 0.007857 | 0.884417 | Mll4 | Rn.57787 |
| A_44_P112098 | 0.001104 | 2.665014 |  |  |
| A_44_P112237 | 0.000208 | 2.90428 |  |  |
| A_44_P112570 | 0.005857 | 1.508257 |  |  |
| A_44_P112670 | 0.005904 | 1.176363 |  | Rn.92856 |
| A_44_P113084 | 3.07E-05 | 1.506544 |  | Rn.98343 |
| A_44_P113088 | 0.005366 | 0.835031 | Slc34a2 | Rn.16933 |
| A_44_P113360 | 0.000166 | 2.382931 |  | Rn.112601 |
| A_44_P113588 | 0.002297 | 1.198777 |  | Rn.63881 |
| A_44_P113775 | 0.00839 | 1.138736 |  | Rn.32626 |
| A_44_P114308 | 0.005052 | 1.591947 | Hsp90aa1 | Rn.119867 |
| A_44_P114491 | 0.016938 | 1.287597 | RGD1559750 | |
| A_44_P114500 | 0.000371 | 3.485538 | Gipc2 | Rn.176667 |
| A_44_P114589 | 0.003436 | 0.874172 |  | Rn.18256 |
| A_44_P114606 | 0.002069 | 2.755705 |  | Rn.124755 |
| A_44_P115896 | 0.041764 | 0.814385 |  | Rn.25006 |
| A_44_P116187 | 0.005926 | 3.356088 |  |  |
| A_44_P116677 | 0.003849 | 0.841913 | Dhrsx | Rn.12762 |
| A_44_P117078 | 0.000745 | 1.188677 |  |  |
| A_44_P117107 | 0.00282 | 0.924241 | Rpsa | Rn.161973 |
| A_44_P117245 | 0.01886 | 0.937553 |  |  |
| A_44_P118326 | 0.033737 | 1.378563 |  | Rn.118264 |
| A_44_P118342 | 0.002464 | 1.275835 |  | Rn.203028 |
| A_44_P118805 | 0.030706 | 0.891193 | Hgs | Rn.21 |
| A_44_P119019 | 0.008824 | 1.342935 | Cck | Rn.9781 |
| A_44_P119244 | 0.043869 | 1.653804 |  | Rn.162594 |
| A_44_P119296 | 0.031429 | 1.102894 | Ggps1 | Rn.54975 |
| A_44_P119442 | 0.039566 | 0.901296 | Creb3 | Rn.104043 |
| A_44_P119633 | 0.0254 | 0.863582 |  |  |
| A_44_P119666 | 0.044292 | 0.851924 | Stard3 | Rn.145430 |
| A_44_P119956 | 0.048742 | 1.135575 |  |  |
| A_44_P120167 | 0.008363 | 1.464289 |  | Rn.8590 |
| A_44_P120367 | 0.018949 | 1.169466 |  | Rn.9406 |
| A_44_P120907 | 0.013107 | 1.181789 |  | Rn.164961 |
| A_44_P121039 | 0.042917 | 0.893914 | Canx | Rn.1762 |
| A_44_P121477 | 0.007211 | 1.379918 | Mrpl21 | Rn.22778 |
| A_44_P121511 | 0.004241 | 1.367867 |  |  |
| A_44_P121736 | 0.039597 | 0.851745 | RGD1564338 | |
| A_44_P121867 | 0.026706 | 1.553322 |  |  |
| A_44_P122141 | 0.038447 | 0.746727 | Klhl22 | Rn.23143 |
| A_44_P122547 | 0.046056 | 1.297903 | Pdcd6ip | Rn.101381 |
| A_44_P123078 | 0.049714 | 0.848494 |  | Rn.122316 |
| A_44_P123102 | 0.006321 | 0.88814 | LOC294154 | Rn.116613 |
| A_44_P123515 | 0.001026 | 1.382967 | Prb1 | Rn.29950 |
| A_44_P123571 | 0.019916 | 0.853138 | Acy1 | Rn.3679 |
| A_44_P123631 | 0.001474 | 0.842293 | Atp9b | Rn.40336 |
| A_44_P123898 | 0.044765 | 0.761656 | Ddhd2 | Rn.214543 |
| A_44_P124225 | 0.00205 | 0.871869 | Rad21 | Rn.3991 |
| A_44_P124592 | 0.022233 | 0.769033 | Cyb561 | Rn.14673 |
| A_44_P124772 | 0.033854 | 0.872872 |  | Rn.24209 |
| A_44_P125575 | 7.54E-06 | 1.275894 |  | Rn.92965 |
| A_44_P126134 | 0.001048 | 0.674905 | Syvn1 | Rn.162486 |
| A_44_P126572 | 0.0475 | 0.836095 | Zfp251 | Rn.214854 |
| A_44_P127196 | 0.001161 | 1.434598 |  | Rn.202951 |
| A_44_P127318 | 0.000417 | 0.881124 |  | Rn.219088 |
| A_44_P128195 | 0.008013 | 0.54155 | Ppp1r1b | Rn.70366 |
| A_44_P128201 | 0.028261 | 1.267666 |  |  |
| A_44_P128229 | 0.006569 | 0.725448 | Baiap2 | Rn.95155 |
| A_44_P128523 | 0.001672 | 1.253097 | RGD1565806 | |
| A_44_P128583 | 0.004631 | 1.256763 | Ranbp3 | Rn.101054 |
| A_44_P128711 | 0.002164 | 1.211159 | Sh3bgrl | Rn.58623 |
| A_44_P128912 | 0.032575 | 0.910583 |  | Rn.129828 |
| A_44_P128980 | 0.007026 | 1.257854 |  | Rn.8320 |
| A_44_P129665 | 0.037817 | 1.27329 |  | Rn.225573 |
| A_44_P129666 | 0.036345 | 1.157285 |  | Rn.225573 |
| A_44_P129853 | 0.001652 | 1.178773 |  | Rn.221237 |
| A_44_P130175 | 0.010998 | 1.16515 |  | Rn.144704 |
| A_44_P130226 | 0.004964 | 1.174752 | Gpbp1 | Rn.33325 |
| A_44_P130516 | 0.026872 | 0.704028 | RT1-Db1 | Rn.33311 |
| A_44_P130641 | 0.034149 | 0.821454 | Txn2 | Rn.55043 |
| A_44_P130780 | 0.000303 | 1.210489 | Rpl13 | Rn.908 |
| A_44_P130909 | 0.00535 | 1.17294 |  |  |
| A_44_P131036 | 0.005834 | 0.842989 | Sdccag3 | Rn.110254 |
| A_44_P131084 | 0.007129 | 1.211509 | Rbmx2 | Rn.67383 |
| A_44_P131240 | 0.00627 | 0.860726 |  | Rn.79807 |
| A_44_P131870 | 0.027889 | 1.236605 |  | Rn.219352 |
| A_44_P132333 | 0.003052 | 1.588663 |  | Rn.203111 |
| A_44_P132411 | 0.006532 | 1.114441 | Mff | Rn.93479 |
| A_44_P132464 | 0.026266 | 1.133316 |  | Rn.1780 |
| A_44_P133044 | 0.000167 | 1.592732 | LOC682663 | Rn.190485 |
| A_44_P133415 | 0.03499 | 0.705475 | MGC94199 | Rn.7569 |
| A_44_P134405 | 0.01051 | 0.764926 | Slc12a2 | Rn.11523 |
| A_44_P134584 | 0.030047 | 0.863863 | Arhgdia | Rn.1801 |
| A_44_P134634 | 0.011601 | 0.783692 |  | Rn.222762 |
| A_44_P134739 | 0.0097 | 0.849312 | Csde1 | Rn.3562 |
| A_44_P134825 | 0.014415 | 1.337097 | Tbce | Rn.3547 |
| A_44_P135434 | 0.000699 | 3.063185 |  |  |
| A_44_P135678 | 0.030581 | 0.848769 | Phf20 | Rn.38255 |
| A_44_P135964 | 0.034376 | 0.879208 | LOC691496 | Rn.48706 |
| A_44_P136841 | 0.002421 | 1.208205 |  | Rn.2758 |
| A_44_P137040 | 0.022809 | 1.186909 |  | Rn.65053 |
| A_44_P137215 | 0.031586 | 0.895747 |  |  |
| A_44_P137247 | 0.045462 | 0.742675 | Trim35 | Rn.153980 |
| A_44_P137262 | 0.011915 | 0.739866 | Nmt1 | Rn.830 |
| A_44_P137356 | 0.040791 | 0.793789 | Herpud1 | Rn.4028 |
| A_44_P137406 | 0.004834 | 0.838817 | Pebp1 | Rn.29745 |
| A_44_P137962 | 0.000779 | 0.749294 | Cdk9 | Rn.98228 |
| A_44_P137981 | 0.008885 | 1.515042 |  |  |
| A_44_P138057 | 0.044623 | 0.942195 | Rpl13 | Rn.908 |
| A_44_P138081 | 0.037471 | 1.203126 | Med14 | Rn.222594 |
| A_44_P138230 | 0.006493 | 1.457427 |  |  |
| A_44_P138231 | 0.002797 | 1.622675 |  |  |
| A_44_P138257 | 0.001843 | 0.806889 | Tsta3 | Rn.6606 |
| A_44_P138289 | 0.038388 | 0.845716 | Osgepl1 | Rn.210040 |
| A_44_P138363 | 0.001365 | 0.840077 | Hira | Rn.14920 |
| A_44_P138689 | 0.000161 | 2.173984 |  | Rn.37338 |
| A_44_P139075 | 0.046126 | 1.135363 | Txnl1 | Rn.40430 |
| A_44_P139494 | 0.007823 | 0.60424 | Mef2bnb | Rn.27139 |
| A_44_P139500 | 0.011956 | 0.475212 |  | Rn.27139 |
| A_44_P139522 | 0.017709 | 1.234893 | Rpl21 | Rn.129926 |
| A_44_P139561 | 0.002735 | 1.344528 |  | Rn.222703 |
| A_44_P140092 | 0.006492 | 0.646459 | Myh14 | Rn.143782 |
| A_44_P140214 | 2.09E-05 | 1.702807 | RGD1560069 | |
| A_44_P140225 | 0.002848 | 0.834579 | Eif4g1 | Rn.101803 |
| A_44_P140400 | 0.005393 | 0.910128 | Rpl10a | Rn.2262 |
| A_44_P140805 | 0.00416 | 0.849967 | Top2b | Rn.104585 |
| A_44_P141513 | 0.014104 | 0.877309 | LOC361990 | Rn.15859 |
| A_44_P141644 | 0.014087 | 1.120542 |  | Rn.122476 |
| A_44_P142184 | 0.013774 | 0.620722 |  | Rn.108603 |
| A_44_P142335 | 0.046922 | 0.84848 | Dyrk1b | Rn.75845 |
| A_44_P142352 | 0.021448 | 0.762404 | Dhx9 | Rn.107359 |
| A_44_P142454 | 0.000764 | 2.574617 |  |  |
| A_44_P142492 | 0.021723 | 0.730836 | Ubqln4 | Rn.17785 |
| A_44_P142536 | 0.003606 | 0.782375 |  |  |
| A_44_P142562 | 0.016167 | 0.815146 | Tmx2 | Rn.3027 |
| A_44_P142606 | 0.009877 | 0.841226 | Papd5 | Rn.22363 |
| A_44_P142652 | 0.00364 | 0.845508 | Pcgf1 | Rn.203514 |
| A_44_P143011 | 0.022026 | 0.864365 |  | Rn.12421 |
| A_44_P143061 | 0.016093 | 1.362869 | Srf | Rn.1501 |
| A_44_P143650 | 0.040881 | 1.097003 |  | Rn.2344 |
| A_44_P144064 | 0.007058 | 0.730207 | Stk16 | Rn.13308 |
| A_44_P144277 | 0.003387 | 0.796622 | Pkn1 | Rn.49880 |
| A_44_P144796 | 0.000131 | 3.370523 |  |  |
| A_44_P144817 | 3.69E-05 | 1.83468 |  |  |
| A_44_P144943 | 0.002663 | 0.854469 | Wdr83 | Rn.3570 |
| A_44_P145239 | 0.002276 | 1.295962 | LOC683920 | |
| A_44_P145422 | 0.002129 | 1.216175 | LOC100360384 | Rn.155723 |
| A_44_P146444 | 0.00354 | 1.115464 |  | Rn.109370 |
| A_44_P147106 | 5.02E-05 | 1.761181 |  |  |
| A_44_P147470 | 0.003927 | 0.847029 |  |  |
| A_44_P147572 | 0.048478 | 0.749402 |  | Rn.219258 |
| A_44_P147600 | 0.043495 | 0.909274 | Npat | Rn.18968 |
| A_44_P147806 | 0.038397 | 0.914668 | LOC100366273 | Rn.42519 |
| A_44_P148196 | 0.047018 | 1.179939 | Klk5l |  |
| A_44_P148280 | 0.009068 | 0.878131 | Crcp | Rn.9449 |
| A_44_P148308 | 0.023458 | 0.791954 | Klf13 | Rn.22229 |
| A_44_P148531 | 0.003474 | 1.604396 |  | Rn.11612 |
| A_44_P148554 | 0.013127 | 1.449995 |  | Rn.6535 |
| A_44_P148742 | 0.032986 | 1.674474 |  | Rn.2339 |
| A_44_P148807 | 0.00302 | 0.83962 | Scyl1 | Rn.143751 |
| A_44_P149036 | 0.012071 | 0.888878 | Rpsa | Rn.161973 |
| A_44_P149380 | 0.007122 | 0.737929 | Aoc3 | Rn.198327 |
| A_44_P149563 | 0.010643 | 0.789226 | Zfp646 | Rn.105699 |
| A_44_P149954 | 0.027316 | 0.87783 | Sdccag3 | Rn.110254 |
| A_44_P149958 | 0.036291 | 0.868683 | Foxn3 | Rn.105640 |
| A_44_P149999 | 0.041556 | 0.831932 | RGD1305464 | Rn.59239 |
| A_44_P150036 | 0.029992 | 0.866621 | Atf7 | Rn.219706 |
| A_44_P151001 | 0.004886 | 1.110758 |  | Rn.4199 |
| A_44_P151422 | 0.040599 | 0.77285 | Zfp709 | Rn.123575 |
| A_44_P151734 | 0.027285 | 0.653417 | Atp2a3 | Rn.9920 |
| A_44_P151920 | 0.000843 | 0.760304 | Fam53b | Rn.12282 |
| A_44_P152017 | 0.000656 | 0.686237 |  | Rn.227857 |
| A_44_P152020 | 0.014703 | 1.472456 |  |  |
| A_44_P152373 | 0.008795 | 2.055899 |  |  |
| A_44_P152557 | 0.036206 | 1.126546 |  | Rn.121465 |
| A_44_P153193 | 0.009745 | 1.377471 | Pdzd9 | Rn.217594 |
| A_44_P153979 | 0.008719 | 0.691504 | Zeb1 | Rn.10600 |
| A_44_P154211 | 0.006483 | 0.913276 |  |  |
| A_44_P154773 | 0.001608 | 1.211295 |  |  |
| A_44_P154785 | 0.046282 | 1.102207 |  |  |
| A_44_P155816 | 0.041806 | 0.795276 | Eef2k | Rn.10958 |
| A_44_P156043 | 0.002615 | 1.199362 | Rer1 | Rn.129142 |
| A_44_P156136 | 0.000901 | 1.30288 |  | Rn.1123 |
| A_44_P156450 | 0.014253 | 0.877821 | Ppm1b | Rn.4143 |
| A_44_P156609 | 0.03373 | 0.839141 | Zscan21 | Rn.203972 |
| A_44_P156647 | 0.008936 | 0.714757 | Tmem163 | Rn.25191 |
| A_44_P156705 | 0.000241 | 2.758586 |  |  |
| A_44_P156718 | 4.13E-05 | 0.88689 | LOC690096 | |
| A_44_P156785 | 0.00555 | 0.827892 | RGD1311847 | Rn.219135 |
| A_44_P157045 | 0.023571 | 1.090093 |  |  |
| A_44_P157118 | 0.002218 | 0.796394 |  | Rn.42737 |
| A_44_P157652 | 0.030432 | 0.787785 | Aes | Rn.11495 |
| A_44_P157690 | 0.043371 | 0.891188 | Snrpn | Rn.11169 |
| A_44_P157721 | 0.022188 | 1.708347 |  |  |
| A_44_P157776 | 0.01636 | 1.217837 |  | Rn.17580 |
| A_44_P158635 | 0.029718 | 0.825242 | Bet1l | Rn.3234 |
| A_44_P158768 | 0.000955 | 0.712624 |  | Rn.100021 |
| A_44_P158777 | 0.014951 | 1.298001 | Prkacb | Rn.202491 |
| A_44_P158783 | 0.024715 | 0.781237 | Aoc3 | Rn.198327 |
| A_44_P158837 | 0.016225 | 0.65625 |  |  |
| A_44_P159218 | 0.000321 | 0.85144 | Sppl2a | Rn.8666 |
| A_44_P159221 | 4.42E-05 | 0.775935 | Sppl2a | Rn.8666 |
| A_44_P159271 | 0.021986 | 0.800615 | Prr15 | Rn.37820 |
| A_44_P159365 | 0.01133 | 0.824192 | Camkmt | Rn.12129 |
| A_44_P159400 | 0.011544 | 1.594857 |  |  |
| A_44_P159500 | 0.048889 | 0.876446 | Mitd1 | Rn.92704 |
| A_44_P159540 | 0.025695 | 0.826686 | Dhrs11 | Rn.204288 |
| A_44_P159745 | 0.009653 | 0.86259 |  | Rn.3356 |
| A_44_P160113 | 0.049285 | 0.870582 |  | Rn.8555 |
| A_44_P160174 | 0.04846 | 1.204302 |  | Rn.203098 |
| A_44_P160239 | 0.003207 | 1.117307 |  | Rn.873 |
| A_44_P160529 | 0.005709 | 0.869213 |  | Rn.195252 |
| A_44_P160545 | 0.001674 | 1.395494 |  | Rn.203252 |
| A_44_P160669 | 0.035506 | 0.883844 | Ddost | Rn.102842 |
| A_44_P160763 | 0.009046 | 0.84858 | Pank2 | Rn.91999 |
| A_44_P161674 | 0.000249 | 3.998615 |  |  |
| A_44_P161887 | 0.014361 | 1.334465 |  | Rn.17804 |
| A_44_P162115 | 0.010049 | 1.441841 | Slc9a9 | Rn.105909 |
| A_44_P162892 | 0.040775 | 1.353509 |  | Rn.106093 |
| A_44_P163242 | 0.000569 | 0.862651 | Epcam | Rn.106481 |
| A_44_P164007 | 0.000985 | 0.870421 | Hectd1 | Rn.36797 |
| A_44_P164170 | 0.03073 | 0.578881 | Ccr10 | Rn.49607 |
| A_44_P165196 | 0.000499 | 1.272026 |  | Rn.5041 |
| A_44_P165466 | 0.000434 | 1.5758 | Ash1l | Rn.86973 |
| A_44_P165918 | 0.039932 | 1.116621 |  |  |
| A_44_P166133 | 0.021541 | 1.104688 |  |  |
| A_44_P166186 | 0.001084 | 0.707592 | Tor1b | Rn.6487 |
| A_44_P166233 | 0.021152 | 1.787826 |  |  |
| A_44_P166267 | 0.036964 | 0.829615 | Abcb10 | Rn.22937 |
| A_44_P166372 | 0.01721 | 1.550141 |  |  |
| A_44_P168102 | 0.014944 | 0.8137 | Ppp5c | Rn.6107 |
| A_44_P168335 | 0.005015 | 1.132477 |  |  |
| A_44_P168613 | 0.004256 | 0.791575 | Wdr33 | Rn.22892 |
| A_44_P168619 | 0.007868 | 0.739338 | Arfgap2 | Rn.3707 |
| A_44_P168867 | 0.013239 | 0.822122 | Laptm4b | Rn.8123 |
| A_44_P169662 | 0.00033 | 1.189993 | Faf1 | Rn.198288 |
| A_44_P169677 | 0.008294 | 1.123977 |  | Rn.14866 |
| A_44_P169956 | 0.021245 | 1.095848 |  | Rn.164450 |
| A_44_P170018 | 0.000249 | 2.079669 | LOC304558 | Rn.137233 |
| A_44_P170496 | 0.005113 | 0.781912 | Sqrdl | Rn.1416 |
| A_44_P170971 | 1.72E-05 | 1.820409 |  | Rn.124815 |
| A_44_P171109 | 0.001389 | 1.709774 |  |  |
| A_44_P171143 | 0.00011 | 2.281347 |  |  |
| A_44_P171220 | 0.037399 | 0.728261 |  | Rn.219735 |
| A_44_P171372 | 0.046082 | 1.089461 | Vat1 | Rn.9118 |
| A_44_P173138 | 0.03161 | 0.898046 | Ehbp1l1 | Rn.103357 |
| A_44_P173239 | 0.019165 | 0.830597 | Ap4b1 | Rn.8746 |
| A_44_P173478 | 0.014694 | 1.758738 |  |  |
| A_44_P174032 | 0.032377 | 0.821168 |  | Rn.174745 |
| A_44_P174365 | 0.005701 | 0.881974 |  | Rn.11987 |
| A_44_P174588 | 0.000938 | 1.197223 |  | Rn.106034 |
| A_44_P174682 | 0.000631 | 1.34 | Nfyc | Rn.1457 |
| A_44_P175234 | 0.043243 | 0.817321 | Rgs6 | Rn.207298 |
| A_44_P175786 | 0.037328 | 0.893367 | Gnl3l | Rn.164274 |
| A_44_P175837 | 0.003727 | 0.813618 |  |  |
| A_44_P175846 | 0.033044 | 1.403965 | LOC307671 | |
| A_44_P176063 | 0.000156 | 2.434765 |  |  |
| A_44_P176494 | 0.003581 | 1.167108 | Cdc73 | Rn.162332 |
| A_44_P177013 | 0.007301 | 0.73828 |  | Rn.204460 |
| A_44_P177425 | 0.02815 | 1.183485 | Creb1 | Rn.90061 |
| A_44_P177678 | 0.00149 | 0.65774 | LOC503192 | Rn.69744 |
| A_44_P178338 | 0.038762 | 1.209802 |  | Rn.223071 |
| A_44_P179210 | 0.021594 | 1.210039 | Rpl37a | Rn.129347 |
| A_44_P180017 | 0.048994 | 0.86626 | Fmr1 | Rn.40595 |
| A_44_P180067 | 0.047402 | 1.350219 | Gjd4 | Rn.217603 |
| A_44_P180255 | 0.007325 | 0.836285 | Akt1s1 | Rn.102669 |
| A_44_P180262 | 0.002007 | 1.496455 | RGD1562397 | |
| A_44_P180294 | 0.000233 | 0.815159 | RGD1311309 | Rn.163244 |
| A_44_P180299 | 0.012405 | 0.778691 |  |  |
| A_44_P180334 | 0.002586 | 1.328735 |  |  |
| A_44_P181616 | 0.028841 | 1.218612 |  | Rn.3324 |
| A_44_P181911 | 0.009281 | 1.143941 |  | Rn.98317 |
| A_44_P182182 | 0.04402 | 0.841953 | Spata6 | Rn.8467 |
| A_44_P182721 | 5.46E-05 | 2.236493 |  |  |
| A_44_P182792 | 0.026704 | 0.886803 | Fkbp8 | Rn.99789 |
| A_44_P183007 | 0.000207 | 2.769023 |  |  |
| A_44_P183087 | 0.003763 | 0.88949 | Moap1 | Rn.98234 |
| A_44_P183134 | 0.04463 | 0.772352 | Smad6 | Rn.42472 |
| A_44_P183137 | 0.043642 | 0.874179 | Ext1 | Rn.106323 |
| A_44_P183142 | 0.001032 | 1.322779 |  | Rn.119950 |
| A_44_P183908 | 0.000138 | 1.875794 |  | Rn.217073 |
| A_44_P183944 | 0.000455 | 1.786307 |  | Rn.202982 |
| A_44_P183979 | 0.043826 | 1.092517 |  | Rn.137199 |
| A_44_P184205 | 5.48E-05 | 1.34855 |  | Rn.1965 |
| A_44_P184261 | 0.03878 | 1.118225 |  | Rn.2989 |
| A_44_P184524 | 0.034317 | 0.928086 | Rab28 | Rn.4023 |
| A_44_P184874 | 0.010304 | 1.873222 |  |  |
| A_44_P184955 | 0.022234 | 0.831904 | C2cd3 | Rn.48 |
| A_44_P185138 | 4.02E-05 | 1.406834 |  |  |
| A_44_P185195 | 0.031463 | 0.901054 | Fbxw9 | Rn.29201 |
| A_44_P185629 | 0.023633 | 1.4144 |  | Rn.128129 |
| A_44_P185661 | 0.001483 | 0.761352 | Rnf170 | Rn.156269 |
| A_44_P185673 | 0.000161 | 1.22573 |  | Rn.80611 |
| A_44_P185921 | 0.033919 | 0.734199 |  | Rn.38371 |
| A_44_P186249 | 0.004285 | 0.829509 | LOC685179 | Rn.20004 |
| A_44_P186317 | 0.012877 | 0.740315 | Eps8 | Rn.30310 |
| A_44_P186766 | 0.016312 | 1.266424 |  | Rn.161803 |
| A_44_P186919 | 0.002374 | 1.459193 | Olr546 | Rn.142913 |
| A_44_P187586 | 0.044185 | 0.893523 |  | Rn.102097 |
| A_44_P187640 | 0.010493 | 0.760028 | Dnajc30 | Rn.29127 |
| A_44_P187789 | 0.002913 | 0.814941 |  | Rn.98517 |
| A_44_P187827 | 0.006656 | 0.848837 | Spop | Rn.162165 |
| A_44_P188332 | 0.005337 | 1.331364 |  | Rn.147930 |
| A_44_P189026 | 0.023943 | 1.188163 | B3galnt2 | Rn.140763 |
| A_44_P189065 | 0.024022 | 0.867217 | Ssr2 | Rn.17458 |
| A_44_P189097 | 0.02894 | 1.276018 |  | Rn.7484 |
| A_44_P189326 | 0.012095 | 0.761443 | Pdia3 | Rn.11527 |
| A_44_P189660 | 0.005864 | 0.872571 | Zfp110 | Rn.204252 |
| A_44_P189917 | 0.029371 | 1.900819 |  |  |
| A_44_P190188 | 0.019007 | 0.79195 |  |  |
| A_44_P190200 | 0.018264 | 0.871664 | Chd3 | Rn.204523 |
| A_44_P190968 | 0.011454 | 1.118219 |  | Rn.108074 |
| A_44_P191194 | 0.022758 | 0.862842 |  | Rn.167420 |
| A_44_P191467 | 0.039113 | 0.910022 | Atp6v0e1 | Rn.789 |
| A_44_P191862 | 0.009545 | 0.897987 | Hras | Rn.102180 |
| A_44_P191878 | 0.01466 | 0.81362 | Myo1c | Rn.55135 |
| A_44_P192117 | 0.010431 | 0.856146 | LOC100362324 | Rn.103224 |
| A_44_P192548 | 0.00319 | 0.76221 | Gtpbp2 | Rn.3595 |
| A_44_P192568 | 0.005275 | 0.832257 | Gm2a | Rn.98783 |
| A_44_P192908 | 0.01989 | 0.690604 |  | Rn.167354 |
| A_44_P193593 | 2.17E-07 | 2.497074 |  | Rn.219950 |
| A_44_P193791 | 0.034613 | 1.23224 |  | Rn.54812 |
| A_44_P194088 | 0.032311 | 1.118894 | Zcchc10 | Rn.163117 |
| A_44_P194166 | 0.045591 | 1.377357 | Dhh | Rn.84274 |
| A_44_P194301 | 0.008993 | 0.85456 | Ubl3 | Rn.12128 |
| A_44_P194331 | 0.010316 | 0.770278 | Zfp787 | Rn.23427 |
| A_44_P194404 | 0.027339 | 0.74416 | Pitpnm1 | Rn.154692 |
| A_44_P194821 | 0.041949 | 1.134653 | Topbp1 | Rn.204161 |
| A_44_P194836 | 0.023405 | 0.800281 | Zdhhc3 | Rn.8573 |
| A_44_P194863 | 0.006115 | 0.840345 | Spag7 | Rn.219156 |
| A_44_P194894 | 0.047694 | 0.903412 | Osbpl2 | Rn.12390 |
| A_44_P194909 | 0.003018 | 0.73147 | Ripk4 | Rn.28403 |
| A_44_P195267 | 0.000307 | 1.4561 |  |  |
| A_44_P195468 | 0.002792 | 1.429549 | Ythdc1 | Rn.48752 |
| A_44_P195499 | 0.018446 | 1.366655 |  | Rn.166161 |
| A_44_P195816 | 0.00959 | 1.189675 |  | Rn.1133 |
| A_44_P195817 | 0.00571 | 1.202462 |  | Rn.1133 |
| A_44_P196172 | 0.043916 | 1.579113 | Rsad2 | Rn.14882 |
| A_44_P196356 | 0.016822 | 0.89395 | Idh3B | Rn.1093 |
| A_44_P196532 | 0.00054 | 2.239476 | Mk1 | Rn.201717 |
| A_44_P196805 | 0.017669 | 0.812688 | Golga7 | Rn.4105 |
| A_44_P196856 | 0.014955 | 0.860726 | Cir1 | Rn.99641 |
| A_44_P196948 | 0.035903 | 0.546764 | Rpgr | Rn.215816 |
| A_44_P197156 | 0.003677 | 0.877969 | Tbcc | Rn.18774 |
| A_44_P197258 | 0.022538 | 1.26606 |  | Rn.17453 |
| A_44_P198315 | 0.022243 | 1.231053 |  | Rn.167993 |
| A_44_P198411 | 0.003622 | 1.238039 |  | Rn.145464 |
| A_44_P199161 | 0.039818 | 0.905684 | Rps16 | Rn.29791 |
| A_44_P199211 | 0.049622 | 0.65611 | Plcb1 | Rn.45523 |
| A_44_P199381 | 0.000128 | 1.42155 | RGD1565679 | |
| A_44_P199560 | 8.71E-05 | 1.188752 |  | Rn.880 |
| A_44_P200146 | 0.006539 | 0.778906 | Oxr1 | Rn.4252 |
| A_44_P200507 | 0.001458 | 1.257082 | Khdrbs1 | Rn.162107 |
| A_44_P200617 | 0.045582 | 0.78326 | Ube2o | Rn.137727 |
| A_44_P200747 | 0.00689 | 1.337832 |  | Rn.13492 |
| A_44_P200960 | 0.028313 | 1.18342 | Olr776 | Rn.142754 |
| A_44_P201077 | 0.042492 | 0.790675 | Asrgl1 | Rn.22774 |
| A_44_P201603 | 0.035409 | 0.647822 | Hnrnpa2b1 | Rn.4057 |
| A_44_P201981 | 0.002655 | 0.810534 | Inpp5a | Rn.8618 |
| A_44_P202573 | 0.001715 | 1.967965 |  | Rn.2947 |
| A_44_P202726 | 0.011257 | 0.725005 |  | Rn.198242 |
| A_44_P203173 | 0.018697 | 1.298262 | Rtn4rl2 | Rn.162683 |
| A_44_P203613 | 0.027409 | 1.296589 | Hnrnpul1 | Rn.95170 |
| A_44_P203665 | 0.027497 | 0.855021 | Ccng2 | Rn.162246 |
| A_44_P203838 | 0.029879 | 0.902303 |  |  |
| A_44_P203967 | 0.01692 | 0.816315 | Recql | Rn.203166 |
| A_44_P204134 | 0.049038 | 0.873 |  |  |
| A_44_P204246 | 0.041463 | 0.82883 | Ubxn7 |  |
| A_44_P204648 | 1.36E-05 | 2.422594 |  |  |
| A_44_P205509 | 0.000245 | 1.180832 |  | Rn.94978 |
| A_44_P205771 | 0.007567 | 1.181529 | Dync1i2 | Rn.11014 |
| A_44_P205928 | 0.000267 | 0.778897 | Zkscan5 | Rn.38399 |
| A_44_P206030 | 0.004421 | 0.8278 | LOC498369 | |
| A_44_P206244 | 0.018918 | 0.773735 | Tpst2 | Rn.3659 |
| A_44_P206270 | 0.004542 | 0.931309 |  |  |
| A_44_P206284 | 0.041117 | 0.76854 | RGD1566215 | Rn.216502 |
| A_44_P206417 | 0.021589 | 1.237795 |  |  |
| A_44_P206434 | 0.000771 | 1.256521 | Lmnb2 | Rn.12408 |
| A_44_P206439 | 0.000678 | 2.145512 |  |  |
| A_44_P206466 | 0.007426 | 0.876388 | Senp6 | Rn.98553 |
| A_44_P206526 | 0.026187 | 0.80625 | Adamts2 | Rn.86986 |
| A_44_P206550 | 0.032681 | 0.890185 | Ctdnep1 | Rn.4211 |
| A_44_P206675 | 0.022933 | 0.845295 |  | Rn.165170 |
| A_44_P206825 | 0.008384 | 2.154975 |  | Rn.225228 |
| A_44_P207242 | 0.01827 | 1.590457 |  | Rn.11017 |
| A_44_P207595 | 0.005125 | 1.144666 |  | Rn.162714 |
| A_44_P207855 | 0.000764 | 1.237255 |  | Rn.202945 |
| A_44_P207881 | 0.003038 | 0.808452 | Asf1a | Rn.163427 |
| A_44_P208624 | 0.021248 | 0.870118 | RGD1308612 | Rn.198184 |
| A_44_P208733 | 0.006089 | 1.448998 |  |  |
| A_44_P208857 | 0.020583 | 0.305486 | Mlc1 | Rn.98819 |
| A_44_P208880 | 0.010158 | 0.731279 | Dnaja3 | Rn.198254 |
| A_44_P209013 | 0.000517 | 1.400756 |  | Rn.9501 |
| A_44_P209220 | 0.049658 | 1.177572 | Zfp474 | Rn.163374 |
| A_44_P209926 | 0.048673 | 0.842946 | Hectd1 | Rn.36797 |
| A_44_P209949 | 0.006169 | 1.104127 |  | Rn.17458 |
| A_44_P210440 | 0.03269 | 0.863349 | Akap13 | Rn.2027 |
| A_44_P210563 | 0.040055 | 0.857853 | H1f0 | Rn.3129 |
| A_44_P210931 | 0.002614 | 0.800067 |  | Rn.201992 |
| A_44_P211028 | 0.000306 | 3.068938 |  |  |
| A_44_P211090 | 0.002447 | 0.831434 | Mbrl | Rn.37168 |
| A_44_P211440 | 0.000967 | 0.870555 | Araf | Rn.1714 |
| A_44_P211878 | 0.002216 | 1.150255 |  | Rn.96380 |
| A_44_P212355 | 0.01666 | 0.866155 |  | Rn.217990 |
| A_44_P212424 | 0.000836 | 0.817656 | RGD1560212 | Rn.98561 |
| A_44_P212552 | 6.21E-05 | 3.775629 | Tob1 | Rn.228429 |
| A_44_P212676 | 0.015563 | 0.753264 | Crat | Rn.6249 |
| A_44_P212695 | 0.022412 | 0.959039 |  |  |
| A_44_P212703 | 0.003619 | 0.757235 | Kcnq1 | Rn.9779 |
| A_44_P212708 | 0.012083 | 0.884619 | Psmd4 | Rn.3830 |
| A_44_P214229 | 0.003173 | 1.193327 |  | Rn.162681 |
| A_44_P214379 | 0.02715 | 0.921229 | Kif16b | Rn.7029 |
| A_44_P214415 | 0.029707 | 0.902621 | Yeats4 | Rn.103329 |
| A_44_P214650 | 0.005385 | 1.300525 |  | Rn.973 |
| A_44_P214705 | 0.049936 | 0.860721 | Tmem59 | Rn.105907 |
| A_44_P214797 | 0.003702 | 1.270025 |  | Rn.178356 |
| A_44_P215544 | 0.000605 | 1.992144 | RGD1565920 | |
| A_44_P215720 | 0.002552 | 2.262823 |  |  |
| A_44_P215731 | 1.71E-06 | 1.440229 |  | Rn.212628 |
| A_44_P216143 | 0.045854 | 0.883173 | Luc7l | Rn.99992 |
| A_44_P217049 | 0.013707 | 1.308735 |  |  |
| A_44_P217273 | 0.039965 | 0.919443 | Csde1 | Rn.3562 |
| A_44_P217821 | 0.012824 | 0.84134 | Bap1 | Rn.3382 |
| A_44_P218097 | 0.012925 | 0.8696 |  | Rn.4241 |
| A_44_P218263 | 0.009682 | 1.334502 | Sned1 | Rn.23447 |
| A_44_P218695 | 0.001777 | 1.351293 |  | Rn.4165 |
| A_44_P219322 | 0.028994 | 0.742437 |  | Rn.94887 |
| A_44_P219470 | 0.018756 | 0.804478 | Snx17 | Rn.162846 |
| A_44_P220197 | 0.021938 | 0.876234 |  | Rn.36809 |
| A_44_P220305 | 0.042007 | 0.707123 |  |  |
| A_44_P220351 | 0.015437 | 1.173085 | Wasl | Rn.216426 |
| A_44_P220378 | 0.001732 | 1.53241 | Mical3 | Rn.167413 |
| A_44_P220410 | 0.040457 | 1.075441 |  |  |
| A_44_P220575 | 0.013636 | 1.484038 | Irak4 | Rn.61427 |
| A_44_P220610 | 0.043201 | 0.833355 | Fahd1 | Rn.3918 |
| A_44_P220661 | 0.027054 | 0.87475 |  |  |
| A_44_P220868 | 0.005454 | 1.113874 | Rpn2 | Rn.2879 |
| A_44_P221213 | 0.000354 | 1.135619 |  | Rn.1441 |
| A_44_P221260 | 0.007474 | 0.746483 |  | Rn.10241 |
| A_44_P221287 | 0.000355 | 0.792951 | Cc2d1b | Rn.3265 |
| A_44_P221295 | 0.0202 | 1.862651 | Pm20d2 | Rn.23149 |
| A_44_P222367 | 0.007085 | 0.786547 |  |  |
| A_44_P222566 | 0.002601 | 1.417257 |  |  |
| A_44_P222673 | 0.008048 | 0.821157 | Tysnd1 | Rn.20699 |
| A_44_P222679 | 0.013507 | 1.270814 | Mobkl1b | Rn.74475 |
| A_44_P222765 | 6.64E-05 | 1.381773 |  |  |
| A_44_P222855 | 0.000668 | 2.621438 |  |  |
| A_44_P222865 | 0.006868 | 1.23246 |  |  |
| A_44_P222878 | 0.01436 | 0.828336 | Eif3s6ip | Rn.22102 |
| A_44_P223252 | 0.00306 | 0.883738 | Psmc3 | Rn.11173 |
| A_44_P223607 | 0.048314 | 0.822756 |  | Rn.175195 |
| A_44_P223741 | 0.001293 | 1.341616 |  | Rn.116073 |
| A_44_P223817 | 0.001262 | 0.79339 | Atp5a1 | Rn.40255 |
| A_44_P224832 | 0.009378 | 1.312024 | Sdhaf2 | Rn.102071 |
| A_44_P224991 | 0.00302 | 2.547417 |  |  |
| A_44_P225239 | 0.001268 | 1.96144 |  |  |
| A_44_P226029 | 0.008699 | 1.266544 | Rbmxrtl | Rn.19966 |
| A_44_P226771 | 0.00906 | 1.385443 | Mapk8ip3 | Rn.16158 |
| A_44_P226858 | 0.029862 | 0.750018 | Mrfap1 | Rn.3393 |
| A_44_P226881 | 0.007453 | 0.695381 | Pde2a | Rn.10044 |
| A_44_P227361 | 0.030994 | 0.871159 | Bms1 | Rn.41094 |
| A_44_P227421 | 0.000186 | 2.471405 |  |  |
| A_44_P228351 | 0.038507 | 0.881091 | Cd47 | Rn.7409 |
| A_44_P228715 | 0.020341 | 0.892331 | Smad4 | Rn.9774 |
| A_44_P228839 | 0.030949 | 0.808562 | Pgrmc2 | Rn.7685 |
| A_44_P229352 | 0.020984 | 1.275562 | Rgl1 | Rn.28005 |
| A_44_P229359 | 0.008094 | 0.738199 | Mesp2 | Rn.217791 |
| A_44_P229451 | 0.034772 | 1.920875 |  |  |
| A_44_P229490 | 0.049569 | 0.930688 | Rpl31 | Rn.1101 |
| A_44_P229656 | 0.027913 | 0.915387 | RGD1306576 | Rn.21605 |
| A_44_P229887 | 0.019907 | 0.690004 | Acbd4 | Rn.18284 |
| A_44_P230813 | 0.017655 | 0.850199 | Rhot1 | Rn.7462 |
| A_44_P230875 | 0.015643 | 0.76555 |  | Rn.985 |
| A_44_P230960 | 0.003839 | 0.85747 |  | Rn.7380 |
| A_44_P231066 | 0.035778 | 0.938525 | Uba1 | Rn.11800 |
| A_44_P231113 | 0.011182 | 1.119979 |  | Rn.177081 |
| A_44_P231283 | 0.020692 | 1.280185 | Nupl1 | Rn.11099 |
| A_44_P231418 | 0.005722 | 1.204937 | Atrn | Rn.53846 |
| A_44_P231497 | 0.001523 | 1.43457 | Ddx1 | Rn.65536 |
| A_44_P231597 | 0.040713 | 0.673974 | Hpcal1 | Rn.102820 |
| A_44_P231689 | 0.03845 | 1.233095 |  |  |
| A_44_P231706 | 0.04731 | 0.796901 | Il16 | Rn.104665 |
| A_44_P231737 | 0.000107 | 2.156867 | RGD1562755 | |
| A_44_P231867 | 0.034303 | 1.160237 | Npepo | Rn.263 |
| A_44_P231994 | 0.014461 | 0.852258 |  | Rn.226440 |
| A_44_P232113 | 0.000301 | 2.914947 |  |  |
| A_44_P232205 | 0.032085 | 0.800351 | Bin2 | Rn.47919 |
| A_44_P232285 | 0.000571 | 3.287113 |  |  |
| A_44_P232290 | 0.011111 | 0.83124 | Dirc2 | Rn.137795 |
| A_44_P233231 | 0.006517 | 0.895898 |  | Rn.216496 |
| A_44_P233399 | 0.004232 | 1.214483 |  | Rn.3491 |
| A_44_P233742 | 0.037968 | 0.808554 | Tspan5 | Rn.98240 |
| A_44_P233753 | 0.03849 | 0.798604 | Tmem106b | Rn.106000 |
| A_44_P233932 | 0.005883 | 0.741479 | Notch1 | Rn.25046 |
| A_44_P234257 | 0.000416 | 2.188019 | LOC291686 | |
| A_44_P234460 | 0.001104 | 1.368371 |  |  |
| A_44_P234500 | 0.000138 | 2.074314 |  |  |
| A_44_P234636 | 0.030957 | 0.901599 | Psmd2 | Rn.29909 |
| A_44_P234970 | 0.002728 | 0.662704 | Prmt2 | Rn.145566 |
| A_44_P235815 | 0.006276 | 1.257079 | Clns1a | Rn.4089 |
| A_44_P235848 | 0.000375 | 1.780391 | Bccip | Rn.154691 |
| A_44_P235978 | 0.030137 | 0.734926 | Fasn | Rn.9486 |
| A_44_P236378 | 0.000688 | 2.494972 |  |  |
| A_44_P236588 | 0.003894 | 0.586494 | Trpm2 | Rn.214495 |
| A_44_P236636 | 0.00178 | 1.779803 |  |  |
| A_44_P236876 | 0.004092 | 1.550774 |  |  |
| A_44_P237153 | 0.009355 | 1.357701 | Birc5 | Rn.54471 |
| A_44_P237194 | 0.008127 | 0.810453 |  | Rn.98420 |
| A_44_P237455 | 0.000968 | 0.820172 |  |  |
| A_44_P237621 | 0.001949 | 1.867784 | Txnip | Rn.2758 |
| A_44_P238733 | 0.006304 | 3.334784 | Tmed5 | Rn.204558 |
| A_44_P238789 | 0.009889 | 1.210227 |  |  |
| A_44_P238906 | 0.000921 | 1.411593 |  |  |
| A_44_P238939 | 0.004085 | 1.246828 |  |  |
| A_44_P239095 | 0.004165 | 0.760839 | Hmgb3 | Rn.203482 |
| A_44_P239105 | 0.009588 | 0.821069 | Zfyve9 | Rn.23612 |
| A_44_P239650 | 0.000285 | 1.404825 |  |  |
| A_44_P239864 | 0.011422 | 1.120824 |  | Rn.2855 |
| A_44_P240512 | 0.026868 | 1.081132 |  | Rn.3792 |
| A_44_P241177 | 0.042863 | 1.257579 |  |  |
| A_44_P241230 | 0.000228 | 2.017058 |  |  |
| A_44_P241498 | 0.018915 | 0.769619 | Map7d1 | Rn.39212 |
| A_44_P241541 | 0.014106 | 0.788567 | Tcp11l2 | Rn.101085 |
| A_44_P242601 | 0.045585 | 1.554552 |  | Rn.98346 |
| A_44_P242614 | 0.005338 | 0.84966 | Znrf2 | Rn.32245 |
| A_44_P243126 | 0.017493 | 0.73076 | Cux1 | Rn.10455 |
| A_44_P243153 | 0.024555 | 0.8487 | Ncoa6 | Rn.9077 |
| A_44_P243238 | 0.041544 | 0.899354 | Got2 | Rn.98650 |
| A_44_P243477 | 0.012248 | 0.540335 |  |  |
| A_44_P243556 | 1.05E-06 | 1.28971 |  |  |
| A_44_P243826 | 0.001136 | 0.749775 | Adck5 | Rn.145222 |
| A_44_P244495 | 0.029901 | 1.325338 |  | Rn.107401 |
| A_44_P244516 | 0.024476 | 1.145086 |  | Rn.4213 |
| A_44_P244535 | 0.031469 | 1.14827 |  | Rn.8555 |
| A_44_P245761 | 0.000451 | 1.248995 | Uvrag | Rn.99218 |
| A_44_P245795 | 0.042964 | 0.793767 | Ccdc88b | Rn.22455 |
| A_44_P245806 | 0.006292 | 1.481254 |  |  |
| A_44_P245825 | 0.000582 | 2.919474 |  |  |
| A_44_P245893 | 0.014261 | 0.780916 | Snx25 | Rn.16962 |
| A_44_P246237 | 0.00184 | 0.839411 | Uaca | Rn.199118 |
| A_44_P246343 | 0.016005 | 0.816873 |  |  |
| A_44_P246381 | 0.040992 | 0.870116 | Dgcr6 | Rn.22467 |
| A_44_P246452 | 0.015236 | 0.831776 | RGD1565043 | Rn.18942 |
| A_44_P247615 | 0.000195 | 2.169493 |  | Rn.21406 |
| A_44_P248083 | 0.004033 | 1.140473 |  |  |
| A_44_P248619 | 0.042031 | 0.819344 |  |  |
| A_44_P248634 | 0.001135 | 0.785301 | Selo | Rn.201762 |
| A_44_P249565 | 0.004893 | 1.11265 |  | Rn.164207 |
| A_44_P249805 | 0.033375 | 0.882415 | Zmym4 | Rn.3185 |
| A_44_P250620 | 0.032783 | 1.117425 | Fhdc1 | Rn.4218 |
| A_44_P250798 | 0.034388 | 1.1118 | RGD1566136 | |
| A_44_P250839 | 3.63E-06 | 1.445673 |  |  |
| A_44_P250983 | 0.049474 | 0.79705 | Nrbp2 | Rn.198238 |
| A_44_P251897 | 0.000341 | 1.351818 |  | Rn.224540 |
| A_44_P251908 | 0.013101 | 1.173868 | Xpnpep1 | Rn.25763 |
| A_44_P252091 | 0.034513 | 0.900836 | Rheb | Rn.859 |
| A_44_P252435 | 0.001941 | 1.158521 | Zc3h18 | Rn.36848 |
| A_44_P252666 | 0.006277 | 0.829487 | Nup62 | Rn.54450 |
| A_44_P252678 | 0.011757 | 0.773235 | Phb2 | Rn.64535 |
| A_44_P252855 | 0.000193 | 1.528971 |  |  |
| A_44_P252861 | 0.002988 | 0.830867 |  | Rn.5977 |
| A_44_P253268 | 0.000332 | 1.88611 |  |  |
| A_44_P253514 | 0.025616 | 0.457914 | LOC363827 | Rn.201233 |
| A_44_P253858 | 0.036362 | 1.084683 | Sumo1 | Rn.1221 |
| A_44_P254144 | 0.018143 | 0.890868 | Aftph | Rn.105388 |
| A_44_P254229 | 0.024661 | 0.813931 | Rbbp7 | Rn.3600 |
| A_44_P254413 | 0.036894 | 0.917974 | Hnrnpu | Rn.4328 |
| A_44_P254855 | 1.31E-05 | 1.986545 | Usp33 | Rn.25188 |
| A_44_P254896 | 0.001005 | 1.398656 | Ddb1 |  |
| A_44_P255201 | 0.030159 | 0.851354 | Cant1 | Rn.7241 |
| A_44_P255254 | 0.027507 | 1.088005 | Ywhab | Rn.8653 |
| A_44_P255359 | 0.001769 | 1.387895 | Ndufs2 | Rn.225926 |
| A_44_P255499 | 0.001476 | 0.923759 | Ube2q1 | Rn.19034 |
| A_44_P255501 | 0.001728 | 0.857335 | Ube2q1 | Rn.19034 |
| A_44_P255905 | 0.002334 | 0.760633 | Tnk1 | Rn.163138 |
| A_44_P255954 | 0.035705 | 0.756887 | Kctd2 | Rn.21595 |
| A_44_P256345 | 0.00021 | 1.26393 | Smarcad1 | Rn.7758 |
| A_44_P256616 | 0.016172 | 1.19659 |  | Rn.2022 |
| A_44_P256629 | 0.045073 | 0.85836 | Cnbp | Rn.6187 |
| A_44_P257058 | 0.018434 | 3.510071 | Adamts1 | Rn.7897 |
| A_44_P257492 | 0.038161 | 0.735169 | Adam4 | Rn.209651 |
| A_44_P257528 | 0.000928 | 1.442003 | LOC287010 | |
| A_44_P257563 | 0.042949 | 0.782002 | Cdk18 | Rn.219420 |
| A_44_P257774 | 0.004626 | 0.859889 | Zmiz2 | Rn.6378 |
| A_44_P258277 | 0.009286 | 0.802424 | Dhrs7b | Rn.203124 |
| A_44_P258497 | 0.015358 | 0.511767 | Prmt2 | Rn.145566 |
| A_44_P258751 | 0.027579 | 0.903542 | Pdpk1 | Rn.10905 |
| A_44_P259329 | 0.028476 | 0.77878 | Tlk1 | Rn.6114 |
| A_44_P259521 | 0.000158 | 1.39022 |  | Rn.40171 |
| A_44_P259644 | 0.009347 | 0.780932 | LOC497934 | Rn.45766 |
| A_44_P259908 | 0.049214 | 0.843337 | Glud1 | Rn.55106 |
| A_44_P260170 | 0.025803 | 0.851164 | Bcl2l12 | Rn.106127 |
| A_44_P260285 | 0.000819 | 1.62942 |  |  |
| A_44_P260487 | 0.010555 | 0.857588 | M6pr | Rn.91427 |
| A_44_P260542 | 0.001368 | 1.774864 |  |  |
| A_44_P260673 | 0.026636 | 0.819918 | Taf8 | Rn.35557 |
| A_44_P260850 | 0.049026 | 0.699362 | Cdig2 | Rn.115253 |
| A_44_P261677 | 0.002698 | 1.166209 | Zfp276 | Rn.140797 |
| A_44_P262113 | 0.00297 | 0.836907 | Napa | Rn.31745 |
| A_44_P262490 | 0.023888 | 2.152566 |  |  |
| A_44_P262608 | 0.003229 | 1.27777 |  |  |
| A_44_P262625 | 0.000105 | 1.818114 |  |  |
| A_44_P263740 | 0.027295 | 0.686896 | Tmem86a | Rn.23056 |
| A_44_P263957 | 0.040503 | 1.172354 | Oxr1 | Rn.4252 |
| A_44_P264276 | 0.047954 | 0.879139 | Tusc2 | Rn.120731 |
| A_44_P264567 | 0.04495 | 0.916773 | Mrp63 | Rn.22630 |
| A_44_P264777 | 0.00954 | 0.806911 | Dnajb9 | Rn.29778 |
| A_44_P264880 | 0.003857 | 2.162651 | LOC100363884 | |
| A_44_P264910 | 0.033276 | 0.880794 | Zfp771 | Rn.32501 |
| A_44_P265416 | 0.032167 | 1.206738 | Aqp12a | Rn.20532 |
| A_44_P265682 | 0.02926 | 0.785016 | Akap11 | Rn.219324 |
| A_44_P265965 | 0.00751 | 0.857932 | Smpd2 | Rn.18572 |
| A_44_P267103 | 0.019393 | 0.908978 | Rpl12 | Rn.128979 |
| A_44_P267117 | 0.009135 | 0.805763 | Gale | Rn.29908 |
| A_44_P267444 | 0.022147 | 0.798298 | Galk2 | Rn.2937 |
| A_44_P267706 | 0.015406 | 0.719915 | Apol11a-ps1 | Rn.225501 |
| A_44_P268325 | 0.0024 | 1.294649 | Ccni | Rn.6670 |
| A_44_P268919 | 0.021858 | 1.115508 |  | Rn.4300 |
| A_44_P269078 | 0.000832 | 1.252365 | Tcp1 | Rn.7102 |
| A_44_P269499 | 0.000102 | 1.926685 |  | Rn.154560 |
| A_44_P269582 | 0.028121 | 1.153077 | Dusp8 | Rn.219421 |
| A_44_P269865 | 9.10E-05 | 3.385348 | Ptges3l1 | Rn.211968 |
| A_44_P270096 | 0.02347 | 0.895494 | Smg6 | Rn.1945 |
| A_44_P270669 | 0.011442 | 1.202576 |  | Rn.203335 |
| A_44_P270700 | 0.009615 | 0.811244 |  | Rn.164882 |
| A_44_P270812 | 0.049403 | 1.25739 | Tmem189 | Rn.101810 |
| A_44_P271039 | 0.03734 | 2.35717 |  | Rn.215881 |
| A_44_P271062 | 0.021294 | 0.870748 | Tmem70 | Rn.22296 |
| A_44_P271269 | 0.020129 | 1.27825 |  | Rn.34397 |
| A_44_P271541 | 0.01271 | 1.194565 | Atp5l | Rn.107458 |
| A_44_P271720 | 0.021016 | 0.790115 | Cd151 | Rn.1465 |
| A_44_P271872 | 0.018367 | 1.546207 |  |  |
| A_44_P272048 | 0.03827 | 1.1486 | LOC680290 | Rn.151034 |
| A_44_P272266 | 0.013813 | 0.812339 | Fam3a | Rn.12310 |
| A_44_P272353 | 0.001309 | 1.598517 | LOC367117 | Rn.123639 |
| A_44_P273146 | 0.008429 | 1.121962 |  | Rn.11132 |
| A_44_P274397 | 0.02033 | 0.759152 | Cers2 | Rn.1448 |
| A_44_P274414 | 0.002734 | 2.015785 |  |  |
| A_44_P274433 | 0.024738 | 0.832611 | Sdccag3 | Rn.110254 |
| A_44_P274491 | 0.000771 | 0.76087 | Large | Rn.24338 |
| A_44_P274605 | 0.001289 | 0.839574 | RGD1562165 | |
| A_44_P274658 | 0.023557 | 0.825386 | Ldlrap1 | Rn.62309 |
| A_44_P274762 | 0.00723 | 0.705718 | Cpped1 | Rn.79124 |
| A_44_P274986 | 0.026514 | 0.90919 | Gnl3l | Rn.164274 |
| A_44_P275491 | 0.042587 | 0.818613 | Mcfd2 | Rn.7871 |
| A_44_P276227 | 0.028236 | 0.8435 | Usp19 | Rn.13484 |
| A_44_P276338 | 0.038188 | 0.863118 | Mcm6 | Rn.33226 |
| A_44_P276530 | 0.029896 | 0.8371 | Ogfod2 | Rn.61120 |
| A_44_P276698 | 0.016242 | 0.889742 | Rrnad1 | Rn.100135 |
| A_44_P276714 | 0.049656 | 0.775359 | Ankrd10 | Rn.30154 |
| A_44_P276932 | 0.003268 | 1.926624 |  |  |
| A_44_P277423 | 0.000334 | 1.656199 |  | Rn.90166 |
| A_44_P277466 | 0.008985 | 0.803775 | Abca3 | Rn.38406 |
| A_44_P277820 | 0.001168 | 1.177221 |  | Rn.33804 |
| A_44_P278113 | 0.049607 | 1.163439 |  | Rn.202982 |
| A_44_P278140 | 0.030322 | 0.884219 |  | Rn.8634 |
| A_44_P278243 | 0.008706 | 1.107705 |  | Rn.47124 |
| A_44_P278770 | 0.016179 | 0.805741 | Sart3 | Rn.101480 |
| A_44_P278843 | 0.002874 | 2.074121 |  |  |
| A_44_P278923 | 0.014878 | 0.835984 | Oxnad1 | Rn.3785 |
| A_44_P278938 | 0.007305 | 0.752985 | Elf2 | Rn.107459 |
| A_44_P279240 | 0.043427 | 0.892759 | Rpl29 | Rn.3973 |
| A_44_P279452 | 0.00192 | 1.810264 |  | Rn.152719 |
| A_44_P280199 | 0.002872 | 1.225577 |  | Rn.3856 |
| A_44_P280776 | 0.011172 | 0.740925 | Bcl2 | Rn.9996 |
| A_44_P281284 | 0.025346 | 0.793205 | RGD1310185 | Rn.168042 |
| A_44_P281393 | 4.76E-05 | 1.551519 |  |  |
| A_44_P281457 | 0.008864 | 1.138888 |  |  |
| A_44_P281482 | 0.002899 | 1.376225 | Chchd6 | Rn.6212 |
| A_44_P281750 | 0.020493 | 0.937706 | Poldip2 | Rn.1344 |
| A_44_P282063 | 0.03882 | 1.133329 |  | Rn.216513 |
| A_44_P282433 | 0.01684 | 0.832415 | Akt1 | Rn.11422 |
| A_44_P282602 | 0.002809 | 1.177744 |  | Rn.43300 |
| A_44_P282716 | 0.01532 | 0.881901 | Nit1 | Rn.64425 |
| A_44_P283366 | 0.007969 | 0.792325 | Kat7 | Rn.12618 |
| A_44_P283609 | 0.009382 | 0.793586 | Rilpl1 | Rn.62627 |
| A_44_P283688 | 0.020245 | 1.063206 | RGD1560936 | |
| A_44_P283819 | 0.017274 | 0.788936 | Dapk1 | Rn.23108 |
| A_44_P284169 | 0.004303 | 1.875536 |  |  |
| A_44_P285161 | 0.048253 | 1.12708 |  | Rn.12939 |
| A_44_P285305 | 0.03559 | 1.170959 |  | Rn.145872 |
| A_44_P285726 | 0.028509 | 0.800334 | Rcn3 | Rn.2997 |
| A_44_P286024 | 0.047844 | 0.868956 | Helq | Rn.104354 |
| A_44_P286158 | 0.003244 | 1.900138 |  |  |
| A_44_P286215 | 0.00061 | 0.765825 | Slc39a7 | Rn.103977 |
| A_44_P286226 | 0.020823 | 0.680765 | Hnrnph3 | Rn.60652 |
| A_44_P286471 | 0.01659 | 1.291379 | Sec24a | Rn.224591 |
| A_44_P287286 | 0.023986 | 0.840529 | Mars | Rn.140163 |
| A_44_P287442 | 0.009798 | 1.149287 |  | Rn.95042 |
| A_44_P288185 | 0.04834 | 0.837316 | Itpr3 | Rn.11242 |
| A_44_P288300 | 0.038619 | 1.088704 |  |  |
| A_44_P288756 | 0.002426 | 1.227955 | RGD1565117 | |
| A_44_P288796 | 0.025728 | 0.75601 | Bfar | Rn.159939 |
| A_44_P289132 | 0.00171 | 1.276965 | Spsb4 | Rn.11581 |
| A_44_P289284 | 0.006294 | 1.861521 | Shank1 | Rn.225968 |
| A_44_P289424 | 0.019961 | 0.9074 | Mrps14 | Rn.67 |
| A_44_P290357 | 0.049539 | 0.689486 | Eml2 | Rn.76362 |
| A_44_P290424 | 0.017492 | 0.892652 | Siah1a | Rn.73937 |
| A_44_P290436 | 0.000577 | 2.575854 |  | Rn.3036 |
| A_44_P290706 | 0.013133 | 0.781381 |  |  |
| A_44_P290867 | 0.000398 | 1.368409 |  |  |
| A_44_P290905 | 0.033843 | 1.19143 |  |  |
| A_44_P290936 | 0.003657 | 1.33335 | RGD1559566 | |
| A_44_P291065 | 0.001625 | 1.522252 |  |  |
| A_44_P291172 | 0.009737 | 1.080862 |  |  |
| A_44_P291175 | 0.017063 | 0.702413 |  |  |
| A_44_P292314 | 0.028561 | 1.16403 |  | Rn.92304 |
| A_44_P293001 | 0.003267 | 0.701538 | Arse | Rn.79118 |
| A_44_P293187 | 0.014753 | 1.285003 | RGD1560997 | |
| A_44_P293283 | 0.031849 | 1.939615 |  |  |
| A_44_P293463 | 0.030257 | 0.871271 |  | Rn.5977 |
| A_44_P293667 | 0.004234 | 0.789846 | Vps33b | Rn.10661 |
| A_44_P293750 | 0.015821 | 0.863247 | Rnf14 | Rn.115357 |
| A_44_P294467 | 0.04764 | 0.870535 | Paxip1 | Rn.136961 |
| A_44_P294961 | 0.012695 | 0.794934 | Exoc8 | Rn.10901 |
| A_44_P295125 | 2.64E-05 | 1.801009 |  |  |
| A_44_P295385 | 0.033381 | 0.904831 |  |  |
| A_44_P295467 | 0.043711 | 0.871448 | RGD1305350 | Rn.99087 |
| A_44_P295673 | 0.011701 | 1.507903 |  | Rn.4313 |
| A_44_P296561 | 0.001905 | 1.427683 |  | Rn.12223 |
| A_44_P296631 | 0.014153 | 1.442897 | Krcc1 | Rn.7008 |
| A_44_P296698 | 0.014723 | 0.888796 | Lypla2 | Rn.8586 |
| A_44_P296859 | 0.01661 | 0.817289 |  | Rn.12128 |
| A_44_P296924 | 0.006464 | 1.211938 | Rdh10 | Rn.16968 |
| A_44_P297092 | 0.031704 | 0.854127 | Phf14 | Rn.117647 |
| A_44_P297217 | 0.009694 | 0.8326 | Sirt2 | Rn.59887 |
| A_44_P297690 | 0.002649 | 0.899503 | Aup1 | Rn.198163 |
| A_44_P298688 | 0.046214 | 1.220349 |  | Rn.106041 |
| A_44_P298709 | 0.049435 | 1.184399 |  | Rn.226935 |
| A_44_P298846 | 0.021087 | 1.376378 |  | Rn.187882 |
| A_44_P299214 | 0.010498 | 1.346808 |  |  |
| A_44_P299671 | 0.035917 | 0.8784 | Vps4b | Rn.25893 |
| A_44_P299909 | 0.00259 | 0.886726 | Wdr55 | Rn.162302 |
| A_44_P299999 | 0.022937 | 0.82812 | Ltbr | Rn.19329 |
| A_44_P300270 | 0.034038 | 0.813707 | Lztr1 | Rn.15759 |
| A_44_P300348 | 0.034048 | 0.848557 |  |  |
| A_44_P300818 | 0.014942 | 1.116068 |  | Rn.22089 |
| A_44_P301248 | 0.009211 | 1.350985 | Smek2 | Rn.35621 |
| A_44_P301494 | 0.001608 | 1.307948 |  | Rn.5802 |
| A_44_P301512 | 0.034266 | 0.831912 | G6pc3 | Rn.66254 |
| A_44_P301633 | 0.001354 | 0.866686 | Zfp191 | Rn.162286 |
| A_44_P301799 | 0.022107 | 0.819923 | Ryk | Rn.11796 |
| A_44_P301805 | 0.025387 | 0.88297 | Ryk | Rn.11796 |
| A_44_P301812 | 0.028211 | 0.835551 | Rap1a | Rn.106321 |
| A_44_P302102 | 0.0001 | 0.793772 | Idua | Rn.203375 |
| A_44_P302250 | 0.036847 | 0.946122 | Rps28 | Rn.6005 |
| A_44_P302417 | 0.009057 | 0.828972 | Setd5 | Rn.49964 |
| A_44_P302676 | 0.000638 | 1.914682 |  |  |
| A_44_P302972 | 0.002131 | 1.319409 |  |  |
| A_44_P303651 | 0.005827 | 0.854457 | Wnk1 | Rn.27409 |
| A_44_P303858 | 0.014323 | 0.878804 | Fam160b1 | Rn.162541 |
| A_44_P303933 | 0.03058 | 1.120097 |  | Rn.161973 |
| A_44_P304043 | 0.000715 | 0.806617 | Dyt1 | Rn.20041 |
| A_44_P304277 | 0.007918 | 1.293983 | Lhx3 | Rn.198623 |
| A_44_P304323 | 0.049179 | 0.762113 | Ccl5 | Rn.8019 |
| A_44_P304374 | 0.005604 | 1.127285 | Zfp422 | Rn.105961 |
| A_44_P304408 | 0.041051 | 0.920208 | Dctn3 | Rn.2038 |
| A_44_P304560 | 0.000322 | 1.878463 | RGD1565093 | |
| A_44_P304752 | 0.002909 | 1.676364 |  |  |
| A_44_P304759 | 0.035965 | 1.724998 |  |  |
| A_44_P304825 | 0.003044 | 1.985666 |  |  |
| A_44_P304873 | 0.039029 | 0.835699 | Toe1 | Rn.105772 |
| A_44_P305027 | 0.003263 | 1.296454 |  |  |
| A_44_P305689 | 0.001625 | 1.675298 | Lap3 | Rn.99790 |
| A_44_P305694 | 0.047646 | 1.35806 |  | Rn.81052 |
| A_44_P306586 | 0.030421 | 1.657337 | Hbe2 | Rn.215837 |
| A_44_P306863 | 0.001752 | 1.100148 |  |  |
| A_44_P307029 | 0.008086 | 0.743027 | Terf2ip | Rn.2055 |
| A_44_P307106 | 0.017632 | 0.790182 |  | Rn.69389 |
| A_44_P307308 | 0.000433 | 2.167049 | LOC301725 | |
| A_44_P308181 | 0.043396 | 0.838339 | Gapvd1 | Rn.22293 |
| A_44_P308266 | 0.002574 | 1.257291 |  | Rn.927 |
| A_44_P308673 | 0.032325 | 0.863875 | Commd5 | Rn.24747 |
| A_44_P309013 | 0.012449 | 0.800541 | Mapt | Rn.2455 |
| A_44_P309224 | 0.000197 | 1.556465 |  |  |
| A_44_P309398 | 0.000926 | 1.250623 | Eif3j | Rn.162681 |
| A_44_P309679 | 0.020193 | 0.830294 | Pgp | Rn.4311 |
| A_44_P310495 | 0.012148 | 1.170856 |  | Rn.112585 |
| A_44_P310851 | 0.001019 | 1.341299 | Psmf1 | Rn.103325 |
| A_44_P310949 | 0.027511 | 0.793745 | Eif3d | Rn.3463 |
| A_44_P311275 | 0.044053 | 2.228054 | Olr1375 | Rn.142195 |
| A_44_P311455 | 0.020572 | 0.894445 | Nrg2 | Rn.22434 |
| A_44_P311609 | 0.042885 | 1.119995 |  | Rn.154691 |
| A_44_P311693 | 0.045453 | 1.477716 |  |  |
| A_44_P311735 | 0.045338 | 0.875577 | Zdhhc12 | Rn.95138 |
| A_44_P312309 | 0.00537 | 0.659136 | Eif3a | Rn.1644 |
| A_44_P312373 | 0.005555 | 1.440751 |  |  |
| A_44_P312484 | 0.042121 | 0.831974 | Mtmr4 | Rn.2567 |
| A_44_P312774 | 0.041635 | 1.142734 |  | Rn.9567 |
| A_44_P313022 | 0.002787 | 1.232778 |  | Rn.98819 |
| A_44_P313040 | 0.003106 | 1.157731 |  | Rn.1233 |
| A_44_P313077 | 0.003916 | 0.827246 | Usp46 | Rn.228328 |
| A_44_P313761 | 0.000397 | 0.832741 | Mgat1 | Rn.2712 |
| A_44_P313918 | 0.00238 | 0.839158 | Dcaf8 | Rn.203073 |
| A_44_P314081 | 0.01505 | 0.877566 | Sptlc1 | Rn.18996 |
| A_44_P314122 | 0.011164 | 1.933786 |  |  |
| A_44_P314152 | 0.001737 | 1.352625 |  |  |
| A_44_P314169 | 0.008618 | 0.710665 | Fuk |  |
| A_44_P314431 | 4.66E-05 | 0.714164 | Ccdc64b | Rn.83293 |
| A_44_P314506 | 0.003448 | 1.947861 |  |  |
| A_44_P314536 | 0.006802 | 1.326637 |  | Rn.1638 |
| A_44_P315022 | 0.041628 | 0.838825 | Rad23b | Rn.67042 |
| A_44_P315085 | 0.038916 | 1.354938 |  |  |
| A_44_P315106 | 0.003989 | 0.81823 | Cfl2 | Rn.101880 |
| A_44_P315183 | 0.020331 | 1.148163 |  | Rn.12133 |
| A_44_P315286 | 0.033923 | 1.084322 |  | Rn.2511 |
| A_44_P315714 | 0.004036 | 0.771735 | Asl | Rn.64591 |
| A_44_P316504 | 0.008091 | 0.752634 | Tmed9 | Rn.9386 |
| A_44_P316507 | 0.004743 | 0.568175 | Tmed9 | Rn.9386 |
| A_44_P316833 | 0.034537 | 0.698112 |  |  |
| A_44_P318029 | 0.044452 | 0.836791 | LOC689959 | Rn.9433 |
| A_44_P318103 | 0.002408 | 1.21492 | Atp9b | Rn.40336 |
| A_44_P318188 | 0.012319 | 1.177148 |  | Rn.226650 |
| A_44_P318662 | 0.033078 | 1.553463 | Tac2 | Rn.9708 |
| A_44_P318819 | 3.39E-05 | 0.866276 |  |  |
| A_44_P318828 | 0.006582 | 0.858679 | Tmem55b | Rn.37250 |
| A_44_P318883 | 0.017379 | 1.253127 | RGD1560648 | |
| A_44_P319035 | 0.006591 | 0.687267 | RGD1565367 | Rn.16045 |
| A_44_P319042 | 0.030332 | 0.834256 | Kbtbd2 | Rn.1688 |
| A_44_P319208 | 0.006479 | 0.85226 | Dpp8 | Rn.17532 |
| A_44_P319971 | 0.008444 | 0.787877 |  | Rn.203512 |
| A_44_P320376 | 0.020452 | 1.208718 |  | Rn.4085 |
| A_44_P320737 | 0.028473 | 0.740665 | Flvcr2 | Rn.92663 |
| A_44_P321488 | 0.048891 | 0.762144 | LOC100294508 | Rn.3730 |
| A_44_P321622 | 0.041028 | 0.730318 | Itfg3 | Rn.6244 |
| A_44_P321669 | 0.003659 | 0.752134 | Map3k3 | Rn.72680 |
| A_44_P321675 | 0.031027 | 0.846928 | Map3k3 | Rn.72680 |
| A_44_P321918 | 0.02791 | 0.881534 | Gramd1a | Rn.16801 |
| A_44_P322118 | 2.95E-06 | 1.630069 | Rps7 | Rn.107039 |
| A_44_P322425 | 0.0208 | 1.112173 |  | Rn.125368 |
| A_44_P322960 | 0.013134 | 0.819392 | Araf | Rn.1714 |
| A_44_P323496 | 0.005026 | 0.762585 | Inpp5d | Rn.10659 |
| A_44_P323558 | 0.008174 | 0.837224 | Ccnt2 | Rn.21407 |
| A_44_P323599 | 0.025419 | 1.153272 | RGD1562886 | |
| A_44_P323892 | 0.000111 | 2.434075 | RGD1559951 | |
| A_44_P324042 | 0.045578 | 0.794024 | Srpr | Rn.18349 |
| A_44_P324093 | 0.002067 | 0.738853 | Spryd3 | Rn.9246 |
| A_44_P324452 | 0.018824 | 0.844855 | Rragc | Rn.6455 |
| A_44_P325268 | 0.006722 | 1.142108 | Scfd2 | Rn.3326 |
| A_44_P325426 | 0.000246 | 1.306203 |  | Rn.164817 |
| A_44_P325605 | 0.001137 | 0.620302 | G7c | Rn.219144 |
| A_44_P325782 | 0.025184 | 0.812879 | Hsd3b7 | Rn.11380 |
| A_44_P325785 | 0.008979 | 1.204176 |  |  |
| A_44_P325791 | 0.030408 | 1.127921 | Ywhag | Rn.29936 |
| A_44_P325841 | 0.047044 | 0.759935 | Dctn1 | Rn.11284 |
| A_44_P326198 | 0.045572 | 0.876834 | Atp6v0d1 | Rn.94752 |
| A_44_P326259 | 0.002784 | 0.752139 | Iffo1 | Rn.38932 |
| A_44_P326350 | 0.03738 | 0.833983 | Wdr8 | Rn.30003 |
| A_44_P326584 | 0.010122 | 1.251604 |  |  |
| A_44_P328323 | 0.008037 | 0.885547 | Usp46 | Rn.228328 |
| A_44_P328664 | 0.000439 | 1.339872 |  |  |
| A_44_P328701 | 0.017928 | 1.41285 | LOC680646 | |
| A_44_P329577 | 0.004296 | 0.772395 | Arl2bp | Rn.985 |
| A_44_P330643 | 0.029497 | 0.790976 | Polr2b | Rn.153952 |
| A_44_P330765 | 0.022496 | 0.774295 | Cdk20 | Rn.7013 |
| A_44_P330931 | 0.01152 | 0.825405 | Prpf4 | Rn.162812 |
| A_44_P332021 | 0.003372 | 1.224091 | S100a4 | Rn.504 |
| A_44_P332041 | 0.0343 | 0.858886 | Arl1 | Rn.3065 |
| A_44_P332694 | 0.01567 | 0.816147 | Guk1 | Rn.203102 |
| A_44_P332928 | 0.0328 | 1.128208 | RGD1306820 | Rn.102186 |
| A_44_P332971 | 0.011843 | 1.176467 |  |  |
| A_44_P333329 | 0.001848 | 0.767 | Rexo1 | Rn.12613 |
| A_44_P333495 | 0.000152 | 1.305099 |  |  |
| A_44_P334166 | 0.01025 | 1.142752 |  | Rn.118772 |
| A_44_P334195 | 0.004459 | 0.809781 | Qars | Rn.98697 |
| A_44_P334258 | 6.63E-05 | 1.164403 |  | Rn.101781 |
| A_44_P334494 | 0.044507 | 1.155442 |  | Rn.170015 |
| A_44_P335165 | 0.00693 | 0.852028 | Ddx6 | Rn.228431 |
| A_44_P335247 | 0.038885 | 0.866366 | Fam18b2 | Rn.75470 |
| A_44_P335377 | 0.007545 | 0.751091 | Fbxw5 | Rn.136952 |
| A_44_P335810 | 0.037118 | 1.157547 |  | Rn.21 |
| A_44_P336054 | 0.000147 | 1.868844 |  |  |
| A_44_P336148 | 0.006216 | 0.809968 |  | Rn.16559 |
| A_44_P337865 | 0.000664 | 2.653849 |  |  |
| A_44_P338002 | 0.008381 | 0.783872 |  |  |
| A_44_P338743 | 0.00023 | 1.224752 |  | Rn.103770 |
| A_44_P339361 | 0.010859 | 1.190219 |  | Rn.9014 |
| A_44_P339558 | 0.031988 | 0.875973 | Myo5b | Rn.10640 |
| A_44_P339757 | 0.010397 | 0.763649 |  | Rn.134436 |
| A_44_P340159 | 0.012298 | 0.792703 |  |  |
| A_44_P340241 | 0.000206 | 2.725365 |  |  |
| A_44_P340329 | 0.003253 | 0.880895 | Pum2 | Rn.8622 |
| A_44_P340357 | 0.002165 | 0.822579 | Rnf113a2 | Rn.18838 |
| A_44_P340401 | 0.000892 | 2.307254 |  |  |
| A_44_P340915 | 0.00057 | 0.859124 | Pdzd8 | Rn.24878 |
| A_44_P340977 | 0.022871 | 0.872892 |  |  |
| A_44_P341428 | 0.039986 | 0.930595 | Tfip11 | Rn.161710 |
| A_44_P341456 | 0.013662 | 1.627344 | Peli1 | Rn.22814 |
| A_44_P342051 | 0.022664 | 0.779582 | Ecm1 | Rn.97792 |
| A_44_P342117 | 0.002818 | 0.848175 | Wbp2 | Rn.198920 |
| A_44_P342202 | 0.023163 | 0.82876 | Cask | Rn.72627 |
| A_44_P342208 | 0.020778 | 0.868101 |  |  |
| A_44_P342372 | 0.027621 | 1.828784 | Hapln4 | Rn.98352 |
| A_44_P342397 | 0.049744 | 0.824824 | Polr3gl | Rn.86210 |
| A_44_P342412 | 0.030854 | 0.863934 | Abhd13 | Rn.6621 |
| A_44_P342416 | 0.000606 | 2.929685 |  |  |
| A_44_P342568 | 0.016813 | 1.977289 |  |  |
| A_44_P342657 | 0.000288 | 0.758388 | Vps13d | Rn.2644 |
| A_44_P342671 | 0.00921 | 1.535914 |  |  |
| A_44_P342756 | 0.01416 | 2.686868 |  |  |
| A_44_P342792 | 0.00343 | 0.756613 | Wdr81 | Rn.163168 |
| A_44_P342966 | 0.016195 | 0.853468 | Rwdd3 | Rn.54470 |
| A_44_P343445 | 0.005775 | 1.110565 |  | Rn.2 |
| A_44_P343716 | 0.033996 | 1.1252 |  | Rn.4057 |
| A_44_P343797 | 0.002814 | 0.807588 | Ubxn4 | Rn.2022 |
| A_44_P343843 | 0.016192 | 1.093184 |  | Rn.107980 |
| A_44_P343859 | 0.022126 | 1.622085 | Csf1 | Rn.83632 |
| A_44_P343888 | 0.00226 | 1.476602 |  | Rn.98833 |
| A_44_P344096 | 0.042762 | 0.702629 | Ehd3 | Rn.7771 |
| A_44_P344181 | 0.046255 | 0.809605 | Map6 | Rn.37490 |
| A_44_P344273 | 0.035103 | 0.82527 | Sec62 | Rn.98327 |
| A_44_P344340 | 0.030111 | 0.871566 | Zfoc1 | Rn.198302 |
| A_44_P344391 | 0.000296 | 0.765231 | Bles03 | Rn.7405 |
| A_44_P344397 | 0.004584 | 0.795008 | Bles03 | Rn.7405 |
| A_44_P344421 | 0.044075 | 0.822545 | Dock9 | Rn.10431 |
| A_44_P344562 | 0.041066 | 0.927687 |  |  |
| A_44_P344777 | 0.003839 | 3.56082 |  |  |
| A_44_P344969 | 0.035605 | 1.143145 | LOC100364111 | |
| A_44_P344999 | 0.008938 | 1.720376 | Mphosph6 | |
| A_44_P345009 | 0.018127 | 0.815224 | Bag5 | Rn.76368 |
| A_44_P345013 | 0.015063 | 0.814228 | Slc44a2 | Rn.140320 |
| A_44_P345476 | 0.047122 | 0.883316 | Myh9 | Rn.11385 |
| A_44_P346301 | 0.00164 | 1.171065 |  | Rn.3464 |
| A_44_P346782 | 0.020934 | 0.761422 | Ncdn | Rn.5653 |
| A_44_P346822 | 0.012483 | 0.765947 | Orai2 | Rn.18207 |
| A_44_P346832 | 0.025017 | 0.646439 | Tesc | Rn.198314 |
| A_44_P347348 | 0.046089 | 1.143972 | Ttc35 | Rn.203197 |
| A_44_P347420 | 0.031981 | 0.836649 | Usp43_predicted | Rn.219475 |
| A_44_P347482 | 0.00478 | 1.223908 |  | Rn.89331 |
| A_44_P347938 | 0.0004 | 1.317006 | Grinl1a | Rn.144666 |
| A_44_P348527 | 0.006482 | 1.159748 |  | Rn.167678 |
| A_44_P348607 | 0.012252 | 0.873729 | Gtf3c2 | Rn.22450 |
| A_44_P348642 | 0.029289 | 1.294304 | Rbm33 | Rn.23554 |
| A_44_P348719 | 0.040038 | 1.258001 |  | Rn.49925 |
| A_44_P349250 | 0.002454 | 0.774771 | Paf1 | Rn.161558 |
| A_44_P349320 | 0.004538 | 0.664935 | Mxd4 | Rn.3279 |
| A_44_P349356 | 0.004131 | 0.807539 | Tnpo1 | Rn.223634 |
| A_44_P349525 | 0.003369 | 1.248505 |  |  |
| A_44_P349539 | 0.044493 | 0.831626 | Elmo2 | Rn.2097 |
| A_44_P349769 | 1.15E-05 | 1.691718 |  |  |
| A_44_P350779 | 0.016899 | 1.178088 |  | Rn.266 |
| A_44_P350843 | 0.018038 | 0.798003 | Erlec1 | Rn.102123 |
| A_44_P351032 | 0.016119 | 1.151235 |  | Rn.220230 |
| A_44_P351452 | 0.023386 | 0.604212 | Bmp1 | Rn.9305 |
| A_44_P351627 | 0.003588 | 2.735874 |  |  |
| A_44_P352973 | 0.044398 | 1.121326 |  | Rn.3660 |
| A_44_P353212 | 0.025246 | 1.164091 |  | Rn.65040 |
| A_44_P353729 | 0.009968 | 0.728161 | Axl | Rn.161805 |
| A_44_P354053 | 0.034305 | 0.882349 | Gdap2 | Rn.24196 |
| A_44_P354534 | 0.035631 | 1.541009 |  | Rn.1215 |
| A_44_P355221 | 0.001873 | 1.303808 |  | Rn.219229 |
| A_44_P355657 | 0.042539 | 0.913171 | Pi4ka | Rn.11015 |
| A_44_P356538 | 0.007468 | 0.82126 | Zfp330 | Rn.137414 |
| A_44_P356561 | 0.00236 | 1.721655 |  |  |
| A_44_P356707 | 0.037419 | 1.194944 |  | Rn.12521 |
| A_44_P358216 | 0.024692 | 0.914845 | Pank4 | Rn.17321 |
| A_44_P358306 | 0.000278 | 1.642663 | Olr193 | Rn.141817 |
| A_44_P358358 | 0.002537 | 1.547798 | RT1-T18 | Rn.96971 |
| A_44_P358712 | 0.014654 | 1.485516 |  |  |
| A_44_P358797 | 0.000173 | 1.193825 |  |  |
| A_44_P358807 | 0.006466 | 0.745039 | Surf6 | Rn.124784 |
| A_44_P358828 | 0.021403 | 0.791716 | Pcdhb13 | Rn.45529 |
| A_44_P359242 | 0.01349 | 1.151458 |  | Rn.2762 |
| A_44_P359869 | 0.019179 | 1.140647 |  | Rn.104497 |
| A_44_P359925 | 0.026923 | 0.760146 |  | Rn.3562 |
| A_44_P360035 | 0.007874 | 0.924689 | Cops4 | Rn.7506 |
| A_44_P360126 | 0.001712 | 1.412454 |  |  |
| A_44_P360946 | 0.017123 | 1.11938 |  |  |
| A_44_P361124 | 0.044571 | 0.764269 |  |  |
| A_44_P361857 | 0.028875 | 1.228573 |  | Rn.18066 |
| A_44_P362380 | 0.030075 | 0.813388 | RGD1559612 | Rn.18093 |
| A_44_P362723 | 0.015563 | 1.157384 | Psmd7 | Rn.20659 |
| A_44_P363252 | 0.009507 | 1.191375 | Set | Rn.76937 |
| A_44_P363494 | 0.047448 | 1.313292 |  |  |
| A_44_P363522 | 0.00018 | 0.730659 | B4galt7 | Rn.41210 |
| A_44_P363525 | 0.018633 | 1.536482 |  |  |
| A_44_P363597 | 0.017475 | 0.788047 | Raly | Rn.162714 |
| A_44_P363814 | 0.009045 | 0.760539 | Sun2 | Rn.2240 |
| A_44_P363824 | 0.000137 | 0.859819 | Brd1 | Rn.9133 |
| A_44_P363847 | 0.001177 | 0.747399 | Fam100a | Rn.94991 |
| A_44_P363882 | 0.037097 | 0.833147 | Myo18a | Rn.106278 |
| A_44_P363913 | 0.000166 | 3.954509 |  |  |
| A_44_P363937 | 0.001401 | 1.72304 |  | Rn.124542 |
| A_44_P364660 | 0.015443 | 1.378504 |  | Rn.162909 |
| A_44_P365462 | 0.043472 | 0.669824 | Cacna1d | Rn.89671 |
| A_44_P365598 | 0.015706 | 0.861751 | Rnf146 | Rn.16849 |
| A_44_P365879 | 0.015323 | 1.23284 |  |  |
| A_44_P366118 | 0.028799 | 0.745985 |  |  |
| A_44_P367243 | 0.020037 | 1.475141 | Ypel5 | Rn.1136 |
| A_44_P367556 | 0.007134 | 0.890392 | Sqstm1 | Rn.107103 |
| A_44_P367688 | 0.003807 | 0.689634 | RT1-Da | Rn.103146 |
| A_44_P368032 | 0.024508 | 0.901825 | Polr3a | Rn.41904 |
| A_44_P368263 | 0.005653 | 1.107457 |  |  |
| A_44_P368365 | 0.022797 | 1.191152 |  |  |
| A_44_P368406 | 0.015726 | 0.884094 | Ctdsp1 | Rn.163070 |
| A_44_P368703 | 0.047817 | 0.879119 |  | Rn.1822 |
| A_44_P369617 | 0.004221 | 1.217087 |  | Rn.219234 |
| A_44_P369701 | 0.040059 | 0.838226 | Ei24 | Rn.6593 |
| A_44_P370281 | 0.017346 | 0.842333 | Herc2 | Rn.28217 |
| A_44_P370328 | 0.032436 | 1.2695 | Ric8a | Rn.94796 |
| A_44_P370385 | 0.038457 | 1.160499 | Dnajc15 | Rn.8092 |
| A_44_P370637 | 0.010007 | 1.14106 | RGD1562265 | |
| A_44_P371552 | 0.012385 | 0.913457 | Pcbp2 | Rn.106644 |
| A_44_P372061 | 0.020167 | 0.79195 | Cyp2d4 | Rn.26060 |
| A_44_P372377 | 0.003983 | 1.276114 | Olr1439 | Rn.142135 |
| A_44_P372484 | 0.003891 | 0.77949 |  |  |
| A_44_P372555 | 0.02761 | 0.882985 | Kdm3a | Rn.10541 |
| A_44_P373285 | 0.000193 | 2.164775 |  | Rn.2894 |
| A_44_P374011 | 0.008887 | 0.887965 | Eif2d | Rn.162387 |
| A_44_P374204 | 0.000744 | 1.209007 |  | Rn.41724 |
| A_44_P374618 | 0.048733 | 0.778944 | Cdkn1c | Rn.162507 |
| A_44_P374620 | 0.043414 | 0.796311 | Cdkn1c | Rn.162507 |
| A_44_P375158 | 0.01133 | 0.892484 |  |  |
| A_44_P375252 | 0.002108 | 0.818069 | Ehmt1 | Rn.7645 |
| A_44_P375322 | 0.000995 | 0.843126 | Trpc4ap | Rn.100109 |
| A_44_P375613 | 0.001702 | 0.74276 | Narfl | Rn.14772 |
| A_44_P377355 | 0.006678 | 1.474228 |  |  |
| A_44_P377520 | 0.004422 | 1.258738 |  |  |
| A_44_P377722 | 0.000145 | 0.842694 | Pef1 | Rn.96097 |
| A_44_P377731 | 4.39E-05 | 0.811866 | Pef1 | Rn.96097 |
| A_44_P378118 | 0.013032 | 1.392542 |  | Rn.164556 |
| A_44_P378383 | 0.002227 | 0.733168 | Rnf26 | Rn.15010 |
| A_44_P378962 | 0.023749 | 1.108297 |  | Rn.30672 |
| A_44_P379331 | 0.010399 | 0.897084 | LOC257642 | |
| A_44_P379420 | 0.006396 | 0.835748 | Gdi1 | Rn.4000 |
| A_44_P379751 | 0.03714 | 0.792007 | Cux1 |  |
| A_44_P379903 | 0.00881 | 1.718599 |  |  |
| A_44_P380031 | 0.000988 | 0.754948 | Tom1 | Rn.198076 |
| A_44_P380142 | 0.00034 | 1.181867 |  |  |
| A_44_P380329 | 0.004547 | 2.392811 |  |  |
| A_44_P380392 | 0.014104 | 0.831587 | Slc38a10 | Rn.18664 |
| A_44_P380397 | 0.00662 | 0.845501 | Slc38a10 | Rn.18664 |
| A_44_P381781 | 0.001761 | 0.796145 | Gpank1 | Rn.203143 |
| A_44_P382318 | 0.035205 | 1.159519 | Cdk14 | Rn.12908 |
| A_44_P382349 | 0.000387 | 3.334831 |  |  |
| A_44_P382438 | 0.027573 | 0.9007 | Supt7l | Rn.11526 |
| A_44_P382611 | 0.028199 | 0.881068 | Tmub2 | Rn.143886 |
| A_44_P382696 | 0.042602 | 1.154253 |  | Rn.224571 |
| A_44_P383231 | 0.000532 | 1.24187 |  | Rn.12038 |
| A_44_P383235 | 0.00651 | 1.226081 |  | Rn.47249 |
| A_44_P383825 | 0.005702 | 0.839484 | Calu | Rn.1841 |
| A_44_P384086 | 0.016821 | 1.254976 | Kcnc3 | Rn.9885 |
| A_44_P384112 | 0.043167 | 0.922327 | Ppp3cc | Rn.22079 |
| A_44_P384304 | 0.018848 | 1.148352 |  |  |
| A_44_P384462 | 7.06E-05 | 1.659548 | RGD1565713 | |
| A_44_P384688 | 0.004227 | 1.715811 |  |  |
| A_44_P384698 | 0.008979 | 2.264104 |  |  |
| A_44_P384772 | 0.029491 | 0.902665 | Eri3 | Rn.228475 |
| A_44_P384910 | 0.014508 | 0.783937 |  |  |
| A_44_P384937 | 0.001443 | 0.747098 | Mterfd2 | Rn.41249 |
| A_44_P384979 | 0.03784 | 0.748792 | Trub2 | Rn.105581 |
| A_44_P386164 | 0.04315 | 1.201823 | Marcks | Rn.163679 |
| A_44_P386187 | 0.001654 | 1.127387 |  | Rn.151653 |
| A_44_P386225 | 0.009516 | 1.233609 |  | Rn.2379 |
| A_44_P386734 | 0.046959 | 0.840277 | Trrap | Rn.92036 |
| A_44_P386799 | 0.005823 | 2.080906 | Nudt9 | Rn.22103 |
| A_44_P387067 | 0.019479 | 1.199636 |  |  |
| A_44_P387213 | 0.047584 | 1.31232 |  |  |
| A_44_P387308 | 0.047017 | 0.784519 | Adamtsl4 | Rn.62115 |
| A_44_P388040 | 0.006119 | 1.370595 | Pkd1 | Rn.127770 |
| A_44_P388170 | 0.000484 | 1.221565 |  | Rn.147976 |
| A_44_P388174 | 0.007687 | 1.186264 |  | Rn.147976 |
| A_44_P389142 | 0.007228 | 0.748741 | Mesdc1 | Rn.162446 |
| A_44_P389243 | 0.009086 | 1.230969 |  |  |
| A_44_P389283 | 0.014735 | 0.605481 |  |  |
| A_44_P389400 | 0.020843 | 0.859565 | Tp53rk | Rn.19574 |
| A_44_P389688 | 0.006733 | 0.729948 | Dcaf7 | Rn.3104 |
| A_44_P391360 | 0.01981 | 0.856052 | Zhx1 | Rn.16902 |
| A_44_P391596 | 0.01608 | 0.801128 | Tbc1d7 | Rn.13671 |
| A_44_P391599 | 0.000989 | 0.763331 | Tbc1d7 | Rn.13671 |
| A_44_P391680 | 0.000532 | 0.768972 | Tnpo2 | Rn.228319 |
| A_44_P391902 | 0.007186 | 1.151 |  |  |
| A_44_P391915 | 0.000217 | 3.213336 |  |  |
| A_44_P392611 | 0.048734 | 0.852508 |  | Rn.17773 |
| A_44_P392890 | 0.026211 | 1.183223 |  | Rn.120398 |
| A_44_P393007 | 0.000403 | 1.190014 |  | Rn.178791 |
| A_44_P393442 | 0.003454 | 0.75735 | Map6 | Rn.37490 |
| A_44_P393731 | 0.001118 | 0.719424 | Cdc42bpg | Rn.99897 |
| A_44_P393835 | 0.03456 | 1.11663 |  |  |
| A_44_P393929 | 0.016784 | 0.864686 | Fto | Rn.11858 |
| A_44_P393985 | 0.004328 | 3.243841 |  |  |
| A_44_P394016 | 0.023046 | 1.086286 | RGD1565054 | |
| A_44_P394042 | 0.00372 | 0.839592 | Itsn2 | Rn.37673 |
| A_44_P394636 | 0.00073 | 0.791515 | Inpp5d | Rn.10659 |
| A_44_P395421 | 0.040366 | 0.926402 |  | Rn.224663 |
| A_44_P395425 | 0.025057 | 0.901475 |  | Rn.224663 |
| A_44_P395950 | 0.022383 | 1.252898 |  |  |
| A_44_P395975 | 0.000748 | 0.845602 | Ndnl2 | Rn.9406 |
| A_44_P395977 | 0.004532 | 0.854483 | Ndnl2 | Rn.9406 |
| A_44_P396198 | 0.0498 | 0.846451 | Celf1 | Rn.22432 |
| A_44_P396249 | 0.020593 | 0.89453 |  |  |
| A_44_P396327 | 0.000513 | 1.782166 |  |  |
| A_44_P396546 | 0.005029 | 2.132931 | Hspa8 | Rn.201298 |
| A_44_P397441 | 0.040278 | 1.092441 | Eif1 | Rn.137199 |
| A_44_P397460 | 0.011909 | 1.40738 |  | Rn.4148 |
| A_44_P397736 | 0.027587 | 1.234821 | Ube2s | Rn.120398 |
| A_44_P398300 | 0.000168 | 1.268228 | Fam160a2 | Rn.23025 |
| A_44_P398368 | 0.001404 | 1.642101 |  |  |
| A_44_P398631 | 0.001894 | 1.156643 |  |  |
| A_44_P398792 | 6.17E-05 | 2.405495 | Tpt1 | Rn.36610 |
| A_44_P398864 | 0.025539 | 0.85836 | Cog1 | Rn.163170 |
| A_44_P398969 | 0.04179 | 0.880377 | Gtpbp2 | Rn.3595 |
| A_44_P399044 | 0.010772 | 0.827886 |  | Rn.7745 |
| A_44_P399459 | 0.030918 | 0.735562 | Col4a3bp | Rn.105704 |
| A_44_P400223 | 0.009794 | 1.110098 | Zmynd11 | Rn.38301 |
| A_44_P400520 | 0.01404 | 0.866633 | Nfyc | Rn.1457 |
| A_44_P400591 | 0.039669 | 1.433079 |  |  |
| A_44_P400718 | 0.011441 | 1.150726 |  |  |
| A_44_P400893 | 0.015751 | 0.860402 | Tmem168 | Rn.7056 |
| A_44_P400936 | 0.007403 | 0.859384 |  |  |
| A_44_P401074 | 0.036241 | 1.150646 | Dcaf13 | Rn.41457 |
| A_44_P401809 | 0.023204 | 0.918151 | Rps20 | Rn.103133 |
| A_44_P402028 | 0.012257 | 1.134791 |  | Rn.167053 |
| A_44_P402425 | 0.045883 | 1.205096 | Bwk1 | Rn.28608 |
| A_44_P402664 | 0.040471 | 0.874597 | Fbxo6 | Rn.57325 |
| A_44_P402668 | 0.001988 | 0.801471 | Fbxo6 | Rn.57325 |
| A_44_P402884 | 0.037287 | 0.778157 | Pitpnm2 | Rn.75968 |
| A_44_P402995 | 0.003448 | 1.262063 |  |  |
| A_44_P403017 | 0.044292 | 1.558102 |  |  |
| A_44_P403065 | 2.23E-05 | 1.752157 |  |  |
| A_44_P403396 | 0.045588 | 0.761633 | Snx33 | Rn.43176 |
| A_44_P403978 | 0.000759 | 1.416589 | Supt4h1 | Rn.7906 |
| A_44_P404161 | 0.044551 | 0.914361 |  | Rn.17226 |
| A_44_P404180 | 0.029966 | 1.113528 |  | Rn.103770 |
| A_44_P404198 | 0.034226 | 0.884548 | Mapk3 | Rn.2592 |
| A_44_P404320 | 0.005407 | 2.123785 |  |  |
| A_44_P404737 | 0.017961 | 1.309302 |  | Rn.205491 |
| A_44_P404836 | 0.048738 | 1.495288 | Cp | Rn.32777 |
| A_44_P405074 | 8.54E-05 | 2.558321 |  |  |
| A_44_P405321 | 0.013356 | 2.045506 |  |  |
| A_44_P405443 | 0.034211 | 0.796237 | Manba | Rn.20578 |
| A_44_P405704 | 7.79E-05 | 1.568667 |  |  |
| A_44_P406493 | 0.00492 | 1.183933 |  | Rn.3126 |
| A_44_P406654 | 0.015963 | 1.101173 |  |  |
| A_44_P406775 | 0.000566 | 1.766013 | Dalrd3 | Rn.120770 |
| A_44_P406874 | 0.011122 | 1.148082 |  | Rn.99661 |
| A_44_P407507 | 0.026497 | 0.788364 | Klc1 | Rn.106230 |
| A_44_P407954 | 0.010651 | 1.249013 |  |  |
| A_44_P408011 | 0.004472 | 1.153827 | Srsf10 | Rn.162118 |
| A_44_P408108 | 0.023483 | 0.833731 |  | Rn.73847 |
| A_44_P408928 | 0.011865 | 1.131366 |  | Rn.71883 |
| A_44_P409132 | 9.91E-05 | 1.20326 |  | Rn.112639 |
| A_44_P409146 | 0.039863 | 1.03879 |  | Rn.93735 |
| A_44_P409696 | 0.017216 | 0.894165 | Raf1 | Rn.33262 |
| A_44_P409797 | 0.014899 | 0.852154 | Ppp5c | Rn.6107 |
| A_44_P409835 | 0.02777 | 0.623145 | Plvap | Rn.53915 |
| A_44_P410033 | 0.042186 | 1.147012 | Wdr41 | Rn.146022 |
| A_44_P410192 | 0.044351 | 0.868628 | Dtd1 | Rn.23561 |
| A_44_P410272 | 0.03076 | 1.086013 | Lamtor1 | Rn.121836 |
| A_44_P410331 | 0.000934 | 1.795671 | Amd1-ps2 | Rn.58708 |
| A_44_P412200 | 0.007107 | 2.52295 |  |  |
| A_44_P412215 | 0.029018 | 0.682932 | Spn | Rn.11144 |
| A_44_P412349 | 0.00321 | 1.95044 |  |  |
| A_44_P412724 | 0.039647 | 0.778676 | Usp45 | Rn.147597 |
| A_44_P412749 | 0.009524 | 1.299818 | LOC301124 | Rn.4199 |
| A_44_P413112 | 0.04407 | 0.897357 | Usf2 | Rn.44637 |
| A_44_P413540 | 0.024269 | 0.82999 |  | Rn.6583 |
| A_44_P414087 | 0.017903 | 1.276237 |  | Rn.4294 |
| A_44_P414345 | 0.010544 | 1.324763 | Olr559 | Rn.142904 |
| A_44_P414615 | 0.003184 | 0.889279 | Daxx | Rn.870 |
| A_44_P414700 | 8.54E-05 | 1.68273 |  |  |
| A_44_P414838 | 0.001524 | 2.648131 | RGD1561841 | |
| A_44_P415003 | 0.042184 | 0.814838 | Rpusd3 | Rn.45622 |
| A_44_P415199 | 0.031166 | 0.801732 | Tubgcp6 | Rn.24182 |
| A_44_P415337 | 0.000212 | 1.294549 |  | Rn.164561 |
| A_44_P415349 | 0.004762 | 1.329888 | Gata6 | Rn.8701 |
| A_44_P415459 | 0.008478 | 0.781888 |  | Rn.161999 |
| A_44_P416005 | 0.001959 | 1.501596 |  | Rn.270 |
| A_44_P416477 | 0.039903 | 0.837437 | Uqcrc1 | Rn.3428 |
| A_44_P416539 | 0.013488 | 0.846749 |  | Rn.186983 |
| A_44_P416540 | 0.006577 | 0.830226 |  | Rn.186983 |
| A_44_P416596 | 0.00415 | 0.791917 | Pdp2 | Rn.30021 |
| A_44_P417083 | 0.034256 | 1.217938 | Atad1 | Rn.54918 |
| A_44_P417267 | 0.010293 | 0.724457 | Thap11 | Rn.2823 |
| A_44_P417317 | 0.022292 | 0.917973 | Ddx47 | Rn.73790 |
| A_44_P418320 | 0.013122 | 0.864273 |  | Rn.64440 |
| A_44_P418672 | 0.049041 | 1.124545 |  | Rn.10 |
| A_44_P418675 | 0.025727 | 1.10623 |  | Rn.10 |
| A_44_P418753 | 0.002538 | 0.894382 |  | Rn.64225 |
| A_44_P419239 | 0.00951 | 1.296373 | Pdap1 | Rn.6158 |
| A_44_P419337 | 0.006609 | 0.850951 | Kdelr2 | Rn.4066 |
| A_44_P419948 | 0.015285 | 1.150135 | Calcoco2 |  |
| A_44_P420005 | 0.040839 | 0.852993 | Ormdl3 | Rn.202345 |
| A_44_P420614 | 0.018457 | 1.192742 |  | Rn.2778 |
| A_44_P420946 | 0.03043 | 1.21881 | Armc8 | Rn.8146 |
| A_44_P421425 | 0.024758 | 1.306035 | Olr309 | Rn.141729 |
| A_44_P421565 | 0.026149 | 0.723797 | Kcnk6 | Rn.43365 |
| A_44_P422006 | 6.29E-05 | 1.234393 |  |  |
| A_44_P422271 | 0.036538 | 0.78874 | Cerk | Rn.99537 |
| A_44_P422696 | 0.030416 | 1.27037 | Scarb1 | Rn.88169 |
| A_44_P423004 | 0.013583 | 0.897586 | Ube2v1 | Rn.220168 |
| A_44_P423084 | 0.008493 | 0.796273 | Mllt10 | Rn.3620 |
| A_44_P423600 | 0.021053 | 1.309023 | Phrf1 | Rn.10530 |
| A_44_P423786 | 0.005242 | 0.915834 | Eef1g | Rn.43300 |
| A_44_P424076 | 0.041943 | 0.683875 | Dhdh | Rn.225221 |
| A_44_P424092 | 0.00345 | 0.86175 | Brox | Rn.3660 |
| A_44_P424164 | 0.000162 | 2.432967 |  |  |
| A_44_P424440 | 7.72E-05 | 2.011042 | LOC291480 | |
| A_44_P424635 | 0.039347 | 0.762106 | Ikzf3 | Rn.218596 |
| A_44_P424665 | 0.018599 | 1.315713 |  |  |
| A_44_P425027 | 0.004094 | 1.167323 |  | Rn.97792 |
| A_44_P425324 | 0.03195 | 1.220546 |  | Rn.62267 |
| A_44_P425481 | 0.013945 | 1.104866 |  | Rn.15451 |
| A_44_P425754 | 0.010082 | 1.141852 |  | Rn.18445 |
| A_44_P425848 | 0.018962 | 1.279733 | Rassf3 | Rn.3702 |
| A_44_P425906 | 0.020342 | 1.111751 |  | Rn.23677 |
| A_44_P426004 | 0.003618 | 0.764206 |  | Rn.7685 |
| A_44_P426102 | 0.015388 | 0.812259 | 15-Sep | Rn.1124 |
| A_44_P426307 | 0.006066 | 1.313636 |  | Rn.158987 |
| A_44_P426387 | 0.008868 | 0.893208 | Snap29 | Rn.17902 |
| A_44_P426478 | 0.013439 | 0.885424 |  |  |
| A_44_P426498 | 0.00261 | 1.466438 | RGD1564617 | |
| A_44_P426764 | 0.00205 | 1.756776 |  |  |
| A_44_P426788 | 0.011959 | 0.755325 | Pmepa1 | Rn.28828 |
| A_44_P426914 | 0.00365 | 1.235704 | Ubxn2a | Rn.225043 |
| A_44_P427774 | 0.037115 | 0.811742 |  | Rn.161296 |
| A_44_P428230 | 0.027352 | 1.183897 |  | Rn.202987 |
| A_44_P428232 | 0.023271 | 1.26985 |  | Rn.202987 |
| A_44_P428233 | 0.023732 | 1.34277 |  | Rn.202987 |
| A_44_P428863 | 0.001209 | 0.793859 | Fbxl14 | Rn.225319 |
| A_44_P428988 | 0.007217 | 1.900252 |  |  |
| A_44_P429304 | 0.037753 | 1.144821 |  |  |
| A_44_P429354 | 4.64E-06 | 2.356744 |  |  |
| A_44_P429364 | 0.01604 | 0.84536 | Ptdss1 | Rn.103732 |
| A_44_P429379 | 0.000641 | 2.274276 | RGD1564268 | |
| A_44_P429483 | 0.004048 | 0.745371 | RGD1308154 | Rn.17212 |
| A_44_P429694 | 0.015471 | 0.894294 |  | Rn.16713 |
| A_44_P430044 | 0.000152 | 1.668355 |  | Rn.3408 |
| A_44_P430359 | 0.045475 | 1.147305 |  | Rn.167943 |
| A_44_P430365 | 0.001399 | 1.292363 |  | Rn.102412 |
| A_44_P430581 | 0.016261 | 0.83742 | Nsmce4a | Rn.8649 |
| A_44_P430604 | 0.047755 | 1.11036 |  | Rn.1221 |
| A_44_P430937 | 0.00206 | 0.729596 | Ptpro | Rn.10163 |
| A_44_P431053 | 0.002347 | 0.688822 | Dpyd | Rn.158382 |
| A_44_P431092 | 0.010774 | 0.748352 | Cyp2d4 | Rn.26060 |
| A_44_P431112 | 0.034917 | 0.807659 |  | Rn.162082 |
| A_44_P431488 | 0.000668 | 1.401545 |  |  |
| A_44_P431771 | 0.00548 | 1.233781 |  | Rn.1423 |
| A_44_P432419 | 0.002613 | 0.843126 |  | Rn.198429 |
| A_44_P432470 | 0.004314 | 1.194348 |  | Rn.100909 |
| A_44_P432826 | 0.040233 | 1.357398 | Rap1gds1 | Rn.160647 |
| A_44_P433349 | 0.04226 | 1.251515 | Cdk5rap2 | Rn.13247 |
| A_44_P433383 | 0.000711 | 1.307181 |  |  |
| A_44_P433579 | 0.017535 | 0.846322 | Fam175a | Rn.106875 |
| A_44_P433771 | 0.040971 | 0.939407 | Reep5 | Rn.40477 |
| A_44_P433865 | 0.044042 | 0.803361 | Xpc | Rn.22820 |
| A_44_P433894 | 0.016404 | 1.139636 | RGD1560821 | Rn.224534 |
| A_44_P434109 | 0.004849 | 1.268411 | Hes7 | Rn.218470 |
| A_44_P434118 | 0.03212 | 2.054167 |  |  |
| A_44_P435149 | 0.010802 | 1.17763 |  | Rn.144669 |
| A_44_P435197 | 0.023532 | 1.122371 |  | Rn.127811 |
| A_44_P435422 | 1.48E-05 | 2.49246 | Mef2a | Rn.162435 |
| A_44_P435596 | 0.031357 | 3.770849 | Zfp36 | Rn.82737 |
| A_44_P435867 | 0.00284 | 1.551954 | Atp5hl1 |  |
| A_44_P436063 | 0.001442 | 2.096264 |  |  |
| A_44_P436078 | 0.049599 | 0.849398 | Cutc | Rn.13248 |
| A_44_P436123 | 0.01615 | 0.861704 | Chmp4c | Rn.162567 |
| A_44_P436280 | 0.011074 | 0.705665 | Pltp | Rn.117434 |
| A_44_P436284 | 0.002837 | 0.818709 |  |  |
| A_44_P436352 | 0.029684 | 1.271915 | Pdzd11 | Rn.144513 |
| A_44_P436628 | 0.000171 | 1.833122 |  | Rn.164465 |
| A_44_P436942 | 0.02865 | 1.126489 |  | Rn.166202 |
| A_44_P438150 | 0.00238 | 0.813877 | Dag1 | Rn.36260 |
| A_44_P438520 | 0.001033 | 0.692165 |  |  |
| A_44_P438529 | 2.90E-05 | 2.249774 |  |  |
| A_44_P438904 | 0.037265 | 0.822156 | Cldn8 | Rn.79297 |
| A_44_P439573 | 0.019692 | 0.874031 |  | Rn.1619 |
| A_44_P439597 | 0.012432 | 1.383904 |  | Rn.14919 |
| A_44_P440466 | 0.022582 | 0.816443 | Ralgapa1 | Rn.86984 |
| A_44_P440953 | 0.017779 | 0.721635 |  |  |
| A_44_P441129 | 0.005268 | 1.231052 | RGD1559724 | |
| A_44_P441296 | 0.033658 | 1.624918 |  |  |
| A_44_P441833 | 0.022286 | 1.361194 |  | Rn.162361 |
| A_44_P441937 | 0.005633 | 1.135268 |  | Rn.3543 |
| A_44_P442161 | 0.045501 | 0.81964 | Ppp2r4 | Rn.24600 |
| A_44_P442589 | 0.001387 | 0.711891 | Efna1 | Rn.8427 |
| A_44_P442873 | 0.015194 | 0.847756 | Becn1 | Rn.2776 |
| A_44_P443349 | 0.014741 | 2.019165 | RGD1306195 | Rn.107841 |
| A_44_P443572 | 0.016476 | 0.889474 | RGD1561360 | |
| A_44_P444116 | 0.000315 | 1.521123 | Aox1 |  |
| A_44_P444343 | 0.024131 | 0.894593 |  | Rn.763 |
| A_44_P444610 | 0.046786 | 1.529404 |  | Rn.101159 |
| A_44_P444897 | 0.00294 | 2.217855 |  | Rn.101115 |
| A_44_P445031 | 0.047603 | 0.809861 | Fbxo32 | Rn.72619 |
| A_44_P445166 | 0.002489 | 0.720053 | Inppl1 | Rn.42902 |
| A_44_P445484 | 0.023362 | 0.906249 |  |  |
| A_44_P445572 | 0.023061 | 0.852363 | Sort1 | Rn.11286 |
| A_44_P445687 | 0.018466 | 0.678298 | Anks1a | Rn.116614 |
| A_44_P445831 | 0.038789 | 1.142743 | Slc39a9 | Rn.101103 |
| A_44_P446375 | 0.023748 | 1.204772 | Atad2 | Rn.6690 |
| A_44_P446539 | 0.014042 | 1.263278 | Naa38 | Rn.14059 |
| A_44_P446886 | 0.04907 | 0.903453 |  | Rn.82537 |
| A_44_P447222 | 0.003645 | 1.379187 |  | Rn.166508 |
| A_44_P447368 | 0.001048 | 0.804013 | Gnb2l1 | Rn.55487 |
| A_44_P447440 | 0.046417 | 0.852782 | Stau2 | Rn.73714 |
| A_44_P447839 | 0.01858 | 0.73183 | Zfp503 | Rn.37928 |
| A_44_P447907 | 0.022074 | 0.673025 | Cgn | Rn.223664 |
| A_44_P448193 | 0.010277 | 0.811429 | RGD1304719 | Rn.3813 |
| A_44_P448223 | 0.001978 | 1.201614 | RGD1563958 | |
| A_44_P448307 | 0.02729 | 0.906653 | Arpc2 | Rn.93317 |
| A_44_P448710 | 0.013251 | 0.657591 | Slc44a1 | Rn.97686 |
| A_44_P449160 | 0.006602 | 0.727025 |  | Rn.22216 |
| A_44_P449263 | 0.004883 | 1.581835 | Fubp3 | Rn.199412 |
| A_44_P449518 | 0.001589 | 1.302761 |  | Rn.8398 |
| A_44_P449555 | 0.012501 | 0.884502 | Yif1 | Rn.7355 |
| A_44_P449567 | 0.013617 | 1.36109 | Rtn4rl1 | Rn.22361 |
| A_44_P449638 | 0.038435 | 1.246344 | Psma2 | Rn.1617 |
| A_44_P449799 | 0.027563 | 0.800408 | Pabpn1 | Rn.39199 |
| A_44_P449969 | 0.027448 | 0.87756 | Chfr | Rn.15515 |
| A_44_P449994 | 0.021188 | 1.36488 | LOC365238 | Rn.218568 |
| A_44_P450107 | 0.002266 | 2.279927 |  |  |
| A_44_P450149 | 0.01138 | 1.097038 | RGD1561928 | Rn.225083 |
| A_44_P450278 | 0.001066 | 3.231808 |  |  |
| A_44_P450307 | 0.036061 | 0.788074 | Eps8 | Rn.30310 |
| A_44_P450421 | 0.034824 | 0.586618 |  |  |
| A_44_P450471 | 0.00856 | 0.786759 | Ppp1r16a | Rn.145223 |
| A_44_P450496 | 0.038598 | 0.755394 | Krt78 |  |
| A_44_P450562 | 0.019733 | 0.827417 | Armc7 | Rn.198899 |
| A_44_P450967 | 0.041262 | 1.249667 |  | Rn.14610 |
| A_44_P451120 | 0.033425 | 0.902002 | Lmbrd1 | Rn.201860 |
| A_44_P451392 | 0.004378 | 1.235397 | Fbxo33 | Rn.228426 |
| A_44_P451496 | 0.013359 | 1.480163 | Lrrc8b | Rn.170566 |
| A_44_P452163 | 0.014082 | 1.365404 | Kcnj3 | Rn.9809 |
| A_44_P452486 | 0.001477 | 0.820916 |  | Rn.225928 |
| A_44_P452512 | 0.002067 | 1.396908 |  |  |
| A_44_P452705 | 0.047749 | 0.79932 | Rgp1 | Rn.205490 |
| A_44_P452987 | 0.001539 | 1.188518 |  | Rn.12128 |
| A_44_P453519 | 0.048625 | 0.655509 |  | Rn.119131 |
| A_44_P453564 | 0.048083 | 1.14002 | RGD1309995 | Rn.22501 |
| A_44_P453573 | 0.010762 | 1.153888 |  | Rn.1457 |
| A_44_P453870 | 0.009685 | 0.847929 |  | Rn.129121 |
| A_44_P453874 | 0.006954 | 0.884187 | Rpl7 | Rn.129121 |
| A_44_P454076 | 0.045004 | 0.819535 |  | Rn.155356 |
| A_44_P454192 | 0.002496 | 0.828788 | Sp1 | Rn.44609 |
| A_44_P454450 | 0.03041 | 1.279755 |  |  |
| A_44_P454587 | 0.047307 | 0.846064 | Cops6 | Rn.35643 |
| A_44_P454861 | 0.001438 | 2.651456 |  |  |
| A_44_P454872 | 0.012757 | 0.880633 | Cnot1 | Rn.56498 |
| A_44_P454882 | 0.021174 | 1.3139 |  |  |
| A_44_P455108 | 0.000331 | 3.191653 | Eif1 | Rn.137199 |
| A_44_P455174 | 0.033322 | 0.829179 | Jmjd8 | Rn.155261 |
| A_44_P455330 | 0.044668 | 0.711403 |  | Rn.3887 |
| A_44_P455715 | 1.35E-05 | 1.291122 |  | Rn.207938 |
| A_44_P456072 | 0.000809 | 1.465811 |  | Rn.225928 |
| A_44_P456339 | 0.009282 | 1.126175 |  | Rn.96174 |
| A_44_P456479 | 0.014793 | 0.756274 | Col23a1 | Rn.206241 |
| A_44_P456564 | 0.036133 | 1.153118 | Nob1 | Rn.102586 |
| A_44_P456642 | 0.010431 | 0.860695 | Dctn2 | Rn.101923 |
| A_44_P456836 | 0.00847 | 1.249873 | LOC100362849 | Rn.214247 |
| A_44_P457008 | 0.039594 | 1.064109 | C1d | Rn.102095 |
| A_44_P457386 | 0.017227 | 1.55071 |  |  |
| A_44_P457578 | 0.009282 | 1.188205 |  | Rn.2283 |
| A_44_P459092 | 0.001667 | 1.894975 |  |  |
| A_44_P459249 | 0.005868 | 0.831031 | Srp72 | Rn.2930 |
| A_44_P459322 | 0.000665 | 4.430342 |  |  |
| A_44_P459679 | 7.48E-05 | 1.493252 | LOC100365604 | Rn.21749 |
| A_44_P460456 | 0.017393 | 0.896448 | Rae1 | Rn.14600 |
| A_44_P460754 | 0.041329 | 1.153978 |  | Rn.794 |
| A_44_P460928 | 0.000501 | 1.32995 |  | Rn.36437 |
| A_44_P460947 | 0.007531 | 0.824229 |  | Rn.165520 |
| A_44_P461201 | 0.00064 | 2.163259 | Pcsk7 | Rn.10653 |
| A_44_P461657 | 0.00254 | 1.297137 |  |  |
| A_44_P461763 | 0.029811 | 0.873158 | Otud7b | Rn.199225 |
| A_44_P461794 | 0.049582 | 0.924881 | RGD1311501 | Rn.105899 |
| A_44_P461932 | 6.85E-05 | 1.58673 |  |  |
| A_44_P461944 | 0.010852 | 1.197032 | Morf4l2 | Rn.6961 |
| A_44_P462018 | 0.000331 | 1.649035 | LOC100360990 | |
| A_44_P462076 | 0.018537 | 0.803419 | Acy1 | Rn.3679 |
| A_44_P462132 | 0.024091 | 0.828539 | Map2k4 | Rn.198875 |
| A_44_P462823 | 0.016739 | 1.125583 | Nub1 | Rn.72440 |
| A_44_P463034 | 0.008526 | 1.374674 |  | Rn.9386 |
| A_44_P463036 | 0.009193 | 1.203396 |  | Rn.9386 |
| A_44_P463345 | 0.016499 | 0.852853 | Vkorc1 | Rn.97942 |
| A_44_P463557 | 0.012985 | 0.89647 | Rpn1 | Rn.4224 |
| A_44_P463972 | 0.048136 | 1.230913 | Eif4g2 | Rn.121213 |
| A_44_P464221 | 0.000551 | 2.222385 | RGD1566373 | |
| A_44_P465554 | 0.00497 | 0.86286 | Vdac2 | Rn.162037 |
| A_44_P465895 | 0.003984 | 0.856073 | Atp6v1f | Rn.6167 |
| A_44_P466211 | 0.035045 | 1.145806 | Tceb3 | Rn.37427 |
| A_44_P466230 | 0.021188 | 0.83419 | Snrk | Rn.10245 |
| A_44_P466271 | 0.0186 | 0.844862 | Sphk2 | Rn.41053 |
| A_44_P466474 | 0.000224 | 1.784802 | Hspd1-ps3 | Rn.224687 |
| A_44_P466796 | 0.034227 | 1.358281 |  |  |
| A_44_P466989 | 0.006337 | 0.846431 |  | Rn.3831 |
| A_44_P468117 | 0.040248 | 1.145703 | Tm9sf3 | Rn.17150 |
| A_44_P468123 | 0.013093 | 0.817902 | Csnk1g2 | Rn.98151 |
| A_44_P468372 | 0.00994 | 0.842826 | Gnb1 | Rn.126047 |
| A_44_P468412 | 0.010013 | 0.843155 | Nup62 | Rn.54450 |
| A_44_P468650 | 0.00597 | 1.122673 |  |  |
| A_44_P468949 | 0.002031 | 0.896844 | Vps13d | Rn.2644 |
| A_44_P469011 | 0.006419 | 0.838972 | Tspan31 | Rn.129220 |
| A_44_P469188 | 0.001608 | 0.849749 | Appbp2 | Rn.104856 |
| A_44_P470935 | 0.03114 | 1.120757 | Ewsr1 | Rn.52785 |
| A_44_P471099 | 0.010188 | 0.877478 |  | Rn.218050 |
| A_44_P471270 | 0.001822 | 2.304646 |  |  |
| A_44_P471318 | 0.001495 | 0.770984 | Slc44a2 | Rn.140320 |
| A_44_P471691 | 0.020291 | 1.167948 | Vcl | Rn.164613 |
| A_44_P471818 | 0.047251 | 0.765503 |  | Rn.225110 |
| A_44_P471848 | 0.032163 | 0.846209 | LOC100362805 | Rn.128652 |
| A_44_P472500 | 0.026174 | 0.797826 | Glud1 | Rn.55106 |
| A_44_P473437 | 0.043806 | 0.937387 | RGD1564425 | |
| A_44_P473449 | 8.21E-05 | 2.436905 |  |  |
| A_44_P473648 | 0.002707 | 2.521956 |  |  |
| A_44_P474388 | 0.019431 | 1.234102 |  | Rn.202982 |
| A_44_P474591 | 5.03E-05 | 1.253334 |  | Rn.3172 |
| A_44_P474867 | 0.013324 | 1.358561 | Sftpc | Rn.3926 |
| A_44_P475197 | 0.031806 | 0.889946 | Fgfr1 | Rn.9797 |
| A_44_P475218 | 0.044442 | 0.853029 | Pip4k2c | Rn.94783 |
| A_44_P476005 | 0.01819 | 0.70806 | Scmh1 | Rn.7761 |
| A_44_P476107 | 0.014475 | 0.835297 |  |  |
| A_44_P476180 | 6.78E-05 | 2.97429 |  |  |
| A_44_P477116 | 0.045104 | 1.108374 | Pcyt1a | Rn.81192 |
| A_44_P477322 | 0.036633 | 0.84418 | Rtn4 | Rn.1348 |
| A_44_P477371 | 0.028147 | 0.776942 |  | Rn.15594 |
| A_44_P477446 | 0.018609 | 0.942597 | Srp72 | Rn.2930 |
| A_44_P477735 | 0.006229 | 0.879228 | RGD1564943 | Rn.28685 |
| A_44_P477785 | 0.014333 | 0.834736 | Eef2 | Rn.55145 |
| A_44_P478436 | 0.033904 | 0.813499 |  |  |
| A_44_P478515 | 0.022272 | 0.713604 |  | Rn.161053 |
| A_44_P479264 | 0.012306 | 1.168228 |  | Rn.228393 |
| A_44_P479286 | 0.000543 | 0.837927 |  | Rn.166169 |
| A_44_P479600 | 0.005344 | 1.280062 |  | Rn.103172 |
| A_44_P479890 | 0.037355 | 1.680222 | Prrx1 | Rn.203584 |
| A_44_P480183 | 0.019537 | 0.493274 |  | Rn.92527 |
| A_44_P480208 | 0.006703 | 0.924525 | Rps3 | Rn.127805 |
| A_44_P480296 | 0.027934 | 0.89031 | Hadha | Rn.3340 |
| A_44_P480307 | 0.012889 | 0.817855 | Hras | Rn.102180 |
| A_44_P480337 | 0.00034 | 0.746336 | Fiz1 | Rn.18880 |
| A_44_P480757 | 0.023574 | 0.788563 | Cachd1 | Rn.64271 |
| A_44_P480771 | 0.014486 | 1.243789 | Hivep3 | Rn.96066 |
| A_44_P480865 | 0.014746 | 0.684007 | Nudt16 | Rn.16063 |
| A_44_P481013 | 0.00062 | 1.242614 |  | Rn.105873 |
| A_44_P482605 | 0.042336 | 0.860943 | Gipc1 | Rn.30044 |
| A_44_P482749 | 0.007477 | 1.292608 | LOC305052 | Rn.156182 |
| A_44_P482894 | 0.025059 | 0.928665 | RGD1564469 | |
| A_44_P483013 | 0.009162 | 1.264298 |  |  |
| A_44_P483325 | 0.002326 | 0.836969 | Lrrc47 | Rn.160689 |
| A_44_P483334 | 0.003485 | 0.868821 | Lrrc47 | Rn.160689 |
| A_44_P483376 | 0.014841 | 1.125491 |  | Rn.102369 |
| A_44_P484362 | 0.002148 | 1.241258 |  | Rn.224475 |
| A_44_P484485 | 0.001539 | 0.829163 | Fastk | Rn.7680 |
| A_44_P484677 | 0.000278 | 1.340039 | Notch3 | Rn.53876 |
| A_44_P484877 | 0.025907 | 0.843259 |  |  |
| A_44_P484902 | 0.020471 | 0.906316 | Pitpna | Rn.9771 |
| A_44_P484950 | 0.035202 | 0.911291 | Atp5g3 | Rn.2180 |
| A_44_P485190 | 0.01124 | 0.841025 | Nhlrc3 | Rn.11681 |
| A_44_P485227 | 0.042822 | 0.880438 | RGD1309747 | Rn.137376 |
| A_44_P485399 | 0.037654 | 0.801293 | Csgalnact2 | Rn.3853 |
| A_44_P485592 | 0.016686 | 0.812887 | Mrps9 | Rn.13913 |
| A_44_P486760 | 0.046469 | 0.89271 | Ubxn4 | Rn.2022 |
| A_44_P486872 | 0.047866 | 1.206514 |  |  |
| A_44_P487300 | 0.001181 | 1.989083 |  |  |
| A_44_P487482 | 0.000802 | 1.758415 |  |  |
| A_44_P487492 | 0.009016 | 0.848966 |  |  |
| A_44_P487753 | 0.01576 | 1.207302 | Zmym4 | Rn.3185 |
| A_44_P487761 | 0.043743 | 1.12362 | C1qa | Rn.105647 |
| A_44_P487874 | 0.036334 | 0.83899 | LOC100360956 | Rn.225089 |
| A_44_P488475 | 0.021832 | 1.167347 | LOC686590 | |
| A_44_P488535 | 0.036741 | 1.200783 |  | Rn.129803 |
| A_44_P489076 | 0.013342 | 1.138884 |  | Rn.51103 |
| A_44_P489244 | 0.03291 | 0.855958 | Casc3 | Rn.162194 |
| A_44_P489793 | 0.001139 | 1.216879 |  |  |
| A_44_P489852 | 0.005695 | 1.626347 |  |  |
| A_44_P489869 | 0.048045 | 0.669441 | Cd101 | Rn.60888 |
| A_44_P490004 | 0.028634 | 0.893833 | Btbd9 | Rn.48897 |
| A_44_P490107 | 0.042634 | 1.52726 |  |  |
| A_44_P490117 | 0.043469 | 0.749017 | Mbip | Rn.24202 |
| A_44_P490235 | 0.016774 | 2.65396 |  |  |
| A_44_P490253 | 7.17E-05 | 2.009383 |  |  |
| A_44_P490265 | 0.000292 | 1.565664 | RGD1564730 | |
| A_44_P490296 | 0.016424 | 0.765095 | Gga3 | Rn.198912 |
| A_44_P491187 | 0.01716 | 1.096123 |  | Rn.3589 |
| A_44_P491217 | 0.024803 | 0.863014 |  | Rn.209064 |
| A_44_P491286 | 0.029398 | 1.187988 |  | Rn.202975 |
| A_44_P492013 | 0.020342 | 0.739455 | Map3k4 | Rn.66100 |
| A_44_P492409 | 0.01557 | 1.125342 | Kdm4c | Rn.15348 |
| A_44_P492543 | 0.033875 | 0.773956 | Cldn18 | Rn.105888 |
| A_44_P493420 | 0.045555 | 1.486122 |  | Rn.74081 |
| A_44_P493665 | 0.021204 | 0.804196 |  | Rn.2274 |
| A_44_P493747 | 0.01083 | 1.236976 |  | Rn.86985 |
| A_44_P493980 | 0.023026 | 0.846044 | Nptxr | Rn.34907 |
| A_44_P494097 | 0.005524 | 1.223699 | Scaf4 | Rn.101886 |
| A_44_P494396 | 3.58E-05 | 2.187742 | Hspd1-ps2 | Rn.216166 |
| A_44_P494608 | 0.006654 | 0.732891 | Gab1 | Rn.1725 |
| A_44_P494696 | 0.000136 | 2.412442 |  |  |
| A_44_P495542 | 0.035803 | 0.867885 | Plagl2 | Rn.107493 |
| A_44_P495611 | 0.024736 | 1.1667 | Wdfy3 | Rn.57981 |
| A_44_P495648 | 0.003656 | 0.844595 | Sec24b | Rn.102003 |
| A_44_P495675 | 0.034608 | 1.233317 |  | Rn.93187 |
| A_44_P496788 | 0.01961 | 1.270603 | Fip1l1 | Rn.138993 |
| A_44_P496848 | 0.004266 | 0.572348 | Arrdc2 | Rn.22831 |
| A_44_P497065 | 0.010232 | 0.810211 | Stoml2 | Rn.2208 |
| A_44_P497339 | 0.011257 | 0.871277 | Asf1a | Rn.163427 |
| A_44_P499309 | 0.035535 | 0.812302 | Fam38a | Rn.20892 |
| A_44_P499318 | 0.015101 | 1.127879 | LOC294154 | Rn.116613 |
| A_44_P499401 | 0.007084 | 0.836788 | Tmem185a | Rn.6505 |
| A_44_P499461 | 0.049065 | 0.895689 |  |  |
| A_44_P499851 | 0.015413 | 0.818893 | Safb | Rn.88640 |
| A_44_P500013 | 0.017987 | 0.824261 | Anpep | Rn.11132 |
| A_44_P500165 | 0.000329 | 1.286613 |  | Rn.2498 |
| A_44_P500586 | 0.048923 | 0.901117 |  | Rn.167594 |
| A_44_P500694 | 0.005709 | 1.16766 | Cldnd1 | Rn.101823 |
| A_44_P500767 | 0.000568 | 1.307066 |  | Rn.107335 |
| A_44_P500880 | 0.008038 | 0.853284 | App | Rn.2104 |
| A_44_P501292 | 0.017733 | 2.69036 |  |  |
| A_44_P501357 | 0.025923 | 0.927226 | Copa | Rn.203109 |
| A_44_P501410 | 0.003321 | 1.666687 |  |  |
| A_44_P501637 | 0.000587 | 0.868144 | Man1a1 | Rn.3576 |
| A_44_P501639 | 0.004461 | 0.824016 | Man1a1 | Rn.3576 |
| A_44_P501984 | 0.014931 | 1.232362 |  | Rn.7715 |
| A_44_P502696 | 0.001679 | 1.139418 |  | Rn.146093 |
| A_44_P503570 | 0.01308 | 0.635252 | Parg | Rn.13634 |
| A_44_P503662 | 0.034494 | 0.810416 | Ftsj2 | Rn.198939 |
| A_44_P503673 | 0.049911 | 0.803257 | Ulk1 | Rn.24509 |
| A_44_P503699 | 0.00139 | 0.8344 | Sergef | Rn.136576 |
| A_44_P503868 | 0.002155 | 2.20214 |  |  |
| A_44_P503964 | 0.016256 | 0.794144 | Trmt1 | Rn.17073 |
| A_44_P504061 | 0.006761 | 0.827461 |  |  |
| A_44_P504374 | 0.006073 | 0.830669 | Mettl13 | Rn.18927 |
| A_44_P505457 | 0.045354 | 1.159889 |  | Rn.4057 |
| A_44_P505885 | 0.043565 | 0.841849 | Vdac1 | Rn.54594 |
| A_44_P506124 | 0.000565 | 1.509566 |  |  |
| A_44_P506568 | 0.000235 | 3.542679 |  |  |
| A_44_P506644 | 0.041182 | 0.895119 | Cpsf1 | Rn.40455 |
| A_44_P506693 | 0.020748 | 0.835851 | Ankzf1 | Rn.163073 |
| A_44_P506751 | 0.000687 | 0.818835 | Abcf3 | Rn.98647 |
| A_44_P506852 | 0.003755 | 1.447666 | Arhgef1 | Rn.64481 |
| A_44_P507274 | 0.014367 | 1.291341 | Dars | Rn.2388 |
| A_44_P507469 | 3.68E-05 | 2.113746 | Myof | Rn.73651 |
| A_44_P508019 | 0.012075 | 0.916117 | Mapkapk5 | Rn.17463 |
| A_44_P508348 | 0.002222 | 1.267606 | Ahnak | Rn.226248 |
| A_44_P508462 | 0.014613 | 0.761583 | Anp32b | Rn.20465 |
| A_44_P508466 | 0.01926 | 0.783504 | Jdp2 | Rn.10721 |
| A_44_P508733 | 0.027062 | 1.318132 | E2f3 | Rn.111536 |
| A_44_P508992 | 0.001073 | 2.121241 |  |  |
| A_44_P509014 | 0.049249 | 1.304663 |  |  |
| A_44_P510087 | 0.048272 | 0.858615 |  | Rn.7993 |
| A_44_P510188 | 0.003608 | 0.814155 |  | Rn.76252 |
| A_44_P510206 | 0.003114 | 1.255875 |  | Rn.95086 |
| A_44_P511049 | 0.032232 | 0.790441 | Prosc | Rn.1880 |
| A_44_P511204 | 0.015103 | 0.912218 | Ndufb11 | Rn.3377 |
| A_44_P511254 | 0.003117 | 2.583929 |  |  |
| A_44_P512656 | 0.02776 | 1.127041 |  | Rn.106351 |
| A_44_P512799 | 0.005126 | 0.772771 | Grik5 | Rn.74042 |
| A_44_P513038 | 0.001154 | 0.831549 | Smarca4 | Rn.23417 |
| A_44_P513114 | 0.030616 | 0.750385 | Trim3 | Rn.203982 |
| A_44_P513677 | 0.00454 | 1.428655 |  |  |
| A_44_P513853 | 0.016191 | 0.731368 | Nxn | Rn.105982 |
| A_44_P514163 | 0.008709 | 1.606993 |  |  |
| A_44_P514379 | 0.0199 | 0.808322 | Acaca | Rn.163753 |
| A_44_P514539 | 0.00275 | 0.758845 | Eif4b | Rn.95954 |
| A_44_P514802 | 0.025024 | 1.203807 |  | Rn.161950 |
| A_44_P514987 | 0.008508 | 0.778397 | Tshz1 | Rn.205514 |
| A_44_P515029 | 0.025973 | 0.742944 | Map1b | Rn.98152 |
| A_44_P515360 | 0.044657 | 1.27878 | Scamp1 | Rn.20374 |
| A_44_P515538 | 0.000107 | 1.480651 | LOC688812 | Rn.224991 |
| A_44_P515593 | 0.000619 | 2.110398 |  |  |
| A_44_P515679 | 0.017395 | 0.84872 | Rab11fip2 | Rn.64225 |
| A_44_P515855 | 0.002901 | 1.968323 |  |  |
| A_44_P515888 | 0.000995 | 0.71859 | Col6a2 | Rn.128903 |
| A_44_P516073 | 0.031141 | 0.719109 | RGD1310552 | Rn.23973 |
| A_44_P516115 | 0.003169 | 1.492205 | RGD1566264 | |
| A_44_P516166 | 0.039723 | 0.79761 |  |  |
| A_44_P517944 | 0.000601 | 2.192511 |  |  |
| A_44_P518528 | 0.019 | 0.861373 | Trim11 | Rn.9308 |
| A_44_P519112 | 0.000368 | 2.99924 |  | Rn.16802 |
| A_44_P519487 | 0.003791 | 1.144568 |  | Rn.3233 |
| A_44_P519709 | 0.005877 | 1.309158 |  | Rn.162443 |
| A_44_P520136 | 0.026908 | 0.76406 | Kcnq5l | Rn.32232 |
| A_44_P520535 | 0.004423 | 0.88544 |  |  |
| A_44_P520557 | 0.012202 | 0.747455 | Arhgap1 | Rn.64898 |
| A_44_P520700 | 0.040835 | 0.891231 |  | Rn.105397 |
| A_44_P520748 | 0.046491 | 0.90783 | Cad | Rn.19416 |
| A_44_P520992 | 0.03242 | 1.147017 |  | Rn.212709 |
| A_44_P521564 | 0.049439 | 0.855574 |  | Rn.10526 |
| A_44_P521946 | 0.038971 | 1.296545 |  | Rn.202991 |
| A_44_P522209 | 0.036129 | 0.742999 | Man2a1 | Rn.163804 |
| A_44_P522293 | 0.040877 | 0.912092 | Ufc1 | Rn.8050 |
| A_44_P522388 | 0.001192 | 0.827262 | Nr2c2 | Rn.10485 |
| A_44_P522482 | 0.002564 | 1.156725 | LOC297756 | Rn.155718 |
| A_44_P522492 | 0.043321 | 0.906828 | Clcc1 | Rn.26586 |
| A_44_P522682 | 0.044787 | 0.791052 | Arf6 | Rn.6993 |
| A_44_P522879 | 0.024702 | 1.283601 | Rnf219 | Rn.154151 |
| A_44_P523016 | 0.032594 | 0.830498 | Abhd12 | Rn.128955 |
| A_44_P523045 | 0.002321 | 1.519319 |  |  |
| A_44_P523072 | 0.001941 | 0.773601 |  | Rn.34141 |
| A_44_P523112 | 0.031318 | 0.846166 |  | Rn.154631 |
| A_44_P523244 | 0.010824 | 1.080827 | Efr3a | Rn.21009 |
| A_44_P524846 | 0.011642 | 0.866398 | Sars | Rn.73067 |
| A_44_P525110 | 0.023054 | 2.44764 |  |  |
| A_44_P525151 | 0.001653 | 0.765599 | Ints5 | Rn.17962 |
| A_44_P525157 | 0.046912 | 0.788773 | Zfp278 | Rn.203833 |
| A_44_P525324 | 0.029007 | 0.815665 | Arhgap11a | Rn.51673 |
| A_44_P525452 | 0.037329 | 1.575429 |  |  |
| A_44_P525534 | 0.049819 | 0.909042 | RGD1562381 | |
| A_44_P525557 | 0.03539 | 0.825217 | Fam108a1 | Rn.15712 |
| A_44_P525649 | 0.001138 | 1.545685 |  |  |
| A_44_P525726 | 0.007558 | 1.227126 |  | Rn.93317 |
| A_44_P526397 | 0.002019 | 1.377784 | Paip1 | Rn.9450 |
| A_44_P526933 | 0.013778 | 0.819054 | Ctnnb1 | Rn.112601 |
| A_44_P527134 | 0.03536 | 0.860923 | Oxr1 | Rn.4252 |
| A_44_P527253 | 0.008149 | 0.809908 | Hmgcl | Rn.12297 |
| A_44_P527300 | 0.032618 | 0.755941 | Tchp | Rn.19337 |
| A_44_P527521 | 0.042599 | 1.378798 | RGD1306058 | Rn.3059 |
| A_44_P527739 | 0.000794 | 0.79647 | Pars2 | Rn.205937 |
| A_44_P527780 | 0.015288 | 0.82211 |  |  |
| A_44_P527983 | 0.030693 | 0.814651 |  | Rn.203607 |
| A_44_P528072 | 0.049228 | 1.298394 |  | Rn.198593 |
| A_44_P528235 | 0.00083 | 0.800892 |  |  |
| A_44_P529114 | 0.00044 | 1.470342 |  | Rn.1425 |
| A_44_P529627 | 0.049201 | 0.93094 | Uba1 | Rn.11800 |
| A_44_P529672 | 6.79E-06 | 2.025665 |  |  |
| A_44_P529691 | 0.000929 | 1.506565 | Rbbp8 | Rn.128724 |
| A_44_P529986 | 0.004725 | 0.885107 |  | Rn.199175 |
| A_44_P530060 | 0.021525 | 0.838923 | LOC689591 | |
| A_44_P530075 | 0.024353 | 0.780191 |  |  |
| A_44_P530999 | 0.001929 | 1.375471 |  | Rn.888 |
| A_44_P531076 | 0.025177 | 1.1241 |  | Rn.873 |
| A_44_P531178 | 0.031033 | 1.157592 |  | Rn.53248 |
| A_44_P531727 | 0.022416 | 0.844883 | LOC367195 | Rn.202516 |
| A_44_P531734 | 0.037504 | 1.201877 |  | Rn.34429 |
| A_44_P532038 | 0.023867 | 1.264939 | Aldh18a1 | Rn.3148 |
| A_44_P532057 | 0.000986 | 1.119692 | LOC501979 | Rn.1742 |
| A_44_P532062 | 0.023729 | 0.889396 |  | Rn.12035 |
| A_44_P532297 | 0.038757 | 1.141031 | RGD1309085 | Rn.94436 |
| A_44_P532320 | 0.025305 | 0.816771 | Dcaf10 | Rn.199359 |
| A_44_P532686 | 0.001176 | 2.247496 | Efnb1 | Rn.44398 |
| A_44_P533034 | 0.028327 | 0.868152 |  | Rn.104990 |
| A_44_P533943 | 0.030757 | 0.899854 | Rtn3 | Rn.11652 |
| A_44_P534361 | 0.018809 | 0.747236 | RGD1565043 | Rn.18942 |
| A_44_P534415 | 0.028542 | 1.804009 |  |  |
| A_44_P534635 | 0.011174 | 0.721205 |  | Rn.43956 |
| A_44_P534717 | 0.000584 | 0.876862 | Sbf1 | Rn.7632 |
| A_44_P534781 | 0.000185 | 5.043419 | RGD1564550 | |
| A_44_P535473 | 0.001854 | 1.253847 |  | Rn.199092 |
| A_44_P535480 | 5.39E-06 | 1.425435 | LOC100360679 | Rn.199092 |
| A_44_P535758 | 0.03965 | 0.806949 |  |  |
| A_44_P535815 | 0.000548 | 1.429434 |  | Rn.170768 |
| A_44_P536315 | 0.014572 | 0.832523 | Rab12 | Rn.11021 |
| A_44_P536365 | 0.005722 | 1.133927 | Nbr1 | Rn.94975 |
| A_44_P536553 | 0.001654 | 1.316836 |  |  |
| A_44_P536873 | 0.033152 | 1.657444 |  |  |
| A_44_P537303 | 0.000729 | 0.821891 | Rpl4 | Rn.1133 |
| A_44_P537780 | 0.000416 | 1.298404 |  | Rn.3841 |
| A_44_P538526 | 0.014049 | 0.630391 | Numb | Rn.102078 |
| A_44_P538602 | 0.006584 | 1.11352 | Copb2 | Rn.37178 |
| A_44_P538670 | 0.033769 | 0.740372 | Slc33a1 | Rn.209601 |
| A_44_P538789 | 0.023884 | 2.596843 |  |  |
| A_44_P538946 | 0.020992 | 0.869326 | Clk2 | Rn.17201 |
| A_44_P539156 | 0.013669 | 0.90857 | Dus3l | Rn.42187 |
| A_44_P539215 | 0.000593 | 1.426444 |  |  |
| A_44_P539231 | 0.008412 | 2.752001 |  |  |
| A_44_P540082 | 0.009823 | 1.402777 |  | Rn.11540 |
| A_44_P540304 | 0.015494 | 1.215077 | LOC100365504 | Rn.216419 |
| A_44_P540570 | 8.93E-05 | 3.346754 | Cox7c | Rn.105968 |
| A_44_P540944 | 0.049361 | 0.874242 | Plekha5 | Rn.118736 |
| A_44_P541074 | 0.001481 | 1.402071 | RragB | Rn.203382 |
| A_44_P541082 | 0.048339 | 0.792345 |  |  |
| A_44_P541187 | 0.00268 | 0.822297 | Tpcn2 | Rn.138237 |
| A_44_P541566 | 0.028282 | 0.936217 | LOC689226 | Rn.203082 |
| A_44_P541623 | 0.001334 | 1.317434 | Trabd | Rn.3565 |
| A_44_P541692 | 0.030711 | 0.881062 | Hexim2 | Rn.8406 |
| A_44_P542274 | 0.015877 | 1.322999 |  | Rn.67042 |
| A_44_P542703 | 0.029136 | 0.701727 |  | Rn.166761 |
| A_44_P542785 | 0.01271 | 1.192581 | Ube2k | Rn.203330 |
| A_44_P543288 | 0.011651 | 0.820513 | Stx5 | Rn.5782 |
| A_44_P543576 | 0.001186 | 1.266254 |  |  |
| A_44_P543733 | 0.024652 | 1.997214 |  |  |
| A_44_P544513 | 0.000483 | 2.151485 |  |  |
| A_44_P545107 | 0.00377 | 1.157006 |  | Rn.1025 |
| A_44_P545363 | 0.004891 | 0.775702 | Fh1 | Rn.29782 |
| A_44_P545727 | 0.029062 | 0.880001 | Trafd1 | Rn.16272 |
| A_44_P545958 | 0.00031 | 1.269035 |  |  |
| A_44_P546000 | 0.039879 | 1.123872 | RGD1565438 | |
| A_44_P546170 | 0.049696 | 1.140936 | Ube2h | Rn.21816 |
| A_44_P546372 | 0.00344 | 1.210808 | LOC679586 | |
| A_44_P546476 | 0.027265 | 0.791232 | Yeats2 | Rn.7090 |
| A_44_P546823 | 0.036396 | 0.826899 | Nomo1 | Rn.8989 |
| A_44_P547745 | 0.005537 | 1.17299 |  | Rn.190571 |
| A_44_P547751 | 0.004009 | 1.222491 |  | Rn.190571 |
| A_44_P548107 | 0.000658 | 0.832809 | Nucb1 | Rn.1492 |
| A_44_P548524 | 0.001491 | 0.878951 | RGD1564093 | Rn.121465 |
| A_44_P548559 | 0.010719 | 0.673851 | Derl3 | Rn.20722 |
| A_44_P548726 | 0.031227 | 0.74146 | Zfp414 | Rn.32868 |
| A_44_P548985 | 0.04658 | 0.757611 | Tgfbi | Rn.1046 |
| A_44_P549445 | 0.022224 | 0.823614 | Cs | Rn.66581 |
| A_44_P549998 | 0.002264 | 0.882327 | Dhps | Rn.105891 |
| A_44_P550228 | 0.009988 | 0.786569 | Lamb2 | Rn.774 |
| A_44_P550346 | 0.040162 | 1.172175 | Bcl2l2 | Rn.44267 |
| A_44_P550412 | 0.027118 | 0.919528 | Trpm7 | Rn.86991 |
| A_44_P550454 | 0.000719 | 0.770755 | LOC687516 | |
| A_44_P550918 | 0.002043 | 0.805853 |  |  |
| A_44_P550981 | 0.028447 | 0.872082 | Dmap1 | Rn.22845 |
| A_44_P551909 | 0.000925 | 1.220139 | Ep400 | Rn.76053 |
| A_44_P551919 | 0.017299 | 0.904242 | Ncor1 | Rn.24948 |
| A_44_P553001 | 0.004574 | 0.735228 |  | Rn.176488 |
| A_44_P553037 | 0.013445 | 0.889629 | Rictor | Rn.61013 |
| A_44_P553374 | 0.004676 | 1.189531 | Commd4 | Rn.17650 |
| A_44_P553939 | 0.04283 | 0.804456 | Fyttd1 | Rn.13677 |
| A_44_P554339 | 0.000251 | 1.116268 |  | Rn.2833 |
| A_44_P554377 | 0.046654 | 0.865776 | Erlec1 | Rn.102123 |
| A_44_P555019 | 0.03915 | 0.94082 | Cdc42 | Rn.60067 |
| A_44_P555498 | 0.043126 | 1.590837 | Arhgef7 | Rn.146763 |
| A_44_P555638 | 0.017363 | 0.826711 | Prickle3 | Rn.155951 |
| A_44_P555664 | 0.000509 | 2.426411 |  |  |
| A_44_P555760 | 0.008161 | 1.323186 | Frmd6 | Rn.1708 |
| A_44_P556345 | 0.021411 | 0.785275 |  | Rn.24338 |
| A_44_P556427 | 0.002395 | 1.482037 | Pgc | Rn.9735 |
| A_44_P556921 | 0.000822 | 1.165039 |  | Rn.4166 |
| A_44_P557199 | 0.045835 | 0.828933 | Mlycd | Rn.13468 |
| A_44_P557303 | 0.014782 | 1.337436 | Olr575 | Rn.142897 |
| A_44_P557575 | 0.024546 | 0.89896 |  |  |
| A_44_P557626 | 0.04433 | 1.284258 | Klk9 | Rn.218609 |
| A_44_P557872 | 0.013289 | 0.886033 |  |  |
| A_44_P557885 | 0.000121 | 2.171817 |  |  |
| A_44_P557994 | 0.002071 | 0.897198 |  |  |
| A_44_P558018 | 0.00281 | 1.511572 | LOC100364959 | |
| A_44_P558079 | 0.006214 | 0.866466 | RGD1359127 | Rn.39052 |
| A_44_P558142 | 0.011589 | 0.770597 | Dip2b | Rn.214124 |
| A_44_P558445 | 0.021971 | 1.257791 |  | Rn.13785 |
| A_44_P558784 | 0.000184 | 1.171671 |  | Rn.3408 |
| A_44_P558838 | 0.007411 | 1.104566 |  | Rn.137099 |
| A_44_P558846 | 0.040506 | 1.115504 |  | Rn.164412 |
| A_44_P559392 | 0.000393 | 1.27859 | Txn1 | Rn.29777 |
| A_44_P560036 | 0.00093 | 1.767156 |  |  |
| A_44_P560038 | 0.000596 | 1.547219 |  |  |
| A_44_P560195 | 0.002492 | 1.650142 |  |  |
| A_44_P560311 | 0.000975 | 1.388909 |  |  |
| A_44_P560432 | 0.046571 | 0.748892 |  |  |
| A_44_P563281 | 0.011303 | 1.282142 |  |  |
| A_44_P564102 | 0.01109 | 0.867372 |  |  |
| A_44_P565432 | 0.041711 | 0.861544 |  |  |
| A_44_P567217 | 0.029856 | 0.845364 |  | Rn.203511 |
| A_44_P567248 | 8.72E-05 | 1.166764 |  |  |
| A_44_P567252 | 0.001712 | 1.233964 |  | Rn.34942 |
| A_44_P568715 | 0.029794 | 0.897595 |  | Rn.24369 |
| A_44_P571530 | 0.007503 | 1.27842 |  | Rn.3997 |
| A_44_P571978 | 0.000627 | 1.450928 |  | Rn.170050 |
| A_44_P573304 | 0.019749 | 0.760597 |  | Rn.28015 |
| A_44_P574134 | 0.010169 | 0.86954 | Slc35f5 | Rn.17967 |
| A_44_P574368 | 0.000156 | 1.86434 |  | Rn.6387 |
| A_44_P575132 | 0.002971 | 1.340542 |  |  |
| A_44_P575173 | 0.000805 | 1.323798 |  |  |
| A_44_P575533 | 0.011582 | 0.8731 | Rpl31 | Rn.1101 |
| A_44_P575571 | 0.008455 | 1.863829 |  |  |
| A_44_P575687 | 0.000354 | 1.281341 | LOC691254 | Rn.156480 |
| A_44_P575763 | 0.000482 | 2.450879 |  |  |
| A_44_P576610 | 0.001176 | 0.696784 |  |  |
| A_44_P576764 | 0.041338 | 0.888369 | RGD1564719 | Rn.159895 |
| A_44_P577138 | 0.033402 | 0.777653 |  |  |
| A_44_P578687 | 0.007055 | 1.327836 |  | Rn.72479 |
| A_44_P578999 | 0.000719 | 1.903224 |  |  |
| A_44_P581903 | 0.00095 | 1.159115 |  | Rn.15119 |
| A_44_P586192 | 0.046653 | 1.455293 |  |  |
| A_44_P587322 | 0.037387 | 0.870136 |  | Rn.225954 |
| A_44_P587335 | 0.024509 | 1.53999 |  |  |
| A_44_P589021 | 0.001837 | 1.378608 |  | Rn.12052 |
| A_44_P589185 | 0.033269 | 1.665213 |  | Rn.96228 |
| A_44_P590197 | 0.012976 | 0.881364 |  | Rn.4268 |
| A_44_P590671 | 0.000249 | 1.734363 |  |  |
| A_44_P591698 | 0.012464 | 0.821807 |  | Rn.205048 |
| A_44_P591963 | 0.016786 | 1.106658 | Kdm1b | Rn.31695 |
| A_44_P593525 | 0.031419 | 1.141475 |  | Rn.105357 |
| A_44_P593668 | 0.037795 | 0.804979 |  |  |
| A_44_P597013 | 0.032008 | 1.208366 |  |  |
| A_44_P597723 | 0.006163 | 0.578582 |  |  |
| A_44_P602419 | 0.000703 | 1.378422 |  | Rn.174538 |
| A_44_P602663 | 0.000164 | 1.930422 |  | Rn.164226 |
| A_44_P604267 | 0.001207 | 0.807982 | Chd8 | Rn.98337 |
| A_44_P604787 | 0.035465 | 0.830402 |  | Rn.13202 |
| A_44_P605461 | 0.004523 | 0.90008 | LOC257642 | Rn.107363 |
| A_44_P605733 | 0.002017 | 0.79901 | Rpl5 | Rn.92980 |
| A_44_P606035 | 0.004482 | 1.169914 |  |  |
| A_44_P606516 | 0.03222 | 0.775902 |  | Rn.801 |
| A_44_P607014 | 0.037578 | 1.164636 |  |  |
| A_44_P607510 | 0.037271 | 0.828465 |  | Rn.169454 |
| A_44_P607659 | 0.003948 | 1.213959 |  |  |
| A_44_P609428 | 0.006426 | 0.697452 |  | Rn.209618 |
| A_44_P610959 | 0.02993 | 0.76592 |  |  |
| A_44_P613019 | 0.038111 | 0.872661 |  |  |
| A_44_P613318 | 0.002689 | 1.509819 |  | Rn.202748 |
| A_44_P614140 | 0.011248 | 1.137907 |  |  |
| A_44_P614514 | 0.004806 | 1.778388 |  | Rn.155246 |
| A_44_P618844 | 0.013245 | 0.741324 |  | Rn.6261 |
| A_44_P619541 | 0.008595 | 0.900761 | Dnajc8 | Rn.105212 |
| A_44_P620222 | 0.004617 | 1.332374 | Rb1cc1 | Rn.33137 |
| A_44_P620300 | 0.011369 | 1.316919 |  | Rn.204596 |
| A_44_P620480 | 0.032287 | 1.097264 |  | Rn.2064 |
| A_44_P621230 | 0.002957 | 1.194754 |  |  |
| A_44_P621304 | 0.000811 | 2.663633 |  |  |
| A_44_P621338 | 0.002447 | 1.453912 | Zfp458 | Rn.122959 |
| A_44_P621366 | 0.002127 | 2.291997 |  |  |
| A_44_P621378 | 0.004276 | 1.455722 |  |  |
| A_44_P621592 | 0.011319 | 0.917131 |  |  |
| A_44_P621723 | 0.000523 | 2.495689 |  |  |
| A_44_P621738 | 0.029209 | 0.795304 | Cldn18 | Rn.105888 |
| A_44_P622060 | 0.035012 | 1.161943 |  | Rn.4067 |
| A_44_P622172 | 0.011519 | 0.856294 | Epn2 | Rn.44273 |
| A_44_P622560 | 0.015218 | 0.725567 | Galnt6 | Rn.104292 |
| A_44_P623115 | 0.004466 | 1.238177 | Slc43a2 | Rn.204988 |
| A_44_P623737 | 0.002551 | 0.778165 |  | Rn.199074 |
| A_44_P625333 | 0.041254 | 0.747566 | Ctdspl | Rn.37030 |
| A_44_P628867 | 0.006153 | 0.790581 |  | Rn.199674 |
| A_44_P633025 | 0.039482 | 1.217931 |  | Rn.202975 |
| A_44_P636337 | 0.033758 | 1.14388 | RGD1565566 | |
| A_44_P636923 | 0.001815 | 0.759561 | Dcp1b | Rn.98474 |
| A_44_P637020 | 0.031199 | 1.298569 |  |  |
| A_44_P637047 | 0.001953 | 1.832059 |  |  |
| A_44_P637322 | 0.011578 | 0.872275 |  |  |
| A_44_P637792 | 0.029393 | 1.197274 | Cbfb | Rn.7308 |
| A_44_P638087 | 0.002651 | 0.815087 |  | Rn.7993 |
| A_44_P638377 | 0.011347 | 1.265122 |  |  |
| A_44_P638446 | 0.030491 | 0.664736 |  |  |
| A_44_P638554 | 0.020186 | 0.852962 |  |  |
| A_44_P639923 | 0.021008 | 1.968951 |  |  |
| A_44_P640364 | 0.020067 | 0.791265 |  |  |
| A_44_P641058 | 0.043256 | 1.341419 | Rbpj | Rn.58803 |
| A_44_P642834 | 0.049914 | 1.199934 |  |  |
| A_44_P643268 | 0.032731 | 0.909136 | Gxylt1 | Rn.14826 |
| A_44_P643531 | 0.02587 | 0.787975 |  | Rn.166505 |
| A_44_P645099 | 0.027746 | 1.222811 |  |  |
| A_44_P650082 | 0.033526 | 1.256602 |  | Rn.169873 |
| A_44_P651694 | 0.017715 | 1.260144 |  |  |
| A_44_P651845 | 0.000533 | 1.769996 |  |  |
| A_44_P651992 | 0.000792 | 2.415894 |  |  |
| A_44_P652021 | 0.001538 | 1.593288 |  |  |
| A_44_P652161 | 0.041646 | 2.092469 |  |  |
| A_44_P652213 | 0.001488 | 1.733673 |  |  |
| A_44_P652611 | 0.035311 | 1.107062 |  |  |
| A_44_P652941 | 0.00597 | 0.780243 |  |  |
| A_44_P653240 | 0.038253 | 1.129539 | Eif4ebp3 | Rn.11853 |
| A_44_P654096 | 0.001633 | 0.756581 |  |  |
| A_44_P654250 | 0.016887 | 1.520658 |  |  |
| A_44_P658414 | 0.009921 | 1.377464 |  |  |
| A_44_P665667 | 0.025916 | 1.101136 |  | Rn.15010 |
| A_44_P665701 | 0.018898 | 0.839768 |  | Rn.32245 |
| A_44_P665738 | 0.003445 | 0.85715 | RGD1561149 | Rn.3216 |
| A_44_P666611 | 0.048524 | 1.218319 |  | Rn.164131 |
| A_44_P667069 | 0.003247 | 1.584212 | Qsox2 | Rn.199433 |
| A_44_P667458 | 0.000549 | 1.396281 |  |  |
| A_44_P667469 | 7.65E-06 | 1.747814 |  |  |
| A_44_P667509 | 0.000322 | 3.803367 |  |  |
| A_44_P667648 | 1.92E-05 | 2.193614 |  |  |
| A_44_P667917 | 0.013603 | 1.141734 |  |  |
| A_44_P669882 | 0.029408 | 1.162989 |  |  |
| A_44_P671098 | 0.036694 | 0.853146 |  |  |
| A_44_P674035 | 0.000697 | 1.236131 |  | Rn.129855 |
| A_44_P674069 | 0.030329 | 0.845338 |  |  |
| A_44_P676250 | 0.04406 | 0.861168 |  |  |
| A_44_P678814 | 0.001067 | 2.337164 |  | Rn.144357 |
| A_44_P679698 | 0.047576 | 1.10029 |  |  |
| A_44_P681544 | 0.003759 | 0.780211 |  | Rn.77521 |
| A_44_P683133 | 0.042141 | 1.838863 |  |  |
| A_44_P683219 | 0.000113 | 1.375204 |  |  |
| A_44_P683323 | 0.000132 | 1.327314 | LOC500584 | Rn.107738 |
| A_44_P683392 | 0.00038 | 1.412046 |  |  |
| A_44_P683538 | 0.006946 | 0.856172 |  |  |
| A_44_P683608 | 3.10E-05 | 1.677005 |  | Rn.167770 |
| A_44_P683994 | 0.000198 | 2.529882 |  |  |
| A_44_P684672 | 0.029235 | 0.874781 |  |  |
| A_44_P684912 | 0.030475 | 1.116593 |  |  |
| A_44_P685508 | 0.043822 | 0.852331 |  |  |
| A_44_P685693 | 0.01363 | 0.81889 |  |  |
| A_44_P686682 | 0.031505 | 0.808427 |  |  |
| A_44_P689950 | 0.014898 | 3.077274 |  | Rn.199248 |
| A_44_P695453 | 0.002027 | 1.104938 | Cpsf7 | Rn.98627 |
| A_44_P696227 | 1.41E-05 | 3.622724 |  | Rn.1701 |
| A_44_P696363 | 0.036633 | 1.201289 | Npw | Rn.14575 |
| A_44_P696866 | 0.00545 | 0.576406 | Hmg20a | Rn.9628 |
| A_44_P697841 | 0.043484 | 0.867393 | Cox11 | Rn.19469 |
| A_44_P698367 | 0.000687 | 1.293595 |  |  |
| A_44_P699369 | 0.026168 | 0.930238 | RGD1307597 | Rn.93564 |
| A_44_P699410 | 6.15E-05 | 1.322155 |  |  |
| A_44_P699502 | 0.036375 | 1.091116 | LOC687105 | Rn.216416 |
| A_44_P701112 | 0.020685 | 0.840035 |  | Rn.167930 |
| A_44_P701794 | 0.018681 | 0.829891 |  |  |
| A_44_P704817 | 0.020124 | 1.112695 |  |  |
| A_44_P704947 | 0.041898 | 0.843935 |  |  |
| A_44_P705949 | 0.046334 | 1.252638 |  |  |
| A_44_P706878 | 0.003156 | 1.171152 | RGD1564381 | Rn.141446 |
| A_44_P707159 | 0.044145 | 0.858665 | LOC100365370 | Rn.144602 |
| A_44_P708829 | 0.008625 | 0.558978 | Vamp4 | Rn.105555 |
| A_44_P710024 | 0.024146 | 1.357628 |  |  |
| A_44_P710285 | 0.011246 | 1.113657 | Stim1 | Rn.106771 |
| A_44_P711001 | 0.025911 | 0.793658 |  | Rn.170579 |
| A_44_P711020 | 0.015661 | 0.868387 | Znrf2 | Rn.32245 |
| A_44_P711164 | 0.009665 | 1.182658 |  | Rn.95170 |
| A_44_P712117 | 0.014342 | 0.735395 |  | Rn.43744 |
| A_44_P712590 | 0.039487 | 1.243646 |  | Rn.17793 |
| A_44_P713311 | 0.005473 | 1.134667 | Ncor1 | Rn.24948 |
| A_44_P713783 | 0.002596 | 1.363679 |  |  |
| A_44_P713802 | 0.000289 | 1.597362 | LOC100360380 | Rn.225673 |
| A_44_P713910 | 0.00224 | 0.833263 | Ube2v1 | Rn.220168 |
| A_44_P714581 | 0.040486 | 0.856886 | Strn4 | Rn.137083 |
| A_44_P715158 | 0.002294 | 0.774433 |  |  |
| A_44_P715164 | 0.033488 | 0.714359 |  | Rn.205471 |
| A_44_P715425 | 0.033384 | 1.10812 |  |  |
| A_44_P716645 | 0.01755 | 0.857169 |  |  |
| A_44_P716822 | 0.012252 | 0.801284 |  |  |
| A_44_P717706 | 0.046249 | 0.935181 | Thtpa | Rn.163276 |
| A_44_P718340 | 0.003196 | 0.862968 |  |  |
| A_44_P719889 | 0.008515 | 1.11983 |  | Rn.203014 |
| A_44_P723176 | 0.01274 | 0.758317 |  | Rn.212883 |
| A_44_P723917 | 0.004103 | 1.441178 |  |  |
| A_44_P725429 | 0.015447 | 1.210651 |  | Rn.15726 |
| A_44_P726050 | 0.031359 | 1.564571 |  | Rn.47315 |
| A_44_P726097 | 0.000633 | 1.24256 | Lrsam1 | Rn.61607 |
| A_44_P726568 | 0.035883 | 0.865987 | Ptpn3 | Rn.22271 |
| A_44_P727067 | 0.047226 | 0.734055 |  | Rn.203074 |
| A_44_P728149 | 0.025732 | 0.850451 | Gstm4 | Rn.198607 |
| A_44_P728387 | 0.008692 | 1.322248 |  | Rn.22719 |
| A_44_P728586 | 0.005197 | 2.779647 |  |  |
| A_44_P728623 | 0.044695 | 1.295605 |  |  |
| A_44_P729100 | 0.043906 | 1.303007 |  |  |
| A_44_P729183 | 0.000113 | 1.722694 |  |  |
| A_44_P729436 | 0.022669 | 1.611187 |  |  |
| A_44_P729898 | 0.022261 | 1.115549 | Morc2 | Rn.224734 |
| A_44_P730758 | 0.041607 | 0.761202 |  |  |
| A_44_P731793 | 0.041133 | 0.909309 |  |  |
| A_44_P731802 | 0.000862 | 1.440389 |  |  |
| A_44_P732273 | 0.008066 | 0.755696 |  | Rn.204229 |
| A_44_P736196 | 0.006526 | 1.106326 |  | Rn.40562 |
| A_44_P737376 | 0.005502 | 0.679117 |  | Rn.165496 |
| A_44_P740324 | 3.27E-05 | 2.088315 |  |  |
| A_44_P742272 | 0.033902 | 1.256615 | Hist1h4b | Rn.1659 |
| A_44_P742369 | 0.000649 | 2.138579 | Creb1 | Rn.90061 |
| A_44_P744488 | 0.00033 | 3.049415 |  |  |
| A_44_P744765 | 0.000137 | 1.947492 |  |  |
| A_44_P745100 | 0.025486 | 1.31989 | Cisd2 | Rn.24858 |
| A_44_P745166 | 0.042077 | 1.141745 | Zfp828 | Rn.3851 |
| A_44_P745177 | 0.019828 | 0.851099 | Vamp4 | Rn.105555 |
| A_44_P746008 | 0.028173 | 1.318632 |  | Rn.17913 |
| A_44_P746944 | 0.033552 | 0.77351 |  |  |
| A_44_P747084 | 0.015145 | 1.365263 |  |  |
| A_44_P747545 | 0.00092 | 0.73198 | Xpr1 | Rn.138575 |
| A_44_P747637 | 0.036257 | 0.707043 |  |  |
| A_44_P748217 | 7.74E-05 | 1.759292 |  | Rn.42633 |
| A_44_P748361 | 0.006082 | 1.544404 |  |  |
| A_44_P749094 | 0.0072 | 0.605501 |  |  |
| A_44_P751956 | 0.003431 | 0.742914 |  | Rn.165139 |
| A_44_P753384 | 0.000826 | 0.768973 |  |  |
| A_44_P753601 | 0.001052 | 1.519034 |  |  |
| A_44_P754597 | 0.038314 | 0.406757 |  |  |
| A_44_P759538 | 0.021567 | 0.761904 |  | Rn.105397 |
| A_44_P759911 | 0.00011 | 2.31472 |  |  |
| A_44_P759962 | 0.049233 | 1.268077 |  |  |
| A_44_P760131 | 0.001717 | 2.742251 |  |  |
| A_44_P760225 | 0.000165 | 2.812399 |  |  |
| A_44_P760235 | 0.038896 | 1.333659 |  |  |
| A_44_P760299 | 0.000471 | 2.320147 |  |  |
| A_44_P760628 | 0.001136 | 2.439302 |  |  |
| A_44_P760998 | 0.011612 | 0.849228 | Fam185a | Rn.134510 |
| A_44_P761886 | 0.001836 | 1.199301 | Zswim1 | Rn.27453 |
| A_44_P762216 | 0.047328 | 1.190547 |  |  |
| A_44_P762624 | 0.020112 | 0.700912 |  |  |
| A_44_P763009 | 0.036968 | 1.481868 |  |  |
| A_44_P766913 | 0.008898 | 0.716229 |  | Rn.20252 |
| A_44_P767184 | 0.026558 | 0.608064 | Gatsl2 | Rn.170742 |
| A_44_P768945 | 0.013879 | 0.864954 | Dtnbp1 | Rn.187290 |
| A_44_P775747 | 0.010602 | 1.188787 | LOC680441 | |
| A_44_P775797 | 0.000434 | 1.37346 |  |  |
| A_44_P776209 | 0.010067 | 1.470684 | Dpy19l1 | Rn.22725 |
| A_44_P776812 | 0.028287 | 1.164722 |  |  |
| A_44_P776953 | 0.00883 | 2.288353 |  |  |
| A_44_P777181 | 0.025004 | 0.844904 | Rab22a | Rn.37799 |
| A_44_P777874 | 0.029256 | 1.25232 |  |  |
| A_44_P778323 | 0.032823 | 0.81296 |  |  |
| A_44_P778557 | 0.045696 | 0.876191 |  | Rn.206928 |
| A_44_P779971 | 0.01643 | 0.725411 |  | Rn.155123 |
| A_44_P782073 | 0.011393 | 1.242121 |  | Rn.178219 |
| A_44_P782990 | 0.022796 | 0.885348 | Efr3a | Rn.21009 |
| A_44_P784011 | 0.045743 | 0.848003 |  |  |
| A_44_P788597 | 0.01554 | 0.705111 |  | Rn.163645 |
| A_44_P789739 | 0.035484 | 0.817231 | Slc19a2 | Rn.19386 |
| A_44_P790750 | 0.000607 | 1.720571 |  |  |
| A_44_P790767 | 4.54E-05 | 1.941917 |  |  |
| A_44_P790825 | 0.005638 | 1.457821 |  |  |
| A_44_P790861 | 0.012712 | 1.352497 |  |  |
| A_44_P791631 | 0.003596 | 0.816925 | Nhlrc3 | Rn.11681 |
| A_44_P791729 | 0.000334 | 1.827144 | Rtn4rl1 | Rn.22361 |
| A_44_P794770 | 0.003297 | 0.781668 | Trak2 | Rn.26957 |
| A_44_P802511 | 0.026641 | 0.757002 | RGD1566319 | Rn.216753 |
| A_44_P802937 | 0.035005 | 0.864937 |  | Rn.126047 |
| A_44_P805242 | 0.014834 | 1.127718 |  | Rn.16968 |
| A_44_P806145 | 0.000366 | 0.826377 |  |  |
| A_44_P806236 | 0.005029 | 0.806736 |  |  |
| A_44_P806572 | 0.000349 | 2.745605 |  |  |
| A_44_P806646 | 0.029996 | 1.270882 | Zfp609 | Rn.161077 |
| A_44_P807146 | 0.043118 | 1.055007 |  |  |
| A_44_P807238 | 0.037303 | 1.301026 | Mcat | Rn.23115 |
| A_44_P807329 | 0.031241 | 1.200092 |  | Rn.200400 |
| A_44_P808072 | 0.010917 | 1.160973 | Tgs1 | Rn.48378 |
| A_44_P809032 | 0.008643 | 0.722523 | RGD1566001 | Rn.59706 |
| A_44_P809962 | 0.01896 | 0.738768 |  |  |
| A_44_P810190 | 0.00382 | 1.266492 | LOC686980 | |
| A_44_P814374 | 0.038819 | 0.772793 |  |  |
| A_44_P820062 | 0.020956 | 1.799177 | Aif1l | Rn.17100 |
| A_44_P820923 | 0.001713 | 1.456011 |  | Rn.22813 |
| A_44_P821613 | 0.007421 | 1.109083 |  |  |
| A_44_P821839 | 0.022315 | 1.413177 |  |  |
| A_44_P822051 | 0.005356 | 1.88512 |  |  |
| A_44_P822283 | 0.024642 | 0.876767 | Sort1 | Rn.11286 |
| A_44_P824102 | 0.025988 | 0.933502 | LOC100364115 | Rn.200709 |
| A_44_P824666 | 0.04181 | 0.76102 |  |  |
| A_44_P825629 | 0.019858 | 0.792672 |  |  |
| A_44_P829888 | 0.018846 | 0.811762 | Fam107b | Rn.105966 |
| A_44_P831621 | 0.010664 | 1.151148 |  |  |
| A_44_P834467 | 0.010298 | 1.50065 |  | Rn.17603 |
| A_44_P836767 | 0.04492 | 1.545257 |  |  |
| A_44_P836950 | 1.34E-05 | 1.261491 |  |  |
| A_44_P837006 | 0.002307 | 2.318155 |  |  |
| A_44_P837070 | 0.000149 | 2.68846 |  |  |
| A_44_P837196 | 0.008359 | 1.634532 |  |  |
| A_44_P837669 | 0.008687 | 1.470322 | Abce1 | Rn.2961 |
| A_44_P838184 | 0.017353 | 1.600511 | Fn3krp | Rn.130706 |
| A_44_P838798 | 0.016223 | 0.846309 | Med1 | Rn.4262 |
| A_44_P839176 | 0.000189 | 1.338074 | Usp25 | Rn.23509 |
| A_44_P839602 | 0.043225 | 0.918766 |  |  |
| A_44_P839612 | 0.035128 | 0.662371 |  |  |
| A_44_P840837 | 0.007909 | 0.601241 | Lgi3 | Rn.50862 |
| A_44_P842364 | 0.042967 | 0.824949 |  |  |
| A_44_P842451 | 0.013223 | 0.832695 |  |  |
| A_44_P845625 | 0.021946 | 1.279092 |  |  |
| A_44_P848376 | 0.000268 | 1.187629 | Fpgs | Rn.216473 |
| A_44_P848759 | 0.001024 | 1.665688 |  | Rn.203336 |
| A_44_P849269 | 0.002365 | 0.81184 |  | Rn.6525 |
| A_44_P850353 | 0.013943 | 0.872739 |  |  |
| A_44_P852219 | 0.00091 | 0.871236 |  |  |
| A_44_P852338 | 0.007479 | 0.915748 | RGD1563145 | |
| A_44_P852564 | 0.043643 | 1.134761 |  |  |
| A_44_P852603 | 0.000849 | 2.140971 |  |  |
| A_44_P852623 | 0.000249 | 2.042585 |  |  |
| A_44_P852679 | 0.007921 | 0.802098 |  | Rn.215588 |
| A_44_P853184 | 0.036617 | 2.087527 |  |  |
| A_44_P853624 | 0.010043 | 1.086729 |  |  |
| A_44_P854011 | 0.019497 | 0.732383 | Ppfia4 | Rn.105913 |
| A_44_P854437 | 0.002013 | 0.607559 |  |  |
| A_44_P854619 | 0.018296 | 0.796207 |  |  |
| A_44_P854745 | 0.046486 | 0.878032 |  | Rn.15119 |
| A_44_P855350 | 0.030531 | 0.790444 |  |  |
| A_44_P855773 | 0.014125 | 1.218624 |  | Rn.120657 |
| A_44_P855808 | 0.001822 | 1.229051 |  |  |
| A_44_P855945 | 0.003077 | 2.788986 |  |  |
| A_44_P856998 | 0.045016 | 0.87028 |  |  |
| A_44_P858108 | 0.014804 | 0.883223 |  | Rn.207457 |
| A_44_P858381 | 0.002181 | 1.151149 |  |  |
| A_44_P867316 | 0.00606 | 0.777422 | LOC688452 | Rn.15197 |
| A_44_P867386 | 0.001202 | 1.431125 |  |  |
| A_44_P867809 | 0.001138 | 1.361566 |  |  |
| A_44_P867900 | 0.039268 | 1.990514 |  |  |
| A_44_P868087 | 0.003515 | 1.868149 |  |  |
| A_44_P868092 | 0.001682 | 2.071881 | Hsp90ab1 | Rn.98667 |
| A_44_P868573 | 0.015643 | 1.30054 | Chmp7 | Rn.136789 |
| A_44_P868920 | 0.021593 | 1.232018 |  | Rn.168623 |
| A_44_P868944 | 0.04838 | 1.22111 |  |  |
| A_44_P869077 | 0.004725 | 1.161733 |  | Rn.187073 |
| A_44_P869559 | 0.019082 | 0.822475 |  |  |
| A_44_P870513 | 0.000135 | 0.78904 |  |  |
| A_44_P871223 | 0.037211 | 0.919812 |  |  |
| A_44_P876215 | 0.049294 | 0.871192 |  | Rn.165314 |
| A_44_P879017 | 0.020664 | 1.422835 |  |  |
| A_44_P879099 | 0.005014 | 2.535405 |  |  |
| A_44_P879104 | 0.030535 | 1.107684 | Ash1l | Rn.86973 |
| A_44_P879120 | 0.003075 | 2.618387 |  |  |
| A_44_P880354 | 0.023896 | 1.138892 |  | Rn.3502 |
| A_44_P880618 | 0.041476 | 0.900918 | Slc25a36 | Rn.221870 |
| A_44_P881194 | 0.019329 | 0.72328 | Dram | Rn.1826 |
| A_44_P881890 | 0.010275 | 0.88677 | RGD1559442 | Rn.37595 |
| A_44_P882450 | 0.029431 | 1.157024 |  | Rn.4240 |
| A_44_P882856 | 0.000237 | 1.739959 |  |  |
| A_44_P882921 | 0.002672 | 1.397205 |  |  |
| A_44_P883154 | 0.001309 | 1.276853 | Matr3 | Rn.29774 |
| A_44_P883191 | 0.018412 | 1.253249 |  |  |
| A_44_P883204 | 0.001097 | 1.289923 |  |  |
| A_44_P883254 | 1.81E-06 | 1.455243 |  | Rn.203267 |
| A_44_P883305 | 0.002513 | 1.342576 |  |  |
| A_44_P883350 | 5.36E-05 | 2.116582 |  |  |
| A_44_P883575 | 0.022595 | 0.912079 |  |  |
| A_44_P883641 | 0.038611 | 0.830237 | LOC500956 | Rn.18715 |
| A_44_P883659 | 0.001411 | 1.16202 |  |  |
| A_44_P883893 | 0.025444 | 0.76759 | Acss1 | Rn.98236 |
| A_44_P883950 | 0.003041 | 1.142025 |  | Rn.171168 |
| A_44_P884566 | 0.028337 | 1.437579 |  | Rn.16340 |
| A_44_P886495 | 0.007355 | 1.491927 |  |  |
| A_44_P887296 | 0.029317 | 0.715478 |  | Rn.204875 |
| A_44_P888285 | 0.002625 | 0.578619 |  |  |
| A_44_P889400 | 0.005053 | 1.101446 |  | Rn.6769 |
| A_44_P890506 | 0.001215 | 3.228316 |  |  |
| A_44_P895064 | 0.034242 | 1.155896 |  | Rn.140732 |
| A_44_P895970 | 0.004884 | 1.187265 |  | Rn.1476 |
| A_44_P897023 | 0.005425 | 1.615207 | Snx24 | Rn.204247 |
| A_44_P898105 | 0.002911 | 3.812636 |  |  |
| A_44_P898125 | 0.003427 | 1.605127 |  |  |
| A_44_P898176 | 0.008973 | 0.826401 |  | Rn.215588 |
| A_44_P898229 | 0.009529 | 1.272004 |  |  |
| A_44_P898412 | 0.001066 | 2.101822 |  |  |
| A_44_P898566 | 0.028593 | 3.416392 |  |  |
| A_44_P898694 | 0.000754 | 1.713558 |  |  |
| A_44_P898803 | 0.0261 | 0.879788 |  | Rn.102138 |
| A_44_P898880 | 0.036404 | 1.097495 |  |  |
| A_44_P899002 | 0.005041 | 1.870816 |  |  |
| A_44_P899141 | 0.007677 | 0.917634 | Ibtk | Rn.35504 |
| A_44_P899293 | 0.034624 | 1.46814 | Snai1 | Rn.8008 |
| A_44_P900178 | 0.044055 | 0.763401 |  | Rn.170695 |
| A_44_P902199 | 0.002591 | 1.355989 |  |  |
| A_44_P902244 | 0.005396 | 0.831641 | Rnf14 | Rn.115357 |
| A_44_P902375 | 0.000952 | 1.476299 |  |  |
| A_44_P910739 | 0.025983 | 1.151501 |  | Rn.3805 |
| A_44_P912777 | 0.016557 | 1.141331 | RGD1310553 | Rn.12996 |
| A_44_P913245 | 0.048997 | 1.178082 |  |  |
| A_44_P913360 | 0.000225 | 1.812471 |  |  |
| A_44_P913386 | 0.005955 | 0.714487 | Fam117a | Rn.93919 |
| A_44_P913570 | 0.01343 | 1.353321 |  |  |
| A_44_P913600 | 0.002898 | 1.720507 |  |  |
| A_44_P913644 | 0.002528 | 1.277764 |  |  |
| A_44_P913689 | 3.38E-05 | 1.555753 |  |  |
| A_44_P913796 | 0.049664 | 0.7399 | LOC500046 | Rn.32719 |
| A_44_P913962 | 0.001188 | 2.230955 |  |  |
| A_44_P914096 | 0.003156 | 2.118 | RGD1560017 | Rn.110954 |
| A_44_P914261 | 0.000617 | 1.246223 |  |  |
| A_44_P914632 | 0.000179 | 1.695957 |  |  |
| A_44_P915360 | 0.040746 | 0.875931 |  |  |
| A_44_P915922 | 0.015614 | 0.719789 |  |  |
| A_44_P917107 | 0.045467 | 1.211014 |  |  |
| A_44_P919148 | 0.03883 | 0.808532 |  |  |
| A_44_P919954 | 0.019096 | 1.26788 |  |  |
| A_44_P920467 | 0.044499 | 1.094244 |  | Rn.23433 |
| A_44_P923868 | 0.023471 | 1.185338 |  | Rn.123302 |
| A_44_P925168 | 0.001226 | 1.258902 |  |  |
| A_44_P927153 | 0.002592 | 1.542255 | Gnb1 | Rn.126047 |
| A_44_P928592 | 0.002763 | 1.439307 |  |  |
| A_44_P928669 | 0.031358 | 1.162542 |  |  |
| A_44_P928735 | 0.010392 | 0.878526 | Blzf1 | Rn.11987 |
| A_44_P928907 | 0.023845 | 0.876818 | Zfp1 | Rn.225679 |
| A_44_P928928 | 0.040795 | 1.351002 |  | Rn.116943 |
| A_44_P928969 | 0.002383 | 1.316187 |  |  |
| A_44_P929119 | 0.00059 | 2.303567 |  |  |
| A_44_P930723 | 0.000795 | 1.306235 | Eid2b | Rn.52077 |
| A_44_P930814 | 0.03581 | 0.752677 |  |  |
| A_44_P931082 | 0.003293 | 0.680303 | Zc3h6 | Rn.46791 |
| A_44_P931865 | 0.003585 | 0.873222 | Fam160b2 | Rn.124637 |
| A_44_P932059 | 0.016095 | 0.721954 | Gpkow | Rn.147981 |
| A_44_P932635 | 0.025021 | 1.52939 |  |  |
| A_44_P933459 | 0.026538 | 0.810918 | RGD1308874 | Rn.2352 |
| A_44_P934325 | 0.00218 | 1.767069 |  |  |
| A_44_P935307 | 0.006351 | 1.166711 |  |  |
| A_44_P935860 | 0.027546 | 0.855433 | Nrip1 | Rn.62118 |
| A_44_P938073 | 9.63E-05 | 1.496902 |  |  |
| A_44_P940285 | 0.010951 | 0.804773 |  | Rn.10485 |
| A_44_P940501 | 0.024042 | 1.121443 |  |  |
| A_44_P940810 | 0.035448 | 1.265172 |  | Rn.134664 |
| A_44_P940889 | 0.004886 | 1.250687 |  | Rn.3239 |
| A_44_P941342 | 0.048842 | 1.237785 | Reep5 | Rn.40477 |
| A_44_P942198 | 0.007723 | 0.875298 |  | Rn.16322 |
| A_44_P943483 | 0.032387 | 0.874059 | RGD1305938 | Rn.29775 |
| A_44_P943774 | 0.049171 | 1.387717 |  | Rn.164226 |
| A_44_P944294 | 0.040859 | 0.850552 | Rfxap | Rn.198587 |
| A_44_P944832 | 0.048865 | 1.130837 |  | Rn.34429 |
| A_44_P946035 | 0.006926 | 1.342565 | Fam168a | Rn.224676 |
| A_44_P946803 | 0.011052 | 0.828843 |  |  |
| A_44_P946892 | 0.022073 | 1.262715 | LOC100366124 | Rn.47621 |
| A_44_P948069 | 0.002619 | 0.853438 |  | Rn.203026 |
| A_44_P950330 | 0.016581 | 1.119029 |  | Rn.103168 |
| A_44_P955563 | 0.015714 | 1.163167 |  |  |
| A_44_P956049 | 0.044539 | 0.886816 | Pnisr | Rn.8700 |
| A_44_P956951 | 0.031896 | 0.887025 | Atg13 | Rn.98797 |
| A_44_P958051 | 0.021448 | 0.846889 | Ogdh | Rn.45991 |
| A_44_P958650 | 0.015462 | 1.294357 | Isy1 | Rn.25170 |
| A_44_P958935 | 0.004536 | 1.114147 |  |  |
| A_44_P958991 | 0.012041 | 1.41604 |  |  |
| A_44_P959044 | 0.025136 | 0.829236 | Clasrp | Rn.17553 |
| A_44_P959127 | 0.024647 | 1.107701 |  |  |
| A_44_P959263 | 0.005632 | 1.411496 |  |  |
| A_44_P959572 | 0.000554 | 0.857441 | Eef1g | Rn.43300 |
| A_44_P959647 | 0.009319 | 1.441619 |  |  |
| A_44_P959692 | 0.013114 | 1.21451 |  |  |
| A_44_P960350 | 0.026801 | 0.882987 |  | Rn.167587 |
| A_44_P961164 | 0.023297 | 1.753667 | Rhbdl3 | Rn.20693 |
| A_44_P962319 | 0.016699 | 0.700872 |  |  |
| A_44_P964256 | 0.02317 | 0.625089 |  |  |
| A_44_P965229 | 0.048696 | 0.858165 |  |  |
| A_44_P966358 | 0.01305 | 2.183484 |  |  |
| A_44_P968823 | 0.030804 | 1.538019 |  | Rn.187458 |
| A_44_P972303 | 0.039779 | 1.153233 |  | Rn.202341 |
| A_44_P973562 | 2.89E-06 | 1.684759 | Tspan5 | Rn.98240 |
| A_44_P974313 | 0.003749 | 1.169775 | Ncor1 | Rn.24948 |
| A_44_P974665 | 0.000499 | 1.290045 |  |  |
| A_44_P974681 | 0.036369 | 0.635262 |  |  |
| A_44_P974849 | 0.004569 | 1.589592 |  |  |
| A_44_P974975 | 0.012922 | 1.44314 |  | Rn.196482 |
| A_44_P975235 | 0.003659 | 1.146127 | RGD1309220 | Rn.34623 |
| A_44_P975418 | 0.040466 | 1.193615 |  | Rn.10056 |
| A_44_P976212 | 0.019512 | 0.816344 |  |  |
| A_44_P977419 | 0.047016 | 0.899069 | Gopc | Rn.27865 |
| A_44_P978472 | 0.018965 | 0.872173 | Aarsd1 | Rn.153971 |
| A_44_P978775 | 0.000986 | 1.966196 |  |  |
| A_44_P978899 | 0.04028 | 0.602654 | Tjp1 | Rn.101871 |
| A_44_P978901 | 0.040712 | 0.676648 | Tjp1 | Rn.101871 |
| A_44_P979990 | 0.04633 | 0.781931 |  | Rn.148677 |
| A_44_P985640 | 0.016615 | 0.76836 |  | Rn.205327 |
| A_44_P986636 | 0.003288 | 0.857754 |  | Rn.199245 |
| A_44_P988240 | 0.003164 | 1.188855 | Rnf2 | Rn.19719 |
| A_44_P988400 | 5.38E-05 | 2.350736 |  | Rn.155647 |
| A_44_P988460 | 0.016436 | 1.185176 |  | Rn.16015 |
| A_44_P989601 | 0.020668 | 1.511547 |  |  |
| A_44_P990937 | 0.014325 | 0.874494 | Smarca4 | Rn.23417 |
| A_44_P991045 | 0.009054 | 1.236759 | RGD1359460 | Rn.13090 |
| A_44_P991239 | 0.006641 | 1.240314 | Sh3glb1 | Rn.203013 |
| A_44_P991459 | 6.94E-05 | 2.437295 | Nsmce2 | Rn.203454 |
| A_44_P991662 | 0.005153 | 0.872528 | Kif1b | Rn.6526 |
| A_44_P991949 | 0.000523 | 1.973033 | Cox18 | Rn.24568 |
| A_44_P992056 | 0.002781 | 1.911902 | Tmem106a | Rn.16484 |
| A_44_P992306 | 0.024193 | 0.873652 | Gnpda2 | Rn.228412 |
| A_44_P992468 | 0.0038 | 0.882957 | Trappc5 | Rn.162083 |
| A_44_P992523 | 0.002596 | 1.138377 | Cops3 | Rn.3963 |
| A_44_P992854 | 0.019351 | 1.271073 | Rhoa | Rn.107401 |
| A_44_P993027 | 0.008478 | 1.411443 | Pla2g16 | Rn.11377 |
| A_44_P993382 | 0.01081 | 0.831507 | Ssrp1 | Rn.35908 |
| A_44_P993651 | 0.039529 | 0.921448 |  | Rn.137099 |
| A_44_P995887 | 0.003233 | 1.355463 |  |  |
| A_44_P996204 | 0.003093 | 1.335223 |  |  |
| A_44_P996392 | 0.002213 | 1.310838 | Trdmt1 | Rn.115 |
| A_44_P996798 | 0.048838 | 0.862622 |  |  |
| A_44_P997146 | 0.000494 | 0.8363 | Gapvd1 | Rn.22293 |
| A_44_P997737 | 0.026408 | 0.901083 |  | Rn.7458 |
| A_44_P997962 | 0.002382 | 0.801787 | RGD1303117 | Rn.12517 |
| A_44_P998072 | 0.000109 | 0.840559 | Hnrnpa3 | Rn.107690 |
| A_44_P998423 | 0.014955 | 1.14607 | Tmed7 | Rn.67055 |

| Table A3 Analyzed proteomics parameters of lung tissue of chronic obstructive pulmonary disease rats | | | | | |
| --- | --- | --- | --- | --- | --- |
| Identified Proteins | Accession Number | Molecular Weight | A | log2(A/B) | Ration |
| transketolase | IPI00231139 | 71 kDa | Ref | -1.3 | 0.406126 |
| Adenylyl cyclase-associated protein 1 | IPI00555187 | 52 kDa | Ref | -1.2 | 0.435275 |
| Sodium/potassium-transporting ATPase subunit alpha-1 | IPI00326305 | 113 kDa | Ref | -1.2 | 0.435275 |
| Isoform Long of 14-3-3 protein beta/alpha | IPI00230837 | 28 kDa | Ref | -1.1 | 0.466517 |
| Aconitate hydratase, mitochondrial | IPI00421539 | 85 kDa | Ref | -1.1 | 0.466517 |
| Glutathione S-transferase alpha-4 | IPI00210542 | 26 kDa | Ref | -1 | 0.5 |
| Heat shock protein HSP 90-alpha | IPI00210566 | 85 kDa | Ref | -0.9 | 0.535887 |
| Peroxiredoxin-2 | IPI00201561 | 22 kDa | Ref | -0.8 | 0.574349 |
| Heat shock 70 kDa protein 1-like | IPI00213546 | 71 kDa | Ref | -0.8 | 0.574349 |
| Calpain-2 catalytic subunit | IPI00388249 | 80 kDa | Ref | -0.8 | 0.574349 |
| cAMP-dependent protein kinase type II-alpha regulatory subunitcAMP | IPI00196684 | 46 kDa | Ref | -0.8 | 0.574349 |
| Plastin-3 | IPI00210234 | 71 kDa | Ref | -0.8 | 0.574349 |
| Fatty acid synthase | IPI00200661 | 273 kDa | Ref | -0.7 | 0.615572 |
| Uncharacterized protein (Fragment) | IPI00768265 | 346 kDa | Ref | -0.7 | 0.615572 |
| Guanine deaminase | IPI00325884 | 51 kDa | Ref | -0.6 | 0.659754 |
| Annexin A3 | IPI00207390 | 36 kDa | Ref | -0.6 | 0.659754 |
| 14-3-3 protein epsilon | IPI00325135 | 29 kDa | Ref | -0.6 | 0.659754 |
| Heat shock protein HSP 90-beta | IPI00471584 | 83 kDa | Ref | -0.5 | 0.707107 |
| Uncharacterized protein | IPI00362931 | 34 kDa | Ref | -0.5 | 0.707107 |
| Creatine kinase B-type | IPI00470288 | 43 kDa | Ref | -0.5 | 0.707107 |
| Xanthine dehydrogenase/oxidase | IPI00231694 | 146 kDa | Ref | -0.5 | 0.707107 |
| Chloride intracellular channel protein 5 | IPI00189503 | 28 kDa | Ref | -0.5 | 0.707107 |
| Isoform 1 of Endoplasmin | IPI00365985 | 93 kDa | Ref | -0.5 | 0.707107 |
| ras GTPase-activating-like protein IQGAP1 | IPI00365769 | 189 kDa | Ref | -0.5 | 0.707107 |
| RCG61183, isoform CRA_b | IPI00362106 | 78 kDa | Ref | -0.5 | 0.707107 |
| Complement C4 | IPI00213036 | 192 kDa | Ref | -0.5 | 0.707107 |
| cadherin-5 | IPI00768626 | 87 kDa | Ref | -0.5 | 0.707107 |
| Alpha-actinin-4 | IPI00213463 | 105 kDa | Ref | -0.4 | 0.757858 |
| Isoform Short of Annexin A2 | IPI00325146 | 39 kDa | Ref | -0.4 | 0.757858 |
| Uncharacterized protein | IPI00190577 | 404 kDa | Ref | -0.4 | 0.757858 |
| 60 kDa heat shock protein, mitochondrial | IPI00339148 | 61 kDa | Ref | -0.4 | 0.757858 |
| Biglycan | IPI00191090 | 42 kDa | Ref | -0.4 | 0.757858 |
| Uncharacterized protein | IPI00231136 | 137 kDa | Ref | -0.4 | 0.757858 |
| Uncharacterized protein | IPI00211813 | 233 kDa | Ref | -0.4 | 0.757858 |
| Plectin 6 | IPI00209000 | 534 kDa | Ref | -0.4 | 0.757858 |
| Fructose-bisphosphate aldolase A | IPI00231734 | 39 kDa | Ref | -0.4 | 0.757858 |
| Trifunctional enzyme subunit alpha, mitochondrial | IPI00212622 | 83 kDa | Ref | -0.4 | 0.757858 |
| Uncharacterized protein | IPI00886470 | 28 kDa | Ref | -0.4 | 0.757858 |
| Rab5c protein | IPI00191761 | 23 kDa | Ref | -0.4 | 0.757858 |
| Uncharacterized protein (Fragment) | IPI00210360 | 394 kDa | Ref | -0.3 | 0.812252 |
| L-lactate dehydrogenase A chain | IPI00197711 | 36 kDa | Ref | -0.3 | 0.812252 |
| ATP synthase subunit alpha, mitochondrial | IPI00396910 | 60 kDa | Ref | -0.3 | 0.812252 |
| Keratin, type I cytoskeletal 19 | IPI00207014 | 45 kDa | Ref | -0.3 | 0.812252 |
| laminin, gamma 1 | IPI00363849 | 177 kDa | Ref | -0.3 | 0.812252 |
| Clathrin heavy chain 1 | IPI00193983 | 192 kDa | Ref | -0.3 | 0.812252 |
| Complement C3 (Fragment) | IPI00480639 | 186 kDa | Ref | -0.3 | 0.812252 |
| Thioredoxin | IPI00231368 | 12 kDa | Ref | -0.3 | 0.812252 |
| Protein disulfide-isomerase A3 | IPI00324741 | 57 kDa | Ref | -0.3 | 0.812252 |
| Ezrin | IPI00470254 | 69 kDa | Ref | -0.3 | 0.812252 |
| Isoform 2 of Tropomyosin beta chain | IPI00187731 | 33 kDa | Ref | -0.3 | 0.812252 |
| Isoform 1 of Fibrinogen beta chain | IPI00205389 | 54 kDa | Ref | -0.3 | 0.812252 |
| Electron transfer flavoprotein subunit alpha, mitochondrial | IPI00205332 | 35 kDa | Ref | -0.3 | 0.812252 |
| Myosin-9 | IPI00209113 | 226 kDa | Ref | -0.2 | 0.870551 |
| Hemopexin | IPI00195516 | 51 kDa | Ref | -0.2 | 0.870551 |
| Liver regeneration protein lrryan | IPI00392216 | 68 kDa | Ref | -0.2 | 0.870551 |
| Isoform M2 of Pyruvate kinase isozymes M1/M2 | IPI00339197 | 58 kDa | Ref | -0.2 | 0.870551 |
| Alpha-1-macroglobulin | IPI00326140 | 167 kDa | Ref | -0.2 | 0.870551 |
| Cytosolic non-specific dipeptidase | IPI00421899 | 53 kDa | Ref | -0.2 | 0.870551 |
| Hemoglobin subunit beta-2 | IPI00231192 | 16 kDa | Ref | -0.2 | 0.870551 |
| Intercellular adhesion molecule 1 | IPI00197164 | 60 kDa | Ref | -0.2 | 0.870551 |
| Catalase | IPI00231742 | 60 kDa | Ref | -0.2 | 0.870551 |
| 14-3-3 protein zeta/delta | IPI00324893 | 28 kDa | Ref | -0.2 | 0.870551 |
| Hnrpk protein | IPI00194974 | 51 kDa | Ref | -0.2 | 0.870551 |
| Serine (Or cysteine) peptidase inhibitor, clade B, member 6a, isoform CRA_a | IPI00782070 | 43 kDa | Ref | -0.2 | 0.870551 |
| Transgelin-2 | IPI00555171 | 22 kDa | Ref | -0.2 | 0.870551 |
| Uncharacterized protein | IPI00565677 | 288 kDa | Ref | -0.1 | 0.933033 |
| collagen type VI alpha 5-like | IPI00765963 | 290 kDa | Ref | -0.1 | 0.933033 |
| EH domain-containing protein 2 | IPI00211448 | 61 kDa | Ref | -0.1 | 0.933033 |
| Moesin | IPI00212314 | 68 kDa | Ref | -0.1 | 0.933033 |
| Heat shock cognate 71 kDa protein | IPI00208205 | 71 kDa | Ref | -0.1 | 0.933033 |
| Uncharacterized protein | IPI00209258 | 285 kDa | Ref | -0.1 | 0.933033 |
| Carboxylesterase 3 | IPI00326972 | 62 kDa | Ref | -0.1 | 0.933033 |
| Glucose-6-phosphate isomerase | IPI00364311 | 63 kDa | Ref | -0.1 | 0.933033 |
| Isoform 1 of Tubulin beta-5 chain | IPI00197579 | 50 kDa | Ref | -0.1 | 0.933033 |
| Myosin light polypeptide 6 | IPI00365944 | 17 kDa | Ref | -0.1 | 0.933033 |
| Superoxide dismutase [Cu-Zn] | IPI00231643 | 16 kDa | Ref | -0.1 | 0.933033 |
| Rab GDP dissociation inhibitor beta | IPI00197568 | 51 kDa | Ref | -0.1 | 0.933033 |
| Serum deprivation-response protein | IPI00362416 | 46 kDa | Ref | -0.1 | 0.933033 |
| X-prolyl aminopeptidase (Aminopeptidase P) 2, membrane-bound | IPI00197684 | 76 kDa | Ref | -0.1 | 0.933033 |
| Isoform 1 of Gelsolin | IPI00363974 | 86 kDa | Ref | -0.1 | 0.933033 |
| EH domain-containing protein 1 | IPI00360340 | 61 kDa | Ref | -0.1 | 0.933033 |
| Phosphatidylethanolamine-binding protein 1 | IPI00230937 | 21 kDa | Ref | -0.1 | 0.933033 |
| Retinal dehydrogenase 1 | IPI00332042 | 54 kDa | Ref | -0.1 | 0.933033 |
| Annexin A8 | IPI00358087 | 37 kDa | Ref | -0.1 | 0.933033 |
| Tubulin polymerization-promoting protein family member 3 | IPI00187857 | 19 kDa | Ref | -0.1 | 0.933033 |
| Transgelin | IPI00231196 | 23 kDa | Ref | -0.1 | 0.933033 |
| Myosin-Ic | IPI00393867 | 120 kDa | Ref | -0.1 | 0.933033 |
| Peptidyl-prolyl cis-trans isomerase FKBP1A | IPI00231434 | 12 kDa | Ref | -0.1 | 0.933033 |
| Isoform 2 of AP-2 complex subunit beta | IPI00231502 | 106 kDa | Ref | -0.1 | 0.933033 |
| Isoform 1 of Periaxin | IPI00328033 | 146 kDa | Ref | -0.1 | 0.933033 |
| Uncharacterized protein | IPI00780087 | 161 kDa | Ref | -0.1 | 0.933033 |
| Nucleic acid binding factor pRM10 | IPI00208193 | 34 kDa | Ref | -0.1 | 0.933033 |
| collagen, type VI, alpha 1 | IPI00371853 | 110 kDa | Ref | 0.1 | 1.071774 |
| Dihydropyrimidinase-related protein 2 | IPI00870112 | 62 kDa | Ref | 0.1 | 1.071774 |
| Peptidyl-prolyl cis-trans isomerase A | IPI00387771 | 18 kDa | Ref | 0.1 | 1.071774 |
| Glyceraldehyde-3-phosphate dehydrogenase | IPI00555252 | 36 kDa | Ref | 0.1 | 1.071774 |
| Laminin subunit beta-2 | IPI00212868 | 196 kDa | Ref | 0.1 | 1.071774 |
| Keratin, type II cytoskeletal 8 | IPI00389571 | 54 kDa | Ref | 0.1 | 1.071774 |
| Carbonic anhydrase 1 | IPI00360930 | 28 kDa | Ref | 0.1 | 1.071774 |
| GPI-anchored ceruloplasmin | IPI00325847 | 124 kDa | Ref | 0.1 | 1.071774 |
| Lumican | IPI00206403 | 38 kDa | Ref | 0.1 | 1.071774 |
| Ras-related protein Rap-1b | IPI00363395 | 21 kDa | Ref | 0.1 | 1.071774 |
| Tubulin alpha-1A chain | IPI00189795 | 50 kDa | Ref | 0.1 | 1.071774 |
| Isocitrate dehydrogenase [NADP], mitochondrial | IPI00193485 | 51 kDa | Ref | 0.1 | 1.071774 |
| Ras-related protein Rab-1A | IPI00421897 | 23 kDa | Ref | 0.1 | 1.071774 |
| Vesicle amine transport protein 1 homolog (T californica), isoform CRA_a | IPI00201969 | 43 kDa | Ref | 0.1 | 1.071774 |
| Uncharacterized protein | IPI00555297 | 20 kDa | Ref | 0.1 | 1.071774 |
| collagen, type IV, alpha 2 | IPI00365380 | 163 kDa | Ref | 0.1 | 1.071774 |
| 6-phosphogluconate dehydrogenase, decarboxylating | IPI00382191 | 121 kDa | Ref | 0.1 | 1.071774 |
| ADP-ribosylation factor 2 | IPI00213677 | 21 kDa | Ref | 0.1 | 1.071774 |
| Voltage-dependent anion-selective channel protein 1 | IPI00421874 | 31 kDa | Ref | 0.1 | 1.071774 |
| epidermal growth factor-like protein 6-like isoform 1 | IPI00766606 | 67 kDa | Ref | 0.1 | 1.071774 |
| Glutamate dehydrogenase 1, mitochondrial | IPI00324633 | 61 kDa | Ref | 0.1 | 1.071774 |
| Chloride intracellular channel protein 1 | IPI00421995 | 27 kDa | Ref | 0.1 | 1.071774 |
| AHNAK nucleoprotein isoform 1 | IPI00766829 | 539 kDa | Ref | 0.2 | 1.148698 |
| hemoglobin alpha 2 chain | IPI00205036 | 15 kDa | Ref | 0.2 | 1.148698 |
| Isoform 1 of Serotransferrin | IPI00679202 | 76 kDa | Ref | 0.2 | 1.148698 |
| Uncharacterized protein | IPI00190088 | 90 kDa | Ref | 0.2 | 1.148698 |
| Alpha-1-inhibitor 3 | IPI00201262 | 164 kDa | Ref | 0.2 | 1.148698 |
| Aldehyde dehydrogenase, mitochondrial | IPI00197770 | 56 kDa | Ref | 0.2 | 1.148698 |
| Heat shock protein beta-1 | IPI00201586 | 23 kDa | Ref | 0.2 | 1.148698 |
| Uncharacterized protein | IPI00767676 | 228 kDa | Ref | 0.2 | 1.148698 |
| 78 kDa glucose-regulated protein | IPI00206624 | 72 kDa | Ref | 0.2 | 1.148698 |
| Cysteine and glycine-rich protein 1 | IPI00231690 | 21 kDa | Ref | 0.2 | 1.148698 |
| Cofilin-1 | IPI00327144 | 19 kDa | Ref | 0.2 | 1.148698 |
| Uncharacterized protein | IPI00202651 | 87 kDa | Ref | 0.2 | 1.148698 |
| Acetyl-CoA acetyltransferase, mitochondrial | IPI00324302 | 45 kDa | Ref | 0.2 | 1.148698 |
| Isoform 2 of Haptoglobin | IPI00382202 | 42 kDa | Ref | 0.2 | 1.148698 |
| Serum albumin | IPI00191737 | 69 kDa | Ref | 0.3 | 1.231144 |
| Transglutaminase 2, C polypeptide | IPI00205135 | 77 kDa | Ref | 0.3 | 1.231144 |
| Carbonic anhydrase 2 | IPI00230787 | 29 kDa | Ref | 0.3 | 1.231144 |
| Uncharacterized protein (Fragment) | IPI00557598 | 39 kDa | Ref | 0.3 | 1.231144 |
| Alpha-2-HS-glycoprotein | IPI00327469 | 38 kDa | Ref | 0.3 | 1.231144 |
| Basal cell adhesion molecule | IPI00192310 | 68 kDa | Ref | 0.3 | 1.231144 |
| Annexin A4, isoform CRA_a | IPI00231968 | 36 kDa | Ref | 0.3 | 1.231144 |
| Isoform 1 of Murinoglobulin-1 | IPI00212666 | 165 kDa | Ref | 0.3 | 1.231144 |
| Cysteine-rich protein 2 | IPI00200352 | 23 kDa | Ref | 0.3 | 1.231144 |
| Peroxiredoxin-1 | IPI00211779 | 22 kDa | Ref | 0.4 | 1.319508 |
| Purine nucleoside phosphorylase | IPI00870631 | 32 kDa | Ref | 0.4 | 1.319508 |
| Desmin | IPI00421517 | 53 kDa | Ref | 0.4 | 1.319508 |
| Alpha-1-antiproteinase | IPI00324019 | 46 kDa | Ref | 0.4 | 1.319508 |
| destrin-like | IPI00373140 | 19 kDa | Ref | 0.4 | 1.319508 |
| Transitional endoplasmic reticulum ATPase | IPI00212014 | 89 kDa | Ref | 0.4 | 1.319508 |
| myosin-6 | IPI00189809 | 224 kDa | Ref | 0.4 | 1.319508 |
| Biliverdin reductase B (Flavin reductase (NADPH)) (Predicted), isoform CRA_b | IPI00392676 | 22 kDa | Ref | 0.4 | 1.319508 |
| Guanine nucleotide-binding protein G(i) subunit alpha-2 | IPI00231925 | 41 kDa | Ref | 0.4 | 1.319508 |
| Ubiquitin-40S ribosomal protein S27a | IPI00190240 | 18 kDa | Ref | 0.5 | 1.414214 |
| Protein DJ-1 | IPI00212523 | 20 kDa | Ref | 0.5 | 1.414214 |
| ADP/ATP translocase 2ADP/ATP | IPI00200466 | 33 kDa | Ref | 0.5 | 1.414214 |
| Isoform B0b of Heterogeneous nuclear ribonucleoproteins A2/B1 | IPI00212969 | 34 kDa | Ref | 0.5 | 1.414214 |
| Pincher | IPI00200271 | 61 kDa | Ref | 0.5 | 1.414214 |
| Sodium/potassium-transporting ATPase subunit beta-3 | IPI00208061 | 32 kDa | Ref | 0.5 | 1.414214 |
| Serine/threonine kinase 25 (STE20 homolog, yeast), isoform CRA_a | IPI00390595 | 48 kDa | Ref | 0.5 | 1.414214 |
| Cytoplasmic dynein 1 heavy chain 1 | IPI00327630 | 532 kDa | Ref | 0.6 | 1.515717 |
| Galectin-1 | IPI00231275 | 15 kDa | Ref | 0.6 | 1.515717 |
| Uncharacterized protein | IPI00191444 | 31 kDa | Ref | 0.6 | 1.515717 |
| Collagen alpha-2(I) chain | IPI00188921 | 130 kDa | Ref | 0.7 | 1.624505 |
| Isoform 1 of NADH-cytochrome b5 reductase 3 | IPI00231662 | 34 kDa | Ref | 0.7 | 1.624505 |
| Transthyretin | IPI00324380 | 16 kDa | Ref | 0.8 | 1.741101 |
| RT1 class I histocompatibility antigen, AA alpha chainRT1 | IPI00382098 | 42 kDa | Ref | 1 | 2 |

| Table A4 Analyzed proteomics parameters of lung tissue of Bufei Jianpi formula-treated rats | | | | | |
| --- | --- | --- | --- | --- | --- |
| Identified Proteins (237) | Accession Number | Molecular Weight | B | Log2(D/B) | Ration |
| Actin-related protein 2 | IPI00362072 | 45 kDa | Ref | -1.2 | 0.435275282 |
| RT1 class I histocompatibility antigen, AA alpha chain | IPI00382098 | 42 kDa | Ref | -1.1 | 0.466516496 |
| Aconitate hydratase, mitochondrial | IPI00421539 | 85 kDa | Ref | -1 | 0.5 |
| Uncharacterized protein | IPI00555297 | 20 kDa | Ref | -0.9 | 0.535886731 |
| Guanine nucleotide-binding protein G(i) subunit alpha-2 | IPI00231925 | 41 kDa | Ref | -0.9 | 0.535886731 |
| Myristoylated alanine-rich C-kinase substrate | IPI00480687 | 30 kDa | Ref | -0.8 | 0.574349177 |
| Sodium/potassium-transporting ATPase subunit beta-3 | IPI00208061 | 32 kDa | Ref | -0.7 | 0.615572207 |
| Collagen alpha-2(I) chain | IPI00188921 | 130 kDa | Ref | -0.6 | 0.659753955 |
| Transthyretin | IPI00324380 | 16 kDa | Ref | -0.6 | 0.659753955 |
| epidermal growth factor-like protein 6-like isoform 1 | IPI00766606 | 67 kDa | Ref | -0.6 | 0.659753955 |
| Uncharacterized protein | IPI00780087 | 161 kDa | Ref | -0.6 | 0.659753955 |
| RCG61183, isoform CRA_b | IPI00362106 | 78 kDa | Ref | -0.6 | 0.659753955 |
| Cysteine-rich protein 2 | IPI00200352 | 23 kDa | Ref | -0.6 | 0.659753955 |
| Alpha-1-inhibitor 3 | IPI00201262 | 164 kDa | Ref | -0.5 | 0.707106781 |
| Annexin A6 | IPI00421888 | 76 kDa | Ref | -0.5 | 0.707106781 |
| Phosphatidylethanolamine-binding protein 1 | IPI00230937 | 21 kDa | Ref | -0.5 | 0.707106781 |
| myosin-6 | IPI00189809 | 224 kDa | Ref | -0.5 | 0.707106781 |
| Ubiquitin-40S ribosomal protein S27a | IPI00190240 | 18 kDa | Ref | -0.4 | 0.757858283 |
| Alpha-2-HS-glycoprotein | IPI00327469 | 38 kDa | Ref | -0.4 | 0.757858283 |
| Uncharacterized protein | IPI00211813 | 233 kDa | Ref | -0.4 | 0.757858283 |
| Superoxide dismutase [Mn], mitochondrial | IPI00211593 | 25 kDa | Ref | -0.4 | 0.757858283 |
| Calpain-2 catalytic subunit | IPI00388249 | 80 kDa | Ref | -0.4 | 0.757858283 |
| ADP-ribosylation factor 2 | IPI00213677 | 21 kDa | Ref | -0.4 | 0.757858283 |
| Isoform 1 of NADH-cytochrome b5 reductase 3 | IPI00231662 | 34 kDa | Ref | -0.4 | 0.757858283 |
| Laminin subunit beta-2 | IPI00212868 | 196 kDa | Ref | -0.3 | 0.812252396 |
| Keratin, type II cytoskeletal 8 | IPI00389571 | 54 kDa | Ref | -0.3 | 0.812252396 |
| Desmin | IPI00421517 | 53 kDa | Ref | -0.3 | 0.812252396 |
| Guanine deaminase | IPI00325884 | 51 kDa | Ref | -0.3 | 0.812252396 |
| Rab GDP dissociation inhibitor beta | IPI00197568 | 51 kDa | Ref | -0.3 | 0.812252396 |
| Carbonic anhydrase 2 | IPI00230787 | 29 kDa | Ref | -0.3 | 0.812252396 |
| ADP/ATP translocase 2 | IPI00200466 | 33 kDa | Ref | -0.3 | 0.812252396 |
| collagen, type IV, alpha 2 | IPI00365380 | 163 kDa | Ref | -0.3 | 0.812252396 |
| inter-alpha-inhibitor H4 heavy chain | IPI00188541 | 104 kDa | Ref | -0.3 | 0.812252396 |
| cAMP-dependent protein kinase type II-alpha regulatory subunit | IPI00196684 | 46 kDa | Ref | -0.3 | 0.812252396 |
| Uncharacterized protein | IPI00191444 | 31 kDa | Ref | -0.3 | 0.812252396 |
| Nucleic acid binding factor pRM10 | IPI00208193 | 34 kDa | Ref | -0.3 | 0.812252396 |
| Uncharacterized protein | IPI00190088 | 90 kDa | Ref | -0.2 | 0.870550563 |
| Collagen alpha-1(I) chain | IPI00188909 | 138 kDa | Ref | -0.2 | 0.870550563 |
| Vimentin | IPI00230941 | 54 kDa | Ref | -0.2 | 0.870550563 |
| Advanced glycosylation end product-specific receptor | IPI00209188 | 43 kDa | Ref | -0.2 | 0.870550563 |
| Superoxide dismutase [Cu-Zn] | IPI00231643 | 16 kDa | Ref | -0.2 | 0.870550563 |
| Alpha-1-macroglobulin | IPI00326140 | 167 kDa | Ref | -0.2 | 0.870550563 |
| Uncharacterized protein | IPI00190577 | 404 kDa | Ref | -0.2 | 0.870550563 |
| Cysteine and glycine-rich protein 1 | IPI00231690 | 21 kDa | Ref | -0.2 | 0.870550563 |
| Isocitrate dehydrogenase [NADP], mitochondrial | IPI00193485 | 51 kDa | Ref | -0.2 | 0.870550563 |
| Cytoplasmic dynein 1 heavy chain 1 | IPI00327630 | 532 kDa | Ref | -0.2 | 0.870550563 |
| Fatty acid synthase | IPI00200661 | 273 kDa | Ref | -0.2 | 0.870550563 |
| Serpin H1 | IPI00204703 | 47 kDa | Ref | -0.2 | 0.870550563 |
| Uncharacterized protein | IPI00949459 | 90 kDa | Ref | -0.2 | 0.870550563 |
| Tubulin polymerization-promoting protein family member 3 | IPI00187857 | 19 kDa | Ref | -0.2 | 0.870550563 |
| Uncharacterized protein | IPI00202651 | 87 kDa | Ref | -0.2 | 0.870550563 |
| Heat shock 70 kDa protein 1-like | IPI00213546 | 71 kDa | Ref | -0.2 | 0.870550563 |
| Uncharacterized protein | IPI00781155 | 37 kDa | Ref | -0.2 | 0.870550563 |
| Isoform 2 of AP-2 complex subunit beta | IPI00231502 | 106 kDa | Ref | -0.2 | 0.870550563 |
| Serine/threonine kinase 25 (STE20 homolog, yeast), isoform CRA_a | IPI00390595 | 48 kDa | Ref | -0.2 | 0.870550563 |
| AHNAK nucleoprotein isoform 1 | IPI00766829 | 539 kDa | Ref | -0.1 | 0.933032992 |
| Uncharacterized protein | IPI00565677 | 288 kDa | Ref | -0.1 | 0.933032992 |
| collagen, type VI, alpha 1 | IPI00371853 | 110 kDa | Ref | -0.1 | 0.933032992 |
| ATP synthase subunit beta, mitochondrial | IPI00551812 | 56 kDa | Ref | -0.1 | 0.933032992 |
| Peptidyl-prolyl cis-trans isomerase A | IPI00387771 | 18 kDa | Ref | -0.1 | 0.933032992 |
| Heat shock cognate 71 kDa protein | IPI00208205 | 71 kDa | Ref | -0.1 | 0.933032992 |
| Hemopexin | IPI00195516 | 51 kDa | Ref | -0.1 | 0.933032992 |
| Glyceraldehyde-3-phosphate dehydrogenase | IPI00555252 | 36 kDa | Ref | -0.1 | 0.933032992 |
| Transglutaminase 2, C polypeptide | IPI00205135 | 77 kDa | Ref | -0.1 | 0.933032992 |
| Annexin A5 | IPI00471889 | 36 kDa | Ref | -0.1 | 0.933032992 |
| LOC500183 protein | IPI00568389 | 26 kDa | Ref | -0.1 | 0.933032992 |
| Aldehyde dehydrogenase, mitochondrial | IPI00197770 | 56 kDa | Ref | -0.1 | 0.933032992 |
| Peroxiredoxin-1 | IPI00211779 | 22 kDa | Ref | -0.1 | 0.933032992 |
| Purine nucleoside phosphorylase | IPI00870631 | 32 kDa | Ref | -0.1 | 0.933032992 |
| Peroxiredoxin-2 | IPI00201561 | 22 kDa | Ref | -0.1 | 0.933032992 |
| destrin-like | IPI00373140 | 19 kDa | Ref | -0.1 | 0.933032992 |
| Biglycan | IPI00191090 | 42 kDa | Ref | -0.1 | 0.933032992 |
| Vesicle amine transport protein 1 homolog (T californica), isoform CRA_a | IPI00201969 | 43 kDa | Ref | -0.1 | 0.933032992 |
| Keratin, type II cytoskeletal 7 | IPI00421788 | 51 kDa | Ref | -0.1 | 0.933032992 |
| Rho GDP-dissociation inhibitor 1 | IPI00196994 | 23 kDa | Ref | -0.1 | 0.933032992 |
| Pulmonary surfactant-associated protein A | IPI00327502 | 26 kDa | Ref | -0.1 | 0.933032992 |
| Isoform Somatic of Angiotensin-converting enzyme | IPI00324300 | 151 kDa | Ref | -0.1 | 0.933032992 |
| Cofilin-1 | IPI00327144 | 19 kDa | Ref | -0.1 | 0.933032992 |
| Isoform B0b of Heterogeneous nuclear ribonucleoproteins A2/B1 | IPI00212969 | 34 kDa | Ref | -0.1 | 0.933032992 |
| Heat shock protein HSP 90-alpha | IPI00210566 | 85 kDa | Ref | -0.1 | 0.933032992 |
| cytochrome P450 2B2 | IPI00193234 | 57 kDa | Ref | -0.1 | 0.933032992 |
| collagen type VI alpha 5-like | IPI00765963 | 290 kDa | Ref | 0.1 | 1.071773463 |
| Tubulin beta-2C chain | IPI00400573 | 50 kDa | Ref | 0.1 | 1.071773463 |
| EH domain-containing protein 2 | IPI00211448 | 61 kDa | Ref | 0.1 | 1.071773463 |
| Moesin | IPI00212314 | 68 kDa | Ref | 0.1 | 1.071773463 |
| Polymerase I and transcript release factor | IPI00201300 | 44 kDa | Ref | 0.1 | 1.071773463 |
| ATP synthase subunit alpha, mitochondrial | IPI00396910 | 60 kDa | Ref | 0.1 | 1.071773463 |
| Keratin, type I cytoskeletal 19 | IPI00207014 | 45 kDa | Ref | 0.1 | 1.071773463 |
| Complement C3 (Fragment) | IPI00480639 | 186 kDa | Ref | 0.1 | 1.071773463 |
| collagen, type VI, alpha 2 | IPI00372839 | 110 kDa | Ref | 0.1 | 1.071773463 |
| Peroxiredoxin-6 | IPI00231260 | 25 kDa | Ref | 0.1 | 1.071773463 |
| Ras-related protein Rap-1b | IPI00363395 | 21 kDa | Ref | 0.1 | 1.071773463 |
| 78 kDa glucose-regulated protein | IPI00206624 | 72 kDa | Ref | 0.1 | 1.071773463 |
| Tubulin alpha-1A chain | IPI00189795 | 50 kDa | Ref | 0.1 | 1.071773463 |
| X-prolyl aminopeptidase (Aminopeptidase P) 2, membrane-bound | IPI00197684 | 76 kDa | Ref | 0.1 | 1.071773463 |
| Isoform 1 of Gelsolin | IPI00363974 | 86 kDa | Ref | 0.1 | 1.071773463 |
| 60 kDa heat shock protein, mitochondrial | IPI00339148 | 61 kDa | Ref | 0.1 | 1.071773463 |
| Ras-related protein Rab-1A | IPI00421897 | 23 kDa | Ref | 0.1 | 1.071773463 |
| Protein DJ-1 | IPI00212523 | 20 kDa | Ref | 0.1 | 1.071773463 |
| Uncharacterized protein | IPI00197362 | 17 kDa | Ref | 0.1 | 1.071773463 |
| Annexin A8 | IPI00358087 | 37 kDa | Ref | 0.1 | 1.071773463 |
| Methyltransferase like 7A, isoform CRA_b | IPI00654464 | 28 kDa | Ref | 0.1 | 1.071773463 |
| Ezrin | IPI00470254 | 69 kDa | Ref | 0.1 | 1.071773463 |
| Biliverdin reductase B (Flavin reductase (NADPH)) (Predicted), isoform CRA_b | IPI00392676 | 22 kDa | Ref | 0.1 | 1.071773463 |
| Lysozyme C-1 | IPI00211927 | 17 kDa | Ref | 0.1 | 1.071773463 |
| Uncharacterized protein | IPI00886470 | 28 kDa | Ref | 0.1 | 1.071773463 |
| RCG33691 | IPI00213611 | 16 kDa | Ref | 0.1 | 1.071773463 |
| Isoform 1 of Murinoglobulin-1 | IPI00212666 | 165 kDa | Ref | 0.1 | 1.071773463 |
| Isoform 1 of Periaxin | IPI00328033 | 146 kDa | Ref | 0.1 | 1.071773463 |
| 6-phosphofructokinase, liver type | IPI00212741 | 85 kDa | Ref | 0.1 | 1.071773463 |
| Glutamate dehydrogenase 1, mitochondrial | IPI00324633 | 61 kDa | Ref | 0.1 | 1.071773463 |
| cadherin-5 | IPI00768626 | 87 kDa | Ref | 0.1 | 1.071773463 |
| Myosin-9 | IPI00209113 | 226 kDa | Ref | 0.2 | 1.148698355 |
| SEC14-like protein 3 | IPI00208939 | 46 kDa | Ref | 0.2 | 1.148698355 |
| Uncharacterized protein | IPI00209258 | 285 kDa | Ref | 0.2 | 1.148698355 |
| LOC367586 protein | IPI00361346 | 51 kDa | Ref | 0.2 | 1.148698355 |
| Uncharacterized protein | IPI00362931 | 34 kDa | Ref | 0.2 | 1.148698355 |
| Uncharacterized protein | IPI00767676 | 228 kDa | Ref | 0.2 | 1.148698355 |
| Lymphocyte cytosolic protein 1 | IPI00373492 | 70 kDa | Ref | 0.2 | 1.148698355 |
| Chloride intracellular channel protein 5 | IPI00189503 | 28 kDa | Ref | 0.2 | 1.148698355 |
| Phosphoglycerate kinase 1 | IPI00231426 | 45 kDa | Ref | 0.2 | 1.148698355 |
| Transitional endoplasmic reticulum ATPase | IPI00212014 | 89 kDa | Ref | 0.2 | 1.148698355 |
| Retinal dehydrogenase 1 | IPI00332042 | 54 kDa | Ref | 0.2 | 1.148698355 |
| Serine (Or cysteine) peptidase inhibitor, clade B, member 6a, isoform CRA_a | IPI00782070 | 43 kDa | Ref | 0.2 | 1.148698355 |
| Myosin-Ic | IPI00393867 | 120 kDa | Ref | 0.2 | 1.148698355 |
| Annexin A3 | IPI00207390 | 36 kDa | Ref | 0.2 | 1.148698355 |
| ras GTPase-activating-like protein IQGAP1 | IPI00365769 | 189 kDa | Ref | 0.2 | 1.148698355 |
| Trifunctional enzyme subunit alpha, mitochondrial | IPI00212622 | 83 kDa | Ref | 0.2 | 1.148698355 |
| Isoform 1 of Fibrinogen beta chain | IPI00205389 | 54 kDa | Ref | 0.2 | 1.148698355 |
| Electron transfer flavoprotein subunit alpha, mitochondrial | IPI00205332 | 35 kDa | Ref | 0.2 | 1.148698355 |
| Uncharacterized protein (Fragment) | IPI00768265 | 346 kDa | Ref | 0.2 | 1.148698355 |
| Voltage-dependent anion-selective channel protein 1 | IPI00421874 | 31 kDa | Ref | 0.2 | 1.148698355 |
| 14-3-3 protein epsilon | IPI00325135 | 29 kDa | Ref | 0.2 | 1.148698355 |
| Vinculin | IPI00365286 | 117 kDa | Ref | 0.3 | 1.231144413 |
| GF20391-like isoform 1 | IPI00958096 | 25 kDa | Ref | 0.3 | 1.231144413 |
| Malate dehydrogenase, mitochondrial | IPI00197696 | 36 kDa | Ref | 0.3 | 1.231144413 |
| Heat shock protein beta-1 | IPI00201586 | 23 kDa | Ref | 0.3 | 1.231144413 |
| Creatine kinase B-type | IPI00470288 | 43 kDa | Ref | 0.3 | 1.231144413 |
| Alpha-actinin-4 | IPI00213463 | 105 kDa | Ref | 0.3 | 1.231144413 |
| Lumican | IPI00206403 | 38 kDa | Ref | 0.3 | 1.231144413 |
| Basal cell adhesion molecule | IPI00192310 | 68 kDa | Ref | 0.3 | 1.231144413 |
| transketolase | IPI00231139 | 71 kDa | Ref | 0.3 | 1.231144413 |
| Peptidyl-prolyl cis-trans isomerase FKBP1A | IPI00231434 | 12 kDa | Ref | 0.3 | 1.231144413 |
| Pincher | IPI00200271 | 61 kDa | Ref | 0.3 | 1.231144413 |
| Acetyl-CoA acetyltransferase, mitochondrial | IPI00324302 | 45 kDa | Ref | 0.3 | 1.231144413 |
| Rab5c protein | IPI00191761 | 23 kDa | Ref | 0.3 | 1.231144413 |
| hemoglobin alpha 2 chain | IPI00205036 | 15 kDa | Ref | 0.4 | 1.319507911 |
| Alpha-1-antiproteinase | IPI00324019 | 46 kDa | Ref | 0.4 | 1.319507911 |
| Isoform Short of Annexin A2 | IPI00325146 | 39 kDa | Ref | 0.4 | 1.319507911 |
| Uncharacterized protein (Fragment) | IPI00557598 | 39 kDa | Ref | 0.4 | 1.319507911 |
| Calpain small subunit 1 | IPI00213536 | 29 kDa | Ref | 0.4 | 1.319507911 |
| Hemoglobin subunit beta-1 | IPI00230897 | 16 kDa | Ref | 0.5 | 1.414213562 |
| 14-3-3 protein theta | IPI00196661 | 28 kDa | Ref | 0.5 | 1.414213562 |
| Glutathione S-transferase alpha-4 | IPI00210542 | 26 kDa | Ref | 0.5 | 1.414213562 |
| Heat shock protein HSP 90-beta | IPI00471584 | 83 kDa | Ref | 0.6 | 1.515716567 |
| Transgelin | IPI00231196 | 23 kDa | Ref | 0.6 | 1.515716567 |
| Sodium/potassium-transporting ATPase subunit alpha-1 | IPI00326305 | 113 kDa | Ref | 0.6 | 1.515716567 |
| Galectin-1 | IPI00231275 | 15 kDa | Ref | 0.6 | 1.515716567 |
| T-complex protein 1 subunit delta | IPI00337168 | 58 kDa | Ref | 0.6 | 1.515716567 |
| Chloride intracellular channel protein 1 | IPI00421995 | 27 kDa | Ref | 0.6 | 1.515716567 |
| Uncharacterized protein | IPI00201333 | 107 kDa | Ref | 0.6 | 1.515716567 |
| Zero beta-1 globin | IPI00207146 | 16 kDa | Ref | 0.7 | 1.624504793 |
| EH domain-containing protein 1 | IPI00360340 | 61 kDa | Ref | 0.7 | 1.624504793 |
| Plastin-3 | IPI00210234 | 71 kDa | Ref | 0.7 | 1.624504793 |
| Uncharacterized protein | IPI00231136 | 137 kDa | Ref | 0.8 | 1.741101127 |
| Complement C4 | IPI00213036 | 192 kDa | Ref | 0.8 | 1.741101127 |
| Hemoglobin subunit beta-2 | IPI00231192 | 16 kDa | Ref | 0.9 | 1.866065983 |
| Isoform Long of 14-3-3 protein beta/alpha | IPI00230837 | 28 kDa | Ref | 0.9 | 1.866065983 |

| Table A5 the overlapping proteins between COPD and Bufei Jianpi-treated group proteomics measurements | | |
| --- | --- | --- |
| Identified Proteins | Accession Number | Molecular Weight |
| Collagen alpha-2(I) chain | IPI00188921 | 130 kDa |
| Chloride intracellular channel protein 5 | IPI00189503 | 28 kDa |
| myosin-6 | IPI00189809 | 224 kDa |
| Uncharacterized protein | IPI00190088 | 90 kDa |
| Ubiquitin-40S ribosomal protein S27a | IPI00190240 | 18 kDa |
| Uncharacterized protein | IPI00191444 | 31 kDa |
| Rab5c protein | IPI00191761 | 23 kDa |
| Isocitrate dehydrogenase [NADP], mitochondrial | IPI00193485 | 51 kDa |
| X-prolyl aminopeptidase (Aminopeptidase P) 2, membrane-bound | IPI00197684 | 76 kDa |
| Aldehyde dehydrogenase, mitochondrial | IPI00197770 | 56 kDa |
| Cysteine-rich protein 2 | IPI00200352 | 23 kDa |
| ADP/ATP translocase 2 | IPI00200466 | 33 kDa |
| Alpha-1-inhibitor 3 | IPI00201262 | 164 kDa |
| Vesicle amine transport protein 1 homolog (T californica), isoform CRA_a | IPI00201969 | 43 kDa |
| Uncharacterized protein | IPI00202651 | 87 kDa |
| Transglutaminase 2, C polypeptide | IPI00205135 | 77 kDa |
| Electron transfer flavoprotein subunit alpha, mitochondrial | IPI00205332 | 35 kDa |
| Isoform 1 of Fibrinogen beta chain | IPI00205389 | 54 kDa |
| Keratin, type I cytoskeletal 19 | IPI00207014 | 45 kDa |
| Annexin A3 | IPI00207390 | 36 kDa |
| Sodium/potassium-transporting ATPase subunit beta-3 | IPI00208061 | 32 kDa |
| Myosin-9 | IPI00209113 | 226 kDa |
| Uncharacterized protein | IPI00209258 | 285 kDa |
| Plastin-3 | IPI00210234 | 71 kDa |
| Glutathione S-transferase alpha-4 | IPI00210542 | 26 kDa |
| EH domain-containing protein 2 | IPI00211448 | 61 kDa |
| Peroxiredoxin-1 | IPI00211779 | 22 kDa |
| Moesin | IPI00212314 | 68 kDa |
| Trifunctional enzyme subunit alpha, mitochondrial | IPI00212622 | 83 kDa |
| Laminin subunit beta-2 | IPI00212868 | 196 kDa |
| Isoform B0b of Heterogeneous nuclear ribonucleoproteins A2/B1 | IPI00212969 | 34 kDa |
| Complement C4 | IPI00213036 | 192 kDa |
| Alpha-actinin-4 | IPI00213463 | 105 kDa |
| ADP-ribosylation factor 2 | IPI00213677 | 21 kDa |
| Carbonic anhydrase 2 | IPI00230787 | 29 kDa |
| Isoform Long of 14-3-3 protein beta/alpha | IPI00230837 | 28 kDa |
| Uncharacterized protein | IPI00231136 | 137 kDa |
| transketolase | IPI00231139 | 71 kDa |
| Hemoglobin subunit beta-2 | IPI00231192 | 16 kDa |
| Transgelin | IPI00231196 | 23 kDa |
| Peptidyl-prolyl cis-trans isomerase FKBP1A | IPI00231434 | 12 kDa |
| Isoform 1 of NADH-cytochrome b5 reductase 3 | IPI00231662 | 34 kDa |
| Cysteine and glycine-rich protein 1 | IPI00231690 | 21 kDa |
| Guanine nucleotide-binding protein G(i) subunit alpha-2 | IPI00231925 | 41 kDa |
| Transthyretin | IPI00324380 | 16 kDa |
| 14-3-3 protein epsilon | IPI00325135 | 29 kDa |
| Isoform Short of Annexin A2 | IPI00325146 | 39 kDa |
| Sodium/potassium-transporting ATPase subunit alpha-1 | IPI00326305 | 113 kDa |
| Cofilin-1 | IPI00327144 | 19 kDa |
| Alpha-2-HS-glycoprotein | IPI00327469 | 38 kDa |
| Cytoplasmic dynein 1 heavy chain 1 | IPI00327630 | 532 kDa |
| Isoform 1 of Periaxin | IPI00328033 | 146 kDa |
| Retinal dehydrogenase 1 | IPI00332042 | 54 kDa |
| 60 kDa heat shock protein, mitochondrial | IPI00339148 | 61 kDa |
| Annexin A8 | IPI00358087 | 37 kDa |
| EH domain-containing protein 1 | IPI00360340 | 61 kDa |
| Uncharacterized protein | IPI00362931 | 34 kDa |
| collagen, type IV, alpha 2 | IPI00365380 | 163 kDa |
| ras GTPase-activating-like protein IQGAP1 | IPI00365769 | 189 kDa |
| collagen, type VI, alpha 1 | IPI00371853 | 110 kDa |
| destrin-like | IPI00373140 | 19 kDa |
| RT1 class I histocompatibility antigen, AA alpha chain | IPI00382098 | 42 kDa |
| Peptidyl-prolyl cis-trans isomerase A | IPI00387771 | 18 kDa |
| Keratin, type II cytoskeletal 8 | IPI00389571 | 54 kDa |
| Serine/threonine kinase 25 (STE20 homolog, yeast), isoform CRA_a | IPI00390595 | 48 kDa |
| Myosin-Ic | IPI00393867 | 120 kDa |
| ATP synthase subunit alpha, mitochondrial | IPI00396910 | 60 kDa |
| Desmin | IPI00421517 | 53 kDa |
| Ezrin | IPI00470254 | 69 kDa |
| Creatine kinase B-type | IPI00470288 | 43 kDa |
| Heat shock protein HSP 90-beta | IPI00471584 | 83 kDa |
| Complement C3 (Fragment) | IPI00480639 | 186 kDa |
| Glyceraldehyde-3-phosphate dehydrogenase | IPI00555252 | 36 kDa |
| Uncharacterized protein | IPI00555297 | 20 kDa |
| collagen type VI alpha 5-like | IPI00765963 | 290 kDa |
| epidermal growth factor-like protein 6-like isoform 1 | IPI00766606 | 67 kDa |
| AHNAK nucleoprotein isoform 1 | IPI00766829 | 539 kDa |
| Uncharacterized protein (Fragment) | IPI00768265 | 346 kDa |
| cadherin-5 | IPI00768626 | 87 kDa |
| Serine (Or cysteine) peptidase inhibitor, clade B, member 6a, isoform CRA_a | IPI00782070 | 43 kDa |
| Purine nucleoside phosphorylase | IPI00870631 | 32 kDa |
| Uncharacterized protein | IPI00886470 | 28 kDa |

| Table A6 Analyzed metabolomics parameters of lung tissue of chronic obstructive pulmonary disease rats | | | | | | |
| --- | --- | --- | --- | --- | --- | --- |
| Name | rt | m/z | VIP | ttest | metabolite | fold(b2/a2) |
| M310T35 | 0.589 | 310.1124 | 1.41 | 0.01 | N-Acetylneuraminic AcidN-Acetylneuraminic Acid | -0.76 |
| M232T288 | 4.802 | 232.1542 | 1.64 | 0.03 | Butyryl-L-carnitine | -1.16 |
| M321T387 | 6.447 | 321.2415 | 1.52 | 0.04 | 9-HETE | -1.07 |
| M323T529 | 8.817 | 323.2581 | 1.72 | 0.02 | 5-HETrE | -1.05 |
| M522T666 | 11.102 | 522.3559 | 1.96 | 0 | PC(18:1) | -0.25 |
| M301T325 | 5.409 | 301.1749 | 1.71 | 0.02 | Adrenosterone | -0.31 |
| M101T51 | 0.847 | 101.0237 | 1.2 | 0.04 | Succinic anhydride | 0.45 |
| M302T475 | 7.917 | 302.3056 | 1.57 | 0.03 | Sphinganine | -0.42 |
| M305T752 | 12.531 | 305.2454 | 1.72 | 0.02 | Arachidonic Acid (peroxide free) | -0.49 |
| M532T568 | 9.468 | 532.3316 | 1.55 | 0.03 | PC(19:3) | -0.75 |
| M508T642 | 10.706 | 508.3395 | 1.68 | 0.02 | LysoPE(20:1) | 0.23 |
| M339T576 | 9.6 | 339.2541 | 1.6 | 0.03 | LTB3 | -0.76 |
| M403T732 | 12.192 | 403.3574 | 1.53 | 0.04 | 20-hydroxycholesterol | 0.53 |
| M146T35 | 0.589 | 146.1169 | 1.64 | 0.01 | Acetylcholine | 0.43 |
| M520T582 | 9.707 | 520.3403 | 1.94 | 0.01 | PC(18:2) | 0.19 |
| M106T38 | 0.632 | 106.05 | 1.12 | 0.04 | L-Serine | -0.22 |
| M309T766 | 12.759 | 309.2791 | 1.57 | 0.03 | Linoleic Acid ethyl ester | -1.46 |
| M502T556 | 9.26 | 502.291 | 1.92 | 0.01 | Glycerophospho-N-Arachidonoyl Ethanolamine | -0.78 |
| M482T555 | 9.255 | 482.3153 | 2.07 | 0 | PC(15:0) | -0.82 |
| M403T810 | 13.501 | 403.3483 | 1.63 | 0.03 | 25-hydroxy-cholesterol | -0.8 |
| M359T772 | 12.871 | 359.3159 | 1.47 | 0.04 | Hexadecyl Acetyl Glycerol | -0.6 |
| M283T753_1 | 12.554 | 283.2637 | 1.81 | 0.01 | Oleic Acid | -0.4 |
| M271T772 | 12.875 | 271.2632 | 1.61 | 0.03 | Palmitic Acid methyl ester | -0.54 |
| M174T307 | 5.12 | 174.1117 | 1.59 | 0.04 | acetyl-L-leucine | -0.6 |
| M153T73 | 1.218 | 153.0655 | 1.21 | 0.04 | N-Hydroxymethylnicotinamide | 0.36 |
| M316T438 | 7.308 | 316.2848 | 1.75 | 0.01 | 6-hydroxysphingosine | -0.63 |
| M114T36 | 0.604 | 114.0657 | 1.43 | 0.04 | Creatinine | 0.48 |
| M256T779 | 12.981 | 256.2637 | 2.02 | 0 | Palmitic amide | -0.75 |
| M329T810 | 13.502 | 329.3053 | 1.6 | 0.03 | 2-hydroxy-eicosanoic acid | -0.57 |
| M452T478 | 7.965 | 452.2775 | 1.57 | 0.03 | LysoPE(16:1) | -0.28 |
| M300T752 | 12.533 | 300.2899 | 1.64 | 0.02 | Sphingosine | -0.46 |
| M279T742 | 12.37 | 279.2299 | 1.58 | 0.02 | γ-Linolenic Acid | -0.35 |
| M143T310 | 5.164 | 143.1062 | 1.59 | 0.04 | 2-Octenoic acid | -0.67 |
| M328T556 | 9.275 | 328.2848 | 1.19 | 0.01 | N-palmitoyl alanine | -0.42 |
| M617T409 | 6.811 | 617.4208 | 1.82 | 0.01 | PA(30:2) | -0.25 |
| M171T336 | 5.608 | 171.1486 | 1.62 | 0.04 | 3-Acrylamidopropyl trimethylammonium | 0.26 |
| M638T682 | 11.369 | 638.4033 | 1.5 | 0.04 | PS(25:0) | 0.24 |
| M401T848 | 14.14 | 401.3417 | 1.65 | 0.02 | 25-hydroxyvitamin D3 | -1.22 |
| M283T752 | 12.535 | 283.3124 | 1.49 | 0.03 | Stearic acid | -1.12 |
| M611T630 | 10.496 | 611.3754 | 1.61 | 0.03 | PG(24:0) | -1.31 |
| M316T386 | 6.44 | 316.2481 | 1.09 | 0.03 | Decanoyl-L-carnitine | -1.14 |

| Table A7 Analyzed metabolomics parameters of lung tissue of Bufei Jianpi formula-treated rats | | | | |
| --- | --- | --- | --- | --- |
| m/z | VIP | ttest | metabolite | fold(D2/B2) |
| 112.09 | 1.04 | 0.03 | Histamine | -0.37 |
| 133.10 | 1.38 | 0.01 | L-Ornithine | -0.30 |
| 175.09 | 1.02 | 0.00 | Suberic acid | -0.32 |
| 131.03 | 1.38 | 0.00 | Itaconic acid | -0.31 |
| 324.06 | 1.19 | 0.02 | 5'-CMP | -0.65 |
| 114.07 | 1.27 | 0.02 | Creatinine | -0.56 |
| 130.05 | 1.39 | 0.01 | Pyroglutamic acid | -0.21 |
| 348.07 | 1.40 | 0.02 | dGMP | -0.19 |
| 232.15 | 1.74 | 0.00 | Butyryl-L-carnitine | 1.18 |
| 90.06 | 1.56 | 0.01 | L-Alanine | 1.02 |
| 289.18 | 1.48 | 0.02 | 4-hydroxyestradiol | 0.56 |
| 174.11 | 1.58 | 0.01 | N-Acetylleucine | 0.55 |
| 147.06 | 1.47 | 0.01 | Adipic acid | -0.23 |
| 89.06 | 1.50 | 0.01 | Butyric acid | -0.26 |
| 189.11 | 1.29 | 0.04 | Nonanedioic acid | 0.36 |
| 353.23 | 1.11 | 0.04 | PGE2 | 0.58 |
| 321.24 | 1.69 | 0.00 | 15-HETE | 1.61 |
| 159.14 | 1.15 | 0.03 | Nonanoic acid | -0.12 |
| 281.25 | 1.51 | 0.01 | Linoleic acid | 0.84 |
| 300.29 | 1.38 | 0.02 | Sphingosine | 1.28 |
| 285.27 | 1.36 | 0.02 | Stearic acid | 1.35 |
| 283.26 | 1.35 | 0.02 | Oleic Acid | 0.57 |
| 702.49 | 1.31 | 0.04 | PC(30:2) | 0.59 |
| 728.51 | 1.35 | 0.02 | PC(32:3) | 0.70 |
| 127.02 | 1.34 | 0.01 | Dimethyl phosphate | -0.35 |
| 146.12 | 1.58 | 0.00 | Acetylcholine | -0.55 |
| 205.08 | 1.34 | 0.02 | γ-D-Glutamylglycine | -0.43 |
| 173.09 | 1.76 | 0.00 | Glycylproline | 1.29 |
| 153.07 | 1.03 | 0.03 | N-hydroxymethylnicotinamide | -0.36 |
| 113.02 | 1.40 | 0.01 | 3-Furoic acid | -0.45 |
| 214.14 | 1.54 | 0.01 | N-heptanoyl-homoserine lactone | 0.62 |
| 412.21 | 1.40 | 0.02 | PE(12:0) | 0.77 |
| 149.04 | 1.50 | 0.02 | D-α-Hydroxyglutaric acid | -0.28 |
| 273.18 | 1.55 | 0.01 | α-estradiol | 0.82 |
| 174.12 | 1.59 | 0.01 | L-NIO | 0.98 |
| 365.27 | 1.51 | 0.01 | Prostaglandin A1 ethyl ester | -0.98 |
| 381.26 | 1.63 | 0.00 | 20-ethyl-PGE2 | 1.08 |
| 156.15 | 1.28 | 0.04 | Ethyldimethylaminopropyl Carbodiimide | 0.53 |
| 478.29 | 1.69 | 0.01 | LysoPE(18:2) | 0.78 |
| 316.28 | 1.27 | 0.04 | 6-hydroxysphingosine | 0.71 |
| 337.24 | 1.19 | 0.03 | LTB4 | 0.74 |
| 647.45 | 1.51 | 0.01 | PA(32:1) | -0.69 |
| 403.25 | 1.49 | 0.01 | dehydrocholic acid | 1.37 |
| 468.31 | 1.54 | 0.01 | PC(14:0) | 0.24 |
| 355.28 | 1.37 | 0.02 | 2-Linoleoyl Glycerol | 0.74 |
| 323.26 | 1.61 | 0.00 | 5-HETrE | 0.91 |
| 504.31 | 1.45 | 0.02 | LysoPE(20:3) | 0.70 |
| 480.51 | 1.11 | 0.01 | Glycerophospho-N-Arachidonoyl Ethanolamine | 0.94 |
| 297.24 | 1.41 | 0.02 | 9-HODE | 0.51 |
| 532.33 | 1.34 | 0.02 | PC(19:3) | -0.89 |
| 482.32 | 1.66 | 0.00 | PC(15:0) | 0.55 |
| 298.27 | 1.43 | 0.02 | 3-ketosphingosine | 0.77 |
| 339.25 | 1.46 | 0.01 | LTB3 | 0.72 |
| 506.32 | 1.33 | 0.03 | LysoPE(20:2) | -0.81 |
| 307.26 | 1.34 | 0.03 | Linolenic Acid ethyl ester | 0.48 |
| 518.32 | 1.30 | 0.02 | PC(18:3) | 0.30 |
| 508.34 | 1.49 | 0.01 | LysoPE(20:1) | -0.79 |
| 333.24 | 1.29 | 0.03 | Leukotriene A4 methyl ester | -0.69 |
| 544.34 | 1.26 | 0.04 | PC(20:4) | 0.69 |
| 522.36 | 1.93 | 0.00 | PC(18:1) | 0.35 |
| 277.21 | 1.41 | 0.01 | 9,12-Octadecadiynoic Acid | 0.98 |
| 340.29 | 1.46 | 0.01 | N-hexadecanoyl-L-Homoserine lactone | 1.16 |
| 403.36 | 1.56 | 0.01 | 4β-hydroxy-cholesterol | -0.77 |
| 305.25 | 1.32 | 0.03 | Arachidonic Acid (peroxide free) | 0.55 |
| 359.32 | 1.32 | 0.03 | Hexadecyl Acetyl Glycerol | 0.80 |
| 271.26 | 1.45 | 0.01 | Palmitic Acid methyl ester | -0.91 |
| 319.26 | 1.30 | 0.04 | Arachidonic Acid methyl ester | 0.88 |
| 401.34 | 1.53 | 0.01 | 25-Hydroxycholecalciferol (25-hydroxyvitamin D3) | 0.94 |

| Table A8 175 candidate targets derived from system pharmacology | |
| --- | --- |
| **Target name** | **Gene name** |
| Nitric oxide synthase, inducible | MAPK8 |
| Prostaglandin G/H synthase 1 | POR |
| Dopamine D1 receptor | CYCS |
| Insulin receptor | GABRA6 |
| Muscarinic acetylcholine receptor M3 | MMP1 |
| Thrombin | ACPP |
| Potassium voltage-gated channel subfamily H member 2 | OPRM1 |
| Muscarinic acetylcholine receptor M1 | IL2 |
| Estrogen receptor | PLA2G4A |
| Androgen receptor | APP |
| D(1B) dopamine receptor | CTRB1 |
| Beta-1 adrenergic receptor | ATP5B |
| Sodium channel protein type 5 subunit alpha |  |
| Peroxisome proliferator activated receptor gamma | CHRM3 |
| Coagulation factor Xa | katA |
| Apoptosis regulator Bcl-2 | AR |
| Muscarinic acetylcholine receptor M5 | LDLR |
| Arachidonate 5-lipoxygenase | BCL2 |
| 4-aminobutyrate aminotransferase, mitochondrial | mvaA |
| Prostaglandin G/H synthase 2 | CHRNA7 |
| Nitric-oxide synthase, endothelial |  |
| Alpha-2A adrenergic receptor | ADRA1D |
| 5-hydroxytryptamine 1A receptor | ABAT |
| 5-hydroxytryptamine receptor 3A | HTR2C |
| Carbonic anhydrase II | penP |
| Coagulation factor VII | katA |
| Alpha-2C adrenergic receptor | ADRA2B |
| Vascular endothelial growth factor receptor 2 | SLC6A3 |
| Gamma-aminobutyric-acid receptor alpha-2 subunit | TOP1 |
| Ornithine decarboxylase | CHRM1 |
| Muscarinic acetylcholine receptor M4 | LTA4H |
| Retinoic acid receptor RXR-alpha | NOS2 |
| Delta-type opioid receptor | F10 |
| Acetylcholinesterase | DHCR7 |
| CGMP-inhibited 3',5'-cyclic phosphodiesterase A | CAT |
| Histamine H1 receptor | GOT1 |
| 5-hydroxytryptamine 2A receptor | HTR1B |
| Gamma-aminobutyric-acid receptor alpha-5 subunit | HTR1A |
| Sodium-dependent noradrenaline transporter | EGF |
| Low-density lipoprotein receptor | GSTP1 |
| Alpha-1A adrenergic receptor | AKR1B1 |
| Gamma-aminobutyric-acid receptor alpha-3 subunit | topA |
| Aspartate aminotransferase, cytoplasmic | AHR |
| 5-hydroxytryptamine 2C receptor | HTR2A |
| Progesterone receptor | NQO1 |
| Muscarinic acetylcholine receptor M2 | IL6 |
| Alpha-2B adrenergic receptor | ADRA2A |
| Alpha-1B adrenergic receptor | ADRA1A |
| Aldo-keto reductase family 1 member C3 | AKR1C1 |
| mRNA of Protein-tyrosine phosphatase, non-receptor type 1 | IFNG |
| Acetyl-CoA carboxylase 1 | ACHE |
| 72 kDa type IV collagenase | CA2 |
| Sodium-dependent dopamine transporter | KCNH2 |
| Glutathione reductase, mitochondrial | ESR1 |
| Cytosolic phospholipase A2 | PDE3A |
| Mineralocorticoid receptor | HMOX1 |
| Beta-2 adrenergic receptor | ADRB1 |
| Tumor necrosis factor | MTOR |
| Alpha-1D adrenergic receptor | ADRA1B |
| Neuronal acetylcholine receptor subunit alpha-2 | MAPK1 |
| DNA topoisomerase II | CCNA2 |
| Aldose reductase | AKR1C3 |
| Sodium-dependent serotonin transporter | PGR |
| D(2) dopamine receptor | F7 |
| Epidermal growth factor receptor | CYP3A4 |
| Mu-type opioid receptor | E |
| Multidrug resistance-associated protein 1 | IL1B |
| Estrogen receptor beta | DRD5 |
| Glucocorticoid receptor | EGFR |
| Gamma-aminobutyric acid receptor subunit alpha-1 | gyrB |
| 5-hydroxytryptamine 1B receptor | HTR1A |
| Maltase-glucoamylase, intestinal | GSK3B |
| Dipeptidyl peptidase IV | COL1A1 |
| Urokinase-type plasminogen activator | PPP3CA |
| Hepatocyte growth factor receptor | ODC1 |
| Glycogen phosphorylase, muscle form | fhuA |
| Interleukin-6 | GSR |
| Interstitial collagenase | GSTM1 |
| Mitogen-activated protein kinase 1 | pbsA1 |
| Serum paraoxonase/arylesterase 1 | PPARG |
| Cathepsin D | cphA |
| Interferon gamma | GJA1 |
| Fatty acid synthase | OPRD1 |
| Collagenase 3 | CCL2 |
| Beta-secretase | blaZ |
| Peroxisome proliferator activated receptor delta | CHRM2 |
| Mitogen-activated protein kinase 14 | MET |
| Transcription factor AP-1 | RXRB |
| C-C motif chemokine 2 | KCNMA1 |
| Interleukin-1 beta | NR3C1 |
| Mitogen-activated protein kinase 3 | HRH1 |
| Glycogen synthase kinase-3 beta | GABRA1 |
| E-selectin | camC |
| Myeloperoxidase | R |
| Cell division control protein 2 homolog | CALM1 |
| Tissue-type plasminogen activator | RXRA |
| Gap junction alpha-1 protein | EGLN1 |
| Vascular cell adhesion protein 1 | PON1 |
| Stromelysin-1 | PTGS1 |
| Heat shock protein HSP 90 | GABRE |
| Thrombomodulin | PIM1 |
| Tissue factor | RB1 |
| Neutrophil collagenase | MAPK14 |
| NAD(P)H dehydrogenase [quinone] 1 | 17 |
| Cell division protein kinase 2 | PKIA |
| Phosphatidylinositol-4,5-bisphosphate 3-kinase catalytic subunit, gamma isoform | CHRM4 |
| Beta-lactamase | ADRB2 |
| Leukotriene A-4 hydrolase | GSTM2 |
| Amine oxidase [flavin-containing] B | MAOA |
| Amine oxidase [flavin-containing] A | ADRA2C |
| Xanthine dehydrogenase/oxidase | SLC6A2 |
| Cell division protein kinase 4 | F7 |
| Neuronal acetylcholine receptor protein, alpha-7 chain | NR3C2 |
| Prostaglandin E2 receptor EP3 subtype | MT-ND6 |
| Serine/threonine-protein kinase mTOR | NCOA2 |
| Superoxide dismutase [Cu-Zn] | PTGS2 |
| Cytochrome P450 3A4 | CDK4 |
| Cellular tumor antigen p53 | CASP7 |
| Serine/threonine-protein kinase Chk1 | NCOA1 |
| Amyloid beta A4 protein | MAOB |
| mRNA of PKA Catalytic Subunit C-alpha | INSR |
| Mitogen-activated protein kinase 8 | IGHG1 |
| Cytochrome P450 1A2 | CDK2 |
| Alcohol dehydrogenase 1B | ACACA |
| Alcohol dehydrogenase 1C | ADH1B |
| Collagen alpha-1(I) chain | katB |
| Glutathione S-transferase P | FASN |
| Pro-epidermal growth factor | MPO |
| Catalase | SHV-7 |
| Vascular endothelial growth factor A | SCN5A |
| NADPH--cytochrome P450 reductase | MAP2 |
| Estrogen sulfotransferase | DRD2 |
| Retinoic acid receptor RXR-beta | NOS3 |
| Placenta growth factor | CHRM5 |
| Cytochrome P450-cam | TP53 |
| Lysozyme | PYGM |
| Ig gamma-1 chain C region | SLC6A2 |
| NADH-ubiquinone oxidoreductase chain 6 | MGAM |
| DNA topoisomerase 1 | MMP13 |
| Ferrichrome-iron receptor | DPP4 |
| DNA gyrase subunit B | COL3A1 |
| 3-hydroxy-3-methylglutaryl-coenzyme A reductase | HMGCR |
| Chymotrypsinogen B | katA |
| Heme oxygenase 1 | GABRG3 |
| Nicotinate-nucleotide--dimethylbenzimidazole phosphoribosyltransferase | MAPK3 |
| Collagen alpha-1(III) chain | CTSD |
| Retinoblastoma-associated protein | cobT |
| ATP synthase subunit beta, mitochondrial | GOT1 |
| Trypsin-1 | CHEK1 |
| Glutathione S-transferase Mu 1 | ESR2 |
| 78 kDa glucose-regulated protein | MMP2 |
| Proto-oncogene serine/threonine-protein kinase Pim-1 | MMP8 |
| Prostatic acid phosphatase | CHRNA2 |
| Cyclin-A2 | CDK1 |
| Sterol O-acyltransferase 1 | PTGER3 |
| Aryl hydrocarbon receptor | ALOX5 |
| Glutamate receptor 2 | SELE |
| Aldo-keto reductase family 1 member C1 | ADH1C |
| Nuclear receptor coactivator 2 | ABCC1 |
| Nuclear receptor coactivator 1 | ACACA |
| Gamma-aminobutyric-acid receptor subunit alpha-6 | DRD1 |
| Gamma-aminobutyric acid receptor subunit gamma-3 | gyrB |
| Egl nine homolog 1 | CYP1A2 |
| 7-dehydrocholesterol reductase | HSPA5 |
| Caspase-7 | blaB |
| cAMP-dependent protein kinase inhibitor alpha | ampC |
| Cytochrome c | CRK2 |
| Glutathione S-transferase Mu 2 | SULT1E1 |
| Calcium-activated potassium channel subunit alpha 1 | ampC |
| Calmodulin | ampC |
| Gamma-aminobutyric acid receptor subunit epsilon | gyrB |
| Serine/threonine-protein phosphatase 2B catalytic subunit alpha isoform | ODC1 |
| Interleukin-2 | GRIA2 |
| Microtubule-associated protein 2 | HSP90 |
